# Supplementary material for: Progress towards a public chemogenomic set for protein kinases and a call for contributions
Source: PLoS One. 2017 Aug 2;12(8):e0181585. doi: 10.1371/journal.pone.0181585 (PMC5540273; doi:10.1371/journal.pone.0181585)
Supplement: S1 Table — (PDF) [file pone.0181585.s001.pdf]

approved symbol	DiscoverX	ProQinase	Reaction Biology Corp	Nanosyn	Eurofins	Carna	Luceome	ThermoFisher	SignalChem	MRC
**AAK1**	yes						yes			
**AATK**										
**ABL1**	yes	yes	yes	yes	yes	yes	yes	yes	yes	Yes
**ABL2**	yes	yes	yes	yes	yes	yes	yes	yes	yes	
**ACVR1**	yes	yes	yes		yes			yes	yes	
**ACVR1B**	yes	yes	yes		yes			yes	yes	
**ACVR1C**										
**ACVR2A**	yes	yes					yes	yes		
**ACVR2B**	yes	yes					yes	yes	yes	
**ACVRL1**	yes		yes		yes			yes		
**ADCK1**										
**ADCK2**										
**ADCK5**										
**AKT1**	yes	yes	yes	yes	yes	yes	yes	yes	yes	Yes
**AKT2**	yes	yes	yes	yes	yes	yes	yes	yes	yes	Yes
**AKT3**	yes	yes	yes	yes	yes	yes	yes	yes	yes	
**ALK**	yes	yes	yes	yes	yes	yes	yes	yes	yes	
**ALPK1**										
**ALPK2**										
**ALPK3**										
**AMHR2**										
**ANKK1**	yes								yes	
**ARAF**	yes		yes							
**ATM**					yes					
**ATR**					yes					
**AURKA**	yes	yes	yes	yes	yes	yes	yes	yes	yes	Yes
**AURKB**	yes	yes	yes	yes	yes	yes	yes	yes	yes	Yes
**AURKC**	yes	yes	yes	yes	yes	yes	yes	yes	yes	
**AXL**	yes	yes	yes	yes	yes	yes	yes	yes	yes	
**BCKDK**										
**BCR**										
**BLK**	yes	yes	yes	yes	yes	yes	yes	yes	yes	
**BMP2K**	yes						yes			
**BMPR1A**	yes	yes	yes					yes	yes	
**BMPR1B**	yes		yes		yes			yes	yes	
**BMPR2**	yes		yes					yes	yes	
**BMX**	yes	yes	yes	yes	yes	yes	yes	yes	yes	
**BRAF**	yes	yes	yes	yes	yes	yes		yes	yes	
**BRSK1**	yes	yes	yes	yes	yes	yes		yes	yes	Yes
**BRSK2**	yes	yes	yes	yes	yes	yes	yes	yes	yes	Yes
**BTK**	yes	yes	yes	yes	yes	yes	yes	yes	yes	Yes
**BUB1**	yes									

**BUB1B**		yes							yes	
**CAMK1**	yes		yes	yes	yes	yes	yes	yes	yes	Yes
**CAMK1D**	yes	yes	yes	yes	yes	yes	yes	yes	yes	
**CAMK1G**	yes		yes		yes		yes		yes	
**CAMK2A**	yes	yes	yes	yes	yes	yes	yes	yes	yes	
**CAMK2B**	yes	yes	yes	yes	yes	yes	yes	yes	yes	
**CAMK2D**	yes	yes	yes	yes	yes	yes	yes	yes	yes	
**CAMK2G**	yes	yes	yes	yes	yes	yes	yes	yes	yes	
**CAMK4**	yes	yes	yes	yes	yes	yes		yes	yes	
**CAMKK1**	yes	yes	yes		yes		yes	yes	yes	
**CAMKK2**	yes	yes	yes		yes		yes	yes	yes	Yes
**CAMKV**										
**CASK**	yes							yes	yes	
**CDC42BPA**	yes	yes	yes	yes	yes	yes		yes	yes	
**CDC42BPB**	yes	yes	yes	yes	yes	yes		yes	yes	
**CDC42BPG**	yes		yes						yes	
**CDC7**		yes	yes			yes		yes	yes	
**CDK1**	yes	yes	yes	yes	yes	yes		yes	yes	
**CDK10**	yes									
**CDK11A**	yes									
**CDK11B**	yes							yes		
**CDK12**	yes	yes								
**CDK13**	yes	yes								
**CDK14**	yes							yes	yes	
**CDK15**	yes		yes							
**CDK16**	yes	yes	yes					yes	yes	
**CDK17**	yes	yes	yes						yes	
**CDK18**	yes	yes	yes						yes	
**CDK19**	yes	yes	yes							
**CDK2**	yes	yes	yes	yes	yes	yes	yes	yes	yes	Yes
**CDK20**	yes	yes								
**CDK3**	yes	yes	yes	yes	yes	yes		yes	yes	
**CDK4**	yes	yes	yes	yes	yes	yes			yes	
**CDK5**	yes	yes	yes	yes	yes	yes	yes	yes	yes	
**CDK6**	yes	yes	yes	yes	yes	yes			yes	
**CDK7**	yes	yes	yes	yes	yes	yes	yes	yes	yes	
**CDK8**	yes	yes					yes	yes	yes	
**CDK9**	yes	yes	yes	yes	yes	yes		yes	yes	Yes
**CDKL1**	yes									
**CDKL2**	yes									
**CDKL3**	yes									
**CDKL4**										
**CDKL5**	yes						yes			
**CHEK1**	yes	yes	yes	yes	yes	yes	yes	yes	yes	Yes
**CHEK2**	yes	yes	yes	yes	yes	yes		yes	yes	Yes

**CHUK**	yes	yes	yes	yes	yes	yes		yes	yes	
**CIT**	yes		yes			yes			yes	
**CLK1**	yes	yes	yes	yes	yes	yes	yes	yes	yes	
**CLK2**	yes	yes	yes	yes	yes	yes	yes	yes	yes	Yes
**CLK3**	yes	yes	yes	yes	yes	yes		yes	yes	
**CLK4**	yes	yes	yes	yes	yes			yes	yes	
**COQ8A**	yes									
**COQ8B**	yes									
**CSF1R**	yes	yes		yes	yes	yes	yes	yes		
**CSK**	yes	yes	yes	yes	yes	yes	yes	yes	yes	Yes
**CSNK1A1**	yes	yes	yes	yes		yes		yes	yes	
**CSNK1A1L**	yes		yes						yes	
**CSNK1D**	yes	yes	yes		yes	yes	yes	yes	yes	Yes
**CSNK1E**	yes	yes	yes	yes		yes		yes	yes	
**CSNK1G1**	yes	yes	yes	yes	yes	yes		yes	yes	
**CSNK1G2**	yes	yes	yes	yes	yes	yes		yes	yes	Yes
**CSNK1G3**	yes	yes	yes	yes	yes	yes		yes	yes	
**CSNK2A1**	yes	yes	yes	yes	yes	yes		yes	yes	Yes
**CSNK2A2**	yes	yes	yes		yes	yes		yes	yes	
**DAPK1**	yes	yes	yes	yes	yes	yes	yes	yes	yes	Yes
**DAPK2**	yes	yes	yes		yes		yes	yes	yes	
**DAPK3**	yes	yes	yes	yes	yes		yes	yes	yes	
**DCLK1**	yes		yes						yes	
**DCLK2**	yes	yes	yes	yes	yes	yes	yes	yes	yes	
**DCLK3**	yes				yes					
**DDR1**	yes		yes	yes	yes	yes	yes	yes	yes	
**DDR2**	yes	yes	yes	yes	yes	yes	yes	yes	yes	Yes
**DMPK**	yes	yes	yes		yes		yes	yes	yes	
**DSTYK**	yes	yes	yes						yes	
**DYRK1A**	yes	yes	yes	yes	yes	yes		yes	yes	Yes
**DYRK1B**	yes	yes	yes	yes	yes	yes		yes	yes	
**DYRK2**	yes	yes	yes	yes	yes	yes		yes	yes	Yes
**DYRK3**		yes	yes	yes	yes	yes		yes	yes	Yes
**DYRK4**		yes	yes	yes				yes	yes	
**EEF2K**		yes	yes		yes	yes		yes	yes	Yes
**EGFR**	yes	yes	yes	yes	yes	yes	yes	yes	yes	
**EIF2AK1**	yes	yes	yes						yes	
**EIF2AK2**	yes	yes	yes		yes	yes		yes	yes	
**EIF2AK3**		yes	yes	yes	yes	yes			yes	Yes
**EIF2AK4**	yes		yes		yes				yes	
**EPHA1**	yes	yes	yes	yes	yes	yes	yes	yes	yes	
**EPHA10**										
**EPHA2**	yes	yes	yes	yes	yes	yes	yes	yes	yes	Yes
**EPHA3**	yes	yes	yes	yes	yes	yes	yes	yes	yes	
**EPHA4**	yes	yes	yes	yes	yes	yes	yes	yes	yes	Yes

**EPHA5**	yes	yes	yes	yes	yes	yes	yes	yes		
**EPHA6**	yes	yes	yes			yes	yes	yes	yes	
**EPHA7**	yes	yes	yes		yes	yes	yes	yes	yes	
**EPHA8**	yes	yes	yes	yes	yes	yes	yes	yes		
**EPHB1**	yes	yes	yes	yes	yes	yes		yes	yes	Yes
**EPHB2**	yes	yes	yes	yes	yes	yes	yes	yes	yes	Yes
**EPHB3**	yes	yes	yes	yes	yes	yes	yes	yes	yes	Yes
**EPHB4**	yes	yes	yes	yes	yes	yes	yes	yes	yes	Yes
**EPHB6**	yes									
**ERBB2**	yes	yes	yes	yes	yes	yes	yes	yes		
**ERBB3**	yes									
**ERBB4**	yes	yes	yes	yes	yes	yes		yes		Yes
**ERN1**	yes		yes		yes				yes	
**ERN2**			yes						yes	
**FASTK**									yes	
**FER**	yes	yes	yes	yes	yes	yes	yes	yes	yes	
**FES**	yes	yes	yes	yes	yes	yes	yes	yes	yes	
**FGFR1**	yes	yes	yes	yes	yes	yes		yes	yes	Yes
**FGFR2**	yes	yes	yes	yes	yes	yes	yes	yes	yes	
**FGFR3**	yes	yes	yes	yes	yes	yes	yes	yes	yes	
**FGFR4**	yes	yes	yes	yes	yes	yes		yes	yes	
**FGR**	yes	yes	yes	yes	yes	yes	yes	yes	yes	
**FLT1**	yes	yes	yes	yes	yes	yes	yes	yes	yes	Yes
**FLT3**	yes	yes	yes	yes	yes	yes	yes	yes	yes	
**FLT4**	yes	yes	yes	yes	yes	yes	yes	yes	yes	
**FRK**	yes	yes	yes	yes	yes	yes	yes	yes	yes	
**FYN**	yes	yes	yes	yes	yes	yes	yes	yes	yes	
**GAK**	yes									
**GRK1**	yes		yes		yes			yes	yes	
**GRK2**	yes	yes	yes		yes			yes	yes	
**GRK3**	yes	yes	yes		yes			yes	yes	
**GRK4**	yes	yes	yes					yes		
**GRK5**		yes	yes		yes			yes	yes	
**GRK6**		yes	yes	yes	yes			yes	yes	
**GRK7**	yes	yes	yes	yes	yes		yes	yes	yes	
**GSG2**	yes	yes	yes	yes	yes	yes		yes	yes	
**GSK3A**	yes	yes	yes	yes	yes	yes	yes	yes	yes	
**GSK3B**	yes	yes	yes	yes	yes	yes		yes	yes	Yes
**GUCY2C**										
**GUCY2D**										
**GUCY2F**										
**HCK**	yes	yes	yes	yes	yes	yes	yes	yes	yes	
**HIPK1**	yes	yes	yes	yes	yes	yes		yes	yes	Yes
**HIPK2**	yes	yes	yes	yes	yes			yes	yes	Yes
**HIPK3**	yes	yes	yes	yes	yes			yes	yes	Yes

**HIPK4**	yes	yes	yes	yes	yes		yes	yes	yes	
**HUNK**	yes								yes	
**ICK**	yes		yes		yes			yes	yes	
**IGF1R**	yes	yes	yes	yes	yes	yes	yes	yes	yes	Yes
**IKBKB**	yes	yes	yes	yes	yes	yes		yes	yes	Yes
**IKBKE**	yes	yes	yes	yes	yes	yes	yes	yes	yes	Yes
**ILK**										
**INSR**	yes	yes	yes	yes	yes	yes	yes	yes	yes	Yes
**INSRR**	yes	yes	yes	yes	yes	yes	yes	yes	yes	Yes
**IRAK1**	yes	yes	yes	yes	yes	yes		yes		Yes
**IRAK2**	yes								yes	
**IRAK3**	yes						yes			
**IRAK4**	yes	yes	yes	yes	yes	yes		yes	yes	Yes
**ITK**	yes	yes	yes	yes	yes	yes	yes	yes	yes	
**JAK1**	yes	yes	yes	yes	yes	yes		yes	yes	
**JAK2**	yes	yes	yes	yes	yes	yes		yes	yes	
**JAK3**	yes	yes	yes	yes	yes	yes		yes	yes	
**KALRN**										
**KDR**	yes	yes	yes	yes	yes	yes	yes	yes	yes	
**KIT**	yes	yes	yes	yes	yes	yes	yes	yes	yes	
**KSR1**			yes						yes	
**KSR2**			yes						yes	
**LATS1**	yes		yes	yes				yes	yes	
**LATS2**	yes		yes	yes		yes		yes	yes	
**LCK**	yes	yes	yes	yes	yes	yes	yes	yes	yes	Yes
**LIMK1**	yes	yes	yes		yes		yes	yes	yes	
**LIMK2**	yes	yes	yes					yes	yes	
**LMTK2**										
**LMTK3**										
**LRRK1**										
**LRRK2**	yes	yes	yes	yes	yes			yes	yes	
**LTK**	yes	yes	yes	yes	yes	yes		yes	yes	
**LYN**	yes	yes	yes	yes	yes	yes	yes	yes	yes	
**MAK**	yes		yes						yes	
**MAP2K1**	yes	yes	yes	yes	yes	yes		yes	yes	Yes
**MAP2K2**	yes	yes	yes	yes	yes	yes		yes	yes	Yes
**MAP2K3**	yes		yes			yes		yes	yes	
**MAP2K4**	yes	yes	yes			yes			yes	
**MAP2K5**	yes	yes	yes			yes			yes	
**MAP2K6**	yes	yes	yes		yes	yes		yes	yes	Yes
**MAP2K7**	yes	yes	yes		yes	yes			yes	
**MAP3K1**	yes	yes	yes			yes			yes	Yes
**MAP3K10**	yes	yes	yes		yes	yes	yes	yes	yes	
**MAP3K11**	yes	yes	yes			yes	yes	yes	yes	Yes
**MAP3K12**	yes		yes			yes				

**MAP3K13**	yes									
**MAP3K14**	yes	yes	yes					yes		
**MAP3K15**	yes									
**MAP3K19**	yes	yes	yes						yes	
**MAP3K2**	yes	yes	yes		yes	yes		yes	yes	
**MAP3K20**	yes	yes	yes		yes			yes	yes	
**MAP3K21**		yes	yes						yes	
**MAP3K3**	yes	yes	yes			yes		yes	yes	
**MAP3K4**	yes					yes				
**MAP3K5**	yes	yes	yes		yes	yes		yes	yes	Yes
**MAP3K6**	yes		yes						yes	
**MAP3K7**	yes	yes	yes	yes	yes	yes			yes	Yes
**MAP3K8**		yes	yes			yes			yes	
**MAP3K9**	yes	yes	yes		yes	yes	yes		yes	Yes
**MAP4K1**	yes		yes						yes	
**MAP4K2**	yes	yes	yes	yes	yes	yes	yes		yes	Yes
**MAP4K3**	yes		yes						yes	Yes
**MAP4K4**	yes	yes	yes	yes	yes	yes				
**MAP4K5**	yes	yes	yes	yes	yes				yes	Yes
**MAPK1**	yes	yes	yes	yes	yes	yes		yes	yes	Yes
**MAPK10**	yes	yes	yes	yes	yes	yes		yes	yes	Yes
**MAPK11**	yes	yes	yes	yes	yes	yes		yes	yes	Yes
**MAPK12**	yes	yes	yes	yes	yes	yes	yes	yes	yes	Yes
**MAPK13**	yes	yes	yes	yes	yes	yes	yes	yes	yes	Yes
**MAPK14**	yes	yes	yes	yes	yes	yes	yes	yes	yes	Yes
**MAPK15**	yes	yes	yes					yes	yes	Yes
**MAPK3**	yes	yes	yes	yes		yes		yes	yes	Yes
**MAPK4**	yes									
**MAPK6**	yes								yes	
**MAPK7**	yes	yes	yes			yes			yes	Yes
**MAPK8**	yes	yes	yes	yes	yes	yes		yes	yes	Yes
**MAPK9**	yes	yes	yes	yes	yes	yes		yes	yes	Yes
**MAPKAPK2**	yes	yes	yes	yes	yes	yes		yes	yes	Yes
**MAPKAPK3**		yes	yes	yes	yes	yes		yes	yes	Yes
**MAPKAPK5**	yes	yes	yes	yes	yes	yes		yes	yes	Yes
**MARK1**	yes	yes	yes	yes	yes	yes	yes	yes	yes	Yes
**MARK2**	yes	yes	yes	yes	yes	yes	yes	yes	yes	Yes
**MARK3**	yes	yes	yes	yes		yes	yes	yes	yes	Yes
**MARK4**	yes	yes	yes	yes	yes	yes	yes	yes	yes	Yes
**MAST1**	yes									
**MAST2**										
**MAST3**										
**MAST4**										
**MASTL**										
**MATK**	yes	yes	yes					yes	yes	

**MELK**	yes	yes	yes	yes	yes	yes	yes	yes	yes	Yes
**MERTK**	yes	yes	yes	yes	yes	yes		yes	yes	
**MET**	yes	yes	yes	yes	yes	yes	yes	yes	yes	
**MINK1**	yes	yes	yes	yes	yes	yes		yes	yes	Yes
**MKNK1**	yes	yes	yes	yes		yes		yes	yes	Yes
**MKNK2**	yes	yes	yes		yes	yes	yes	yes	yes	Yes
**MLKL**	yes									
**MOK**					yes					
**MOS**						yes				
**MST1R**	yes	yes	yes	yes	yes	yes	yes	yes	yes	
**MTOR**	yes	yes	yes	yes	yes			yes		
**MUSK**	yes	yes	yes	yes	yes	yes	yes	yes	yes	
**MYLK**	yes	yes	yes		yes		yes	yes	yes	Yes
**MYLK2**	yes	yes	yes		yes	yes	yes	yes	yes	
**MYLK3**	yes	yes	yes						yes	
**MYLK4**	yes		yes				yes		yes	
**MYO3A**	yes		yes						yes	
**MYO3B**	yes		yes		yes			yes	yes	
**NEK1**	yes	yes	yes	yes	yes	yes		yes	yes	
**NEK10**	yes									
**NEK11**	yes	yes	yes		yes				yes	
**NEK2**	yes	yes	yes	yes	yes	yes		yes	yes	Yes
**NEK3**	yes	ye	yes		yes				yes	
**NEK4**	yes	yes	yes			yes		yes	yes	
**NEK5**	yes		yes						yes	
**NEK6**	yes	yes	yes	yes	yes	yes		yes	yes	Yes
**NEK7**	yes	yes	yes	yes	yes	yes		yes	yes	
**NEK8**			yes						yes	
**NEK9**	yes	yes	yes	yes	yes	yes		yes	yes	
**NIM1K**	yes		yes		yes	yes			yes	
**NLK**	yes	yes	yes		yes			yes	yes	
**NPR1**										
**NPR2**										
**NRBP1**										
**NRBP2**										
**NRK**										
**NTRK1**	yes	yes	yes	yes	yes	yes		yes	yes	Yes
**NTRK2**	yes	yes	yes	yes	yes	yes	yes	yes	yes	
**NTRK3**	yes	yes	yes	yes	yes	yes	yes	yes	yes	
**NUAK1**	yes	yes	yes	yes	yes	yes	yes	yes	yes	Yes
**NUAK2**	yes	yes	yes		yes	yes	yes	yes	yes	
**OBSCN**										
**OXSR1**	yes		yes							Yes
**PAK1**	yes	yes	yes	yes	yes	yes	yes	yes	yes	
**PAK2**	yes	yes	yes	yes	yes	yes		yes		Yes

**PAK3**	yes	yes	yes	yes	yes			yes	yes	
**PAK4**	yes	yes	yes	yes	yes	yes		yes	yes	Yes
**PAK5**	yes	yes	yes	yes	yes	yes		yes	yes	Yes
**PAK6**	yes	yes	yes	yes	yes	yes		yes	yes	Yes
**PASK**	yes	yes	yes	yes	yes	yes		yes	yes	
**PBK**		yes	yes			yes			yes	
**PDGFRA**	yes	yes	yes	yes	yes	yes	yes	yes	yes	Yes
**PDGFRB**	yes	yes	yes	yes	yes	yes	yes	yes	yes	
**PDIK1L**										
**PDK1**			yes	yes	yes			yes	yes	Yes
**PDK2**			yes			yes			yes	
**PDK3**			yes						yes	
**PDK4**			yes		yes	yes			yes	
**PDPK1**	yes	yes				yes	yes		yes	
**PEAK1**			yes						yes	
**PHKG1**	yes	yes	yes	yes		yes	yes	yes	yes	Yes
**PHKG2**	yes	yes	yes	yes	yes	yes		yes	yes	
**PIM1**	yes	yes	yes	yes	yes	yes	yes	yes	yes	Yes
**PIM2**	yes	yes	yes		yes	yes	yes	yes	yes	Yes
**PIM3**	yes	yes	yes	yes	yes	yes			yes	Yes
**PINK1**										Yes
**PKDCC**										
**PKMYT1**	yes	yes					yes		yes	
**PKN1**	yes	yes	yes	yes		yes		yes	yes	
**PKN2**	yes	yes	yes	yes	yes			yes	yes	Yes
**PKN3**		yes	yes				yes		yes	
**PLK1**	yes	yes	yes	yes	yes	yes		yes	yes	Yes
**PLK2**	yes	yes	yes	yes	yes	yes		yes	yes	
**PLK3**	yes	yes	yes	yes	yes	yes		yes	yes	
**PLK4**	yes	yes	yes	yes			yes	yes	yes	
**PNCK**	yes		yes		yes				yes	
**POMK**										
**PRAG1**										
**PRKAA1**	yes	yes	yes	yes	yes	yes	yes	yes	yes	Yes
**PRKAA2**	yes		yes	yes	yes	yes	yes	yes	yes	Yes
**PRKACA**	yes	yes	yes	yes	yes	yes	yes	yes	yes	Yes
**PRKACB**	yes		yes	yes	yes	yes	yes	yes	yes	
**PRKACG**			yes			yes		yes	yes	
**PRKCA**		yes	yes	yes	yes	yes		yes	yes	Yes
**PRKCB**		yes	yes	yes	yes	yes		yes	yes	
**PRKCD**	yes	yes	yes		yes	yes	yes	yes	yes	
**PRKCE**	yes	yes	yes	yes	yes	yes	yes	yes	yes	
**PRKCG**		yes	yes	yes	yes	yes	yes	yes	yes	Yes
**PRKCH**	yes	yes	yes	yes	yes	yes	yes	yes	yes	
**PRKCI**	yes	yes	yes	yes	yes	yes		yes	yes	

**PRKCQ**	yes	yes	yes	yes	yes	yes	yes	yes	yes	
**PRKCZ**		yes	yes	yes	yes	yes		yes	yes	Yes
**PRKD1**	yes	yes	yes	yes	yes	yes		yes		Yes
**PRKD2**	yes	yes	yes	yes	yes	yes	yes	yes	yes	
**PRKD3**	yes	yes	yes	yes	yes	yes	yes	yes		
**PRKDC**		yes	yes		yes			yes		
**PRKG1**	yes	yes	yes	yes	yes	yes	yes	yes	yes	
**PRKG2**	yes	yes	yes	yes	yes	yes	yes	yes	yes	
**PRKX**	yes	yes	yes	yes	yes	yes	yes	yes	yes	
**PRKY**										
**PRPF4B**	yes									
**PSKH1**										
**PSKH2**										
**PTK2**	yes	yes	yes	yes	yes	yes	yes	yes	yes	
**PTK2B**	yes	yes	yes	yes	yes	yes	yes	yes	yes	
**PTK6**	yes	yes	yes	yes		yes	yes	yes	yes	Yes
**PTK7**										
**PXK**										
**RAF1**	yes	yes	yes	yes	yes	yes		yes	yes	
**RET**	yes	yes	yes	yes	yes	yes	yes	yes		
**RIOK1**	yes									
**RIOK2**	yes									
**RIOK3**	yes									
**RIPK1**	yes								yes	
**RIPK2**	yes	yes	yes	yes	yes		yes	yes	yes	Yes
**RIPK3**	yes		yes					yes	yes	
**RIPK4**	yes	yes	yes						yes	
**RNASEL**										
**ROCK1**	yes	yes	yes	yes	yes	yes		yes	yes	
**ROCK2**	yes	yes	yes	yes	yes	yes		yes	yes	Yes
**ROR1**									yes	
**ROR2**									yes	
**ROS1**	yes	yes	yes	yes	yes	yes	yes	yes	yes	
**RPS6KA1**	yes	yes	yes	yes		yes	yes	yes	yes	
**RPS6KA2**	yes	yes	yes	yes		yes	yes	yes	yes	Yes
**RPS6KA3**	yes	yes	yes	yes		yes	yes	yes	yes	Yes
**RPS6KA4**	yes	yes	yes	yes	yes	yes	yes	yes	yes	
**RPS6KA5**	yes	yes	yes	yes	yes	yes	yes	yes	yes	Yes
**RPS6KA6**	yes	yes	yes	yes		yes	yes	yes	yes	
**RPS6KB1**	yes	yes	yes	yes	yes	yes		yes	yes	Yes
**RPS6KB2**		yes	yes	yes		yes			yes	
**RPS6KC1**										
**RPS6KL1**										
**RYK**										
**SBK1**	yes		yes						yes	

**SBK2**										
**SBK3**	yes									
**SCYL1**										
**SCYL2**										
**SCYL3**										
**SGK1**	yes	yes	yes	yes	yes	yes		yes	yes	Yes
**SGK2**	yes	yes	yes	yes	yes	yes	yes	yes	yes	
**SGK3**	yes	yes	yes	yes	yes	yes	yes	yes	yes	
**SGK494**										
**SIK1**	yes	yes	yes	yes	yes	yes	yes	yes	yes	
**SIK2**	yes	yes	yes	yes	yes	yes	yes	yes	yes	Yes
**SIK3**	yes	yes	yes		yes		yes	yes	yes	Yes
**SLK**	yes	yes	yes	yes	yes	yes	yes	yes	yes	
**SMG1**										
**SNRK**	yes		yes		yes				yes	
**SPEG**										
**SRC**	yes	yes	yes	yes	yes	yes	yes	yes	yes	Yes
**SRMS**	yes	yes	yes	yes		yes	yes	yes	yes	
**SRPK1**	yes	yes	yes	yes	yes	yes		yes	yes	Yes
**SRPK2**	yes	yes	yes	yes	yes	yes		yes	yes	
**SRPK3**	yes	yes	yes	yes	yes	yes		yes	yes	
**STK10**	yes		yes	yes	yes	yes	yes		yes	
**STK11**	yes		yes		yes					Yes
**STK16**	yes	yes	yes	yes			yes	yes		Yes
**STK17A**	yes	yes	yes		yes		yes	yes	yes	
**STK17B**	yes						yes	yes	yes	
**STK19**									yes	
**STK24**	yes	yes	yes	yes	yes	yes		yes	yes	Yes
**STK25**		yes	yes		yes		yes	yes	yes	
**STK26**	yes	yes	yes		yes	yes		yes	yes	Yes
**STK3**	yes	yes	yes	yes	yes	yes	yes	yes	yes	Yes
**STK31**										
**STK32A**	yes									
**STK32B**	yes		yes				yes	yes	yes	
**STK32C**	yes		yes					yes	yes	
**STK33**	yes	yes	yes		yes		yes	yes	yes	Yes
**STK35**	yes				yes					
**STK36**	yes	yes							yes	
**STK38**	yes		yes			yes		yes	yes	
**STK38L**	yes		yes	yes		yes		yes	yes	
**STK39**	yes	yes	yes						yes	
**STK4**	yes	yes	yes	yes	yes	yes		yes	yes	
**STK40**										
**STKLD1**										
**STRADA**										

**STRADB**										
**STYK1**										
**SYK**	yes	yes	yes	yes	yes	yes	yes	yes	yes	Yes
**TAF1**										
**TAOK1**	yes		yes		yes			yes	yes	Yes
**TAOK2**	yes	yes	yes	yes	yes	yes		yes	yes	
**TAOK3**	yes	yes	yes	yes	yes			yes	yes	
**TBCK**										
**TBK1**	yes	yes	yes	yes	yes	yes	yes	yes	yes	Yes
**TEC**	yes	yes	yes	yes	yes	yes	yes	yes	yes	
**TEK**	yes	yes	yes		yes	yes	yes	yes	yes	Yes
**TESK1**	yes		yes				yes		yes	Yes
**TESK2**			yes				yes	yes	yes	
**TEX14**										
**TGFBR1**	yes	yes	yes		yes			yes	yes	Yes
**TGFBR2**	yes	yes	yes					yes	yes	
**TIE1**	yes						yes			
**TLK1**	yes	yes	yes		yes			yes	yes	Yes
**TLK2**	yes	yes	yes		yes			yes	yes	
**TNIK**	yes		yes	yes	yes	yes		yes	yes	
**TNK1**	yes	yes	yes			yes	yes		yes	
**TNK2**	yes	yes	yes	yes	yes	yes	yes	yes	yes	
**TNNI3K**	yes						yes			
**TP53RK**										
**TRIB1**										
**TRIB2**										
**TRIB3**										
**TRIO**									yes	
**TRPM6**	yes									
**TRPM7**			yes		yes					
**TRRAP**										
**TSSK1B**	yes	yes	yes	yes	yes	yes		yes	yes	Yes
**TSSK2**		yes	yes	yes	yes	yes		yes	yes	
**TSSK3**	yes		yes		yes	yes				
**TSSK4**					yes					
**TSSK6**			yes							
**TTBK1**		yes	yes		yes				yes	Yes
**TTBK2**		yes	yes		yes				yes	Yes
**TTK**	yes	yes		yes	yes			yes	yes	Yes
**TTN**										
**TXK**	yes	yes	yes	yes	yes	yes	yes	yes	yes	
**TYK2**	yes	yes	yes	yes	yes	yes		yes	yes	
**TYRO3**	yes	yes	yes	yes	yes	yes		yes	yes	
**UHMK1**										
**ULK1**	yes		yes		yes			yes	yes	Yes

**ULK2**	yes		yes		yes			yes	yes	Yes
**ULK3**	yes		yes		yes			yes	yes	
**ULK4**										
**VRK1**		yes	yes						yes	
**VRK2**	yes	yes	yes		yes				yes	
**VRK3**										
**WEE1**	yes	yes	yes		yes			yes	yes	
**WEE2**	yes									
**WNK1**	yes	yes	yes			yes			yes	Yes
**WNK2**	yes	yes	yes		yes	yes		yes	yes	
**WNK3**	yes	yes	yes		yes	yes		yes		
**WNK4**	yes								yes	
**YES1**	yes	yes	yes	yes	yes	yes	yes	yes	yes	Yes
**ZAP70**	yes	yes	yes	yes	yes			yes	yes	Yes
**SGK424**										
