# Supplementary material for: Progress towards a public chemogenomic set for protein kinases and a call for contributions
Source: PLoS One. 2017 Aug 2;12(8):e0181585. doi: 10.1371/journal.pone.0181585 (PMC5540273; doi:10.1371/journal.pone.0181585)
Supplement: S2 Table — (PDF) [file pone.0181585.s002.pdf]

Compound Name	Chemotype	GINI Criteria Met	Entropy Criteria Met	S(10) Criteria Met	Entropy 1000nM	GINI 1000nM	S(10)	# kinases >90% Inh 1000nM	Kinases >90%Inh 1000nM
GW589961A	Benzimidazolyl_diaryl_ureas	Y	N	Y	3.073	0.773	0.005	1	[KIT]
GW700494A	Benzimidazolyl_diaryl_ureas	Y	N	Y	2.992	0.769	0.005	1	[KIT]
GSK1511931A	2,4-dianilino_pyrrolopyrimidines	Y	N	Y	2.625	0.763	0.005	1	[PYK2]
SB-675259-M	3-amino_pyrazolopyridazines	Y	N	Y	2.822	0.820	0.010	2	[CDK2/cyclinA, DYRK1A]
GSK2186269A	2,4-dianilino_pyrrolopyrimidines	Y	N	Y	2.001	0.762	0.015	3	[IGF1R, INSR, IRR]
GSK614526A	Furazan_benzimidazoles	Y	N	Y	2.309	0.769	0.021	4	[AKT1, IKKE, AKT3, PKC-eta]
SB-630812	4-pyridyl_ortho-aryl_azoles	N	Y	Y	1.639	0.741	0.005	1	[LOK]
GSK2219385A	2,4-dianilino_pyrrolopyrimidines	N	Y	Y	0.503	0.740	0.005	1	[INSR]
GSK317315A	Benzimidazole_N-thiophenes	N	Y	Y	0.814	0.726	0.005	1	[PLK1]
GSK200398A	Anilino_thienopyrimidines	N	Y	Y	0.343	0.723	0.005	1	[ERBB4]
GSK238583A	Anilino_thienopyrimidines	N	Y	Y	0.372	0.678	0.005	1	[ERBB4]
GSK317314A	Benzimidazole_N-thiophenes	N	Y	Y	1.624	0.660	0.005	1	[PLK1]
GW280670X	Oxindoles	N	Y	Y	1.231	0.639	0.005	1	[CDK2/cyclinA]
GW572399X	2-amino_oxazoles	N	Y	Y	1.247	0.611	0.005	1	[CDK2/cyclinA]
GSK182497A	Anilino_thienopyrimidines	N	Y	Y	0.481	0.597	0.005	1	[ERBB4]
GW577921A	2-amino_oxazoles	N	Y	Y	1.589	0.577	0.005	1	[KIT]
GSK571989A	Benzimidazole_N-thiophenes	N	Y	Y	1.563	0.567	0.005	1	[PLK1]
GW301888X	4-anilino_quinazolines_and_related	N	Y	Y	0.471	0.557	0.005	1	[ERBB4]
GR269666A	4-anilino_quinazolines_and_related	N	Y	Y	0.527	0.506	0.005	1	[ERBB4]
GW827106X	2-aryl_3-pyridimidinyl_pyrazolopyridazines	N	Y	Y	1.468	0.745	0.010	2	[ERBB4, YES]
GW694234A	Benzimidazolyl_diaryl_ureas	N	Y	Y	1.222	0.697	0.010	2	[DDR2, KIT]
GSK238063A	Anilino_thienopyrimidines	N	Y	Y	0.433	0.683	0.010	2	[EGFR, ERBB4]
GSK192082A	Anilino_thienopyrimidines	N	Y	Y	0.770	0.594	0.010	2	[EGFR, ERBB4]
GSK326090A	Benzimidazole_N-thiophenes	N	Y	Y	1.514	0.735	0.015	3	[NEK9, PLK1, LOK]
GSK1392956A	2,4-dianilino_pyrrolopyrimidines	N	Y	Y	1.591	0.680	0.015	3	[IGF1R, INSR, PYK2]
GW770249X	Furopyrimidines_and_related	N	Y	Y	1.574	0.714	0.021	4	[PDGFRA, MUSK, KIT, TRKA]
GSK619487A	Furazan_benzimidazoles	N	Y	N	0.823	0.679	0.026	5	[AKT1, IKKE, AKT3, PKC-eta, AKT2]
GSK2213727A	2,4-dianilino_pyrrolopyrimidines	N	Y	N	1.082	0.723	0.036	7	[IGF1R, PYK2, LTK, IRR, PLK1, LRRK2, INSR]
GSK2110236A	2,4-dianilino_pyrrolopyrimidines	N	Y	N	1.156	0.743	0.041	8	[IGF1R, LTK, PYK2, IRR, PLK1, TSSK2, TSSK1, INSR]
GW693917A	Benzimidazolyl_diaryl_ureas	N	N	Y	3.475	0.749	0.005	1	[EPHA2]
GSK248233A	Furazan_benzimidazoles	N	N	Y	3.390	0.737	0.005	1	[p70s6K1]
GW678313X	2-amino_oxazoles	N	N	Y	3.423	0.737	0.005	1	[KIT]
GW683134A	Benzimidazolyl_diaryl_ureas	N	N	Y	3.633	0.730	0.005	1	[KIT]
GSK1751853A	2,4-dianilino_pyrrolopyrimidines	N	N	Y	3.019	0.702	0.005	1	[PYK2]
SB-220025-A	4-pyrimidinyl_ortho-aryl_azoles	N	N	Y	2.653	0.701	0.005	1	[P38alpha]
GW435821X	3-vinyl_pyridines	N	N	Y	3.455	0.697	0.005	1	[IKKE]
GW819077X	2H-3_pyrimidinyl_pyrazolopyridazines	N	N	Y	2.763	0.693	0.005	1	[CLK2]

GW804482X	Benzimidazole_N-thiophenes	N	N	Y	1.815	0.691	0.005	1	[PLK1]
GW301784X	Oxindoles	N	N	Y	3.679	0.681	0.005	1	[CDK2/cyclinA]
GW282449A	4-anilino_quinazolines_and_related	N	N	Y	3.064	0.681	0.005	1	[ERBB4]
GW632580X	3-benzyl_pyrimidines	N	N	Y	1.787	0.681	0.005	1	[FMS]
GSK299115A	Indazole-5-carboxamides	N	N	Y	3.115	0.675	0.005	1	[ROCK1]
GW576924A	4-anilino_quinazolines_and_related	N	N	Y	2.404	0.674	0.005	1	[EGFR]
SB-236687	4-pyrimidinyl_ortho-aryl_azoles	N	N	Y	3.403	0.672	0.005	1	[P38alpha]
GW833373X	2-aryl_3-pyridimidinyl_pyrazolopyridazines	N	N	Y	3.738	0.670	0.005	1	[YES]
GW335962X	Oxindoles	N	N	Y	3.557	0.669	0.005	1	[KIT]
GW439255X	3-vinyl_pyridines	N	N	Y	3.399	0.665	0.005	1	[IKKE]
SB-772077-B	Furazan_benzimidazoles	N	N	Y	3.542	0.661	0.005	1	[PIM1]
GW575533A	2-amino_oxazoles	N	N	Y	3.220	0.659	0.005	1	[KIT]
GW807982X	2H-3_pyrimidinyl_pyrazolopyridazines	N	N	Y	2.237	0.655	0.005	1	[CLK2]
GW282974X	4-anilino_quinazolines_and_related	N	N	Y	2.519	0.654	0.005	1	[ERBB4]
GW811761X	2H-3_pyrimidinyl_pyrazolopyridazines	N	N	Y	2.380	0.649	0.005	1	[CLK2]
GW843682X	Benzimidazole_N-thiophenes	N	N	Y	3.030	0.644	0.005	1	[PLK1]
GW709042A	Benzimidazolyl_diaryl_ureas	N	N	Y	3.738	0.643	0.005	1	[RET]
GSK270822A	2Indazole-5-carboxamides	N	N	Y	3.762	0.640	0.005	1	[ROCK1]
GW829906X	2-aryl_3-pyridimidinyl_pyrazolopyridazines	N	N	Y	3.831	0.639	0.005	1	[YES]
GW276655X	Oxindoles	N	N	Y	3.655	0.639	0.005	1	[CDK2/cyclinA]
GW830900A	2,4-diamino_pyrimidines	N	N	Y	3.138	0.636	0.005	1	[Aurora-C]
GSK949675A	Furazan_benzimidazoles	N	N	Y	2.953	0.632	0.005	1	[PKC-eta]
GW830263A	2,4-diamino_pyrimidines	N	N	Y	3.714	0.628	0.005	1	[Aurora-C]
GW805758X	2H-3_pyrimidinyl_pyrazolopyridazines	N	N	Y	3.547	0.621	0.005	1	[KIT]
GW569293E	4-pyrimidinyl_ortho-aryl_azoles	N	N	Y	3.057	0.620	0.005	1	[P38alpha]
GW513184X	4-hydrazinly_pyrazolopyrimidines	N	N	Y	3.826	0.616	0.005	1	[Aurora-A]
GSK237700A	Benzimidazole_N-thiophenes	N	N	Y	2.742	0.607	0.005	1	[PLK1]
SB-242717	4-pyrimidinyl_ortho-aryl_azoles	N	N	Y	2.566	0.601	0.005	1	[P38alpha]
GW406108X	Oxindoles	N	N	Y	3.719	0.585	0.005	1	[CLK2]
GW814408X	4-hydrazinly_pyrazolopyrimidines	N	N	Y	4.057	0.581	0.005	1	[Aurora-C]
SB-278538	4-pyrimidinyl_ortho-aryl_azoles	N	N	Y	2.871	0.581	0.005	1	[P38alpha]
GW784307A	4-hydrazinly_pyrazolopyrimidines	N	N	Y	4.132	0.569	0.005	1	[LCK]
GW305074X	Oxindoles	N	N	Y	2.369	0.569	0.005	1	[MAPK3]
GW784752X	4-hydrazinly_pyrazolopyrimidines	N	N	Y	4.108	0.565	0.005	1	[GSK3A]
SB-633825	4-pyridyl_ortho-aryl_azoles	N	N	Y	2.704	0.557	0.005	1	[LOK]
GW290597X	Oxindoles	N	N	Y	3.176	0.540	0.005	1	[CDK2/cyclinA]
SB-284847-BT	4-pyrimidinyl_ortho-aryl_azoles	N	N	Y	2.314	0.535	0.005	1	[P38alpha]
SB-245392	4-pyrimidinyl_ortho-aryl_azoles	N	N	Y	2.516	0.528	0.005	1	[P38alpha]
SB-242719	4-pyrimidinyl_ortho-aryl_azoles	N	N	Y	3.182	0.513	0.005	1	[P38alpha]
GW576609A	4-anilino_quinazolines_and_related	N	N	Y	3.035	0.511	0.005	1	[ERBB4]
GW578748X	4-hydrazinly_pyrazolopyrimidines	N	N	Y	3.793	0.507	0.005	1	[IKKE]
GW853606X	Benzimidazole_N-thiophenes	N	N	Y	2.335	0.504	0.005	1	[PLK1]
GSK1023156A	Benzimidazole_N-thiophenes	N	N	Y	2.569	0.459	0.005	1	[PLK1]
SB-657836-AAA	3-cyano_thiophenes	N	N	Y	3.007	0.419	0.005	1	[IKKE]
GSK978744A	Benzimidazole_N-thiophenes	N	N	Y	2.689	0.749	0.010	2	[NEK9, PLK1]
SB-223133	4-pyrimidinyl_ortho-aryl_azoles	N	N	Y	3.041	0.743	0.010	2	[CK1a, P38alpha]
GW673715X	Benzimidazolyl_diaryl_ureas	N	N	Y	2.603	0.737	0.010	2	[PDGFRA, KIT]
GW627512B	2-amino_oxazoles	N	N	Y	1.950	0.726	0.010	2	[FLT3, KIT]
GW654652C	2,4-diamino_pyrimidines	N	N	Y	1.784	0.694	0.010	2	[FLT4, FLT1]
GW580509X	2-amino_oxazoles	N	N	Y	3.325	0.682	0.010	2	[TNK1, KIT]
GSK1713088A	2,4-dianilino_pyrrolopyrimidines	N	N	Y	3.020	0.678	0.010	2	[IGF1R, PYK2]
GSK554170A	Furazan_benzimidazoles	N	N	Y	2.143	0.674	0.010	2	[PKC-eta, IKKE]

GSK579289A	Benzimidazole_N-thiophenes	N	N	Y	2.432	0.664	0.010	2	[NEK9, PLK1]
SB-698596-AC	3-amino_pyrazolopyridazines	N	N	Y	1.896	0.657	0.010	2	[GSK3B, GSK3A]
GW770220A	2,4-diamino_pyrimidines	N	N	Y	3.623	0.645	0.010	2	[Aurora-C, FMS]
GW429374A	Oxindoles	N	N	Y	3.321	0.644	0.010	2	[IKKE, MAP4K4]
GSK312948A	Benzimidazole_N-thiophenes	N	N	Y	3.200	0.625	0.010	2	[PLK1, LOK]
GW784684X	1Anilino_thienopyrimidines	N	N	Y	3.183	0.619	0.010	2	[ERBB4]
GW784684X	1Anilino_thienopyrimidines	N	N	Y	3.183	0.619	0.010	2	[ERBB4]
GW621970X	2-amino_oxazoles	N	N	Y	3.292	0.614	0.010	2	[YES, KIT]
GW442130X	Oxindoles	N	N	Y	2.911	0.604	0.010	2	[Aurora-A, TRKC]
GW458787A	4-anilino_quinazolines_and_related	N	N	Y	1.930	0.598	0.010	2	[EGFR, ERBB4]
GW631581B	2-amino_oxazoles	N	N	Y	2.441	0.592	0.010	2	[FLT3, KIT]
GW810372X	2H-3_pyrimidinyl_pyrazolopyridazines	N	N	Y	2.448	0.587	0.010	2	[HIPK1, CLK2]
GW780056X	2H-3_pyrimidinyl_pyrazolopyridazines	N	N	Y	3.898	0.581	0.010	2	[CLK2, KIT]
GR105659X	Oxindoles	N	N	Y	3.723	0.580	0.010	2	[Aurora-C, KIT]
GW566221A	4-anilino_quinazolines_and_related	N	N	Y	2.342	0.554	0.010	2	[EGFR, ERBB4]
GW784684X	1Anilino_thienopyrimidines	N	N	Y	3.183	0.539	0.010	2	[ERBB4]
GW784684X	1Anilino_thienopyrimidines	N	N	Y	3.183	0.539	0.010	2	[ERBB4]
SB-725317	3-amino_pyrazolopyridines	N	N	Y	2.201	0.520	0.010	2	[CDK2/cyclinA, LTK]
GW795486X	1Furopyrimidines_and_related	N	N	Y	3.440	0.695	0.015	3	[FMS, TRKA, KIT]
GW852849X	Benzimidazole_N-thiophenes	N	N	Y	1.915	0.674	0.015	3	[PDGFRA, PLK1, LOK]
GW876790X	Furazan_benzimidazoles	N	N	Y	3.490	0.655	0.015	3	[ROCK1, ROCK2, PKC-eta]
SB-736302	Furazan_benzimidazoles	N	N	Y	4.300	0.641	0.015	3	[PKC-eta, RSK3]
SB-736302	Furazan_benzimidazoles	N	N	Y	4.300	0.641	0.015	3	[PKC-eta, RSK3]
GW410563A	4-anilino_quinazolines_and_related	N	N	Y	1.661	0.639	0.015	3	[PDGFRA, FLT4, FLT1]
GSK1819799A	2,4-dianilino_pyrrolopyrimidines	N	N	Y	2.826	0.637	0.015	3	[INSR, LTK, PYK2]
SB-736302	Furazan_benzimidazoles	N	N	Y	4.300	0.628	0.015	3	[PKC-eta, RSK3]
SB-736302	Furazan_benzimidazoles	N	N	Y	4.300	0.628	0.015	3	[PKC-eta, RSK3]
GW300657X	Oxindoles	N	N	Y	1.928	0.601	0.015	3	[CDK2/cyclinA, CDK3/cyclinE, MELK]
GSK319347A	Benzimidazole_N-thiophenes	N	N	Y	2.462	0.593	0.015	3	[IKKE, MAP4K4, Aurora-C]
GW416981X	Oxindoles	N	N	Y	4.016	0.585	0.015	3	[CDK3/cyclinE, IKKE, MELK]
GW300660X	Oxindoles	N	N	Y	3.382	0.576	0.015	3	[CDK2/cyclinE, CLK2, MELK]
GW296115X	Maleimide	N	N	Y	2.762	0.527	0.015	3	[BRSK2, BRSK1, RSK3]
SB-734117	Furazan_benzimidazoles	N	N	Y	3.499	0.722	0.021	4	[GSK3A, GSK3B, RSK3, p70s6K1]

GSK938890A	Furazan_benzimidazoles	N	N	Y	3.065	0.718	0.021	4	[PAK6, PKC-eta, ARK5, AKT2]
GSK269962B	Furazan_benzimidazoles	N	N	Y	3.185	0.690	0.021	4	[ROCK1, ROCK2, PKC-eta, p70s6K1]
GW809897X	2,4-diamino_pyrimidines	N	N	Y	2.300	0.671	0.021	4	[Aurora-A, Aurora-C, FMS, KIT]
GW771127A	2,4-diamino_pyrimidines	N	N	Y	3.209	0.666	0.021	4	[FMS, KIT, FLT1, FLT4]
GW827099X	2-aryl_3-pyridimidinyl_pyrazolopyridazines	N	N	Y	3.550	0.659	0.021	4	[YES, KIT, ERBB4, P38alpha]
GW396574X	Oxindoles	N	N	Y	2.563	0.640	0.021	4	[Aurora-A, CDK2/cyclinA, CDK3/cyclinE, CDK6/cyclinD3]
SB-750140	Furazan_benzimidazoles	N	N	Y	3.603	0.598	0.021	4	[RSK3, ROCK1, ROCK2, MRCKA]
GSK237701A	Benzimidazole_N-thiophenes	N	N	Y	2.009	0.584	0.021	4	[PDGFRA, NEK9, PDGFRB, PLK1]
GW297361X	Oxindoles	N	N	Y	4.101	0.507	0.021	4	[CDK2/cyclinA, CDK3/cyclinE, FLT4, KIT]
