# Supplementary material for: Progress towards a public chemogenomic set for protein kinases and a call for contributions
Source: PLoS One. 2017 Aug 2;12(8):e0181585. doi: 10.1371/journal.pone.0181585 (PMC5540273; doi:10.1371/journal.pone.0181585)
Supplement: S3 Table — (PDF) [file pone.0181585.s003.pdf]

Compound Name	Smiles	%Inh >90	DiscoveRx Gene Symbol	Entrez Gene Symbol	Modifier	Kd (nM)
GSK2358994	Clc1cccc(Cn2c(nn3c2nc(cc3=O)N2CCOCC2)C2CC2)c1Cl	2	PIK3CB	PIK3CB	=	0.94
GSK2358994	Clc1cccc(Cn2c(nn3c2nc(cc3=O)N2CCOCC2)C2CC2)c1Cl	2	PIK3CD	PIK3CD	=	820
GSK2358994	Clc1cccc(Cn2c(nn3c2nc(cc3=O)N2CCOCC2)C2CC2)c1Cl	2	VPS34	PIK3C3	=	0.39
GSK350559	CS(=O)(=O)Cc1ccc(Nc2nccc(n2)-c2c(nn3ncccc23)-c2cccc(NC(=O)C3CCCCC3)c2)cc1	8	BUB1	BUB1	=	29
GSK350559	CS(=O)(=O)Cc1ccc(Nc2nccc(n2)-c2c(nn3ncccc23)-c2cccc(NC(=O)C3CCCCC3)c2)cc1	8	DDR1	DDR1	=	23
GSK350559	CS(=O)(=O)Cc1ccc(Nc2nccc(n2)-c2c(nn3ncccc23)-c2cccc(NC(=O)C3CCCCC3)c2)cc1	8	FLT3	FLT3	=	74
GSK350559	CS(=O)(=O)Cc1ccc(Nc2nccc(n2)-c2c(nn3ncccc23)-c2cccc(NC(=O)C3CCCCC3)c2)cc1	8	KIT	KIT	=	110
GSK350559	CS(=O)(=O)Cc1ccc(Nc2nccc(n2)-c2c(nn3ncccc23)-c2cccc(NC(=O)C3CCCCC3)c2)cc1	8	LOK	STK10	=	360
GSK350559	CS(=O)(=O)Cc1ccc(Nc2nccc(n2)-c2c(nn3ncccc23)-c2cccc(NC(=O)C3CCCCC3)c2)cc1	8	MEK5	MAP2K5	=	35
GSK350559	CS(=O)(=O)Cc1ccc(Nc2nccc(n2)-c2c(nn3ncccc23)-c2cccc(NC(=O)C3CCCCC3)c2)cc1	8	PDGFRB	PDGFRB	=	74
GSK350559	CS(=O)(=O)Cc1ccc(Nc2nccc(n2)-c2c(nn3ncccc23)-c2cccc(NC(=O)C3CCCCC3)c2)cc1	8	PIP5K1C	PIP5K1C	>	10000
GSK350559	CS(=O)(=O)Cc1ccc(Nc2nccc(n2)-c2c(nn3ncccc23)-c2cccc(NC(=O)C3CCCCC3)c2)cc1	8	PIP5K2C	PIP4K2C	=	60
GSK350559	CS(=O)(=O)Cc1ccc(Nc2nccc(n2)-c2c(nn3ncccc23)-c2cccc(NC(=O)C3CCCCC3)c2)cc1	8	STK36	STK36	=	67
GSK955403	Cc1ccc(NC(=O)Nc2cccc(c2)C(F)(F)F)cc1	4	ACVR1B	ACVR1B	>	10000
GSK955403	Cc1ccc(NC(=O)Nc2cccc(c2)C(F)(F)F)cc1	4	BMPR1A	BMPR1A	>	10000
GSK955403	Cc1ccc(NC(=O)Nc2cccc(c2)C(F)(F)F)cc1	4	IRAK3	IRAK3	>	10000
GSK955403	Cc1ccc(NC(=O)Nc2cccc(c2)C(F)(F)F)cc1	4	KIT	KIT	=	240
GW345098	CCn1cnc2c(N)ncnc12	0	TRPM6	TRPM6	=	240
GW630823	Cc1ccc(O)cc1Nc1cc(nc2ccc(cc12)-c1ccncc1)C(F)(F)F	1	EPHA7	EPHA7	>	10000
GW630823	Cc1ccc(O)cc1Nc1cc(nc2ccc(cc12)-c1ccncc1)C(F)(F)F	1	TEC	TEC	>	10000
GW857175	Cc1cccc(n1)-c1nc(Nc2ccc3[nH]ncc3c2)c2ccccc2n1	7	ACVR1B	ACVR1B	=	54
GW857175	Cc1cccc(n1)-c1nc(Nc2ccc3[nH]ncc3c2)c2ccccc2n1	7	CSNK2A1	CSNK2A1	=	100
GW857175	Cc1cccc(n1)-c1nc(Nc2ccc3[nH]ncc3c2)c2ccccc2n1	7	CSNK2A2	CSNK2A2	=	42
GW857175	Cc1cccc(n1)-c1nc(Nc2ccc3[nH]ncc3c2)c2ccccc2n1	7	JAK1(JH2do	JAK1	=	650
GW857175	Cc1cccc(n1)-c1nc(Nc2ccc3[nH]ncc3c2)c2ccccc2n1	7	PIKFYVE	PIKFYVE	=	700
GW857175	Cc1cccc(n1)-c1nc(Nc2ccc3[nH]ncc3c2)c2ccccc2n1	7	PRKD1	PRKD1	=	19
GW857175	Cc1cccc(n1)-c1nc(Nc2ccc3[nH]ncc3c2)c2ccccc2n1	7	PRKD2	PRKD2	=	15
GW857175	Cc1cccc(n1)-c1nc(Nc2ccc3[nH]ncc3c2)c2ccccc2n1	7	PRKD3	PRKD3	=	11
GW857175	Cc1cccc(n1)-c1nc(Nc2ccc3[nH]ncc3c2)c2ccccc2n1	7	TGFBR1	TGFBR1	=	84
GW869979	COc1cc2ncn(-c3cc(OCc4ccc(cc4)S(C)(=O)=O)c(s3)C(N)=O)c2cc1OC	3	MEK5	MAP2K5	=	470
GW869979	COc1cc2ncn(-c3cc(OCc4ccc(cc4)S(C)(=O)=O)c(s3)C(N)=O)c2cc1OC	3	PLK1	PLK1	=	14
GW869979	COc1cc2ncn(-c3cc(OCc4ccc(cc4)S(C)(=O)=O)c(s3)C(N)=O)c2cc1OC	3	PLK2	PLK2	=	5.9
GW869979	COc1cc2ncn(-c3cc(OCc4ccc(cc4)S(C)(=O)=O)c(s3)C(N)=O)c2cc1OC	3	PLK3	PLK3	=	100
PFE-PKIS 1	O=C1C(Br)=C(OCC2=C(F)C=C(F)C=C2)C=C(C)N1C3=CC(C(NCC(N)=O)=O)=CC=C3C	2	p38-alpha	MAPK14	=	0.23
PFE-PKIS 1	O=C1C(Br)=C(OCC2=C(F)C=C(F)C=C2)C=C(C)N1C3=CC(C(NCC(N)=O)=O)=CC=C3C	2	p38-beta	MAPK11	=	10

PFE-PKIS 12	O=C(NCCCN1CCOC1=O)C2=CN=C(NCC3=C(Cl)C=CC(Cl)=C3)N=C2NC4CCCC4	2	CASK	CASK	=	4.6
PFE-PKIS 2	ClC(C=C1)=CC=C1C2=C(C3=CC=NN3)SC(N4CCOCC4)=C2C#N	6	PIK3CA	PIK3CA	=	32
PFE-PKIS 2	ClC(C=C1)=CC=C1C2=C(C3=CC=NN3)SC(N4CCOCC4)=C2C#N	6	PIK3CB	PIK3CB	=	750
PFE-PKIS 2	ClC(C=C1)=CC=C1C2=C(C3=CC=NN3)SC(N4CCOCC4)=C2C#N	6	VPS34	PIK3C3	=	58
PFE-PKIS 21	FC1=C(F)C=CC(C(NOC[C@H](O)CO)=O)=C1NC2=CC=C(I)C=C2F	2	MEK1	MAP2K1	=	47
PFE-PKIS 21	FC1=C(F)C=CC(C(NOC[C@H](O)CO)=O)=C1NC2=CC=C(I)C=C2F	2	MEK2	MAP2K2	=	110
PFE-PKIS 25	CN([C@H]1CN(C(CC#N)=O)CC[C@H]1C)C2=C3C(NC=C3)=NC=N2	10	DCAMKL3	DCLK3	=	26
PFE-PKIS 25	CN([C@H]1CN(C(CC#N)=O)CC[C@H]1C)C2=C3C(NC=C3)=NC=N2	10	JAK1(JH1do	JAK1	=	3.2
PFE-PKIS 25	CN([C@H]1CN(C(CC#N)=O)CC[C@H]1C)C2=C3C(NC=C3)=NC=N2	10	JAK2(JH1do	JAK2	=	0.22
PFE-PKIS 25	CN([C@H]1CN(C(CC#N)=O)CC[C@H]1C)C2=C3C(NC=C3)=NC=N2	10	JAK3(JH1do	JAK3	=	0.13
PFE-PKIS 25	CN([C@H]1CN(C(CC#N)=O)CC[C@H]1C)C2=C3C(NC=C3)=NC=N2	10	MST3	STK24	>	10000
PFE-PKIS 25	CN([C@H]1CN(C(CC#N)=O)CC[C@H]1C)C2=C3C(NC=C3)=NC=N2	10	RSK3(Kin.Dc	RPS6KA2	=	1300
PFE-PKIS 25	CN([C@H]1CN(C(CC#N)=O)CC[C@H]1C)C2=C3C(NC=C3)=NC=N2	10	RSK4(Kin.Dc	RPS6KA6	=	1400
PFE-PKIS 25	CN([C@H]1CN(C(CC#N)=O)CC[C@H]1C)C2=C3C(NC=C3)=NC=N2	10	TYK2(JH1do	TYK2	=	6.9
PFE-PKIS 27	FC1=C(F)C=CC(C(NOCC2CC2)=O)=C1NC3=CC=C(I)C=C3Cl	3	ICK	ICK	>	10000
PFE-PKIS 27	FC1=C(F)C=CC(C(NOCC2CC2)=O)=C1NC3=CC=C(I)C=C3Cl	3	PIK4CB	PI4KB	>	10000
PFE-PKIS 27	FC1=C(F)C=CC(C(NOCC2CC2)=O)=C1NC3=CC=C(I)C=C3Cl	3	RIPK4	RIPK4	>	10000
PFE-PKIS 28	O=C(C1=CN=C(N=C1NC2CCNCC2)NCC3=C(C=CC(Cl)=C3)Cl)NCCCN4C(CCC4)=O	3	CSF1R	CSF1R	>	10000
PFE-PKIS 28	O=C(C1=CN=C(N=C1NC2CCNCC2)NCC3=C(C=CC(Cl)=C3)Cl)NCCCN4C(CCC4)=O	3	CSNK1E	CSNK1E	=	230
PFE-PKIS 28	O=C(C1=CN=C(N=C1NC2CCNCC2)NCC3=C(C=CC(Cl)=C3)Cl)NCCCN4C(CCC4)=O	3	MAK	MAK	>	10000
PFE-PKIS 28	O=C(C1=CN=C(N=C1NC2CCNCC2)NCC3=C(C=CC(Cl)=C3)Cl)NCCCN4C(CCC4)=O	3	RSK1(Kin.Dc	RPS6KA1	>	10000
PFE-PKIS 29	COC(N=C1)=CC=C1C2=CC3=C(C)N=C(N)N=C3N([C@H]4CC[C@H](OCCO)CC4)C2=O	17	MTOR	MTOR	=	3.3
PFE-PKIS 29	COC(N=C1)=CC=C1C2=CC3=C(C)N=C(N)N=C3N([C@H]4CC[C@H](OCCO)CC4)C2=O	17	PIK3C2B	PIK3C2B	=	1.3
PFE-PKIS 29	COC(N=C1)=CC=C1C2=CC3=C(C)N=C(N)N=C3N([C@H]4CC[C@H](OCCO)CC4)C2=O	17	PIK3C2G	PIK3C2G	=	0.92
PFE-PKIS 29	COC(N=C1)=CC=C1C2=CC3=C(C)N=C(N)N=C3N([C@H]4CC[C@H](OCCO)CC4)C2=O	17	PIK3CA	PIK3CA	=	0.45
PFE-PKIS 29	COC(N=C1)=CC=C1C2=CC3=C(C)N=C(N)N=C3N([C@H]4CC[C@H](OCCO)CC4)C2=O	17	PIK3CB	PIK3CB	=	1.7
PFE-PKIS 29	COC(N=C1)=CC=C1C2=CC3=C(C)N=C(N)N=C3N([C@H]4CC[C@H](OCCO)CC4)C2=O	17	PIK3CD	PIK3CD	=	1.6
PFE-PKIS 29	COC(N=C1)=CC=C1C2=CC3=C(C)N=C(N)N=C3N([C@H]4CC[C@H](OCCO)CC4)C2=O	17	PIK3CG	PIK3CG	=	0.27
PFE-PKIS 29	COC(N=C1)=CC=C1C2=CC3=C(C)N=C(N)N=C3N([C@H]4CC[C@H](OCCO)CC4)C2=O	17	PIK4CB	PI4KB	=	46

PFE-PKIS 3	COC1=CC(C2=CC3=CN=C(N)N=C3N=C2NC(NC(C)(C)C)=O)=CC(OC)=C1	7	FGFR1	FGFR1	=	66
PFE-PKIS 3	COC1=CC(C2=CC3=CN=C(N)N=C3N=C2NC(NC(C)(C)C)=O)=CC(OC)=C1	7	FGFR2	FGFR2	=	310
PFE-PKIS 3	COC1=CC(C2=CC3=CN=C(N)N=C3N=C2NC(NC(C)(C)C)=O)=CC(OC)=C1	7	FGFR3	FGFR3	=	110
PFE-PKIS 3	COC1=CC(C2=CC3=CN=C(N)N=C3N=C2NC(NC(C)(C)C)=O)=CC(OC)=C1	7	TNK2	TNK2	>	10000
PFE-PKIS 3	COC1=CC(C2=CC3=CN=C(N)N=C3N=C2NC(NC(C)(C)C)=O)=CC(OC)=C1	7	TYRO3	TYRO3	>	10000
PFE-PKIS 3	COC1=CC(C2=CC3=CN=C(N)N=C3N=C2NC(NC(C)(C)C)=O)=CC(OC)=C1	7	YES	YES1	>	10000
PFE-PKIS 32	CC(C1=CN=C(NC2=NC=C(N3CCNCC3)C=C2)N=C1N4C5CCCC5)=C(C(C)=O)C4=O	8	CDK4-cyclin	CDK4	=	1.3
PFE-PKIS 32	CC(C1=CN=C(NC2=NC=C(N3CCNCC3)C=C2)N=C1N4C5CCCC5)=C(C(C)=O)C4=O	8	CDK4-cyclin	CDK4	=	0.62
PFE-PKIS 32	CC(C1=CN=C(NC2=NC=C(N3CCNCC3)C=C2)N=C1N4C5CCCC5)=C(C(C)=O)C4=O	8	CLK3	CLK3	>	10000
PFE-PKIS 32	CC(C1=CN=C(NC2=NC=C(N3CCNCC3)C=C2)N=C1N4C5CCCC5)=C(C(C)=O)C4=O	8	CSNK1E	CSNK1E	>	10000
PFE-PKIS 32	CC(C1=CN=C(NC2=NC=C(N3CCNCC3)C=C2)N=C1N4C5CCCC5)=C(C(C)=O)C4=O	8	DMPK2	CDC42BPG	>	10000
PFE-PKIS 32	CC(C1=CN=C(NC2=NC=C(N3CCNCC3)C=C2)N=C1N4C5CCCC5)=C(C(C)=O)C4=O	8	ERK3	MAPK6	>	10000
PFE-PKIS 32	CC(C1=CN=C(NC2=NC=C(N3CCNCC3)C=C2)N=C1N4C5CCCC5)=C(C(C)=O)C4=O	8	LIMK2	LIMK2	>	10000
PFE-PKIS 32	CC(C1=CN=C(NC2=NC=C(N3CCNCC3)C=C2)N=C1N4C5CCCC5)=C(C(C)=O)C4=O	8	LZK	MAP3K13	>	10000
PFE-PKIS 32	CC(C1=CN=C(NC2=NC=C(N3CCNCC3)C=C2)N=C1N4C5CCCC5)=C(C(C)=O)C4=O	8	TTK	TTK	=	9.2
PFE-PKIS 34	CN([C@H]1CN(C(CO)=O)CC[C@H]1C)C2=C3C(NC=C3)=NC=N2	9	DCAMKL3	DCLK3	=	240
PFE-PKIS 34	CN([C@H]1CN(C(CO)=O)CC[C@H]1C)C2=C3C(NC=C3)=NC=N2	9	JAK1(JH1do	JAK1	=	120
PFE-PKIS 34	CN([C@H]1CN(C(CO)=O)CC[C@H]1C)C2=C3C(NC=C3)=NC=N2	9	JAK2(JH1do	JAK2	=	11
PFE-PKIS 34	CN([C@H]1CN(C(CO)=O)CC[C@H]1C)C2=C3C(NC=C3)=NC=N2	9	JAK3(JH1do	JAK3	=	3.6
PFE-PKIS 34	CN([C@H]1CN(C(CO)=O)CC[C@H]1C)C2=C3C(NC=C3)=NC=N2	9	MRCKB	CDC42BPB	>	10000
PFE-PKIS 34	CN([C@H]1CN(C(CO)=O)CC[C@H]1C)C2=C3C(NC=C3)=NC=N2	9	NLK	NLK	>	10000
PFE-PKIS 34	CN([C@H]1CN(C(CO)=O)CC[C@H]1C)C2=C3C(NC=C3)=NC=N2	9	PKAC-beta	PRKACB	>	10000
PFE-PKIS 34	CN([C@H]1CN(C(CO)=O)CC[C@H]1C)C2=C3C(NC=C3)=NC=N2	9	PKMYT1	PKMYT1	>	10000
PFE-PKIS 34	CN([C@H]1CN(C(CO)=O)CC[C@H]1C)C2=C3C(NC=C3)=NC=N2	9	PRKG1	PRKG1	>	10000
PFE-PKIS 34	CN([C@H]1CN(C(CO)=O)CC[C@H]1C)C2=C3C(NC=C3)=NC=N2	9	RPS6KA4(Ki	RPS6KA4	>	10000
PFE-PKIS 34	CN([C@H]1CN(C(CO)=O)CC[C@H]1C)C2=C3C(NC=C3)=NC=N2	9	TYK2(JH1do	TYK2	=	110
PFE-PKIS 35	N#CC1=C(N2CCOCC2)SC(C3=NC=NN3)=C1C4=CC=C(S(=O)(C)=O)C=C4	16	BRSK1	BRSK1	>	10000

PFE-PKIS 35	N#CC1=C(N2CCOCC2)SC(C3=NC=NN3)=C1C4=CC=C(S(=O)(C)=O)C=C4	16	IRAK3	IRAK3	>	10000
PFE-PKIS 35	N#CC1=C(N2CCOCC2)SC(C3=NC=NN3)=C1C4=CC=C(S(=O)(C)=O)C=C4	16	MST3	STK24	>	10000
PFE-PKIS 35	N#CC1=C(N2CCOCC2)SC(C3=NC=NN3)=C1C4=CC=C(S(=O)(C)=O)C=C4	16	PIK3CA	PIK3CA	=	21
PFE-PKIS 35	N#CC1=C(N2CCOCC2)SC(C3=NC=NN3)=C1C4=CC=C(S(=O)(C)=O)C=C4	16	PIK3CB	PIK3CB	=	77
PFE-PKIS 35	N#CC1=C(N2CCOCC2)SC(C3=NC=NN3)=C1C4=CC=C(S(=O)(C)=O)C=C4	16	RSK3(Kin.Dc	RPS6KA2	>	10000
PFE-PKIS 35	N#CC1=C(N2CCOCC2)SC(C3=NC=NN3)=C1C4=CC=C(S(=O)(C)=O)C=C4	16	RSK4(Kin.Dc	RPS6KA6	>	10000
PFE-PKIS 35	N#CC1=C(N2CCOCC2)SC(C3=NC=NN3)=C1C4=CC=C(S(=O)(C)=O)C=C4	16	VPS34	PIK3C3	=	110
PFE-PKIS 39	CN(N=C1)C=C1C(C=C2)=NN3C2=NN=C3[C@@H](C)C4=CC=C(N=CC=C5)C5=C4	13	BRSK2	BRSK2	>	10000
PFE-PKIS 39	CN(N=C1)C=C1C(C=C2)=NN3C2=NN=C3[C@@H](C)C4=CC=C(N=CC=C5)C5=C4	13	CHEK1	CHEK1	>	10000
PFE-PKIS 39	CN(N=C1)C=C1C(C=C2)=NN3C2=NN=C3[C@@H](C)C4=CC=C(N=CC=C5)C5=C4	13	DAPK2	DAPK2	>	10000
PFE-PKIS 39	CN(N=C1)C=C1C(C=C2)=NN3C2=NN=C3[C@@H](C)C4=CC=C(N=CC=C5)C5=C4	13	DAPK3	DAPK3	>	10000
PFE-PKIS 39	CN(N=C1)C=C1C(C=C2)=NN3C2=NN=C3[C@@H](C)C4=CC=C(N=CC=C5)C5=C4	13	DDR1	DDR1	>	10000
PFE-PKIS 39	CN(N=C1)C=C1C(C=C2)=NN3C2=NN=C3[C@@H](C)C4=CC=C(N=CC=C5)C5=C4	13	EPHA1	EPHA1	>	10000
PFE-PKIS 39	CN(N=C1)C=C1C(C=C2)=NN3C2=NN=C3[C@@H](C)C4=CC=C(N=CC=C5)C5=C4	13	MAP3K4	MAP3K4	>	10000
PFE-PKIS 39	CN(N=C1)C=C1C(C=C2)=NN3C2=NN=C3[C@@H](C)C4=CC=C(N=CC=C5)C5=C4	13	MET	MET	=	0.28
PFE-PKIS 39	CN(N=C1)C=C1C(C=C2)=NN3C2=NN=C3[C@@H](C)C4=CC=C(N=CC=C5)C5=C4	13	MYO3A	MYO3A	>	10000
PFE-PKIS 39	CN(N=C1)C=C1C(C=C2)=NN3C2=NN=C3[C@@H](C)C4=CC=C(N=CC=C5)C5=C4	13	NEK4	NEK4	>	10000
PFE-PKIS 39	CN(N=C1)C=C1C(C=C2)=NN3C2=NN=C3[C@@H](C)C4=CC=C(N=CC=C5)C5=C4	13	PHKG2	PHKG2	>	10000
PFE-PKIS 39	CN(N=C1)C=C1C(C=C2)=NN3C2=NN=C3[C@@H](C)C4=CC=C(N=CC=C5)C5=C4	13	PRKD3	PRKD3	>	10000
PFE-PKIS 39	CN(N=C1)C=C1C(C=C2)=NN3C2=NN=C3[C@@H](C)C4=CC=C(N=CC=C5)C5=C4	13	TRPM6	TRPM6	>	10000
PFE-PKIS 40	O=C1N([C@@H]2CC[C@@H](O)CC2)C3=NC(N)=NC(C)=C3C=C1C4=CC=C(OC)N=C4	19	MTOR	MTOR	=	4.8
PFE-PKIS 40	O=C1N([C@@H]2CC[C@@H](O)CC2)C3=NC(N)=NC(C)=C3C=C1C4=CC=C(OC)N=C4	19	MYLK	MYLK	>	10000
PFE-PKIS 40	O=C1N([C@@H]2CC[C@@H](O)CC2)C3=NC(N)=NC(C)=C3C=C1C4=CC=C(OC)N=C4	19	p38-gamma	MAPK12	>	10000
PFE-PKIS 40	O=C1N([C@@H]2CC[C@@H](O)CC2)C3=NC(N)=NC(C)=C3C=C1C4=CC=C(OC)N=C4	19	PIK3C2B	PIK3C2B	=	2.7
PFE-PKIS 40	O=C1N([C@@H]2CC[C@@H](O)CC2)C3=NC(N)=NC(C)=C3C=C1C4=CC=C(OC)N=C4	19	PIK3C2G	PIK3C2G	=	0.46
PFE-PKIS 40	O=C1N([C@@H]2CC[C@@H](O)CC2)C3=NC(N)=NC(C)=C3C=C1C4=CC=C(OC)N=C4	19	PIK3CA	PIK3CA	=	0.34
PFE-PKIS 40	O=C1N([C@@H]2CC[C@@H](O)CC2)C3=NC(N)=NC(C)=C3C=C1C4=CC=C(OC)N=C4	19	PIK3CB	PIK3CB	=	1.7

PFE-PKIS 40	O=C1N([C@@H]2CC[C@@H](O)CC2)C3=NC(N)=NC(C)=C3C=C1C4=CC=C(OC)N=C4	19	PIK3CD	PIK3CD	=	0.49
PFE-PKIS 40	O=C1N([C@@H]2CC[C@@H](O)CC2)C3=NC(N)=NC(C)=C3C=C1C4=CC=C(OC)N=C4	19	PIK3CG	PIK3CG	=	0.32
PFE-PKIS 40	O=C1N([C@@H]2CC[C@@H](O)CC2)C3=NC(N)=NC(C)=C3C=C1C4=CC=C(OC)N=C4	19	PIK4CB	PI4KB	=	25
PFE-PKIS 40	O=C1N([C@@H]2CC[C@@H](O)CC2)C3=NC(N)=NC(C)=C3C=C1C4=CC=C(OC)N=C4	19	TBK1	TBK1	>	10000
PFE-PKIS 41	CC1=CC(OCC2=C(F)C=C(F)C=C2)=C(Br)C(N1C3=C(C)C=CC(C(NC)=O)=C3)=O	8	CDKL1	CDKL1	>	10000
PFE-PKIS 41	CC1=CC(OCC2=C(F)C=C(F)C=C2)=C(Br)C(N1C3=C(C)C=CC(C(NC)=O)=C3)=O	8	JNK2	MAPK9	>	10000
PFE-PKIS 41	CC1=CC(OCC2=C(F)C=C(F)C=C2)=C(Br)C(N1C3=C(C)C=CC(C(NC)=O)=C3)=O	8	JNK3	MAPK10	>	10000
PFE-PKIS 41	CC1=CC(OCC2=C(F)C=C(F)C=C2)=C(Br)C(N1C3=C(C)C=CC(C(NC)=O)=C3)=O	8	MKNK2	MKNK2	>	10000
PFE-PKIS 41	CC1=CC(OCC2=C(F)C=C(F)C=C2)=C(Br)C(N1C3=C(C)C=CC(C(NC)=O)=C3)=O	8	p38-alpha	MAPK14	=	4.1
PFE-PKIS 41	CC1=CC(OCC2=C(F)C=C(F)C=C2)=C(Br)C(N1C3=C(C)C=CC(C(NC)=O)=C3)=O	8	p38-beta	MAPK11	=	120
PFE-PKIS 41	CC1=CC(OCC2=C(F)C=C(F)C=C2)=C(Br)C(N1C3=C(C)C=CC(C(NC)=O)=C3)=O	8	PRKCI	PRKCI	>	10000
PFE-PKIS 41	CC1=CC(OCC2=C(F)C=C(F)C=C2)=C(Br)C(N1C3=C(C)C=CC(C(NC)=O)=C3)=O	8	PRKG2	PRKG2	>	10000
PFE-PKIS 42	C[C@H](CN1)N=C(N(C)C)C2=C1C3=C(C=CC(C4=CN=C(C)C=C4)=N5)C5=CC=C3S2	1	MYLK4	MYLK4	>	10000
PFE-PKIS 43	O=C(CSC1=C2C(C=C(S2)C3=CC=C(C=C3)SC)=NC=N1)O	2	DRAK2	STK17B	=	3.8
PFE-PKIS 43	O=C(CSC1=C2C(C=C(S2)C3=CC=C(C=C3)SC)=NC=N1)O	2	SRPK2	SRPK2	>	10000
PFE-PKIS 44	CC(C1=CN=C(NC2=NN(C)C=C2)N=C1N3C4CCCC4)=C(C(C)=O)C3=O	6	CDC2L2	CDC2L2	>	10000
PFE-PKIS 44	CC(C1=CN=C(NC2=NN(C)C=C2)N=C1N3C4CCCC4)=C(C(C)=O)C3=O	6	CDK4-cyclin	CDK4	=	23
PFE-PKIS 44	CC(C1=CN=C(NC2=NN(C)C=C2)N=C1N3C4CCCC4)=C(C(C)=O)C3=O	6	CDK4-cyclin	CDK4	=	9.9
PFE-PKIS 44	CC(C1=CN=C(NC2=NN(C)C=C2)N=C1N3C4CCCC4)=C(C(C)=O)C3=O	6	DCAMKL2	DCLK2	>	10000
PFE-PKIS 44	CC(C1=CN=C(NC2=NN(C)C=C2)N=C1N3C4CCCC4)=C(C(C)=O)C3=O	6	PCTK3	CDK18	>	10000
PFE-PKIS 44	CC(C1=CN=C(NC2=NN(C)C=C2)N=C1N3C4CCCC4)=C(C(C)=O)C3=O	6	PIP5K2C	PIP4K2C	=	110
PFE-PKIS 46	O=C(C1)NC(C1=C2)=CC=C2NC3=NC(NCC4=CC(S(=O)(C(F)(F)F)=O)=CC=C4)=NC=C3C(F)(F)F	2	PAK4	PAK4	>	10000
PFE-PKIS 46	O=C(C1)NC(C1=C2)=CC=C2NC3=NC(NCC4=CC(S(=O)(C(F)(F)F)=O)=CC=C4)=NC=C3C(F)(F)F	2	PIM1	PIM1	>	10000
PFE-PKIS 47	CN([C@H]1CN(C(CC#N)=O)CC[C@H]1C)C2=C3C(NC=C3)=NC(C)=N2	1	JAK3(JH1do	JAK3	=	140
PFE-PKIS 9	O=C(O)COC1=C2C(C=C(C3=CC=C(NN=C4)C4=C3)S2)=NC=N1	5	BRK	PTK6	>	10000
PFE-PKIS 9	O=C(O)COC1=C2C(C=C(C3=CC=C(NN=C4)C4=C3)S2)=NC=N1	5	DRAK1	STK17A	=	250
PFE-PKIS 9	O=C(O)COC1=C2C(C=C(C3=CC=C(NN=C4)C4=C3)S2)=NC=N1	5	DRAK2	STK17B	=	30
PFE-PKIS 9	O=C(O)COC1=C2C(C=C(C3=CC=C(NN=C4)C4=C3)S2)=NC=N1	5	FRK	FRK	>	10000

PFE-PKIS 9	O=C(O)COC1=C2C(C=C(C3=CC=C(NN=C4)C4=C3)S2)=NC=N1	5	FYN	FYN	>	10000
PFE-PKIS 9	O=C(O)COC1=C2C(C=C(C3=CC=C(NN=C4)C4=C3)S2)=NC=N1	5	LTK	LTK	>	10000
PFE-PKIS 9	O=C(O)COC1=C2C(C=C(C3=CC=C(NN=C4)C4=C3)S2)=NC=N1	5	NEK3	NEK3	=	210
PFE-PKIS 9	O=C(O)COC1=C2C(C=C(C3=CC=C(NN=C4)C4=C3)S2)=NC=N1	5	YSK4	MAP3K19	=	20
SB-210486	Fc1ccc(cc1)-c1ncn(CCCNCc2ccccc2)c1-c1ccncc1	3	CSNK1A1	CSNK1A1	=	210
SB-210486	Fc1ccc(cc1)-c1ncn(CCCNCc2ccccc2)c1-c1ccncc1	3	CSNK1D	CSNK1D	=	47
SB-210486	Fc1ccc(cc1)-c1ncn(CCCNCc2ccccc2)c1-c1ccncc1	3	CSNK1E	CSNK1E	=	130
SKF-97510	Cc1ccccc1-n1cnc2c(N)ncnc12	8	ABL1-nonpł	ABL1	>	10000
SKF-97510	Cc1ccccc1-n1cnc2c(N)ncnc12	8	DAPK1	DAPK1	>	10000
SKF-97510	Cc1ccccc1-n1cnc2c(N)ncnc12	8	LCK	LCK	>	10000
SKF-97510	Cc1ccccc1-n1cnc2c(N)ncnc12	8	RSK1(Kin.Dc	RPS6KA1	>	10000
SKF-97510	Cc1ccccc1-n1cnc2c(N)ncnc12	8	SLK	SLK	>	10000
SKF-97510	Cc1ccccc1-n1cnc2c(N)ncnc12	8	SYK	SYK	>	10000
SKF-97510	Cc1ccccc1-n1cnc2c(N)ncnc12	8	TLK1	TLK1	>	10000
SKF-97560	Nc1ncnc2n(cnc12)-c1cccc(F)c1	6	FGFR4	FGFR4	>	10000
SKF-97560	Nc1ncnc2n(cnc12)-c1cccc(F)c1	6	RIPK2	RIPK2	>	10000
SKF-97560	Nc1ncnc2n(cnc12)-c1cccc(F)c1	6	TNNI3K	TNNI3K	>	10000
TPKI-100	COC(=O)C1=C(C2=CC=CC=C2)C2=CC(Br)=CC=C2C(=O)N1CC1=CC=C(C=C1)S(C)(=O)=O	3	JNK1	MAPK8	=	480
TPKI-100	COC(=O)C1=C(C2=CC=CC=C2)C2=CC(Br)=CC=C2C(=O)N1CC1=CC=C(C=C1)S(C)(=O)=O	3	JNK2	MAPK9	=	350
TPKI-100	COC(=O)C1=C(C2=CC=CC=C2)C2=CC(Br)=CC=C2C(=O)N1CC1=CC=C(C=C1)S(C)(=O)=O	3	JNK3	MAPK10	=	73
TPKI-101	CCC(=O)C1=C(C2=CC=CC=C2)C2=CC(Cl)=CC=C2C(=O)N1CC1=CC=C(C=C1)C(O)=O	3	JNK1	MAPK8	=	66
TPKI-101	CCC(=O)C1=C(C2=CC=CC=C2)C2=CC(Cl)=CC=C2C(=O)N1CC1=CC=C(C=C1)C(O)=O	3	JNK2	MAPK9	=	77
TPKI-101	CCC(=O)C1=C(C2=CC=CC=C2)C2=CC(Cl)=CC=C2C(=O)N1CC1=CC=C(C=C1)C(O)=O	3	JNK3	MAPK10	=	12
TPKI-102	CCC(=O)C1=C(C2=CC=CC=C2)C2=CC(Cl)=CC=C2C(=O)N1CC1=NN(C)C(=C1)C(=O)NC1CC1	3	JNK1	MAPK8	=	20
TPKI-102	CCC(=O)C1=C(C2=CC=CC=C2)C2=CC(Cl)=CC=C2C(=O)N1CC1=NN(C)C(=C1)C(=O)NC1CC1	3	JNK2	MAPK9	=	10
TPKI-102	CCC(=O)C1=C(C2=CC=CC=C2)C2=CC(Cl)=CC=C2C(=O)N1CC1=NN(C)C(=C1)C(=O)NC1CC1	3	JNK3	MAPK10	=	3.8
TPKI-103	CCC(=O)C1=C(C2=CC=CC=C2)C2=CC(Cl)=CC=C2C(=O)N1CC1=NN(CC(F)F)C(=C1)S(C)(=O)=O	3	JNK1	MAPK8	=	37
TPKI-103	CCC(=O)C1=C(C2=CC=CC=C2)C2=CC(Cl)=CC=C2C(=O)N1CC1=NN(CC(F)F)C(=C1)S(C)(=O)=O	3	JNK2	MAPK9	=	33
TPKI-103	CCC(=O)C1=C(C2=CC=CC=C2)C2=CC(Cl)=CC=C2C(=O)N1CC1=NN(CC(F)F)C(=C1)S(C)(=O)=O	3	JNK3	MAPK10	=	9.2
TPKI-104	CCC(=O)C1=C(C2=CC=CC=C2)C2=CC(Cl)=CC=C2C(=O)N1CC1=NN(CCC(C)(C)O)C(=C1)S(C)(=O)=O	3	JNK1	MAPK8	=	19
TPKI-104	CCC(=O)C1=C(C2=CC=CC=C2)C2=CC(Cl)=CC=C2C(=O)N1CC1=NN(CCC(C)(C)O)C(=C1)S(C)(=O)=O	3	JNK2	MAPK9	=	15
TPKI-104	CCC(=O)C1=C(C2=CC=CC=C2)C2=CC(Cl)=CC=C2C(=O)N1CC1=NN(CCC(C)(C)O)C(=C1)S(C)(=O)=O	3	JNK3	MAPK10	=	4.6
TPKI-105	CCC(=O)C1=C(C2=CC=CC=C2)C2=CC(Cl)=CC=C2C(=O)N1CC1=NN(CC(N)=O)C(=C1)S(C)(=O)=O	6	JNK1	MAPK8	=	160
TPKI-105	CCC(=O)C1=C(C2=CC=CC=C2)C2=CC(Cl)=CC=C2C(=O)N1CC1=NN(CC(N)=O)C(=C1)S(C)(=O)=O	6	JNK2	MAPK9	=	120

TPKI-105	CCC(=O)C1=C(C2=CC=CC=C2)C2=CC(Cl)=CC=C2C(=O)N1CC1=NN(CC(N)=O)C(=C1)S(C)(=O)=O	6	JNK3	MAPK10	=	60
TPKI-105	CCC(=O)C1=C(C2=CC=CC=C2)C2=CC(Cl)=CC=C2C(=O)N1CC1=NN(CC(N)=O)C(=C1)S(C)(=O)=O	6	PRKCH	PRKCH	>	10000
TPKI-106	CCC1=NC(=C(S1)C1=CC=NC=C1)C1=CC(C)=CC=C1	4	FAK	PTK2	>	10000
TPKI-106	CCC1=NC(=C(S1)C1=CC=NC=C1)C1=CC(C)=CC=C1	4	JNK3	MAPK10	=	81
TPKI-106	CCC1=NC(=C(S1)C1=CC=NC=C1)C1=CC(C)=CC=C1	4	NLK	NLK	=	180
TPKI-106	CCC1=NC(=C(S1)C1=CC=NC=C1)C1=CC(C)=CC=C1	4	RSK1(Kin.Dc	RPS6KA1	=	250
TPKI-106	CCC1=NC(=C(S1)C1=CC=NC=C1)C1=CC(C)=CC=C1	4	RSK4(Kin.Dc	RPS6KA6	=	120
TPKI-107	CCC1=NC(=C(S1)C1=CC(NC(=O)C2=CC=CC=C2)=NC=C1)C1=CC(C)=CC=C1	6	CSNK1A1	CSNK1A1	=	1400
TPKI-107	CCC1=NC(=C(S1)C1=CC(NC(=O)C2=CC=CC=C2)=NC=C1)C1=CC(C)=CC=C1	6	CSNK1D	CSNK1D	=	690
TPKI-107	CCC1=NC(=C(S1)C1=CC(NC(=O)C2=CC=CC=C2)=NC=C1)C1=CC(C)=CC=C1	6	CSNK1E	CSNK1E	=	880
TPKI-107	CCC1=NC(=C(S1)C1=CC(NC(=O)C2=CC=CC=C2)=NC=C1)C1=CC(C)=CC=C1	6	EPHB6	EPHB6	=	7600
TPKI-107	CCC1=NC(=C(S1)C1=CC(NC(=O)C2=CC=CC=C2)=NC=C1)C1=CC(C)=CC=C1	6	IKK-epsilon	IKBKE	>	10000
TPKI-107	CCC1=NC(=C(S1)C1=CC(NC(=O)C2=CC=CC=C2)=NC=C1)C1=CC(C)=CC=C1	6	JNK3	MAPK10	=	190
TPKI-107	CCC1=NC(=C(S1)C1=CC(NC(=O)C2=CC=CC=C2)=NC=C1)C1=CC(C)=CC=C1	6	LATS2	LATS2	>	10000
TPKI-107	CCC1=NC(=C(S1)C1=CC(NC(=O)C2=CC=CC=C2)=NC=C1)C1=CC(C)=CC=C1	6	p38-alpha	MAPK14	=	100
TPKI-107	CCC1=NC(=C(S1)C1=CC(NC(=O)C2=CC=CC=C2)=NC=C1)C1=CC(C)=CC=C1	6	PIP5K2C	PIP4K2C	=	4100
TPKI-108	CCC1=NC(=C(S1)C1=CC(NCC2=CC=CC=C2)=NC=C1)C1=CC(C)=CC=C1	1	p38-alpha	MAPK14	=	150
TPKI-109	CC1=CC=CC(=C1)C1=C(SC(=N1)C1=CC=C(C=C1)S(C)(=O)=O)C1=CC=NC=C1	1	p38-alpha	MAPK14	=	540
TPKI-110	CC1=CC=CC(=C1)C1=C(SC(=N1)C1=CC=C(C=C1)S(C)(=O)=O)C1=CC(NCCC2=CC=CC=C2)=NC=C1	9	ABL1-nonpl	ABL1	=	330
TPKI-110	CC1=CC=CC(=C1)C1=C(SC(=N1)C1=CC=C(C=C1)S(C)(=O)=O)C1=CC(NCCC2=CC=CC=C2)=NC=C1	9	BRAF	BRAF	=	81
TPKI-110	CC1=CC=CC(=C1)C1=C(SC(=N1)C1=CC=C(C=C1)S(C)(=O)=O)C1=CC(NCCC2=CC=CC=C2)=NC=C1	9	DDR1	DDR1	=	230
TPKI-110	CC1=CC=CC(=C1)C1=C(SC(=N1)C1=CC=C(C=C1)S(C)(=O)=O)C1=CC(NCCC2=CC=CC=C2)=NC=C1	9	KIT	KIT	=	120
TPKI-110	CC1=CC=CC(=C1)C1=C(SC(=N1)C1=CC=C(C=C1)S(C)(=O)=O)C1=CC(NCCC2=CC=CC=C2)=NC=C1	9	p38-alpha	MAPK14	=	1900
TPKI-110	CC1=CC=CC(=C1)C1=C(SC(=N1)C1=CC=C(C=C1)S(C)(=O)=O)C1=CC(NCCC2=CC=CC=C2)=NC=C1	9	PDGFRA	PDGFRA	=	550
TPKI-110	CC1=CC=CC(=C1)C1=C(SC(=N1)C1=CC=C(C=C1)S(C)(=O)=O)C1=CC(NCCC2=CC=CC=C2)=NC=C1	9	PDGFRB	PDGFRB	=	68
TPKI-110	CC1=CC=CC(=C1)C1=C(SC(=N1)C1=CC=C(C=C1)S(C)(=O)=O)C1=CC(NCCC2=CC=CC=C2)=NC=C1	9	RAF1	RAF1	=	150
TPKI-110	CC1=CC=CC(=C1)C1=C(SC(=N1)C1=CC=C(C=C1)S(C)(=O)=O)C1=CC(NCCC2=CC=CC=C2)=NC=C1	9	VPS34	PIK3C3	=	8400
TPKI-111	CC(=O)NC1=NC2=CN=C(NC3=C(C)C=CC(NC(=O)C4=CC=CC(CCC#N)=C4)=C3)N=C2S1	11	ABL1-nonpl	ABL1	>	10000
TPKI-111	CC(=O)NC1=NC2=CN=C(NC3=C(C)C=CC(NC(=O)C4=CC=CC(CCC#N)=C4)=C3)N=C2S1	11	BRAF	BRAF	>	10000
TPKI-111	CC(=O)NC1=NC2=CN=C(NC3=C(C)C=CC(NC(=O)C4=CC=CC(CCC#N)=C4)=C3)N=C2S1	11	CSF1R	CSF1R	>	10000

TPKI-111	CC(=O)NC1=NC2=CN=C(NC3=C(C)C=CC(NC(=O)C4=CC=CC(CCC#N)=C4)=C3)N=C2S1	11	DDR1	DDR1	>	10000
TPKI-111	CC(=O)NC1=NC2=CN=C(NC3=C(C)C=CC(NC(=O)C4=CC=CC(CCC#N)=C4)=C3)N=C2S1	11	DDR2	DDR2	>	10000
TPKI-111	CC(=O)NC1=NC2=CN=C(NC3=C(C)C=CC(NC(=O)C4=CC=CC(CCC#N)=C4)=C3)N=C2S1	11	FRK	FRK	>	10000
TPKI-111	CC(=O)NC1=NC2=CN=C(NC3=C(C)C=CC(NC(=O)C4=CC=CC(CCC#N)=C4)=C3)N=C2S1	11	KIT	KIT	>	10000
TPKI-111	CC(=O)NC1=NC2=CN=C(NC3=C(C)C=CC(NC(=O)C4=CC=CC(CCC#N)=C4)=C3)N=C2S1	11	PDGFRA	PDGFRA	>	10000
TPKI-111	CC(=O)NC1=NC2=CN=C(NC3=C(C)C=CC(NC(=O)C4=CC=CC(CCC#N)=C4)=C3)N=C2S1	11	PDGFRB	PDGFRB	>	10000
TPKI-111	CC(=O)NC1=NC2=CN=C(NC3=C(C)C=CC(NC(=O)C4=CC=CC(CCC#N)=C4)=C3)N=C2S1	11	RAF1	RAF1	>	10000
TPKI-16	Cn1c2ncn(OC[C@H](O)CO)c(=O)c2c(Nc3ccc(I)cc3F)c(F)c1=O	2	HIPK4	HIPK4	>	10000
TPKI-16	Cn1c2ncn(OC[C@H](O)CO)c(=O)c2c(Nc3ccc(I)cc3F)c(F)c1=O	2	MEK1	MAP2K1	=	34
TPKI-16	Cn1c2ncn(OC[C@H](O)CO)c(=O)c2c(Nc3ccc(I)cc3F)c(F)c1=O	2	MEK2	MAP2K2	=	200
TPKI-24	COc1cc(ccc1Nc2ncc3N(C)C(=O)C(F)(F)CN(C4CCCC4)c3n2)C(=O)NC5CCN(C)CC5	7	DAPK3	DAPK3	=	82
TPKI-24	COc1cc(ccc1Nc2ncc3N(C)C(=O)C(F)(F)CN(C4CCCC4)c3n2)C(=O)NC5CCN(C)CC5	7	FAK	PTK2	=	160
TPKI-24	COc1cc(ccc1Nc2ncc3N(C)C(=O)C(F)(F)CN(C4CCCC4)c3n2)C(=O)NC5CCN(C)CC5	7	MINK	MINK1	=	4000
TPKI-24	COc1cc(ccc1Nc2ncc3N(C)C(=O)C(F)(F)CN(C4CCCC4)c3n2)C(=O)NC5CCN(C)CC5	7	MYLK	MYLK	=	79
TPKI-24	COc1cc(ccc1Nc2ncc3N(C)C(=O)C(F)(F)CN(C4CCCC4)c3n2)C(=O)NC5CCN(C)CC5	7	PIK3C2B	PIK3C2B	>	10000
TPKI-24	COc1cc(ccc1Nc2ncc3N(C)C(=O)C(F)(F)CN(C4CCCC4)c3n2)C(=O)NC5CCN(C)CC5	7	PIK3CA	PIK3CA	>	10000
TPKI-24	COc1cc(ccc1Nc2ncc3N(C)C(=O)C(F)(F)CN(C4CCCC4)c3n2)C(=O)NC5CCN(C)CC5	7	PLK1	PLK1	=	0.058
TPKI-24	COc1cc(ccc1Nc2ncc3N(C)C(=O)C(F)(F)CN(C4CCCC4)c3n2)C(=O)NC5CCN(C)CC5	7	PLK2	PLK2	=	2.2
TPKI-24	COc1cc(ccc1Nc2ncc3N(C)C(=O)C(F)(F)CN(C4CCCC4)c3n2)C(=O)NC5CCN(C)CC5	7	PLK3	PLK3	=	1.7
TPKI-25	CC[C@@]1(F)CN(C2CCCC2)c3nc(Nc4ccc(cc4OC)C(=O)NC5CCN(C)CC5)ncc3N(C)C1=O	2	MYLK	MYLK	=	360
TPKI-25	CC[C@@]1(F)CN(C2CCCC2)c3nc(Nc4ccc(cc4OC)C(=O)NC5CCN(C)CC5)ncc3N(C)C1=O	2	PLK1	PLK1	=	0.77
TPKI-25	CC[C@@]1(F)CN(C2CCCC2)c3nc(Nc4ccc(cc4OC)C(=O)NC5CCN(C)CC5)ncc3N(C)C1=O	2	PLK2	PLK2	=	76
TPKI-25	CC[C@@]1(F)CN(C2CCCC2)c3nc(Nc4ccc(cc4OC)C(=O)NC5CCN(C)CC5)ncc3N(C)C1=O	2	PLK3	PLK3	=	180
TPKI-25	CC[C@@]1(F)CN(C2CCCC2)c3nc(Nc4ccc(cc4OC)C(=O)NC5CCN(C)CC5)ncc3N(C)C1=O	2	VPS34	PIK3C3	>	10000
TPKI-26	CC[C@]1(F)CN(C2CCCC2)c3nc(Nc4ccc(cc4OC)C(=O)NC5CCN(C)CC5)ncc3N(C)C1=O	3	PLK1	PLK1	=	0.23
TPKI-26	CC[C@]1(F)CN(C2CCCC2)c3nc(Nc4ccc(cc4OC)C(=O)NC5CCN(C)CC5)ncc3N(C)C1=O	3	PLK2	PLK2	=	5.9
TPKI-26	CC[C@]1(F)CN(C2CCCC2)c3nc(Nc4ccc(cc4OC)C(=O)NC5CCN(C)CC5)ncc3N(C)C1=O	3	PLK3	PLK3	=	4.8

TPKI-27	COc1cc(ccc1Nc2ncc3N(C)C(=O)[C@@H](CC=C)CN(C4CCCC4)c3n2)C(=O)NC5CCN(C)CC5	12	EGFR	EGFR	=	260
TPKI-27	COc1cc(ccc1Nc2ncc3N(C)C(=O)[C@@H](CC=C)CN(C4CCCC4)c3n2)C(=O)NC5CCN(C)CC5	12	EPHA2	EPHA2	>	10000
TPKI-27	COc1cc(ccc1Nc2ncc3N(C)C(=O)[C@@H](CC=C)CN(C4CCCC4)c3n2)C(=O)NC5CCN(C)CC5	12	MEK3	MAP2K3	>	10000
TPKI-27	COc1cc(ccc1Nc2ncc3N(C)C(=O)[C@@H](CC=C)CN(C4CCCC4)c3n2)C(=O)NC5CCN(C)CC5	12	PLK1	PLK1	=	0.33
TPKI-27	COc1cc(ccc1Nc2ncc3N(C)C(=O)[C@@H](CC=C)CN(C4CCCC4)c3n2)C(=O)NC5CCN(C)CC5	12	PLK2	PLK2	=	64
TPKI-27	COc1cc(ccc1Nc2ncc3N(C)C(=O)[C@@H](CC=C)CN(C4CCCC4)c3n2)C(=O)NC5CCN(C)CC5	12	PLK3	PLK3	=	22
TPKI-27	COc1cc(ccc1Nc2ncc3N(C)C(=O)[C@@H](CC=C)CN(C4CCCC4)c3n2)C(=O)NC5CCN(C)CC5	12	VPS34	PIK3C3	>	10000
TPKI-27	COc1cc(ccc1Nc2ncc3N(C)C(=O)[C@@H](CC=C)CN(C4CCCC4)c3n2)C(=O)NC5CCN(C)CC5	12	WEE2	WEE2	=	480
TPKI-28	COc1cc(cc(F)c1Nc2ncc3N(C)C(=O)C(F)(F)CN(C4CCCC4)c3n2)C(=O)NC5CCN(C)CC5	3	PLK1	PLK1	=	0.3
TPKI-28	COc1cc(cc(F)c1Nc2ncc3N(C)C(=O)C(F)(F)CN(C4CCCC4)c3n2)C(=O)NC5CCN(C)CC5	3	PLK2	PLK2	=	79
TPKI-28	COc1cc(cc(F)c1Nc2ncc3N(C)C(=O)C(F)(F)CN(C4CCCC4)c3n2)C(=O)NC5CCN(C)CC5	3	PLK3	PLK3	=	22
TPKI-32	NC(=O)c1nnc2ccccc2c1N	2	ADCK3	CABC1	>	10000
TPKI-32	NC(=O)c1nnc2ccccc2c1N	2	BMX	BMX	>	10000
TPKI-32	NC(=O)c1nnc2ccccc2c1N	2	FGFR3	FGFR3	>	10000
TPKI-32	NC(=O)c1nnc2ccccc2c1N	2	MLK1	MAP3K9	>	10000
TPKI-32	NC(=O)c1nnc2ccccc2c1N	2	NEK10	NEK10	=	430
TPKI-32	NC(=O)c1nnc2ccccc2c1N	2	TIE2	TEK	>	10000
TPKI-32	NC(=O)c1nnc2ccccc2c1N	2	YSK4	MAP3K19	=	300
TPKI-38	O=C(NC1=CC=CC=C1)NC1=CC=C(OC2=NN3C=CN=C3C=C2)C=C1	8	CTK	MATK	>	10000
TPKI-38	O=C(NC1=CC=CC=C1)NC1=CC=C(OC2=NN3C=CN=C3C=C2)C=C1	8	DDR1	DDR1	=	9.3
TPKI-38	O=C(NC1=CC=CC=C1)NC1=CC=C(OC2=NN3C=CN=C3C=C2)C=C1	8	DDR2	DDR2	=	3
TPKI-38	O=C(NC1=CC=CC=C1)NC1=CC=C(OC2=NN3C=CN=C3C=C2)C=C1	8	ERN1	ERN1	>	10000
TPKI-38	O=C(NC1=CC=CC=C1)NC1=CC=C(OC2=NN3C=CN=C3C=C2)C=C1	8	KIT	KIT	=	99
TPKI-38	O=C(NC1=CC=CC=C1)NC1=CC=C(OC2=NN3C=CN=C3C=C2)C=C1	8	PDGFRA	PDGFRA	=	71
TPKI-38	O=C(NC1=CC=CC=C1)NC1=CC=C(OC2=NN3C=CN=C3C=C2)C=C1	8	PDGFRB	PDGFRB	=	62
TPKI-38	O=C(NC1=CC=CC=C1)NC1=CC=C(OC2=NN3C=CN=C3C=C2)C=C1	8	RIPK2	RIPK2	>	10000
TPKI-38	O=C(NC1=CC=CC=C1)NC1=CC=C(OC2=NN3C=CN=C3C=C2)C=C1	8	TNNI3K	TNNI3K	>	10000
TPKI-39	O=C(NC1=CC=CC=C1)NC1=CC=CC(OC2=NN3C=CN=C3C=C2)=C1	10	ABL1-nonpł	ABL1	=	140
TPKI-39	O=C(NC1=CC=CC=C1)NC1=CC=CC(OC2=NN3C=CN=C3C=C2)=C1	10	CIT	CIT	=	1.1
TPKI-39	O=C(NC1=CC=CC=C1)NC1=CC=CC(OC2=NN3C=CN=C3C=C2)=C1	10	CSF1R	CSF1R	=	27
TPKI-39	O=C(NC1=CC=CC=C1)NC1=CC=CC(OC2=NN3C=CN=C3C=C2)=C1	10	DDR1	DDR1	=	24

TPKI-39	O=C(NC1=CC=CC=C1)NC1=CC=CC(OC2=NN3C=CN=C3C=C2)=C1	10	FLT1	FLT1	=	91
TPKI-39	O=C(NC1=CC=CC=C1)NC1=CC=CC(OC2=NN3C=CN=C3C=C2)=C1	10	KIT	KIT	=	0.94
TPKI-39	O=C(NC1=CC=CC=C1)NC1=CC=CC(OC2=NN3C=CN=C3C=C2)=C1	10	PDGFRA	PDGFRA	=	3.6
TPKI-39	O=C(NC1=CC=CC=C1)NC1=CC=CC(OC2=NN3C=CN=C3C=C2)=C1	10	PDGFRB	PDGFRB	=	1
TPKI-39	O=C(NC1=CC=CC=C1)NC1=CC=CC(OC2=NN3C=CN=C3C=C2)=C1	10	TIE2	TEK	>	10000
TPKI-39	O=C(NC1=CC=CC=C1)NC1=CC=CC(OC2=NN3C=CN=C3C=C2)=C1	10	YSK4	MAP3K19	=	270
TPKI-48	CCC1=CC=C(C=C1)C1=CC2=C(C=C1)N(C)S(=O)(=O)C1=CNN=C21	4	CDK4-cyclin	CDK4	>	10000
TPKI-48	CCC1=CC=C(C=C1)C1=CC2=C(C=C1)N(C)S(=O)(=O)C1=CNN=C21	4	MEK2	MAP2K2	>	10000
TPKI-48	CCC1=CC=C(C=C1)C1=CC2=C(C=C1)N(C)S(=O)(=O)C1=CNN=C21	4	PIKFYVE	PIKFYVE	>	10000
TPKI-49	CN1C2=C(C=C(C=C2)C2=CC=C(C=C2)C(N)=O)C2=C(C=NN2)S1(=O)=O	2	CAMKK1	CAMKK1	>	10000
TPKI-49	CN1C2=C(C=C(C=C2)C2=CC=C(C=C2)C(N)=O)C2=C(C=NN2)S1(=O)=O	2	PIP5K1C	PIP5K1C	=	6400
TPKI-49	CN1C2=C(C=C(C=C2)C2=CC=C(C=C2)C(N)=O)C2=C(C=NN2)S1(=O)=O	2	PRKD2	PRKD2	>	10000
TPKI-49	CN1C2=C(C=C(C=C2)C2=CC=C(C=C2)C(N)=O)C2=C(C=NN2)S1(=O)=O	2	TYK2(JH2do	TYK2	=	930
TPKI-50	CN1C2=C(C=C(C=C2)C2=CC=C(C=C2)C(=O)NCCO)C2=C(C=NN2)S1(=O)=O	2	CLK2	CLK2	=	700
TPKI-50	CN1C2=C(C=C(C=C2)C2=CC=C(C=C2)C(=O)NCCO)C2=C(C=NN2)S1(=O)=O	2	JAK3(JH1do	JAK3	=	6800
TPKI-50	CN1C2=C(C=C(C=C2)C2=CC=C(C=C2)C(=O)NCCO)C2=C(C=NN2)S1(=O)=O	2	MYLK	MYLK	=	480
TPKI-50	CN1C2=C(C=C(C=C2)C2=CC=C(C=C2)C(=O)NCCO)C2=C(C=NN2)S1(=O)=O	2	TYK2(JH2do	TYK2	=	130
TPKI-52	CCC1=CC=C(C=C1)C1=CC=C2N(C)S(=O)(=O)C3=CN(C)N=C3C2=C1	1	MYO3A	MYO3A	>	10000
TPKI-53	CN1N=CC2=C1C1=C(C=CC(CNC3=CC=C(F)C=C3)=C1)N(C)S2(=O)=O	1	SGK2	SGK2	>	10000
TPKI-54	CN1N=CC2=C1C1=C(C=CC(NCC3=CC=C(F)C=C3)=C1)N(C)S2(=O)=O	1	RSK4(Kin.Dc	RPS6KA6	>	10000
TPKI-55	CC(C)(C)C1=CC=C(C=C1)C(=O)NC1=CN2C=C(Cl)C=CC2=N1	7	ASK1	MAP3K5	=	1500
TPKI-55	CC(C)(C)C1=CC=C(C=C1)C(=O)NC1=CN2C=C(Cl)C=CC2=N1	7	CAMKK1	CAMKK1	>	10000
TPKI-55	CC(C)(C)C1=CC=C(C=C1)C(=O)NC1=CN2C=C(Cl)C=CC2=N1	7	FYN	FYN	>	10000
TPKI-55	CC(C)(C)C1=CC=C(C=C1)C(=O)NC1=CN2C=C(Cl)C=CC2=N1	7	IKK-beta	IKBKB	>	10000
TPKI-55	CC(C)(C)C1=CC=C(C=C1)C(=O)NC1=CN2C=C(Cl)C=CC2=N1	7	NDR1	STK38	>	10000
TPKI-55	CC(C)(C)C1=CC=C(C=C1)C(=O)NC1=CN2C=C(Cl)C=CC2=N1	7	PRKCI	PRKCI	>	10000
TPKI-55	CC(C)(C)C1=CC=C(C=C1)C(=O)NC1=CN2C=C(Cl)C=CC2=N1	7	PYK2	PTK2B	>	10000
TPKI-56	CNCC1=CN2C=C(NC(=O)C3=CC=C(C=C3)C(C)(C)C)N=C2C=C1	4	CLK1	CLK1	=	7500
TPKI-56	CNCC1=CN2C=C(NC(=O)C3=CC=C(C=C3)C(C)(C)C)N=C2C=C1	4	CSNK1G3	CSNK1G3	>	10000
TPKI-56	CNCC1=CN2C=C(NC(=O)C3=CC=C(C=C3)C(C)(C)C)N=C2C=C1	4	MAP3K15	MAP3K15	=	3800
TPKI-56	CNCC1=CN2C=C(NC(=O)C3=CC=C(C=C3)C(C)(C)C)N=C2C=C1	4	p38-alpha	MAPK14	>	10000

TPKI-56	CNCC1=CN2C=C(NC(=O)C3=CC=C(C=C3)C(C)(C)C)N=C2C=C1	4	PIK3C2B	PIK3C2B	>	10000
TPKI-56	CNCC1=CN2C=C(NC(=O)C3=CC=C(C=C3)C(C)(C)C)N=C2C=C1	4	PIK3CA	PIK3CA	>	10000
TPKI-56	CNCC1=CN2C=C(NC(=O)C3=CC=C(C=C3)C(C)(C)C)N=C2C=C1	4	RIOK2	RIOK2	>	10000
TPKI-56	CNCC1=CN2C=C(NC(=O)C3=CC=C(C=C3)C(C)(C)C)N=C2C=C1	4	TAOK1	TAOK1	>	10000
TPKI-56	CNCC1=CN2C=C(NC(=O)C3=CC=C(C=C3)C(C)(C)C)N=C2C=C1	4	TAOK3	TAOK3	>	10000
TPKI-57	CC(C)(C)C1=CC=C(C=C1)C(=O)NC1=CN2C=C(C=CC2=N1)C1=CC=CC=C1	4	KIT	KIT	>	10000
TPKI-57	CC(C)(C)C1=CC=C(C=C1)C(=O)NC1=CN2C=C(C=CC2=N1)C1=CC=CC=C1	4	PDGFRB	PDGFRB	=	3700
TPKI-57	CC(C)(C)C1=CC=C(C=C1)C(=O)NC1=CN2C=C(C=CC2=N1)C1=CC=CC=C1	4	RSK3(Kin.Do	RPS6KA2	>	10000
TPKI-58	CC(C)(C)C1=CC=C(C=C1)C(=O)NC1=CN2C=C(C=CC2=N1)C1=CC=CN=C1	6	ASK1	MAP3K5	=	64
TPKI-58	CC(C)(C)C1=CC=C(C=C1)C(=O)NC1=CN2C=C(C=CC2=N1)C1=CC=CN=C1	6	CLK1	CLK1	=	280
TPKI-58	CC(C)(C)C1=CC=C(C=C1)C(=O)NC1=CN2C=C(C=CC2=N1)C1=CC=CN=C1	6	CSNK1E	CSNK1E	=	110
TPKI-58	CC(C)(C)C1=CC=C(C=C1)C(=O)NC1=CN2C=C(C=CC2=N1)C1=CC=CN=C1	6	KIT	KIT	=	320
TPKI-58	CC(C)(C)C1=CC=C(C=C1)C(=O)NC1=CN2C=C(C=CC2=N1)C1=CC=CN=C1	6	MEK5	MAP2K5	=	190
TPKI-58	CC(C)(C)C1=CC=C(C=C1)C(=O)NC1=CN2C=C(C=CC2=N1)C1=CC=CN=C1	6	PDGFRB	PDGFRB	=	220
TPKI-60	ClC1=C(OC2=CC=C(C=C2)C#N)C=CC(NC2=NC=NC3=C2NC=C3)=C1	3	EGFR	EGFR	=	97
TPKI-61	FC1=CC(COC2=CC=C(NC3=NC=NC4=C3NC=C4)C=C2Cl)=CC=C1	9	EGFR	EGFR	=	1.3
TPKI-61	FC1=CC(COC2=CC=C(NC3=NC=NC4=C3NC=C4)C=C2Cl)=CC=C1	9	ERBB2	ERBB2	=	2.2
TPKI-61	FC1=CC(COC2=CC=C(NC3=NC=NC4=C3NC=C4)C=C2Cl)=CC=C1	9	ERBB4	ERBB4	=	320
TPKI-62	ClC1=C(OCC2=NC=CC=C2)C=CC(NC2=NC=NC3=C2NC=C3)=C1	6	EGFR	EGFR	=	8.4
TPKI-62	ClC1=C(OCC2=NC=CC=C2)C=CC(NC2=NC=NC3=C2NC=C3)=C1	6	ERBB2	ERBB2	=	9
TPKI-63	CC1=CC=C(OC2=C(C)C=C(NC3=NC=NC4=C3NC=C4)C=C2)C=N1	5	ERBB2	ERBB2	=	6.5
TPKI-63	CC1=CC=C(OC2=C(C)C=C(NC3=NC=NC4=C3NC=C4)C=C2)C=N1	5	FLT1	FLT1	>	10000
TPKI-63	CC1=CC=C(OC2=C(C)C=C(NC3=NC=NC4=C3NC=C4)C=C2)C=N1	5	MEK5	MAP2K5	=	9
TPKI-63	CC1=CC=C(OC2=C(C)C=C(NC3=NC=NC4=C3NC=C4)C=C2)C=N1	5	RIOK1	RIOK1	>	10000
TPKI-64	CN1C=CC2=C1C(NC1=CC(Cl)=C(OC3=CC=CC(Cl)=C3)C=C1)=NC=N2	10	EGFR	EGFR	=	4.3
TPKI-64	CN1C=CC2=C1C(NC1=CC(Cl)=C(OC3=CC=CC(Cl)=C3)C=C1)=NC=N2	10	EPHA7	EPHA7	>	10000
TPKI-64	CN1C=CC2=C1C(NC1=CC(Cl)=C(OC3=CC=CC(Cl)=C3)C=C1)=NC=N2	10	EPHA8	EPHA8	>	10000

TPKI-64	CN1C=CC2=C1C(NC1=CC(Cl)=C(OC3=CC=CC(Cl)=C3)C=C1)=NC=N2	10	ERBB2	ERBB2	=	1.2
TPKI-64	CN1C=CC2=C1C(NC1=CC(Cl)=C(OC3=CC=CC(Cl)=C3)C=C1)=NC=N2	10	ERBB4	ERBB4	=	74
TPKI-64	CN1C=CC2=C1C(NC1=CC(Cl)=C(OC3=CC=CC(Cl)=C3)C=C1)=NC=N2	10	MEK5	MAP2K5	=	25
TPKI-64	CN1C=CC2=C1C(NC1=CC(Cl)=C(OC3=CC=CC(Cl)=C3)C=C1)=NC=N2	10	TEC	TEC	>	10000
TPKI-65	OCCOCCN1C=CC2=C1C(NC1=CC(Cl)=C(OC3=CC=CC(Cl)=C3)C=C1)=NC=N2	8	EGFR	EGFR	=	8.5
TPKI-65	OCCOCCN1C=CC2=C1C(NC1=CC(Cl)=C(OC3=CC=CC(Cl)=C3)C=C1)=NC=N2	8	ERBB2	ERBB2	=	2.9
TPKI-65	OCCOCCN1C=CC2=C1C(NC1=CC(Cl)=C(OC3=CC=CC(Cl)=C3)C=C1)=NC=N2	8	ERBB4	ERBB4	=	77
TPKI-65	OCCOCCN1C=CC2=C1C(NC1=CC(Cl)=C(OC3=CC=CC(Cl)=C3)C=C1)=NC=N2	8	MEK5	MAP2K5	=	200
TPKI-66	OCCC(=O)NCCN1C=CC2=C1C(NC1=CC(Cl)=C(OC3=CC=CC(=C3)C(F)(F)F)C=C1)=NC=N2	9	EGFR	EGFR	=	2.3
TPKI-66	OCCC(=O)NCCN1C=CC2=C1C(NC1=CC(Cl)=C(OC3=CC=CC(=C3)C(F)(F)F)C=C1)=NC=N2	9	ERBB2	ERBB2	=	0.88
TPKI-66	OCCC(=O)NCCN1C=CC2=C1C(NC1=CC(Cl)=C(OC3=CC=CC(=C3)C(F)(F)F)C=C1)=NC=N2	9	ERBB4	ERBB4	=	100
TPKI-66	OCCC(=O)NCCN1C=CC2=C1C(NC1=CC(Cl)=C(OC3=CC=CC(=C3)C(F)(F)F)C=C1)=NC=N2	9	MEK5	MAP2K5	=	15
TPKI-69	CN1C=CC2=C1C(OC1=CC=C(NC(=O)NC3=CC=CC=C3)C=C1)=NC=N2	13	AURKB	AURKB	=	110
TPKI-69	CN1C=CC2=C1C(OC1=CC=C(NC(=O)NC3=CC=CC=C3)C=C1)=NC=N2	13	AURKC	AURKC	=	110
TPKI-69	CN1C=CC2=C1C(OC1=CC=C(NC(=O)NC3=CC=CC=C3)C=C1)=NC=N2	13	CSF1R	CSF1R	=	82
TPKI-69	CN1C=CC2=C1C(OC1=CC=C(NC(=O)NC3=CC=CC=C3)C=C1)=NC=N2	13	FLT1	FLT1	=	350
TPKI-69	CN1C=CC2=C1C(OC1=CC=C(NC(=O)NC3=CC=CC=C3)C=C1)=NC=N2	13	FLT3	FLT3	=	33
TPKI-69	CN1C=CC2=C1C(OC1=CC=C(NC(=O)NC3=CC=CC=C3)C=C1)=NC=N2	13	KIT	KIT	=	0.6
TPKI-69	CN1C=CC2=C1C(OC1=CC=C(NC(=O)NC3=CC=CC=C3)C=C1)=NC=N2	13	LOK	STK10	=	280
TPKI-69	CN1C=CC2=C1C(OC1=CC=C(NC(=O)NC3=CC=CC=C3)C=C1)=NC=N2	13	PDGFRA	PDGFRA	=	25
TPKI-69	CN1C=CC2=C1C(OC1=CC=C(NC(=O)NC3=CC=CC=C3)C=C1)=NC=N2	13	PDGFRB	PDGFRB	=	16
TPKI-69	CN1C=CC2=C1C(OC1=CC=C(NC(=O)NC3=CC=CC=C3)C=C1)=NC=N2	13	VEGFR2	KDR	=	45
TPKI-69	CN1C=CC2=C1C(OC1=CC=C(NC(=O)NC3=CC=CC=C3)C=C1)=NC=N2	13	YSK4	MAP3K19	=	170
TPKI-70	CN1C=CC2=C1C(OC1=CC(=CC=C1)C(=O)NC1=CC=CC=C1)=NC=N2	2	ACVR1	ACVR1	>	10000
TPKI-70	CN1C=CC2=C1C(OC1=CC(=CC=C1)C(=O)NC1=CC=CC=C1)=NC=N2	2	LIMK1	LIMK1	>	10000
TPKI-71	CCCNC(=O)NC1=CC=C(OC2=NC=NC3=C2N(C)C=C3)C=C1	1	KIT	KIT	=	67
TPKI-72	CN1C=CC2=C1C(OC1=CC=C(NC3=NC4=C(N3)C=CC=C4)C=C1)=NC=N2	5	AURKB	AURKB	=	59
TPKI-72	CN1C=CC2=C1C(OC1=CC=C(NC3=NC4=C(N3)C=CC=C4)C=C1)=NC=N2	5	LOK	STK10	=	200

TPKI-72	CN1C=CC2=C1C(OC1=CC=C(NC3=NC4=C(N3)C=CC=C4)C=C1)=NC=N2	5	NEK4	NEK4	>	10000
TPKI-72	CN1C=CC2=C1C(OC1=CC=C(NC3=NC4=C(N3)C=CC=C4)C=C1)=NC=N2	5	TRPM6	TRPM6	>	10000
TPKI-73	CN1C=CC2=C1C(OC1=CC=C(NC(=O)NC3=CC=CN=C3)C(Cl)=C1)=NC=N2	8	CSF1R	CSF1R	=	290
TPKI-73	CN1C=CC2=C1C(OC1=CC=C(NC(=O)NC3=CC=CN=C3)C(Cl)=C1)=NC=N2	8	DDR1	DDR1	=	57
TPKI-73	CN1C=CC2=C1C(OC1=CC=C(NC(=O)NC3=CC=CN=C3)C(Cl)=C1)=NC=N2	8	DDR2	DDR2	=	76
TPKI-73	CN1C=CC2=C1C(OC1=CC=C(NC(=O)NC3=CC=CN=C3)C(Cl)=C1)=NC=N2	8	KIT	KIT	=	47
TPKI-73	CN1C=CC2=C1C(OC1=CC=C(NC(=O)NC3=CC=CN=C3)C(Cl)=C1)=NC=N2	8	PDGFRA	PDGFRA	=	110
TPKI-73	CN1C=CC2=C1C(OC1=CC=C(NC(=O)NC3=CC=CN=C3)C(Cl)=C1)=NC=N2	8	PDGFRB	PDGFRB	=	95
TPKI-74	CN1C=CC2=C1C(OC1=CC=C(NC(=O)NC3=CC=NC=C3)C(Cl)=C1)=NC=N2	1	STK39	STK39	>	10000
TPKI-79	COC1=CC=C(C=C1)C1=COC2=CC=C(C=C12)C1=NN=C(C)O1	3	BRSK2	BRSK2	>	10000
TPKI-79	COC1=CC=C(C=C1)C1=COC2=CC=C(C=C12)C1=NN=C(C)O1	3	MARK1	MARK1	>	10000
TPKI-79	COC1=CC=C(C=C1)C1=COC2=CC=C(C=C12)C1=NN=C(C)O1	3	MARK4	MARK4	>	10000
TPKI-80	CC(O)C1=CC=C(C=C1)C1=COC2=C1C=C(C=C2)C1=NN=C(C)O1	1	PIM2	PIM2	=	940
TPKI-80	CC(O)C1=CC=C(C=C1)C1=COC2=C1C=C(C=C2)C1=NN=C(C)O1	1	RAF1	RAF1	>	10000
TPKI-81	CCS(=O)C1=CC=C(C=C1)C1=COC2=C1C=C(C=C2)C1=NN=C(C)O1	2	GSK3A	GSK3A	=	260
TPKI-81	CCS(=O)C1=CC=C(C=C1)C1=COC2=C1C=C(C=C2)C1=NN=C(C)O1	2	PIP5K2C	PIP4K2C	=	85
TPKI-82	CC1=NN=C(O1)C1=CC2=C(C=C1)N=CN2C1=CC=C(C=C1)S(C)=O	3	MAP4K2	MAP4K2	>	10000
TPKI-82	CC1=NN=C(O1)C1=CC2=C(C=C1)N=CN2C1=CC=C(C=C1)S(C)=O	3	TAOK1	TAOK1	>	10000
TPKI-82	CC1=NN=C(O1)C1=CC2=C(C=C1)N=CN2C1=CC=C(C=C1)S(C)=O	3	TAOK3	TAOK3	>	10000
TPKI-83	CC1=NN=C(O1)C1=CN2C(C=C1)=NC=C2C1=CC=C(C=C1)S(C)=O	0	PDGFRB	PDGFRB	=	490
TPKI-85	CC1=NN=C(N1)C1=CC=C2OC=C(C2=C1)C1=CC=C(C=C1)S(C)=O	4	AMPK-alpha	PRKAA1	>	10000
TPKI-85	CC1=NN=C(N1)C1=CC=C2OC=C(C2=C1)C1=CC=C(C=C1)S(C)=O	4	GSK3A	GSK3A	=	190
TPKI-85	CC1=NN=C(N1)C1=CC=C2OC=C(C2=C1)C1=CC=C(C=C1)S(C)=O	4	HIPK2	HIPK2	=	320
TPKI-85	CC1=NN=C(N1)C1=CC=C2OC=C(C2=C1)C1=CC=C(C=C1)S(C)=O	4	HIPK3	HIPK3	=	340
TPKI-85	CC1=NN=C(N1)C1=CC=C2OC=C(C2=C1)C1=CC=C(C=C1)S(C)=O	4	JAK1(JH2do	JAK1	=	190
TPKI-85	CC1=NN=C(N1)C1=CC=C2OC=C(C2=C1)C1=CC=C(C=C1)S(C)=O	4	LOK	STK10	>	10000
TPKI-85	CC1=NN=C(N1)C1=CC=C2OC=C(C2=C1)C1=CC=C(C=C1)S(C)=O	4	RIPK1	RIPK1	>	10000

TPKI-85	CC1=NN=C(N1)C1=CC=C2OC=C(C2=C1)C1=CC=C(C=C1)S(C)=O	4	VPS34	PIK3C3	=	62
TPKI-88	FC1=CC(CSC2=NN=C(O2)C2=CC3=C(OCC3)C=C2)=CC=C1	1	IRAK4	IRAK4	>	10000
TPKI-89	COC1=CC=C(CSC2=NN=C(O2)C2=CC3=C(OCC3)C=C2)C=C1	0	CDC2L5	CDK13	>	10000
TPKI-89	COC1=CC=C(CSC2=NN=C(O2)C2=CC3=C(OCC3)C=C2)C=C1	0	CLK4	CLK4	=	1300
TPKI-90	FC1=CC(COC2=NN=C(O2)C2=CC3=C(OCC3)C=C2)=CC=C1	1	ACVR2B	ACVR2B	>	10000
TPKI-91	COC1=CC=C(CSC2=NN=C(O2)C2=CC3=C(C=C2)N=CS3)C=C1C(F)(F)F	2	CLK1	CLK1	=	170
TPKI-91	COC1=CC=C(CSC2=NN=C(O2)C2=CC3=C(C=C2)N=CS3)C=C1C(F)(F)F	2	CLK4	CLK4	=	1100
TPKI-91	COC1=CC=C(CSC2=NN=C(O2)C2=CC3=C(C=C2)N=CS3)C=C1C(F)(F)F	2	GSK3A	GSK3A	=	79
TPKI-91	COC1=CC=C(CSC2=NN=C(O2)C2=CC3=C(C=C2)N=CS3)C=C1C(F)(F)F	2	GSK3B	GSK3B	=	39
TPKI-92	COC1=C(C=C(CSC2=NN=C(O2)C2=CN3C=CN=C3C=C2)C=C1)C(F)(F)F	6	CLK1	CLK1	=	70
TPKI-92	COC1=C(C=C(CSC2=NN=C(O2)C2=CN3C=CN=C3C=C2)C=C1)C(F)(F)F	6	CLK2	CLK2	=	150
TPKI-92	COC1=C(C=C(CSC2=NN=C(O2)C2=CN3C=CN=C3C=C2)C=C1)C(F)(F)F	6	CLK4	CLK4	=	130
TPKI-92	COC1=C(C=C(CSC2=NN=C(O2)C2=CN3C=CN=C3C=C2)C=C1)C(F)(F)F	6	GSK3A	GSK3A	=	250
TPKI-92	COC1=C(C=C(CSC2=NN=C(O2)C2=CN3C=CN=C3C=C2)C=C1)C(F)(F)F	6	GSK3B	GSK3B	=	22
TPKI-92	COC1=C(C=C(CSC2=NN=C(O2)C2=CN3C=CN=C3C=C2)C=C1)C(F)(F)F	6	YSK4	MAP3K19	=	22
TPKI-94	COC1=CC=C(C=C1)C1=COC2=C1C=C(C=C2)C1=NN=C(O1)SCC1=CC=CC(=C1)C#N	1	GSK3A	GSK3A	=	150
TPKI-94	COC1=CC=C(C=C1)C1=COC2=C1C=C(C=C2)C1=NN=C(O1)SCC1=CC=CC(=C1)C#N	1	ULK3	ULK3	>	10000
TPKI-95	COC(=O)C1=C(C2=CC=CC=C2)C2=CC(Br)=CC=C2C(=O)N1CC1=CC=C(NC(=O)CCC(O)=O)C=C1	6	JNK1	MAPK8	=	58
TPKI-95	COC(=O)C1=C(C2=CC=CC=C2)C2=CC(Br)=CC=C2C(=O)N1CC1=CC=C(NC(=O)CCC(O)=O)C=C1	6	JNK2	MAPK9	=	66
TPKI-95	COC(=O)C1=C(C2=CC=CC=C2)C2=CC(Br)=CC=C2C(=O)N1CC1=CC=C(NC(=O)CCC(O)=O)C=C1	6	JNK3	MAPK10	=	13
TPKI-95	COC(=O)C1=C(C2=CC=CC=C2)C2=CC(Br)=CC=C2C(=O)N1CC1=CC=C(NC(=O)CCC(O)=O)C=C1	6	MYO3A	MYO3A	>	10000
TPKI-96	COC(=O)C1=C(C2=CC=CC=C2)C2=CC(C)=CC=C2C(=O)N1CC1=CC=CC=C1	3	NIK	MAP3K14	>	10000
TPKI-96	COC(=O)C1=C(C2=CC=CC=C2)C2=CC(C)=CC=C2C(=O)N1CC1=CC=CC=C1	3	PDGFRB	PDGFRB	=	170
TPKI-97	COC(=O)C1=C(C2=CC=CC=C2)C2=CC(OC)=CC=C2C(=O)N1CC1=CC=CC=C1	4	BRAF	BRAF	=	61
TPKI-97	COC(=O)C1=C(C2=CC=CC=C2)C2=CC(OC)=CC=C2C(=O)N1CC1=CC=CC=C1	4	DDR1	DDR1	=	280
TPKI-97	COC(=O)C1=C(C2=CC=CC=C2)C2=CC(OC)=CC=C2C(=O)N1CC1=CC=CC=C1	4	JNK3	MAPK10	=	110
TPKI-97	COC(=O)C1=C(C2=CC=CC=C2)C2=CC(OC)=CC=C2C(=O)N1CC1=CC=CC=C1	4	KIT	KIT	=	170
TPKI-97	COC(=O)C1=C(C2=CC=CC=C2)C2=CC(OC)=CC=C2C(=O)N1CC1=CC=CC=C1	4	PDGFRB	PDGFRB	=	110

TPKI-97	COC(=O)C1=C(C2=CC=CC=C2)C2=CC(OC)=CC=C2C(=O)N1CC1=CC=CC=C1	4	RAF1	RAF1	=	390
TPKI-98	COC(=O)C1=C(C2=CC=CC=C2)C2=CC(=CC=C2C(=O)N1CC1=CC=CC=C1)C(O)=O	8	BRAF	BRAF	=	50
TPKI-98	COC(=O)C1=C(C2=CC=CC=C2)C2=CC(=CC=C2C(=O)N1CC1=CC=CC=C1)C(O)=O	8	DDR1	DDR1	=	160
TPKI-98	COC(=O)C1=C(C2=CC=CC=C2)C2=CC(=CC=C2C(=O)N1CC1=CC=CC=C1)C(O)=O	8	KIT	KIT	=	100
TPKI-98	COC(=O)C1=C(C2=CC=CC=C2)C2=CC(=CC=C2C(=O)N1CC1=CC=CC=C1)C(O)=O	8	PDGFRB	PDGFRB	=	75
TPKI-98	COC(=O)C1=C(C2=CC=CC=C2)C2=CC(=CC=C2C(=O)N1CC1=CC=CC=C1)C(O)=O	8	RAF1	RAF1	=	170
TPKI-99	COC(=O)C1=C(C2=CC=CC=C2)C2=CC(Cl)=CC=C2C(=O)N1CC1=CC=C(C=C1)S(C)(=O)=O	5	CSNK1G1	CSNK1G1	>	10000
TPKI-99	COC(=O)C1=C(C2=CC=CC=C2)C2=CC(Cl)=CC=C2C(=O)N1CC1=CC=C(C=C1)S(C)(=O)=O	5	GAK	GAK	>	10000
TPKI-99	COC(=O)C1=C(C2=CC=CC=C2)C2=CC(Cl)=CC=C2C(=O)N1CC1=CC=C(C=C1)S(C)(=O)=O	5	JNK1	MAPK8	=	61
TPKI-99	COC(=O)C1=C(C2=CC=CC=C2)C2=CC(Cl)=CC=C2C(=O)N1CC1=CC=C(C=C1)S(C)(=O)=O	5	JNK2	MAPK9	=	44
TPKI-99	COC(=O)C1=C(C2=CC=CC=C2)C2=CC(Cl)=CC=C2C(=O)N1CC1=CC=C(C=C1)S(C)(=O)=O	5	JNK3	MAPK10	=	14
TPKI-99	COC(=O)C1=C(C2=CC=CC=C2)C2=CC(Cl)=CC=C2C(=O)N1CC1=CC=C(C=C1)S(C)(=O)=O	5	NEK2	NEK2	>	10000
UNC10112604A	Cl.CS(=O)(=O)CCNCc1ccc(o1)-c1ccc2ncnc(Nc3ccc(OCc4ccccc4)c(Cl)c3)c2c1	13	EGFR	EGFR	=	0.82
UNC10112604A	Cl.CS(=O)(=O)CCNCc1ccc(o1)-c1ccc2ncnc(Nc3ccc(OCc4ccccc4)c(Cl)c3)c2c1	13	ERBB2	ERBB2	=	2.8
UNC10112604A	Cl.CS(=O)(=O)CCNCc1ccc(o1)-c1ccc2ncnc(Nc3ccc(OCc4ccccc4)c(Cl)c3)c2c1	13	ERBB4	ERBB4	=	28
UNC10112604A	Cl.CS(=O)(=O)CCNCc1ccc(o1)-c1ccc2ncnc(Nc3ccc(OCc4ccccc4)c(Cl)c3)c2c1	13	MEK5	MAP2K5	=	1000
UNC10112604A	Cl.CS(=O)(=O)CCNCc1ccc(o1)-c1ccc2ncnc(Nc3ccc(OCc4ccccc4)c(Cl)c3)c2c1	13	NIK	MAP3K14	>	10000
UNC10112764A	Cl.NS(=O)(=O)c1cccc(Nc2cc(n[nH]2)-c2ccc(F)cc2)c1	2	FLT3	FLT3	=	7.5
UNC10112764A	Cl.NS(=O)(=O)c1cccc(Nc2cc(n[nH]2)-c2ccc(F)cc2)c1	2	KIT	KIT	=	16
UNC10112764A	Cl.NS(=O)(=O)c1cccc(Nc2cc(n[nH]2)-c2ccc(F)cc2)c1	2	PDGFRB	PDGFRB	=	210
UNC10112764A	Cl.NS(=O)(=O)c1cccc(Nc2cc(n[nH]2)-c2ccc(F)cc2)c1	2	SGK2	SGK2	>	10000
UNC10112830A	CCn1c(nc2ccccc12)-c1nonc1N	0	LATS2	LATS2	=	1800
UNC10112830A	CCn1c(nc2ccccc12)-c1nonc1N	0	LRRK2	LRRK2	>	10000
UNC10112830A	CCn1c(nc2ccccc12)-c1nonc1N	0	YSK4	MAP3K19	=	10
UNC10224985A	Cc1ccc(NC(=O)c2ccc(Cl)cc2)cc1-c1ccc(cc1)C(=O)Nc1ccncc1	8	CAMK2A	CAMK2A	>	10000
UNC10224985A	Cc1ccc(NC(=O)c2ccc(Cl)cc2)cc1-c1ccc(cc1)C(=O)Nc1ccncc1	8	CAMK2B	CAMK2B	>	10000
UNC10224985A	Cc1ccc(NC(=O)c2ccc(Cl)cc2)cc1-c1ccc(cc1)C(=O)Nc1ccncc1	8	CLK2	CLK2	>	10000
UNC10224985A	Cc1ccc(NC(=O)c2ccc(Cl)cc2)cc1-c1ccc(cc1)C(=O)Nc1ccncc1	8	CSF1R	CSF1R	=	880
UNC10224985A	Cc1ccc(NC(=O)c2ccc(Cl)cc2)cc1-c1ccc(cc1)C(=O)Nc1ccncc1	8	DDR1	DDR1	=	710
UNC10224985A	Cc1ccc(NC(=O)c2ccc(Cl)cc2)cc1-c1ccc(cc1)C(=O)Nc1ccncc1	8	EPHA3	EPHA3	>	10000
UNC10224985A	Cc1ccc(NC(=O)c2ccc(Cl)cc2)cc1-c1ccc(cc1)C(=O)Nc1ccncc1	8	KIT	KIT	=	170

UNC10224985A	Cc1ccc(NC(=O)c2ccc(Cl)cc2)cc1-c1ccc(cc1)C(=O)Nc1ccncc1	8	LRRK2	LRRK2	>	10000
UNC10224985A	Cc1ccc(NC(=O)c2ccc(Cl)cc2)cc1-c1ccc(cc1)C(=O)Nc1ccncc1	8	MARK2	MARK2	>	10000
UNC10224985A	Cc1ccc(NC(=O)c2ccc(Cl)cc2)cc1-c1ccc(cc1)C(=O)Nc1ccncc1	8	PDGFRB	PDGFRB	=	9.7
UNC10224990A	Cl.Fc1cccc(c1)S(=O)(=O)c1ccc(Nc2ncnc3cc(Br)sc23)cc1Cl	12	EGFR	EGFR	=	61
UNC10224990A	Cl.Fc1cccc(c1)S(=O)(=O)c1ccc(Nc2ncnc3cc(Br)sc23)cc1Cl	12	ERBB2	ERBB2	=	95
UNC10224990A	Cl.Fc1cccc(c1)S(=O)(=O)c1ccc(Nc2ncnc3cc(Br)sc23)cc1Cl	12	FLT4	FLT4	>	10000
UNC10224990A	Cl.Fc1cccc(c1)S(=O)(=O)c1ccc(Nc2ncnc3cc(Br)sc23)cc1Cl	12	RET	RET	>	10000
UNC10224990A	Cl.Fc1cccc(c1)S(=O)(=O)c1ccc(Nc2ncnc3cc(Br)sc23)cc1Cl	12	SRPK1	SRPK1	>	10000
UNC10224992A	Cc1ccc(NC(=O)Nc2ccccc2)cc1-c1ccc(cc1)C(=O)Nc1ccncc1	8	CIT	CIT	=	540
UNC10224992A	Cc1ccc(NC(=O)Nc2ccccc2)cc1-c1ccc(cc1)C(=O)Nc1ccncc1	8	CSF1R	CSF1R	=	3300
UNC10224992A	Cc1ccc(NC(=O)Nc2ccccc2)cc1-c1ccc(cc1)C(=O)Nc1ccncc1	8	DDR1	DDR1	=	180
UNC10224992A	Cc1ccc(NC(=O)Nc2ccccc2)cc1-c1ccc(cc1)C(=O)Nc1ccncc1	8	KIT	KIT	=	140
UNC10224992A	Cc1ccc(NC(=O)Nc2ccccc2)cc1-c1ccc(cc1)C(=O)Nc1ccncc1	8	PDGFRB	PDGFRB	=	28
UNC10224992A	Cc1ccc(NC(=O)Nc2ccccc2)cc1-c1ccc(cc1)C(=O)Nc1ccncc1	8	YSK4	MAP3K19	=	71
UNC10224993A	Cl.Cc1cc(nc(Nc2ccc(cc2)S(=O)(=O)N2CCOCC2)n1)N1Cc2ccccc2C1	8	AKT1	AKT1	>	10000
UNC10224993A	Cl.Cc1cc(nc(Nc2ccc(cc2)S(=O)(=O)N2CCOCC2)n1)N1Cc2ccccc2C1	8	DAPK3	DAPK3	>	10000
UNC10224993A	Cl.Cc1cc(nc(Nc2ccc(cc2)S(=O)(=O)N2CCOCC2)n1)N1Cc2ccccc2C1	8	ICK	ICK	>	10000
UNC10224993A	Cl.Cc1cc(nc(Nc2ccc(cc2)S(=O)(=O)N2CCOCC2)n1)N1Cc2ccccc2C1	8	INSR	INSR	>	10000
UNC10224993A	Cl.Cc1cc(nc(Nc2ccc(cc2)S(=O)(=O)N2CCOCC2)n1)N1Cc2ccccc2C1	8	PIK4CB	PI4KB	>	10000
UNC10224993A	Cl.Cc1cc(nc(Nc2ccc(cc2)S(=O)(=O)N2CCOCC2)n1)N1Cc2ccccc2C1	8	RIPK4	RIPK4	>	10000
UNC10224994A	CNc1nc(cc(n1)-c1ccc2c(N)n[nH]c2c1)N1C[C@H](OC[C@H]1C)C(=O)NC1CCCC1	1	CDK4	CDK4	>	10000
UNC10224995A	CS(=O)(=O)c1ccc(cc1)-c1nc2CCCn2c1-c1ccncc1	12	AKT2	AKT2	>	10000
UNC10224995A	CS(=O)(=O)c1ccc(cc1)-c1nc2CCCn2c1-c1ccncc1	12	CDC2L1	CDK11B	>	10000
UNC10224995A	CS(=O)(=O)c1ccc(cc1)-c1nc2CCCn2c1-c1ccncc1	12	CDK9	CDK9	>	10000
UNC10224995A	CS(=O)(=O)c1ccc(cc1)-c1nc2CCCn2c1-c1ccncc1	12	CSK	CSK	>	10000
UNC10224995A	CS(=O)(=O)c1ccc(cc1)-c1nc2CCCn2c1-c1ccncc1	12	ERK4	MAPK4	>	10000
UNC10224995A	CS(=O)(=O)c1ccc(cc1)-c1nc2CCCn2c1-c1ccncc1	12	ICK	ICK	>	10000
UNC10224995A	CS(=O)(=O)c1ccc(cc1)-c1nc2CCCn2c1-c1ccncc1	12	PAK6	PAK6	>	10000
UNC10224995A	CS(=O)(=O)c1ccc(cc1)-c1nc2CCCn2c1-c1ccncc1	12	PIK4CB	PI4KB	>	10000
UNC10224995A	CS(=O)(=O)c1ccc(cc1)-c1nc2CCCn2c1-c1ccncc1	12	RIPK4	RIPK4	>	10000
UNC10224995A	CS(=O)(=O)c1ccc(cc1)-c1nc2CCCn2c1-c1ccncc1	12	STK16	STK16	>	10000
UNC10224995A	CS(=O)(=O)c1ccc(cc1)-c1nc2CCCn2c1-c1ccncc1	12	TSSK1B	TSSK1B	>	10000
UNC10224995A	CS(=O)(=O)c1ccc(cc1)-c1nc2CCCn2c1-c1ccncc1	12	YANK3	STK32C	>	10000
UNC10224997A	Cc1cccc(n1)-c1nc(Nc2ccnc(C)c2)c2ccccc2n1	2	JAK1(JH2do	JAK1	=	410
UNC10224997A	Cc1cccc(n1)-c1nc(Nc2ccnc(C)c2)c2ccccc2n1	2	PRKD1	PRKD1	=	6.1
UNC10224997A	Cc1cccc(n1)-c1nc(Nc2ccnc(C)c2)c2ccccc2n1	2	PRKD2	PRKD2	=	9.4
UNC10224997A	Cc1cccc(n1)-c1nc(Nc2ccnc(C)c2)c2ccccc2n1	2	TGFBR1	TGFBR1	=	270
UNC10224998A	CCCc1ccc(cc1)S(=O)(=O)NC1CCN(C1)c1ccnc(Nc2ccc(F)cc2)n1	7	AKT2	AKT2	>	10000
UNC10224998A	CCCc1ccc(cc1)S(=O)(=O)NC1CCN(C1)c1ccnc(Nc2ccc(F)cc2)n1	7	CDC2L5	CDK13	>	10000
UNC10224998A	CCCc1ccc(cc1)S(=O)(=O)NC1CCN(C1)c1ccnc(Nc2ccc(F)cc2)n1	7	GRK1	GRK1	>	10000

UNC10224998A	CCCc1ccc(cc1)S(=O)(=O)NC1CCN(C1)c1ccnc(Nc2ccc(F)cc2)n1	7	MEK6	MAP2K6	>	10000
UNC10224998A	CCCc1ccc(cc1)S(=O)(=O)NC1CCN(C1)c1ccnc(Nc2ccc(F)cc2)n1	7	NEK1	NEK1	>	10000
UNC10224998A	CCCc1ccc(cc1)S(=O)(=O)NC1CCN(C1)c1ccnc(Nc2ccc(F)cc2)n1	7	PDPK1	PDPK1	>	10000
UNC10224998A	CCCc1ccc(cc1)S(=O)(=O)NC1CCN(C1)c1ccnc(Nc2ccc(F)cc2)n1	7	PKAC-alpha	PRKACA	>	10000
UNC10224998A	CCCc1ccc(cc1)S(=O)(=O)NC1CCN(C1)c1ccnc(Nc2ccc(F)cc2)n1	7	TSSK1B	TSSK1B	>	10000
UNC10224998A	CCCc1ccc(cc1)S(=O)(=O)NC1CCN(C1)c1ccnc(Nc2ccc(F)cc2)n1	7	YANK3	STK32C	>	10000
UNC10224998A	CCCc1ccc(cc1)S(=O)(=O)NC1CCN(C1)c1ccnc(Nc2ccc(F)cc2)n1	7	YSK1	STK25	>	10000
UNC10225000A	Nc1ncnc2occ(-c3ccc4N(CCc4c3)C(=O)Cc3cc(F)ccc3F)c12	2	CDK11	CDK19	=	1100
UNC10225000A	Nc1ncnc2occ(-c3ccc4N(CCc4c3)C(=O)Cc3cc(F)ccc3F)c12	2	EPHB6	EPHB6	=	500
UNC10225000A	Nc1ncnc2occ(-c3ccc4N(CCc4c3)C(=O)Cc3cc(F)ccc3F)c12	2	MEK5	MAP2K5	=	750
UNC10225000A	Nc1ncnc2occ(-c3ccc4N(CCc4c3)C(=O)Cc3cc(F)ccc3F)c12	2	RIPK1	RIPK1	=	410
UNC10225002A	Fc1cccc(c1)-c1ccc2c(NC(=O)C3CC3)n[nH]c2c1	1	NLK	NLK	=	87
UNC10225002A	Fc1cccc(c1)-c1ccc2c(NC(=O)C3CC3)n[nH]c2c1	1	YSK4	MAP3K19	=	310
UNC10225004A	COc1ccccc1-c1ccc2c(NC(=O)C3CC3)n[nH]c2n1	2	ABL1-phosp	ABL1	>	10000
UNC10225004A	COc1ccccc1-c1ccc2c(NC(=O)C3CC3)n[nH]c2n1	2	ULK3	ULK3	>	10000
UNC10225005A	CC1CN(CCO1)c1cc(=O)n2nc(C)n(Cc3cccc(c3C)C(F)(F)F)c2n1	2	PIK3CB	PIK3CB	=	0.67
UNC10225005A	CC1CN(CCO1)c1cc(=O)n2nc(C)n(Cc3cccc(c3C)C(F)(F)F)c2n1	2	PIK3CD	PIK3CD	=	80
UNC10225006A	OC(=O)C(F)(F)F.OC(=O)C(F)(F)F.Nc1n[nH]c2cc(ccc12)-c1cc(nc(N)n1)N1CCC[C@H](C1)C(=O)Nc1ccccc1	5	CSNK1G2	CSNK1G2	>	10000
UNC10225006A	OC(=O)C(F)(F)F.OC(=O)C(F)(F)F.Nc1n[nH]c2cc(ccc12)-c1cc(nc(N)n1)N1CCC[C@H](C1)C(=O)Nc1ccccc1	5	ERK1	MAPK3	=	5300
UNC10225006A	OC(=O)C(F)(F)F.OC(=O)C(F)(F)F.Nc1n[nH]c2cc(ccc12)-c1cc(nc(N)n1)N1CCC[C@H](C1)C(=O)Nc1ccccc1	5	IGF1R	IGF1R	>	10000
UNC10225006A	OC(=O)C(F)(F)F.OC(=O)C(F)(F)F.Nc1n[nH]c2cc(ccc12)-c1cc(nc(N)n1)N1CCC[C@H](C1)C(=O)Nc1ccccc1	5	JAK2(JH1do	JAK2	>	10000
UNC10225006A	OC(=O)C(F)(F)F.OC(=O)C(F)(F)F.Nc1n[nH]c2cc(ccc12)-c1cc(nc(N)n1)N1CCC[C@H](C1)C(=O)Nc1ccccc1	5	PDPK1	PDPK1	=	25
UNC10225006A	OC(=O)C(F)(F)F.OC(=O)C(F)(F)F.Nc1n[nH]c2cc(ccc12)-c1cc(nc(N)n1)N1CCC[C@H](C1)C(=O)Nc1ccccc1	5	RIOK3	RIOK3	=	570
UNC10225008A	Cc1cccc(n1)-c1cc(Nc2ccncc2)c2ccccc2n1	6	ACVR1B	ACVR1B	=	31
UNC10225008A	Cc1cccc(n1)-c1cc(Nc2ccncc2)c2ccccc2n1	6	JAK2(JH1do	JAK2	>	10000
UNC10225008A	Cc1cccc(n1)-c1cc(Nc2ccncc2)c2ccccc2n1	6	PRKD1	PRKD1	=	100
UNC10225008A	Cc1cccc(n1)-c1cc(Nc2ccncc2)c2ccccc2n1	6	PRKD2	PRKD2	=	300
UNC10225008A	Cc1cccc(n1)-c1cc(Nc2ccncc2)c2ccccc2n1	6	PRKD3	PRKD3	=	210
UNC10225008A	Cc1cccc(n1)-c1cc(Nc2ccncc2)c2ccccc2n1	6	RPS6KA5(Ki	RPS6KA5	>	10000
UNC10225008A	Cc1cccc(n1)-c1cc(Nc2ccncc2)c2ccccc2n1	6	TGFBR1	TGFBR1	=	20
UNC10225008A	Cc1cccc(n1)-c1cc(Nc2ccncc2)c2ccccc2n1	6	TXK	TXK	>	10000
UNC10225008A	Cc1cccc(n1)-c1cc(Nc2ccncc2)c2ccccc2n1	6	ZAK	ZAK	>	10000
UNC10225009A	CN1CCN(CC1)c1cc(ccn1)C(=O)Nc1ccc(C)c(c1)-c1ccc(cc1)C(=O)NCC1CC1	3	PDGFRB	PDGFRB	=	300
UNC10225009A	CN1CCN(CC1)c1cc(ccn1)C(=O)Nc1ccc(C)c(c1)-c1ccc(cc1)C(=O)NCC1CC1	3	TYK2(JH1do	TYK2	>	10000
UNC10225009A	CN1CCN(CC1)c1cc(ccn1)C(=O)Nc1ccc(C)c(c1)-c1ccc(cc1)C(=O)NCC1CC1	3	ULK2	ULK2	>	10000
UNC10225010A	COc1ccc(Nc2nccc(n2)N2CCC(C2)NC(=O)C(C)c2ccc(Cl)cc2)cc1	1	CDKL1	CDKL1	>	10000

UNC10225010A	COc1ccc(Nc2nccc(n2)N2CCC(C2)NC(=O)C(C)c2ccc(Cl)cc2)cc1	1	PRKG2	PRKG2	>	10000
UNC10225013A	Cl.COc1cc2nccc(Nc3ccc(NC(=O)c4ccc(C)cc4)c(O)c3)c2cc1OC	5	BLK	BLK	=	230
UNC10225013A	Cl.COc1cc2nccc(Nc3ccc(NC(=O)c4ccc(C)cc4)c(O)c3)c2cc1OC	5	CLK3	CLK3	>	10000
UNC10225013A	Cl.COc1cc2nccc(Nc3ccc(NC(=O)c4ccc(C)cc4)c(O)c3)c2cc1OC	5	LCK	LCK	=	560
UNC10225013A	Cl.COc1cc2nccc(Nc3ccc(NC(=O)c4ccc(C)cc4)c(O)c3)c2cc1OC	5	MEK5	MAP2K5	=	8.7
UNC10225013A	Cl.COc1cc2nccc(Nc3ccc(NC(=O)c4ccc(C)cc4)c(O)c3)c2cc1OC	5	PFTK1	CDK14	>	10000
UNC10225013A	Cl.COc1cc2nccc(Nc3ccc(NC(=O)c4ccc(C)cc4)c(O)c3)c2cc1OC	5	PIK3C2B	PIK3C2B	>	10000
UNC10225013A	Cl.COc1cc2nccc(Nc3ccc(NC(=O)c4ccc(C)cc4)c(O)c3)c2cc1OC	5	PIK3CA	PIK3CA	>	10000
UNC10225013A	Cl.COc1cc2nccc(Nc3ccc(NC(=O)c4ccc(C)cc4)c(O)c3)c2cc1OC	5	PIK3CG	PIK3CG	>	10000
UNC10225014A	Clc1ccc(cc1)C1=C(Nc2ccccc2)C(=O)NC1=O	1	ZAP70	ZAP70	>	10000
UNC10225018A	CNc1nc(cc(n1)-c1ccc2c(N)n[nH]c2c1)N1C[C@H](CC[C@H]1C)C(=O)Nc1ccccc1	3	CAMK2A	CAMK2A	>	10000
UNC10225018A	CNc1nc(cc(n1)-c1ccc2c(N)n[nH]c2c1)N1C[C@H](CC[C@H]1C)C(=O)Nc1ccccc1	3	CAMK2B	CAMK2B	>	10000
UNC10225018A	CNc1nc(cc(n1)-c1ccc2c(N)n[nH]c2c1)N1C[C@H](CC[C@H]1C)C(=O)Nc1ccccc1	3	CIT	CIT	=	500
UNC10225018A	CNc1nc(cc(n1)-c1ccc2c(N)n[nH]c2c1)N1C[C@H](CC[C@H]1C)C(=O)Nc1ccccc1	3	DRAK1	STK17A	=	89
UNC10225018A	CNc1nc(cc(n1)-c1ccc2c(N)n[nH]c2c1)N1C[C@H](CC[C@H]1C)C(=O)Nc1ccccc1	3	DRAK2	STK17B	=	83
UNC10225018A	CNc1nc(cc(n1)-c1ccc2c(N)n[nH]c2c1)N1C[C@H](CC[C@H]1C)C(=O)Nc1ccccc1	3	GRK1	GRK1	>	10000
UNC10225018A	CNc1nc(cc(n1)-c1ccc2c(N)n[nH]c2c1)N1C[C@H](CC[C@H]1C)C(=O)Nc1ccccc1	3	PDPK1	PDPK1	=	1.7
UNC10225018A	CNc1nc(cc(n1)-c1ccc2c(N)n[nH]c2c1)N1C[C@H](CC[C@H]1C)C(=O)Nc1ccccc1	3	PRKX	PRKX	=	65
UNC10225018A	CNc1nc(cc(n1)-c1ccc2c(N)n[nH]c2c1)N1C[C@H](CC[C@H]1C)C(=O)Nc1ccccc1	3	YANK2	STK32B	=	220
UNC10225019A	Cc1ccc(NC(=O)Nc2cccc(Cl)c2)cc1-c1ccc(cc1)C(=O)Nc1ccncc1	12	BRSK1	BRSK1	>	10000
UNC10225019A	Cc1ccc(NC(=O)Nc2cccc(Cl)c2)cc1-c1ccc(cc1)C(=O)Nc1ccncc1	12	CIT	CIT	=	150
UNC10225019A	Cc1ccc(NC(=O)Nc2cccc(Cl)c2)cc1-c1ccc(cc1)C(=O)Nc1ccncc1	12	DDR1	DDR1	=	76
UNC10225019A	Cc1ccc(NC(=O)Nc2cccc(Cl)c2)cc1-c1ccc(cc1)C(=O)Nc1ccncc1	12	IRAK3	IRAK3	>	10000

UNC10225019A	Cc1ccc(NC(=O)Nc2cccc(Cl)c2)cc1-c1ccc(cc1)C(=O)Nc1ccncc1	12	KIT	KIT	=	180
UNC10225019A	Cc1ccc(NC(=O)Nc2cccc(Cl)c2)cc1-c1ccc(cc1)C(=O)Nc1ccncc1	12	MEK1	MAP2K1	>	10000
UNC10225019A	Cc1ccc(NC(=O)Nc2cccc(Cl)c2)cc1-c1ccc(cc1)C(=O)Nc1ccncc1	12	MYO3A	MYO3A	>	10000
UNC10225019A	Cc1ccc(NC(=O)Nc2cccc(Cl)c2)cc1-c1ccc(cc1)C(=O)Nc1ccncc1	12	PDGFRB	PDGFRB	=	50
UNC10225019A	Cc1ccc(NC(=O)Nc2cccc(Cl)c2)cc1-c1ccc(cc1)C(=O)Nc1ccncc1	12	PRKX	PRKX	=	7400
UNC10225019A	Cc1ccc(NC(=O)Nc2cccc(Cl)c2)cc1-c1ccc(cc1)C(=O)Nc1ccncc1	12	RAF1	RAF1	=	180
UNC10225019A	Cc1ccc(NC(=O)Nc2cccc(Cl)c2)cc1-c1ccc(cc1)C(=O)Nc1ccncc1	12	YSK4	MAP3K19	=	61
UNC10225024A	FC(F)(F)c1cccc(Cn2ccn3c2nc(cc3=O)N2CCOCC2)c1	2	PIK3CB	PIK3CB	=	140
UNC10225024A	FC(F)(F)c1cccc(Cn2ccn3c2nc(cc3=O)N2CCOCC2)c1	2	VPS34	PIK3C3	=	130
UNC10225028A	Cc1ccc(s1)-c1nc(Nc2ccncc2)c2ccccc2n1	3	CDK5	CDK5	>	10000
UNC10225028A	Cc1ccc(s1)-c1nc(Nc2ccncc2)c2ccccc2n1	3	EPHA5	EPHA5	>	10000
UNC10225028A	Cc1ccc(s1)-c1nc(Nc2ccncc2)c2ccccc2n1	3	ERK2	MAPK1	>	10000
UNC10225030A	CC(C)c1cc(nc(N)n1)-c1ccc2c(N)n[nH]c2c1	1	CHEK2	CHEK2	=	320
UNC10225030A	CC(C)c1cc(nc(N)n1)-c1ccc2c(N)n[nH]c2c1	1	DRAK1	STK17A	=	29
UNC10225030A	CC(C)c1cc(nc(N)n1)-c1ccc2c(N)n[nH]c2c1	1	HASPIN	GSG2	=	200
UNC10225030A	CC(C)c1cc(nc(N)n1)-c1ccc2c(N)n[nH]c2c1	1	MKNK2	MKNK2	=	160
UNC10225030A	CC(C)c1cc(nc(N)n1)-c1ccc2c(N)n[nH]c2c1	1	RIOK1	RIOK1	=	580
UNC10225030A	CC(C)c1cc(nc(N)n1)-c1ccc2c(N)n[nH]c2c1	1	RIOK3	RIOK3	=	440
UNC10225031A	CNc1nccc(n1)-c1c(ncn1CCCN1CCOCC1)-c1ccc(F)cc1	3	DMPK2	CDC42BPG	>	10000
UNC10225031A	CNc1nccc(n1)-c1c(ncn1CCCN1CCOCC1)-c1ccc(F)cc1	3	LIMK2	LIMK2	>	10000
UNC10225031A	CNc1nccc(n1)-c1c(ncn1CCCN1CCOCC1)-c1ccc(F)cc1	3	NEK5	NEK5	>	10000
UNC10225031A	CNc1nccc(n1)-c1c(ncn1CCCN1CCOCC1)-c1ccc(F)cc1	3	PAK2	PAK2	>	10000
UNC10225031A	CNc1nccc(n1)-c1c(ncn1CCCN1CCOCC1)-c1ccc(F)cc1	3	PIM3	PIM3	>	10000
UNC10225031A	CNc1nccc(n1)-c1c(ncn1CCCN1CCOCC1)-c1ccc(F)cc1	3	RSK4(Kin.Dc	RPS6KA6	>	10000
UNC10225035A	Cn1cncc1-c1cc2c(Nc3ccc(OCc4ccccc4)cc3)ncnc2cn1	12	CDKL1	CDKL1	>	10000
UNC10225035A	Cn1cncc1-c1cc2c(Nc3ccc(OCc4ccccc4)cc3)ncnc2cn1	12	DRAK1	STK17A	>	10000
UNC10225035A	Cn1cncc1-c1cc2c(Nc3ccc(OCc4ccccc4)cc3)ncnc2cn1	12	EGFR	EGFR	=	10
UNC10225035A	Cn1cncc1-c1cc2c(Nc3ccc(OCc4ccccc4)cc3)ncnc2cn1	12	ERBB2	ERBB2	=	17
UNC10225035A	Cn1cncc1-c1cc2c(Nc3ccc(OCc4ccccc4)cc3)ncnc2cn1	12	MEK5	MAP2K5	=	140
UNC10225035A	Cn1cncc1-c1cc2c(Nc3ccc(OCc4ccccc4)cc3)ncnc2cn1	12	PRKG2	PRKG2	=	3300
UNC10225035A	Cn1cncc1-c1cc2c(Nc3ccc(OCc4ccccc4)cc3)ncnc2cn1	12	RIPK2	RIPK2	=	65
UNC10225035A	Cn1cncc1-c1cc2c(Nc3ccc(OCc4ccccc4)cc3)ncnc2cn1	12	ROCK1	ROCK1	=	590
UNC10225035A	Cn1cncc1-c1cc2c(Nc3ccc(OCc4ccccc4)cc3)ncnc2cn1	12	TYK2(JH1do	TYK2	>	10000
UNC10225037A	C1COc2cc(Nc3nccc(n3)-c3cnn4nc(ccc34)-c3ccccc3)ccc2O1	4	CSK	CSK	>	10000
UNC10225037A	C1COc2cc(Nc3nccc(n3)-c3cnn4nc(ccc34)-c3ccccc3)ccc2O1	4	ERK8	MAPK15	=	670
UNC10225037A	C1COc2cc(Nc3nccc(n3)-c3cnn4nc(ccc34)-c3ccccc3)ccc2O1	4	MAK	MAK	>	10000
UNC10225037A	C1COc2cc(Nc3nccc(n3)-c3cnn4nc(ccc34)-c3ccccc3)ccc2O1	4	MET	MET	>	10000
UNC10225037A	C1COc2cc(Nc3nccc(n3)-c3cnn4nc(ccc34)-c3ccccc3)ccc2O1	4	PAK6	PAK6	>	10000
UNC10225037A	C1COc2cc(Nc3nccc(n3)-c3cnn4nc(ccc34)-c3ccccc3)ccc2O1	4	STK16	STK16	=	620
UNC10225042A	COc1ccc2nc(nc(Nc3ccncc3)c2c1)-c1cccc(C)n1	6	ACVR1B	ACVR1B	=	150
UNC10225042A	COc1ccc2nc(nc(Nc3ccncc3)c2c1)-c1cccc(C)n1	6	DMPK2	CDC42BPG	=	510
UNC10225042A	COc1ccc2nc(nc(Nc3ccncc3)c2c1)-c1cccc(C)n1	6	JAK1(JH2do	JAK1	=	260

UNC10225042A	COc1ccc2nc(nc(Nc3ccncc3)c2c1)-c1cccc(C)n1	6	MRCKB	CDC42BPB	=	520
UNC10225042A	COc1ccc2nc(nc(Nc3ccncc3)c2c1)-c1cccc(C)n1	6	PIKFYVE	PIKFYVE	=	1200
UNC10225042A	COc1ccc2nc(nc(Nc3ccncc3)c2c1)-c1cccc(C)n1	6	PRKD1	PRKD1	=	51
UNC10225042A	COc1ccc2nc(nc(Nc3ccncc3)c2c1)-c1cccc(C)n1	6	PRKD2	PRKD2	=	34
UNC10225042A	COc1ccc2nc(nc(Nc3ccncc3)c2c1)-c1cccc(C)n1	6	PRKD3	PRKD3	=	490
UNC10225042A	COc1ccc2nc(nc(Nc3ccncc3)c2c1)-c1cccc(C)n1	6	TGFBR1	TGFBR1	=	170
UNC10225042A	COc1ccc2nc(nc(Nc3ccncc3)c2c1)-c1cccc(C)n1	6	TYK2(JH2do	TYK2	=	2000
UNC10225044A	Cc1cccc(n1)-c1nc(Nc2cccc(c2)C(N)=O)c2ccccc2n1	1	ACVR1B	ACVR1B	=	1600
UNC10225044A	Cc1cccc(n1)-c1nc(Nc2cccc(c2)C(N)=O)c2ccccc2n1	1	MEK1	MAP2K1	>	10000
UNC10225044A	Cc1cccc(n1)-c1nc(Nc2cccc(c2)C(N)=O)c2ccccc2n1	1	PRKD3	PRKD3	=	820
UNC10225050A	Brc1cc2c(NC(=O)C3CCCC3)n[nH]c2nc1-c1nccs1	5	BRSK1	BRSK1	=	430
UNC10225050A	Brc1cc2c(NC(=O)C3CCCC3)n[nH]c2nc1-c1nccs1	5	BRSK2	BRSK2	=	330
UNC10225050A	Brc1cc2c(NC(=O)C3CCCC3)n[nH]c2nc1-c1nccs1	5	CDC2L1	CDK11B	>	10000
UNC10225050A	Brc1cc2c(NC(=O)C3CCCC3)n[nH]c2nc1-c1nccs1	5	CDK2	CDK2	=	280
UNC10225050A	Brc1cc2c(NC(=O)C3CCCC3)n[nH]c2nc1-c1nccs1	5	CDK4	CDK4	=	4200
UNC10225050A	Brc1cc2c(NC(=O)C3CCCC3)n[nH]c2nc1-c1nccs1	5	CDK9	CDK9	=	2500
UNC10225050A	Brc1cc2c(NC(=O)C3CCCC3)n[nH]c2nc1-c1nccs1	5	CDKL2	CDKL2	=	500
UNC10225050A	Brc1cc2c(NC(=O)C3CCCC3)n[nH]c2nc1-c1nccs1	5	ERK4	MAPK4	>	10000
UNC10225050A	Brc1cc2c(NC(=O)C3CCCC3)n[nH]c2nc1-c1nccs1	5	GSK3A	GSK3A	=	61
UNC10225050A	Brc1cc2c(NC(=O)C3CCCC3)n[nH]c2nc1-c1nccs1	5	ICK	ICK	=	220
UNC10225050A	Brc1cc2c(NC(=O)C3CCCC3)n[nH]c2nc1-c1nccs1	5	MYLK2	MYLK2	=	130
UNC10225052A	COc1cc(Nc2nccc(n2)N2CCC(C2)NC(=O)C2COc3ccccc3O2)c c(OC)c1OC	5	EPHB6	EPHB6	=	110
UNC10225052A	COc1cc(Nc2nccc(n2)N2CCC(C2)NC(=O)C2COc3ccccc3O2)c c(OC)c1OC	5	JAK2(JH1do	JAK2	=	47
UNC10225052A	COc1cc(Nc2nccc(n2)N2CCC(C2)NC(=O)C2COc3ccccc3O2)c c(OC)c1OC	5	MEK5	MAP2K5	=	180
UNC10225052A	COc1cc(Nc2nccc(n2)N2CCC(C2)NC(=O)C2COc3ccccc3O2)c c(OC)c1OC	5	NEK5	NEK5	=	370
UNC10225052A	COc1cc(Nc2nccc(n2)N2CCC(C2)NC(=O)C2COc3ccccc3O2)c c(OC)c1OC	5	PIP5K2C	PIP4K2C	=	670
UNC10225052A	COc1cc(Nc2nccc(n2)N2CCC(C2)NC(=O)C2COc3ccccc3O2)c c(OC)c1OC	5	TYK2(JH1do	TYK2	=	1500
UNC10225056A	COc1ccc(Nc2nccc(n2)- c2cccc(NC(=O)c3cc4cc(F)ccc4[nH]3)c2)cc1	5	ABL1-nonpl	ABL1	=	100
UNC10225056A	COc1ccc(Nc2nccc(n2)- c2cccc(NC(=O)c3cc4cc(F)ccc4[nH]3)c2)cc1	5	MEK5	MAP2K5	=	200
UNC10225056A	COc1ccc(Nc2nccc(n2)- c2cccc(NC(=O)c3cc4cc(F)ccc4[nH]3)c2)cc1	5	PIP5K2C	PIP4K2C	=	1400
UNC10225056A	COc1ccc(Nc2nccc(n2)- c2cccc(NC(=O)c3cc4cc(F)ccc4[nH]3)c2)cc1	5	TNIK	TNIK	=	820
UNC10225057A	Clc1ccc(cc1)-c1cc(ccn1)-c1c[nH]nc1-c1ccccn1	2	DLK	MAP3K12	>	10000
UNC10225057A	Clc1ccc(cc1)-c1cc(ccn1)-c1c[nH]nc1-c1ccccn1	2	TGFBR2	TGFBR2	=	530
UNC10225057A	Clc1ccc(cc1)-c1cc(ccn1)-c1c[nH]nc1-c1ccccn1	2	TNIK	TNIK	=	390
UNC10225057A	Clc1ccc(cc1)-c1cc(ccn1)-c1c[nH]nc1-c1ccccn1	2	TRKC	NTRK3	>	10000
UNC10225058A	Nc1nccc(n1)-c1c(ncn1C1CCN(Cc2ccccc2)CC1)-c1ccc(F)cc1	9	CIT	CIT	=	130
UNC10225058A	Nc1nccc(n1)-c1c(ncn1C1CCN(Cc2ccccc2)CC1)-c1ccc(F)cc1	9	CSNK1A1	CSNK1A1	=	41
UNC10225058A	Nc1nccc(n1)-c1c(ncn1C1CCN(Cc2ccccc2)CC1)-c1ccc(F)cc1	9	CSNK1D	CSNK1D	=	7.5
UNC10225058A	Nc1nccc(n1)-c1c(ncn1C1CCN(Cc2ccccc2)CC1)-c1ccc(F)cc1	9	CSNK1E	CSNK1E	=	24
UNC10225058A	Nc1nccc(n1)-c1c(ncn1C1CCN(Cc2ccccc2)CC1)-c1ccc(F)cc1	9	DDR1	DDR1	=	140

UNC10225058A	Nc1nccc(n1)-c1c(ncn1C1CCN(Cc2ccccc2)CC1)-c1ccc(F)cc1	9	JNK2	MAPK9	=	43
UNC10225058A	Nc1nccc(n1)-c1c(ncn1C1CCN(Cc2ccccc2)CC1)-c1ccc(F)cc1	9	JNK3	MAPK10	=	31
UNC10225058A	Nc1nccc(n1)-c1c(ncn1C1CCN(Cc2ccccc2)CC1)-c1ccc(F)cc1	9	NLK	NLK	=	140
UNC10225058A	Nc1nccc(n1)-c1c(ncn1C1CCN(Cc2ccccc2)CC1)-c1ccc(F)cc1	9	p38-alpha	MAPK14	=	46
UNC10225058A	Nc1nccc(n1)-c1c(ncn1C1CCN(Cc2ccccc2)CC1)-c1ccc(F)cc1	9	PIP5K2C	PIP4K2C	=	93
UNC10225058A	Nc1nccc(n1)-c1c(ncn1C1CCN(Cc2ccccc2)CC1)-c1ccc(F)cc1	9	TNIK	TNIK	=	4100
UNC10225060A	CC(=O)Nc1n[nH]c2ncc(cc12)-c1ccccc1	4	DAPK1	DAPK1	>	10000
UNC10225060A	CC(=O)Nc1n[nH]c2ncc(cc12)-c1ccccc1	4	JAK1(JH2do	JAK1	=	380
UNC10225060A	CC(=O)Nc1n[nH]c2ncc(cc12)-c1ccccc1	4	MEK5	MAP2K5	=	290
UNC10225060A	CC(=O)Nc1n[nH]c2ncc(cc12)-c1ccccc1	4	NIM1	MGC42105	>	10000
UNC10225060A	CC(=O)Nc1n[nH]c2ncc(cc12)-c1ccccc1	4	PDGFRB	PDGFRB	=	270
UNC10225060A	CC(=O)Nc1n[nH]c2ncc(cc12)-c1ccccc1	4	RSK1(Kin.Dc	RPS6KA1	>	10000
UNC10225060A	CC(=O)Nc1n[nH]c2ncc(cc12)-c1ccccc1	4	TRKA	NTRK1	=	2500
UNC10225060A	CC(=O)Nc1n[nH]c2ncc(cc12)-c1ccccc1	4	TYK2(JH2do	TYK2	=	580
UNC10225061A	COc1cccc(c1)C1=C(Nc2cccc(c2)C(O)=O)C(=O)NC1=O	2	JAK3(JH1do	JAK3	=	480
UNC10225061A	COc1cccc(c1)C1=C(Nc2cccc(c2)C(O)=O)C(=O)NC1=O	2	PRKCI	PRKCI	>	10000
UNC10225063A	OC(=O)c1cc(NC2=C(C(=O)NC2=O)c2ccccc2)ccc1Cl	0	NEK10	NEK10	=	460
UNC10225065A	Oc1cccc(NC2=C(C(=O)NC2=O)c2ccccc2)c1	3	NIM1	MGC42105	>	10000
UNC10225065A	Oc1cccc(NC2=C(C(=O)NC2=O)c2ccccc2)c1	3	PRKR	EIF2AK2	>	10000
UNC10225065A	Oc1cccc(NC2=C(C(=O)NC2=O)c2ccccc2)c1	3	TLK2	TLK2	=	2400
UNC10225069A	COc1cc2ncn(-c3cc(OCc4ccncc4)c(s3)C(N)=O)c2cc1OC	4	INSRR	INSRR	>	10000
UNC10225069A	COc1cc2ncn(-c3cc(OCc4ccncc4)c(s3)C(N)=O)c2cc1OC	4	MEK5	MAP2K5	=	670
UNC10225069A	COc1cc2ncn(-c3cc(OCc4ccncc4)c(s3)C(N)=O)c2cc1OC	4	MUSK	MUSK	>	10000
UNC10225069A	COc1cc2ncn(-c3cc(OCc4ccncc4)c(s3)C(N)=O)c2cc1OC	4	PLK1	PLK1	=	110
UNC10225069A	COc1cc2ncn(-c3cc(OCc4ccncc4)c(s3)C(N)=O)c2cc1OC	4	PLK2	PLK2	=	41
UNC10225069A	COc1cc2ncn(-c3cc(OCc4ccncc4)c(s3)C(N)=O)c2cc1OC	4	TIE1	TIE1	>	10000
UNC10225070A	Nc1n[nH]c2cc(ccc12)-c1cccc(N)n1	2	CDK7	CDK7	>	10000
UNC10225070A	Nc1n[nH]c2cc(ccc12)-c1cccc(N)n1	2	JAK2(JH1do	JAK2	>	10000
UNC10225070A	Nc1n[nH]c2cc(ccc12)-c1cccc(N)n1	2	MYLK2	MYLK2	=	180
UNC10225070A	Nc1n[nH]c2cc(ccc12)-c1cccc(N)n1	2	RIOK1	RIOK1	=	390
UNC10225070A	Nc1n[nH]c2cc(ccc12)-c1cccc(N)n1	2	RIOK3	RIOK3	=	490
UNC10225070A	Nc1n[nH]c2cc(ccc12)-c1cccc(N)n1	2	YSK4	MAP3K19	=	130
UNC10225072A	N#Cc1ccc(cc1)-c1cc(ccn1)-c1c[nH]nc1-c1ccccn1	0	TGFBR1	TGFBR1	=	150
UNC10225072A	N#Cc1ccc(cc1)-c1cc(ccn1)-c1c[nH]nc1-c1ccccn1	0	TGFBR2	TGFBR2	=	580
UNC10225073A	Fc1ccc(Nc2nccc(n2)-c2c(nn3ncccc23)-c2ccc(Cl)cc2)cc1F	1	DDR1	DDR1	=	750
UNC10225073A	Fc1ccc(Nc2nccc(n2)-c2c(nn3ncccc23)-c2ccc(Cl)cc2)cc1F	1	TNIK	TNIK	>	10000
UNC10225073A	Fc1ccc(Nc2nccc(n2)-c2c(nn3ncccc23)-c2ccc(Cl)cc2)cc1F	1	ULK2	ULK2	>	10000
UNC10225075A	CN(C)c1cc(nc(N)n1)-c1ccc2c(N)n[nH]c2c1	2	KIT-autoinh	KIT	>	10000
UNC10225075A	CN(C)c1cc(nc(N)n1)-c1ccc2c(N)n[nH]c2c1	2	TXK	TXK	>	10000
UNC10225075A	CN(C)c1cc(nc(N)n1)-c1ccc2c(N)n[nH]c2c1	2	ZAK	ZAK	>	10000
UNC10225080A	COc1cc(Nc2nccc(n2)N2CCC(C2)NC(=O)C(C)c2ccc(Cl)cc2)cc(OC)c1OC	2	EPHB6	EPHB6	=	100
UNC10225080A	COc1cc(Nc2nccc(n2)N2CCC(C2)NC(=O)C(C)c2ccc(Cl)cc2)cc(OC)c1OC	2	JAK2(JH1do	JAK2	=	140
UNC10225080A	COc1cc(Nc2nccc(n2)N2CCC(C2)NC(=O)C(C)c2ccc(Cl)cc2)cc(OC)c1OC	2	NEK5	NEK5	=	740
UNC10225083A	C[C@@H]1CCCCN1c1cc(nc(N)n1)-c1ccc2c(N)n[nH]c2c1	1	AURKA	AURKA	>	10000
UNC10225083A	C[C@@H]1CCCCN1c1cc(nc(N)n1)-c1ccc2c(N)n[nH]c2c1	1	DRAK1	STK17A	=	88
UNC10225083A	C[C@@H]1CCCCN1c1cc(nc(N)n1)-c1ccc2c(N)n[nH]c2c1	1	p38-alpha	MAPK14	=	2100

UNC10225085A	Cl.C(c1ccccc1)n1ncc2cc(Nc3ncnc4ccccc34)ccc12	9	EGFR	EGFR	=	7.6
UNC10225085A	Cl.C(c1ccccc1)n1ncc2cc(Nc3ncnc4ccccc34)ccc12	9	ERBB2	ERBB2	=	11
UNC10225085A	Cl.C(c1ccccc1)n1ncc2cc(Nc3ncnc4ccccc34)ccc12	9	ERBB4	ERBB4	=	61
UNC10225085A	Cl.C(c1ccccc1)n1ncc2cc(Nc3ncnc4ccccc34)ccc12	9	MEK5	MAP2K5	=	90
UNC10225086A	Fc1ccc(Nc2nccc(NCCNC(=O)c3cc4ccccc4s3)n2)cc1	3	TESK1	TESK1	>	10000
UNC10225086A	Fc1ccc(Nc2nccc(NCCNC(=O)c3cc4ccccc4s3)n2)cc1	3	TXK	TXK	>	10000
UNC10225086A	Fc1ccc(Nc2nccc(NCCNC(=O)c3cc4ccccc4s3)n2)cc1	3	ZAK	ZAK	>	10000
UNC10225087A	CN(C1=C(C(=O)NC1=O)c1ccccc1)c1ccccc1	0	TXK	TXK	>	10000
UNC10225088A	CC(=O)c1cccc(NC(=O)NC2CCN(C2)c2ccnc(Nc3ccc(F)cc3)n2)c1	0	CDK11	CDK19	=	2000
UNC10225088A	CC(=O)c1cccc(NC(=O)NC2CCN(C2)c2ccnc(Nc3ccc(F)cc3)n2)c1	0	TRKC	NTRK3	=	94
UNC10225090A	Oc1ccc(NC2=C(C(=O)NC2=O)c2ccc(Cl)cc2)cc1	3	CAMK1	CAMK1	>	10000
UNC10225090A	Oc1ccc(NC2=C(C(=O)NC2=O)c2ccc(Cl)cc2)cc1	3	CAMK1D	CAMK1D	>	10000
UNC10225090A	Oc1ccc(NC2=C(C(=O)NC2=O)c2ccc(Cl)cc2)cc1	3	TTK	TTK	=	5500
UNC10225091A	Cl.CS(=O)(=O)CCNCc1nc(cs1)-c1ccc2ncnc(Nc3ccc(OCc4cccc(F)c4)c(Cl)c3)c2c1	14	EGFR	EGFR	=	0.69
UNC10225091A	Cl.CS(=O)(=O)CCNCc1nc(cs1)-c1ccc2ncnc(Nc3ccc(OCc4cccc(F)c4)c(Cl)c3)c2c1	14	ERBB2	ERBB2	=	5.9
UNC10225091A	Cl.CS(=O)(=O)CCNCc1nc(cs1)-c1ccc2ncnc(Nc3ccc(OCc4cccc(F)c4)c(Cl)c3)c2c1	14	ERBB4	ERBB4	=	26
UNC10225091A	Cl.CS(=O)(=O)CCNCc1nc(cs1)-c1ccc2ncnc(Nc3ccc(OCc4cccc(F)c4)c(Cl)c3)c2c1	14	TESK1	TESK1	>	10000
UNC10225091A	Cl.CS(=O)(=O)CCNCc1nc(cs1)-c1ccc2ncnc(Nc3ccc(OCc4cccc(F)c4)c(Cl)c3)c2c1	14	TXK	TXK	>	10000
UNC10225091A	Cl.CS(=O)(=O)CCNCc1nc(cs1)-c1ccc2ncnc(Nc3ccc(OCc4cccc(F)c4)c(Cl)c3)c2c1	14	ZAK	ZAK	>	10000
UNC10225093A	COc1ccc(Nc2nccc(n2)N2CCC(C2)NC(=O)c2cc3ccccc3s2)cc 1	3	CHEK1	CHEK1	>	10000
UNC10225093A	COc1ccc(Nc2nccc(n2)N2CCC(C2)NC(=O)c2cc3ccccc3s2)cc 1	3	DDR1	DDR1	>	10000
UNC10225093A	COc1ccc(Nc2nccc(n2)N2CCC(C2)NC(=O)c2cc3ccccc3s2)cc 1	3	JAK2(JH1do	JAK2	=	1800
UNC10225093A	COc1ccc(Nc2nccc(n2)N2CCC(C2)NC(=O)c2cc3ccccc3s2)cc 1	3	MAK	MAK	>	10000
UNC10225093A	COc1ccc(Nc2nccc(n2)N2CCC(C2)NC(=O)c2cc3ccccc3s2)cc 1	3	PRKD3	PRKD3	>	10000
UNC10225095A	CCn1c(nc2cnccc12)-c1c(C)onc1N	7	CDC2L5	CDK13	>	10000
UNC10225095A	CCn1c(nc2cnccc12)-c1c(C)onc1N	7	DMPK2	CDC42BPG	>	10000
UNC10225095A	CCn1c(nc2cnccc12)-c1c(C)onc1N	7	ERK3	MAPK6	>	10000
UNC10225095A	CCn1c(nc2cnccc12)-c1c(C)onc1N	7	LIMK2	LIMK2	>	10000
UNC10225095A	CCn1c(nc2cnccc12)-c1c(C)onc1N	7	PIK3CB	PIK3CB	>	10000
UNC10225097A	Cc1cccc2c(c(nn12)-c1ccc(F)cc1)-c1ccncn1	6	CIT	CIT	=	2500
UNC10225097A	Cc1cccc2c(c(nn12)-c1ccc(F)cc1)-c1ccncn1	6	CLK1	CLK1	>	10000
UNC10225097A	Cc1cccc2c(c(nn12)-c1ccc(F)cc1)-c1ccncn1	6	CLK3	CLK3	>	10000
UNC10225097A	Cc1cccc2c(c(nn12)-c1ccc(F)cc1)-c1ccncn1	6	CSNK1D	CSNK1D	=	1200
UNC10225097A	Cc1cccc2c(c(nn12)-c1ccc(F)cc1)-c1ccncn1	6	CSNK1E	CSNK1E	=	3400
UNC10225097A	Cc1cccc2c(c(nn12)-c1ccc(F)cc1)-c1ccncn1	6	CSNK1G3	CSNK1G3	>	10000
UNC10225097A	Cc1cccc2c(c(nn12)-c1ccc(F)cc1)-c1ccncn1	6	ERK8	MAPK15	>	10000
UNC10225097A	Cc1cccc2c(c(nn12)-c1ccc(F)cc1)-c1ccncn1	6	p38-alpha	MAPK14	=	1300
UNC10225097A	Cc1cccc2c(c(nn12)-c1ccc(F)cc1)-c1ccncn1	6	RSK4(Kin.Dc	RPS6KA6	=	6800
UNC10225099A	Cl.Cc1c(O)cccc1Nc1ccnc2ccc(cc12)C(F)(F)F	3	ACVR2B	ACVR2B	=	240
UNC10225099A	Cl.Cc1c(O)cccc1Nc1ccnc2ccc(cc12)C(F)(F)F	3	ADCK4	ADCK4	=	510
UNC10225099A	Cl.Cc1c(O)cccc1Nc1ccnc2ccc(cc12)C(F)(F)F	3	BRK	PTK6	=	190
UNC10225099A	Cl.Cc1c(O)cccc1Nc1ccnc2ccc(cc12)C(F)(F)F	3	HUNK	HUNK	>	10000

UNC10225101A	Fc1cccc(COc2ccc(Nc3ncnc4sc(cc34)-c3ccc[nH]3)cc2Cl)c1	15	CLK4	CLK4	>	10000
UNC10225101A	Fc1cccc(COc2ccc(Nc3ncnc4sc(cc34)-c3ccc[nH]3)cc2Cl)c1	15	EGFR	EGFR	=	2.6
UNC10225101A	Fc1cccc(COc2ccc(Nc3ncnc4sc(cc34)-c3ccc[nH]3)cc2Cl)c1	15	ERBB2	ERBB2	=	100
UNC10225101A	Fc1cccc(COc2ccc(Nc3ncnc4sc(cc34)-c3ccc[nH]3)cc2Cl)c1	15	ERBB4	ERBB4	=	560
UNC10225101A	Fc1cccc(COc2ccc(Nc3ncnc4sc(cc34)-c3ccc[nH]3)cc2Cl)c1	15	JAK2(JH1do	JAK2	>	10000
UNC10225101A	Fc1cccc(COc2ccc(Nc3ncnc4sc(cc34)-c3ccc[nH]3)cc2Cl)c1	15	LZK	MAP3K13	>	10000
UNC10225101A	Fc1cccc(COc2ccc(Nc3ncnc4sc(cc34)-c3ccc[nH]3)cc2Cl)c1	15	PRKCH	PRKCH	>	10000
UNC10225101A	Fc1cccc(COc2ccc(Nc3ncnc4sc(cc34)-c3ccc[nH]3)cc2Cl)c1	15	RPS6KA5(Ki	RPS6KA5	>	10000
UNC10225105A	COc1cc(Nc2nccc(n2)N2CCC(C2)NC(=O)Nc2cc(C)ccc2C)cc(OC)c1OC	8	AURKA	AURKA	=	1100
UNC10225105A	COc1cc(Nc2nccc(n2)N2CCC(C2)NC(=O)Nc2cc(C)ccc2C)cc(OC)c1OC	8	AURKB	AURKB	=	240
UNC10225105A	COc1cc(Nc2nccc(n2)N2CCC(C2)NC(=O)Nc2cc(C)ccc2C)cc(OC)c1OC	8	AURKC	AURKC	=	200
UNC10225105A	COc1cc(Nc2nccc(n2)N2CCC(C2)NC(=O)Nc2cc(C)ccc2C)cc(OC)c1OC	8	EPHB6	EPHB6	=	1100
UNC10225105A	COc1cc(Nc2nccc(n2)N2CCC(C2)NC(=O)Nc2cc(C)ccc2C)cc(OC)c1OC	8	MEK5	MAP2K5	=	53
UNC10225105A	COc1cc(Nc2nccc(n2)N2CCC(C2)NC(=O)Nc2cc(C)ccc2C)cc(OC)c1OC	8	NEK5	NEK5	=	2300
UNC10225105A	COc1cc(Nc2nccc(n2)N2CCC(C2)NC(=O)Nc2cc(C)ccc2C)cc(OC)c1OC	8	ULK3	ULK3	=	4300
UNC10225109A	COc1cc(Nc2ccnc3ccc(cc23)C(F)(F)F)cc(OC)c1OC	2	ADCK3	CABC1	=	220
UNC10225109A	COc1cc(Nc2ccnc3ccc(cc23)C(F)(F)F)cc(OC)c1OC	2	GAK	GAK	=	31
UNC10225109A	COc1cc(Nc2ccnc3ccc(cc23)C(F)(F)F)cc(OC)c1OC	2	JAK3(JH1do	JAK3	>	10000
UNC10225109A	COc1cc(Nc2ccnc3ccc(cc23)C(F)(F)F)cc(OC)c1OC	2	NIM1	MGC42105	>	10000
UNC10225109A	COc1cc(Nc2ccnc3ccc(cc23)C(F)(F)F)cc(OC)c1OC	2	RSK2(Kin.Dc	RPS6KA3	>	10000
UNC10225110A	COc1cccc(NC(=O)NC2CCN(C2)c2ccnc(Nc3ccc(F)cc3)n2)c1	2	ACVR2A	ACVR2A	>	10000
UNC10225110A	COc1cccc(NC(=O)NC2CCN(C2)c2ccnc(Nc3ccc(F)cc3)n2)c1	2	MAP4K4	MAP4K4	>	10000
UNC10225110A	COc1cccc(NC(=O)NC2CCN(C2)c2ccnc(Nc3ccc(F)cc3)n2)c1	2	MAP4K5	MAP4K5	>	10000
UNC10225110A	COc1cccc(NC(=O)NC2CCN(C2)c2ccnc(Nc3ccc(F)cc3)n2)c1	2	MEK5	MAP2K5	=	500
UNC10225110A	COc1cccc(NC(=O)NC2CCN(C2)c2ccnc(Nc3ccc(F)cc3)n2)c1	2	TRKC	NTRK3	=	310
UNC10225112A	COc1ccc(CNC(=O)NC2CCN(C2)c2ccnc(Nc3ccc(F)cc3)n2)cc1	0	MINK	MINK1	>	10000
UNC10225112A	COc1ccc(CNC(=O)NC2CCN(C2)c2ccnc(Nc3ccc(F)cc3)n2)cc1	0	SRPK2	SRPK2	>	10000
UNC10225113A	CCCc1ccc(cc1)S(=O)(=O)NC1CCN(C1)c1ccnc(Nc2ccc(OC)cc2)n1	7	DMPK2	CDC42BPG	>	10000
UNC10225113A	CCCc1ccc(cc1)S(=O)(=O)NC1CCN(C1)c1ccnc(Nc2ccc(OC)cc2)n1	7	LIMK2	LIMK2	>	10000
UNC10225113A	CCCc1ccc(cc1)S(=O)(=O)NC1CCN(C1)c1ccnc(Nc2ccc(OC)cc2)n1	7	LTK	LTK	=	8700
UNC10225113A	CCCc1ccc(cc1)S(=O)(=O)NC1CCN(C1)c1ccnc(Nc2ccc(OC)cc2)n1	7	OSR1	OXSR1	>	10000
UNC10225113A	CCCc1ccc(cc1)S(=O)(=O)NC1CCN(C1)c1ccnc(Nc2ccc(OC)cc2)n1	7	PAK3	PAK3	>	10000
UNC10225113A	CCCc1ccc(cc1)S(=O)(=O)NC1CCN(C1)c1ccnc(Nc2ccc(OC)cc2)n1	7	SBK1	SBK1	>	10000
UNC10225113A	CCCc1ccc(cc1)S(=O)(=O)NC1CCN(C1)c1ccnc(Nc2ccc(OC)cc2)n1	7	ULK1	ULK1	>	10000
UNC10225114A	CCn1c(nc2cnccc12)-c1cccnc1O	0	ADCK4	ADCK4	>	10000
UNC10225114A	CCn1c(nc2cnccc12)-c1cccnc1O	0	CSNK1A1L	CSNK1A1L	>	10000
UNC10225114A	CCn1c(nc2cnccc12)-c1cccnc1O	0	WEE2	WEE2	>	10000
UNC10225115A	Cl.Cl.CS(=O)(=O)CCNCc1nc(cs1)-c1ccc2ncnc(Nc3ccc4n(Cc5ccccc5)ncc4c3)c2c1	15	CDKL3	CDKL3	>	10000

UNC10225115A	Cl.Cl.CS(=O)(=O)CCNCc1nc(cs1)-c1ccc2ncnc(Nc3ccc4n(Cc5ccccc5)ncc4c3)c2c1	15	EGFR	EGFR	=	0.8
UNC10225115A	Cl.Cl.CS(=O)(=O)CCNCc1nc(cs1)-c1ccc2ncnc(Nc3ccc4n(Cc5ccccc5)ncc4c3)c2c1	15	ERBB2	ERBB2	=	1.1
UNC10225115A	Cl.Cl.CS(=O)(=O)CCNCc1nc(cs1)-c1ccc2ncnc(Nc3ccc4n(Cc5ccccc5)ncc4c3)c2c1	15	ERBB3	ERBB3	=	69
UNC10225115A	Cl.Cl.CS(=O)(=O)CCNCc1nc(cs1)-c1ccc2ncnc(Nc3ccc4n(Cc5ccccc5)ncc4c3)c2c1	15	ERBB4	ERBB4	=	8.2
UNC10225115A	Cl.Cl.CS(=O)(=O)CCNCc1nc(cs1)-c1ccc2ncnc(Nc3ccc4n(Cc5ccccc5)ncc4c3)c2c1	15	MEK5	MAP2K5	=	43
UNC10225115A	Cl.Cl.CS(=O)(=O)CCNCc1nc(cs1)-c1ccc2ncnc(Nc3ccc4n(Cc5ccccc5)ncc4c3)c2c1	15	RIPK2	RIPK2	=	67
UNC10225116A	Fc1ccc(cc1)-c1nn2cccc(F)c2c1-c1ccncc1	7	CIT	CIT	=	310
UNC10225116A	Fc1ccc(cc1)-c1nn2cccc(F)c2c1-c1ccncc1	7	CSNK1D	CSNK1D	=	77
UNC10225116A	Fc1ccc(cc1)-c1nn2cccc(F)c2c1-c1ccncc1	7	CSNK1E	CSNK1E	=	220
UNC10225116A	Fc1ccc(cc1)-c1nn2cccc(F)c2c1-c1ccncc1	7	DMPK2	CDC42BPG	=	1000
UNC10225116A	Fc1ccc(cc1)-c1nn2cccc(F)c2c1-c1ccncc1	7	JNK2	MAPK9	=	230
UNC10225116A	Fc1ccc(cc1)-c1nn2cccc(F)c2c1-c1ccncc1	7	JNK3	MAPK10	=	58
UNC10225116A	Fc1ccc(cc1)-c1nn2cccc(F)c2c1-c1ccncc1	7	p38-alpha	MAPK14	=	140
UNC10225116A	Fc1ccc(cc1)-c1nn2cccc(F)c2c1-c1ccncc1	7	RET	RET	>	10000
UNC10225119A	Nc1nccc2scc(-c3ccc4N(CCc4c3)C(=O)Cc3ccccc3)c12	2	EPHB6	EPHB6	=	340
UNC10225119A	Nc1nccc2scc(-c3ccc4N(CCc4c3)C(=O)Cc3ccccc3)c12	2	GRK3	ADRBK2	>	10000
UNC10225119A	Nc1nccc2scc(-c3ccc4N(CCc4c3)C(=O)Cc3ccccc3)c12	2	MEK5	MAP2K5	=	530
UNC10225122A	Clc1cccc(c1)-c1nc(Nc2ccncc2)c2ccccc2n1	2	DMPK2	CDC42BPG	>	10000
UNC10225122A	Clc1cccc(c1)-c1nc(Nc2ccncc2)c2ccccc2n1	2	EPHA1	EPHA1	>	10000
UNC10225123A	Cn1cnc2c(N)ncnc12	9	CDKL1	CDKL1	>	10000
UNC10225123A	Cn1cnc2c(N)ncnc12	9	DAPK1	DAPK1	>	10000
UNC10225123A	Cn1cnc2c(N)ncnc12	9	FLT3-autoin	FLT3	>	10000
UNC10225123A	Cn1cnc2c(N)ncnc12	9	PDGFRA	PDGFRA	>	10000
UNC10225123A	Cn1cnc2c(N)ncnc12	9	PRKG2	PRKG2	>	10000
UNC10225123A	Cn1cnc2c(N)ncnc12	9	RSK1(Kin.Dc	RPS6KA1	>	10000
UNC10225123A	Cn1cnc2c(N)ncnc12	9	VEGFR2	KDR	>	10000
UNC10225123A	Cn1cnc2c(N)ncnc12	9	VRK2	VRK2	>	10000
UNC10225125A	Cc1ccc(cc1)-c1cc(ccn1)-c1c[nH]nc1-c1ccccn1	2	ARK5	NUAK1	>	10000
UNC10225125A	Cc1ccc(cc1)-c1cc(ccn1)-c1c[nH]nc1-c1ccccn1	2	GAK	GAK	=	110
UNC10225125A	Cc1ccc(cc1)-c1cc(ccn1)-c1c[nH]nc1-c1ccccn1	2	MINK	MINK1	=	61
UNC10225125A	Cc1ccc(cc1)-c1cc(ccn1)-c1c[nH]nc1-c1ccccn1	2	TGFBR1	TGFBR1	=	260
UNC10225125A	Cc1ccc(cc1)-c1cc(ccn1)-c1c[nH]nc1-c1ccccn1	2	TNIK	TNIK	=	160
UNC10225125A	Cc1ccc(cc1)-c1cc(ccn1)-c1c[nH]nc1-c1ccccn1	2	WNK4	WNK4	>	10000
UNC10225126A	Cc1csc(n1)-c1nc(Nc2ccncc2)c2ccccc2n1	3	ACVR1B	ACVR1B	=	39
UNC10225126A	Cc1csc(n1)-c1nc(Nc2ccncc2)c2ccccc2n1	3	HASPIN	GSG2	=	77
UNC10225126A	Cc1csc(n1)-c1nc(Nc2ccncc2)c2ccccc2n1	3	TGFBR1	TGFBR1	=	8.9
UNC10225126A	Cc1csc(n1)-c1nc(Nc2ccncc2)c2ccccc2n1	3	TGFBR2	TGFBR2	=	250
UNC10225126A	Cc1csc(n1)-c1nc(Nc2ccncc2)c2ccccc2n1	3	TYK2(JH1do	TYK2	>	10000
UNC10225126A	Cc1csc(n1)-c1nc(Nc2ccncc2)c2ccccc2n1	3	TYK2(JH2do	TYK2	=	530
UNC10225127A	COc1ccc(COc2cc(sc2C(N)=O)-n2cnc3ccccc23)cc1	4	PIK3CB	PIK3CB	>	10000
UNC10225127A	COc1ccc(COc2cc(sc2C(N)=O)-n2cnc3ccccc23)cc1	4	PLK1	PLK1	=	37
UNC10225127A	COc1ccc(COc2cc(sc2C(N)=O)-n2cnc3ccccc23)cc1	4	PLK2	PLK2	=	250
UNC10225127A	COc1ccc(COc2cc(sc2C(N)=O)-n2cnc3ccccc23)cc1	4	PLK3	PLK3	=	380
UNC10225127A	COc1ccc(COc2cc(sc2C(N)=O)-n2cnc3ccccc23)cc1	4	RIOK2	RIOK2	=	76
UNC10225129A	Fc1ccc(CNc2nccc(n2)N2CCC(CC2)NC(=O)Nc2ccc3OCOc3c2)cc1	2	MELK	MELK	>	10000
UNC10225129A	Fc1ccc(CNc2nccc(n2)N2CCC(CC2)NC(=O)Nc2ccc3OCOc3c2)cc1	2	RSK2(Kin.Dc	RPS6KA3	>	10000
UNC10225131A	Clc1cccc(Cn2ccn3c2nc(cc3=O)N2CCOCC2)c1	3	EPHA3	EPHA3	>	10000

UNC10225131A	Clc1cccc(Cn2ccn3c2nc(cc3=O)N2CCOCC2)c1	3	ERBB4	ERBB4	>	10000
UNC10225131A	Clc1cccc(Cn2ccn3c2nc(cc3=O)N2CCOCC2)c1	3	MARK2	MARK2	>	10000
UNC10225131A	Clc1cccc(Cn2ccn3c2nc(cc3=O)N2CCOCC2)c1	3	PIK3CB	PIK3CB	=	15
UNC10225131A	Clc1cccc(Cn2ccn3c2nc(cc3=O)N2CCOCC2)c1	3	RET	RET	>	10000
UNC10225131A	Clc1cccc(Cn2ccn3c2nc(cc3=O)N2CCOCC2)c1	3	VPS34	PIK3C3	=	35
UNC10225133A	Cl.CCS(=O)(=O)c1ccc(OC)c(Nc2ncc(o2)-c2ccc(F)cc2)c1	0	MAK	MAK	>	10000
UNC10225134A	COc1ccc(Nc2nccc(n2)N2CCC(C2)NS(=O)(=O)c2ccc(NC(C)=O)cc2)cc1	6	FGFR4	FGFR4	>	10000
UNC10225134A	COc1ccc(Nc2nccc(n2)N2CCC(C2)NS(=O)(=O)c2ccc(NC(C)=O)cc2)cc1	6	JAK2(JH1do	JAK2	=	38
UNC10225134A	COc1ccc(Nc2nccc(n2)N2CCC(C2)NS(=O)(=O)c2ccc(NC(C)=O)cc2)cc1	6	p38-alpha	MAPK14	>	10000
UNC10225134A	COc1ccc(Nc2nccc(n2)N2CCC(C2)NS(=O)(=O)c2ccc(NC(C)=O)cc2)cc1	6	RIPK2	RIPK2	>	10000
UNC10225134A	COc1ccc(Nc2nccc(n2)N2CCC(C2)NS(=O)(=O)c2ccc(NC(C)=O)cc2)cc1	6	TNNI3K	TNNI3K	>	10000
UNC10225134A	COc1ccc(Nc2nccc(n2)N2CCC(C2)NS(=O)(=O)c2ccc(NC(C)=O)cc2)cc1	6	TYK2(JH1do	TYK2	=	86
UNC10225134A	COc1ccc(Nc2nccc(n2)N2CCC(C2)NS(=O)(=O)c2ccc(NC(C)=O)cc2)cc1	6	VPS34	PIK3C3	=	97
UNC10225136A	Clc1cccc(Cn2c3ccccc3n3c2nc(cc3=O)N2CCOCC2)c1Cl	4	CDK11	CDK19	>	10000
UNC10225136A	Clc1cccc(Cn2c3ccccc3n3c2nc(cc3=O)N2CCOCC2)c1Cl	4	MELK	MELK	>	10000
UNC10225136A	Clc1cccc(Cn2c3ccccc3n3c2nc(cc3=O)N2CCOCC2)c1Cl	4	PIK3CB	PIK3CB	=	860
UNC10225136A	Clc1cccc(Cn2c3ccccc3n3c2nc(cc3=O)N2CCOCC2)c1Cl	4	VPS34	PIK3C3	=	470
UNC10225137A	Clc1cccc(c1)C1=C(N2CCc3ccccc23)C(=O)NC1=O	0	WNK4	WNK4	>	10000
UNC10225138A	FC(F)(F)c1ccc(cc1)-c1nn2ncccc2c1-c1ccnc(Nc2cccc(c2)C(F)(F)F)n1	1	DDR1	DDR1	>	10000
UNC10225138A	FC(F)(F)c1ccc(cc1)-c1nn2ncccc2c1-c1ccnc(Nc2cccc(c2)C(F)(F)F)n1	1	EPHA4	EPHA4	>	10000
UNC10225138A	FC(F)(F)c1ccc(cc1)-c1nn2ncccc2c1-c1ccnc(Nc2cccc(c2)C(F)(F)F)n1	1	FGFR2	FGFR2	>	10000
UNC10225140A	Brc1cc2c(NC(=O)C3CCCC3)n[nH]c2nc1-c1cccs1	2	CDKL2	CDKL2	=	330
UNC10225140A	Brc1cc2c(NC(=O)C3CCCC3)n[nH]c2nc1-c1cccs1	2	GSK3A	GSK3A	=	62
UNC10225140A	Brc1cc2c(NC(=O)C3CCCC3)n[nH]c2nc1-c1cccs1	2	GSK3B	GSK3B	=	120
UNC10225140A	Brc1cc2c(NC(=O)C3CCCC3)n[nH]c2nc1-c1cccs1	2	MLCK	MYLK3	=	290
UNC10225140A	Brc1cc2c(NC(=O)C3CCCC3)n[nH]c2nc1-c1cccs1	2	MYLK2	MYLK2	=	26
UNC10225141A	Cl.Cn1ccc(n1)-c1cccc(Nc2ccnc3cc(ccc23)-c2nccs2)c1	4	EPHA3	EPHA3	>	10000
UNC10225141A	Cl.Cn1ccc(n1)-c1cccc(Nc2ccnc3cc(ccc23)-c2nccs2)c1	4	EPHB6	EPHB6	=	82
UNC10225141A	Cl.Cn1ccc(n1)-c1cccc(Nc2ccnc3cc(ccc23)-c2nccs2)c1	4	MARK2	MARK2	>	10000
UNC10225141A	Cl.Cn1ccc(n1)-c1cccc(Nc2ccnc3cc(ccc23)-c2nccs2)c1	4	MEK5	MAP2K5	=	960
UNC10225141A	Cl.Cn1ccc(n1)-c1cccc(Nc2ccnc3cc(ccc23)-c2nccs2)c1	4	PAK7	PAK7	>	10000
UNC10225143A	C[C@H]1CC[C@H](CN1c1cc(nc(N)n1)-c1ccc2c(N)n[nH]c2c1)C(=O)Nc1ccccc1	2	DRAK1	STK17A	=	11
UNC10225143A	C[C@H]1CC[C@H](CN1c1cc(nc(N)n1)-c1ccc2c(N)n[nH]c2c1)C(=O)Nc1ccccc1	2	MET	MET	>	10000
UNC10225145A	Cl.Cl.Cl.N#Cc1ccc(CCNc2nccc(n2)-c2cccc(CN3CCNCC3)c2)cc1	4	CIT	CIT	=	8600
UNC10225145A	Cl.Cl.Cl.N#Cc1ccc(CCNc2nccc(n2)-c2cccc(CN3CCNCC3)c2)cc1	4	INSR	INSR	>	10000
UNC10225145A	Cl.Cl.Cl.N#Cc1ccc(CCNc2nccc(n2)-c2cccc(CN3CCNCC3)c2)cc1	4	STK33	STK33	=	3900
UNC10225145A	Cl.Cl.Cl.N#Cc1ccc(CCNc2nccc(n2)-c2cccc(CN3CCNCC3)c2)cc1	4	WEE1	WEE1	>	10000
UNC10225145A	Cl.Cl.Cl.N#Cc1ccc(CCNc2nccc(n2)-c2cccc(CN3CCNCC3)c2)cc1	4	WNK4	WNK4	>	10000

UNC10225147A	COc1cccc(CNC(=O)NC2CCN(C2)c2ccnc(Nc3ccc(F)cc3)n2)c1	3	CAMK2G	CAMK2G	>	10000
UNC10225147A	COc1cccc(CNC(=O)NC2CCN(C2)c2ccnc(Nc3ccc(F)cc3)n2)c1	3	TESK1	TESK1	=	9800
UNC10225147A	COc1cccc(CNC(=O)NC2CCN(C2)c2ccnc(Nc3ccc(F)cc3)n2)c1	3	TXK	TXK	>	10000
UNC10225147A	COc1cccc(CNC(=O)NC2CCN(C2)c2ccnc(Nc3ccc(F)cc3)n2)c1	3	ZAK	ZAK	>	10000
UNC10225148A	CC(C)(C)OC(=O)NC1CCCN(C1)c1cc(nc(N)n1)-c1ccc2c(N)n[nH]c2c1	6	DRAK1	STK17A	=	130
UNC10225148A	CC(C)(C)OC(=O)NC1CCCN(C1)c1cc(nc(N)n1)-c1ccc2c(N)n[nH]c2c1	6	DYRK1B	DYRK1B	>	10000
UNC10225148A	CC(C)(C)OC(=O)NC1CCCN(C1)c1cc(nc(N)n1)-c1ccc2c(N)n[nH]c2c1	6	DYRK2	DYRK2	=	440
UNC10225148A	CC(C)(C)OC(=O)NC1CCCN(C1)c1cc(nc(N)n1)-c1ccc2c(N)n[nH]c2c1	6	EPHB2	EPHB2	>	10000
UNC10225148A	CC(C)(C)OC(=O)NC1CCCN(C1)c1cc(nc(N)n1)-c1ccc2c(N)n[nH]c2c1	6	MEK1	MAP2K1	>	10000
UNC10225148A	CC(C)(C)OC(=O)NC1CCCN(C1)c1cc(nc(N)n1)-c1ccc2c(N)n[nH]c2c1	6	PDPK1	PDPK1	=	37
UNC10225148A	CC(C)(C)OC(=O)NC1CCCN(C1)c1cc(nc(N)n1)-c1ccc2c(N)n[nH]c2c1	6	RSK2(Kin.Dc	RPS6KA3	>	10000
UNC10225148A	CC(C)(C)OC(=O)NC1CCCN(C1)c1cc(nc(N)n1)-c1ccc2c(N)n[nH]c2c1	6	TSSK3	TSSK3	>	10000
UNC10225149A	Nc1nc(N)c2nc([nH]c2n1)-c1ccccc1	0	JAK1(JH2do	JAK1	=	150
UNC10225152A	Fc1ccc(Nc2nccc(n2)N2CCC(C2)NC(=O)Nc2ccccc2F)cc1	6	CSNK1G2	CSNK1G2	>	10000
UNC10225152A	Fc1ccc(Nc2nccc(n2)N2CCC(C2)NC(=O)Nc2ccccc2F)cc1	6	ERK1	MAPK3	>	10000
UNC10225152A	Fc1ccc(Nc2nccc(n2)N2CCC(C2)NC(=O)Nc2ccccc2F)cc1	6	ICK	ICK	>	10000
UNC10225152A	Fc1ccc(Nc2nccc(n2)N2CCC(C2)NC(=O)Nc2ccccc2F)cc1	6	IGF1R	IGF1R	>	10000
UNC10225152A	Fc1ccc(Nc2nccc(n2)N2CCC(C2)NC(=O)Nc2ccccc2F)cc1	6	PIK4CB	PI4KB	>	10000
UNC10225152A	Fc1ccc(Nc2nccc(n2)N2CCC(C2)NC(=O)Nc2ccccc2F)cc1	6	RIPK4	RIPK4	>	10000
UNC10225156A	CCn1c(nc2cnccc12)-c1ccccn1	9	BRSK1	BRSK1	>	10000
UNC10225156A	CCn1c(nc2cnccc12)-c1ccccn1	9	CDC2L5	CDK13	>	10000
UNC10225156A	CCn1c(nc2cnccc12)-c1ccccn1	9	IRAK3	IRAK3	=	7500
UNC10225156A	CCn1c(nc2cnccc12)-c1ccccn1	9	MEK6	MAP2K6	>	10000
UNC10225156A	CCn1c(nc2cnccc12)-c1ccccn1	9	NEK1	NEK1	>	10000
UNC10225156A	CCn1c(nc2cnccc12)-c1ccccn1	9	PDPK1	PDPK1	>	10000
UNC10225156A	CCn1c(nc2cnccc12)-c1ccccn1	9	PKAC-alpha	PRKACA	>	10000
UNC10225156A	CCn1c(nc2cnccc12)-c1ccccn1	9	PRKX	PRKX	>	10000
UNC10225156A	CCn1c(nc2cnccc12)-c1ccccn1	9	YSK1	STK25	>	10000
UNC10225161A	COC(=O)C(NC(=O)NC1CCN(C1)c1ccnc(Nc2ccc(F)cc2)n1)c1ccccc1	2	CDK2	CDK2	>	10000
UNC10225161A	COC(=O)C(NC(=O)NC1CCN(C1)c1ccnc(Nc2ccc(F)cc2)n1)c1ccccc1	2	CSNK1D	CSNK1D	>	10000
UNC10225161A	COC(=O)C(NC(=O)NC1CCN(C1)c1ccnc(Nc2ccc(F)cc2)n1)c1ccccc1	2	EPHB6	EPHB6	=	58
UNC10225161A	COC(=O)C(NC(=O)NC1CCN(C1)c1ccnc(Nc2ccc(F)cc2)n1)c1ccccc1	2	MEK5	MAP2K5	=	270
UNC10225162A	COc1ccc(Nc2nccc(n2)N2CCC(CC2)NC(=O)C(C)c2ccc(Cl)cc2)cc1	7	CDKL5	CDKL5	>	10000
UNC10225162A	COc1ccc(Nc2nccc(n2)N2CCC(CC2)NC(=O)C(C)c2ccc(Cl)cc2)cc1	7	DYRK1A	DYRK1A	>	10000
UNC10225162A	COc1ccc(Nc2nccc(n2)N2CCC(CC2)NC(=O)C(C)c2ccc(Cl)cc2)cc1	7	EPHB6	EPHB6	>	10000

UNC10225162A	COc1ccc(Nc2nccc(n2)N2CCC(CC2)NC(=O)C(C)c2ccc(Cl)cc2)cc1	7	IKK-epsilon	IKBKE	>	10000
UNC10225162A	COc1ccc(Nc2nccc(n2)N2CCC(CC2)NC(=O)C(C)c2ccc(Cl)cc2)cc1	7	LATS2	LATS2	>	10000
UNC10225162A	COc1ccc(Nc2nccc(n2)N2CCC(CC2)NC(=O)C(C)c2ccc(Cl)cc2)cc1	7	PCTK1	CDK16	>	10000
UNC10225162A	COc1ccc(Nc2nccc(n2)N2CCC(CC2)NC(=O)C(C)c2ccc(Cl)cc2)cc1	7	RSK2(Kin.Dc	RPS6KA3	>	10000
UNC10225163A	Fc1ccc(cc1)-c1nc2SCCn2c1-c1ccncc1	8	CIT	CIT	=	760
UNC10225163A	Fc1ccc(cc1)-c1nc2SCCn2c1-c1ccncc1	8	CSNK1A1	CSNK1A1	=	260
UNC10225163A	Fc1ccc(cc1)-c1nc2SCCn2c1-c1ccncc1	8	CSNK1D	CSNK1D	=	83
UNC10225163A	Fc1ccc(cc1)-c1nc2SCCn2c1-c1ccncc1	8	CSNK1E	CSNK1E	=	180
UNC10225163A	Fc1ccc(cc1)-c1nc2SCCn2c1-c1ccncc1	8	DMPK2	CDC42BPG	=	1200
UNC10225163A	Fc1ccc(cc1)-c1nc2SCCn2c1-c1ccncc1	8	JNK2	MAPK9	=	270
UNC10225163A	Fc1ccc(cc1)-c1nc2SCCn2c1-c1ccncc1	8	JNK3	MAPK10	=	57
UNC10225163A	Fc1ccc(cc1)-c1nc2SCCn2c1-c1ccncc1	8	p38-alpha	MAPK14	=	130
UNC10225163A	Fc1ccc(cc1)-c1nc2SCCn2c1-c1ccncc1	8	PIK3C2B	PIK3C2B	>	10000
UNC10225163A	Fc1ccc(cc1)-c1nc2SCCn2c1-c1ccncc1	8	PIK3CA	PIK3CA	>	10000
UNC10225163A	Fc1ccc(cc1)-c1nc2SCCn2c1-c1ccncc1	8	PIK3CG	PIK3CG	>	10000
UNC10225163A	Fc1ccc(cc1)-c1nc2SCCn2c1-c1ccncc1	8	RSK4(Kin.Dc	RPS6KA6	=	260
UNC10225164A	COC(=O)c1cccc(NC(=O)NC2CCN(C2)c2ccnc(Nc3ccc(F)cc3)n2)c1	0	CDKL2	CDKL2	=	680
UNC10225164A	COC(=O)c1cccc(NC(=O)NC2CCN(C2)c2ccnc(Nc3ccc(F)cc3)n2)c1	0	TRKC	NTRK3	=	340
UNC10225165A	Cn1cc(-c2ccc3N(CCc3c2)C(=O)Cc2cccc(c2)C(F)(F)F)c2c(N)ncnc12	5	DAPK3	DAPK3	>	10000
UNC10225165A	Cn1cc(-c2ccc3N(CCc3c2)C(=O)Cc2cccc(c2)C(F)(F)F)c2c(N)ncnc12	5	DDR1	DDR1	=	280
UNC10225165A	Cn1cc(-c2ccc3N(CCc3c2)C(=O)Cc2cccc(c2)C(F)(F)F)c2c(N)ncnc12	5	GRK4	GRK4	>	10000
UNC10225165A	Cn1cc(-c2ccc3N(CCc3c2)C(=O)Cc2cccc(c2)C(F)(F)F)c2c(N)ncnc12	5	KIT	KIT	=	5200
UNC10225165A	Cn1cc(-c2ccc3N(CCc3c2)C(=O)Cc2cccc(c2)C(F)(F)F)c2c(N)ncnc12	5	MAPKAPK2	MAPKAPK2	>	10000
UNC10225165A	Cn1cc(-c2ccc3N(CCc3c2)C(=O)Cc2cccc(c2)C(F)(F)F)c2c(N)ncnc12	5	SRPK3	SRPK3	>	10000
UNC10225167A	Clc1cccc(Cn2ccn3c2nc(cc3=O)N2CCOCC2)c1Cl	4	AXL	AXL	>	10000
UNC10225167A	Clc1cccc(Cn2ccn3c2nc(cc3=O)N2CCOCC2)c1Cl	4	LYN	LYN	>	10000
UNC10225167A	Clc1cccc(Cn2ccn3c2nc(cc3=O)N2CCOCC2)c1Cl	4	PIK3CB	PIK3CB	=	3.5
UNC10225167A	Clc1cccc(Cn2ccn3c2nc(cc3=O)N2CCOCC2)c1Cl	4	PIK3CD	PIK3CD	=	79
UNC10225167A	Clc1cccc(Cn2ccn3c2nc(cc3=O)N2CCOCC2)c1Cl	4	VPS34	PIK3C3	=	2.1
UNC10225168A	Clc1ccccc1C1=C(Nc2ccccc2)C(=O)NC1=O	2	CSNK1G1	CSNK1G1	>	10000
UNC10225168A	Clc1ccccc1C1=C(Nc2ccccc2)C(=O)NC1=O	2	GAK	GAK	>	10000
UNC10225168A	Clc1ccccc1C1=C(Nc2ccccc2)C(=O)NC1=O	2	LKB1	STK11	>	10000
UNC10225169A	c1[nH]nc(c1-c1ccnc(c1)-c1ccccc1)-c1ccccn1	1	RIPK2	RIPK2	=	7600
UNC10225169A	c1[nH]nc(c1-c1ccnc(c1)-c1ccccc1)-c1ccccn1	1	TNNI3K	TNNI3K	>	10000
UNC10225170A	Cl.COc1cc2nccc(Nc3cccc(Oc4ccccc4)c3)c2cc1OC	2	MEK1	MAP2K1	=	100
UNC10225170A	Cl.COc1cc2nccc(Nc3cccc(Oc4ccccc4)c3)c2cc1OC	2	MEK2	MAP2K2	=	270
UNC10225170A	Cl.COc1cc2nccc(Nc3cccc(Oc4ccccc4)c3)c2cc1OC	2	MEK5	MAP2K5	=	11
UNC10225170A	Cl.COc1cc2nccc(Nc3cccc(Oc4ccccc4)c3)c2cc1OC	2	RIPK2	RIPK2	=	440
UNC10225175A	Cc1cccc(n1)-c1nc(C)cc(Nc2ccncc2)n1	9	AAK1	AAK1	>	10000
UNC10225175A	Cc1cccc(n1)-c1nc(C)cc(Nc2ccncc2)n1	9	CAMK1G	CAMK1G	>	10000
UNC10225175A	Cc1cccc(n1)-c1nc(C)cc(Nc2ccncc2)n1	9	CAMK2D	CAMK2D	>	10000
UNC10225175A	Cc1cccc(n1)-c1nc(C)cc(Nc2ccncc2)n1	9	CDC2L1	CDK11B	>	10000
UNC10225175A	Cc1cccc(n1)-c1nc(C)cc(Nc2ccncc2)n1	9	CDK9	CDK9	>	10000

UNC10225175A	Cc1cccc(n1)-c1nc(C)cc(Nc2ccncc2)n1	9	DMPK2	CDC42BPG	=	560
UNC10225175A	Cc1cccc(n1)-c1nc(C)cc(Nc2ccncc2)n1	9	ERK4	MAPK4	>	10000
UNC10225175A	Cc1cccc(n1)-c1nc(C)cc(Nc2ccncc2)n1	9	MRCKB	CDC42BPB	=	580
UNC10225175A	Cc1cccc(n1)-c1nc(C)cc(Nc2ccncc2)n1	9	PRKD2	PRKD2	=	1100
UNC10225175A	Cc1cccc(n1)-c1nc(C)cc(Nc2ccncc2)n1	9	PRKD3	PRKD3	=	1000
UNC10225175A	Cc1cccc(n1)-c1nc(C)cc(Nc2ccncc2)n1	9	RAF1	RAF1	>	10000
UNC10225175A	Cc1cccc(n1)-c1nc(C)cc(Nc2ccncc2)n1	9	TGFBR1	TGFBR1	=	200
UNC10225177A	COc1ccc(Nc2nccc(n2)N2CCC(C2)NC(=O)Nc2ccc3OCOc3c2)cc1	0	CSNK1D	CSNK1D	>	10000
UNC10225178A	Cc1nn2c(nc(cc2=O)N2CCOCC2)n1Cc1cccc(Cl)c1Cl	3	GRK3	ADRBK2	>	10000
UNC10225178A	Cc1nn2c(nc(cc2=O)N2CCOCC2)n1Cc1cccc(Cl)c1Cl	3	PFTK1	CDK14	>	10000
UNC10225178A	Cc1nn2c(nc(cc2=O)N2CCOCC2)n1Cc1cccc(Cl)c1Cl	3	PIK3C2B	PIK3C2B	=	130
UNC10225178A	Cc1nn2c(nc(cc2=O)N2CCOCC2)n1Cc1cccc(Cl)c1Cl	3	PIK3CB	PIK3CB	=	0.79
UNC10225178A	Cc1nn2c(nc(cc2=O)N2CCOCC2)n1Cc1cccc(Cl)c1Cl	3	PIK3CD	PIK3CD	=	200
UNC10225178A	Cc1nn2c(nc(cc2=O)N2CCOCC2)n1Cc1cccc(Cl)c1Cl	3	VPS34	PIK3C3	=	0.43
UNC10225180A	Brc1cc2c(NC(=O)C3CC3)n[nH]c2nc1-c1nccs1	5	BRSK1	BRSK1	=	230
UNC10225180A	Brc1cc2c(NC(=O)C3CC3)n[nH]c2nc1-c1nccs1	5	BRSK2	BRSK2	=	140
UNC10225180A	Brc1cc2c(NC(=O)C3CC3)n[nH]c2nc1-c1nccs1	5	CDK2	CDK2	=	350
UNC10225180A	Brc1cc2c(NC(=O)C3CC3)n[nH]c2nc1-c1nccs1	5	CDKL2	CDKL2	=	480
UNC10225180A	Brc1cc2c(NC(=O)C3CC3)n[nH]c2nc1-c1nccs1	5	ERK8	MAPK15	=	870
UNC10225180A	Brc1cc2c(NC(=O)C3CC3)n[nH]c2nc1-c1nccs1	5	FLT3	FLT3	=	190
UNC10225180A	Brc1cc2c(NC(=O)C3CC3)n[nH]c2nc1-c1nccs1	5	GSK3A	GSK3A	=	290
UNC10225180A	Brc1cc2c(NC(=O)C3CC3)n[nH]c2nc1-c1nccs1	5	MYLK2	MYLK2	=	160
UNC10225180A	Brc1cc2c(NC(=O)C3CC3)n[nH]c2nc1-c1nccs1	5	YSK4	MAP3K19	=	210
UNC10225182A	Cl.Cc1ccc(cc1)C(=O)Nc1ccc(Nc2ccnc3cc(ccc23)-c2ccccn2)cc1O	6	AURKB	AURKB	=	290
UNC10225182A	Cl.Cc1ccc(cc1)C(=O)Nc1ccc(Nc2ccnc3cc(ccc23)-c2ccccn2)cc1O	6	AURKC	AURKC	=	500
UNC10225182A	Cl.Cc1ccc(cc1)C(=O)Nc1ccc(Nc2ccnc3cc(ccc23)-c2ccccn2)cc1O	6	BLK	BLK	=	63
UNC10225182A	Cl.Cc1ccc(cc1)C(=O)Nc1ccc(Nc2ccnc3cc(ccc23)-c2ccccn2)cc1O	6	KIT	KIT	=	2200
UNC10225182A	Cl.Cc1ccc(cc1)C(=O)Nc1ccc(Nc2ccnc3cc(ccc23)-c2ccccn2)cc1O	6	LCK	LCK	=	260
UNC10225182A	Cl.Cc1ccc(cc1)C(=O)Nc1ccc(Nc2ccnc3cc(ccc23)-c2ccccn2)cc1O	6	MEK5	MAP2K5	=	4.7
UNC10225182A	Cl.Cc1ccc(cc1)C(=O)Nc1ccc(Nc2ccnc3cc(ccc23)-c2ccccn2)cc1O	6	PDGFRB	PDGFRB	=	480
UNC10225182A	Cl.Cc1ccc(cc1)C(=O)Nc1ccc(Nc2ccnc3cc(ccc23)-c2ccccn2)cc1O	6	PIK3CD	PIK3CD	>	10000
UNC10225182A	Cl.Cc1ccc(cc1)C(=O)Nc1ccc(Nc2ccnc3cc(ccc23)-c2ccccn2)cc1O	6	RIOK3	RIOK3	>	10000
UNC10225182A	Cl.Cc1ccc(cc1)C(=O)Nc1ccc(Nc2ccnc3cc(ccc23)-c2ccccn2)cc1O	6	SRMS	SRMS	=	350
UNC10225182A	Cl.Cc1ccc(cc1)C(=O)Nc1ccc(Nc2ccnc3cc(ccc23)-c2ccccn2)cc1O	6	TNK2	TNK2	=	120
UNC10225184A	FC(F)(F)c1ccccc1-c1cc(ccn1)-c1c[nH]nc1-c1ccccn1	5	CHEK1	CHEK1	>	10000
UNC10225184A	FC(F)(F)c1ccccc1-c1cc(ccn1)-c1c[nH]nc1-c1ccccn1	5	EPHB6	EPHB6	=	8500
UNC10225184A	FC(F)(F)c1ccccc1-c1cc(ccn1)-c1c[nH]nc1-c1ccccn1	5	IKK-epsilon	IKBKE	>	10000
UNC10225184A	FC(F)(F)c1ccccc1-c1cc(ccn1)-c1c[nH]nc1-c1ccccn1	5	p38-alpha	MAPK14	=	310
UNC10225184A	FC(F)(F)c1ccccc1-c1cc(ccn1)-c1c[nH]nc1-c1ccccn1	5	PRKD3	PRKD3	>	10000
UNC10225185A	Nc1n[nH]c2nnc(-c3ccccc3)c(-c3ccccc3)c12	3	GSK3A	GSK3A	=	300
UNC10225185A	Nc1n[nH]c2nnc(-c3ccccc3)c(-c3ccccc3)c12	3	HIPK2	HIPK2	=	410
UNC10225185A	Nc1n[nH]c2nnc(-c3ccccc3)c(-c3ccccc3)c12	3	LYN	LYN	>	10000
UNC10225185A	Nc1n[nH]c2nnc(-c3ccccc3)c(-c3ccccc3)c12	3	NIK	MAP3K14	>	10000

UNC10225186A	CCCC(=O)Nc1n[nH]c2ncc(cc12)-c1cccc2ccccc12	2	GSK3A	GSK3A	=	360
UNC10225186A	CCCC(=O)Nc1n[nH]c2ncc(cc12)-c1cccc2ccccc12	2	NEK10	NEK10	>	10000
UNC10225186A	CCCC(=O)Nc1n[nH]c2ncc(cc12)-c1cccc2ccccc12	2	TLK2	TLK2	>	10000
UNC10225189A	Fc1cc(Cl)ccc1Nc1ccnc2cc(I)ccc12	0	ACVR2B	ACVR2B	=	110
UNC10225189A	Fc1cc(Cl)ccc1Nc1ccnc2cc(I)ccc12	0	GAK	GAK	=	340
UNC10225189A	Fc1cc(Cl)ccc1Nc1ccnc2cc(I)ccc12	0	MST1R	MST1R	>	10000
UNC10225189A	Fc1cc(Cl)ccc1Nc1ccnc2cc(I)ccc12	0	PDGFRB	PDGFRB	=	410
UNC10225189A	Fc1cc(Cl)ccc1Nc1ccnc2cc(I)ccc12	0	RSK4(Kin.Dc	RPS6KA6	=	730
UNC10225192A	FC(F)(F)c1cc(Nc2nccc(n2)-c2c(nn3ncccc23)-c2ccc(Cl)cc2)ccc1Cl	0	CSNK1D	CSNK1D	=	2200
UNC10225193A	COc1ccc(Nc2nccc(NCCNC(=O)Nc3cc(C)ccc3C)n2)cc1	3	CHEK2	CHEK2	>	10000
UNC10225193A	COc1ccc(Nc2nccc(NCCNC(=O)Nc3cc(C)ccc3C)n2)cc1	3	PIK3C2B	PIK3C2B	>	10000
UNC10225193A	COc1ccc(Nc2nccc(NCCNC(=O)Nc3cc(C)ccc3C)n2)cc1	3	PIK3CA	PIK3CA	>	10000
UNC10225195A	Cc1c(Cn2c3ccccc3n3c2nc(cc3=O)N2CCOCC2)cccc1C(F)(F)F	7	CDC2L1	CDK11B	>	10000
UNC10225195A	Cc1c(Cn2c3ccccc3n3c2nc(cc3=O)N2CCOCC2)cccc1C(F)(F)F	7	CDK9	CDK9	>	10000
UNC10225195A	Cc1c(Cn2c3ccccc3n3c2nc(cc3=O)N2CCOCC2)cccc1C(F)(F)F	7	ERK4	MAPK4	>	10000
UNC10225195A	Cc1c(Cn2c3ccccc3n3c2nc(cc3=O)N2CCOCC2)cccc1C(F)(F)F	7	PIK3CB	PIK3CB	=	680
UNC10225195A	Cc1c(Cn2c3ccccc3n3c2nc(cc3=O)N2CCOCC2)cccc1C(F)(F)F	7	PIK4CB	PI4KB	>	10000
UNC10225195A	Cc1c(Cn2c3ccccc3n3c2nc(cc3=O)N2CCOCC2)cccc1C(F)(F)F	7	PKN1	PKN1	>	10000
UNC10225195A	Cc1c(Cn2c3ccccc3n3c2nc(cc3=O)N2CCOCC2)cccc1C(F)(F)F	7	VPS34	PIK3C3	>	10000
UNC10225199A	Cc1ccc(C)c(NC(=O)NCCNc2ccnc(Nc3ccc(F)cc3)n2)c1	4	CSK	CSK	>	10000
UNC10225199A	Cc1ccc(C)c(NC(=O)NCCNc2ccnc(Nc3ccc(F)cc3)n2)c1	4	ERK5	MAPK7	>	10000
UNC10225199A	Cc1ccc(C)c(NC(=O)NCCNc2ccnc(Nc3ccc(F)cc3)n2)c1	4	PAK6	PAK6	>	10000
UNC10225199A	Cc1ccc(C)c(NC(=O)NCCNc2ccnc(Nc3ccc(F)cc3)n2)c1	4	STK16	STK16	>	10000
UNC10225200A	COc1cccc(NC(=O)Nc2ccc(C)c(c2)-c2ccc(cc2)C(=O)Nc2ccncc2)c1	15	BRSK2	BRSK2	>	10000
UNC10225200A	COc1cccc(NC(=O)Nc2ccc(C)c(c2)-c2ccc(cc2)C(=O)Nc2ccncc2)c1	15	CIT	CIT	=	280
UNC10225200A	COc1cccc(NC(=O)Nc2ccc(C)c(c2)-c2ccc(cc2)C(=O)Nc2ccncc2)c1	15	CSF1R	CSF1R	=	3300
UNC10225200A	COc1cccc(NC(=O)Nc2ccc(C)c(c2)-c2ccc(cc2)C(=O)Nc2ccncc2)c1	15	DDR1	DDR1	=	270
UNC10225200A	COc1cccc(NC(=O)Nc2ccc(C)c(c2)-c2ccc(cc2)C(=O)Nc2ccncc2)c1	15	IKK-beta	IKBKB	>	10000
UNC10225200A	COc1cccc(NC(=O)Nc2ccc(C)c(c2)-c2ccc(cc2)C(=O)Nc2ccncc2)c1	15	KIT	KIT	=	73
UNC10225200A	COc1cccc(NC(=O)Nc2ccc(C)c(c2)-c2ccc(cc2)C(=O)Nc2ccncc2)c1	15	LYN	LYN	=	4400
UNC10225200A	COc1cccc(NC(=O)Nc2ccc(C)c(c2)-c2ccc(cc2)C(=O)Nc2ccncc2)c1	15	MAK	MAK	>	10000
UNC10225200A	COc1cccc(NC(=O)Nc2ccc(C)c(c2)-c2ccc(cc2)C(=O)Nc2ccncc2)c1	15	MARK1	MARK1	>	10000
UNC10225200A	COc1cccc(NC(=O)Nc2ccc(C)c(c2)-c2ccc(cc2)C(=O)Nc2ccncc2)c1	15	PDGFRB	PDGFRB	=	40
UNC10225200A	COc1cccc(NC(=O)Nc2ccc(C)c(c2)-c2ccc(cc2)C(=O)Nc2ccncc2)c1	15	PIK3C2B	PIK3C2B	>	10000
UNC10225200A	COc1cccc(NC(=O)Nc2ccc(C)c(c2)-c2ccc(cc2)C(=O)Nc2ccncc2)c1	15	PIK3CA	PIK3CA	>	10000

UNC10225200A	COc1cccc(NC(=O)Nc2ccc(C)c(c2)-c2ccc(cc2)C(=O)Nc2ccncc2)c1	15	PIK3CG	PIK3CG	>	10000
UNC10225201A	Nc1nc(c(s1)-c1ccnc2ccccc12)-c1ccccn1	0	CSNK1A1	CSNK1A1	=	1200
UNC10225201A	Nc1nc(c(s1)-c1ccnc2ccccc12)-c1ccccn1	0	CSNK1D	CSNK1D	=	1800
UNC10225201A	Nc1nc(c(s1)-c1ccnc2ccccc12)-c1ccccn1	0	PIP5K1C	PIP5K1C	>	10000
UNC10225201A	Nc1nc(c(s1)-c1ccnc2ccccc12)-c1ccccn1	0	WNK4	WNK4	>	10000
UNC10225202A	Cc1cccc(Cn2ccn3c2nc(cc3=O)N2CCOCC2)c1	2	PIK3CB	PIK3CB	=	23
UNC10225202A	Cc1cccc(Cn2ccn3c2nc(cc3=O)N2CCOCC2)c1	2	VPS34	PIK3C3	=	75
UNC10225203A	Nc1ncnc2n(cnc12)-c1ccc(Cl)cc1	0	NIK	MAP3K14	>	10000
UNC10225203A	Nc1ncnc2n(cnc12)-c1ccc(Cl)cc1	0	PDGFRB	PDGFRB	=	1200
UNC10225204A	CCn1c(nc2cnccc12)-c1ccncc1	3	EPHA3	EPHA3	>	10000
UNC10225204A	CCn1c(nc2cnccc12)-c1ccncc1	3	MARK2	MARK2	>	10000
UNC10225204A	CCn1c(nc2cnccc12)-c1ccncc1	3	MARK3	MARK3	>	10000
UNC10225205A	CCc1nc(c(s1)-c1ccnc(Nc2ccc(nc2)N2CCN(CC2)C(C)=O)n1)-c1cccc(c1)N(C)S(=O)(=O)c1ccccc1	7	EPHB6	EPHB6	=	24
UNC10225205A	CCc1nc(c(s1)-c1ccnc(Nc2ccc(nc2)N2CCN(CC2)C(C)=O)n1)-c1cccc(c1)N(C)S(=O)(=O)c1ccccc1	7	GAK	GAK	=	250
UNC10225205A	CCc1nc(c(s1)-c1ccnc(Nc2ccc(nc2)N2CCN(CC2)C(C)=O)n1)-c1cccc(c1)N(C)S(=O)(=O)c1ccccc1	7	MEK5	MAP2K5	=	7
UNC10225205A	CCc1nc(c(s1)-c1ccnc(Nc2ccc(nc2)N2CCN(CC2)C(C)=O)n1)-c1cccc(c1)N(C)S(=O)(=O)c1ccccc1	7	RIPK2	RIPK2	=	130
UNC10225205A	CCc1nc(c(s1)-c1ccnc(Nc2ccc(nc2)N2CCN(CC2)C(C)=O)n1)-c1cccc(c1)N(C)S(=O)(=O)c1ccccc1	7	STK36	STK36	=	56
UNC10225205A	CCc1nc(c(s1)-c1ccnc(Nc2ccc(nc2)N2CCN(CC2)C(C)=O)n1)-c1cccc(c1)N(C)S(=O)(=O)c1ccccc1	7	ZAK	ZAK	=	220
UNC10225209A	Nc1n[nH]c2nnc(cc12)-c1ccccc1	2	DYRK1B	DYRK1B	=	7000
UNC10225209A	Nc1n[nH]c2nnc(cc12)-c1ccccc1	2	EPHB2	EPHB2	>	10000
UNC10225209A	Nc1n[nH]c2nnc(cc12)-c1ccccc1	2	HIPK3	HIPK3	=	290
UNC10225209A	Nc1n[nH]c2nnc(cc12)-c1ccccc1	2	MLCK	MYLK3	>	10000
UNC10225209A	Nc1n[nH]c2nnc(cc12)-c1ccccc1	2	RET	RET	>	10000
UNC10225209A	Nc1n[nH]c2nnc(cc12)-c1ccccc1	2	TSSK3	TSSK3	>	10000
UNC10225210A	Fc1ccc(Nc2nccc(NCCNC(=O)Nc3ccccc3F)n2)cc1	1	PLK1	PLK1	>	10000
UNC10225212A	CN1C(=O)C(Nc2cc(Cl)c(O)c(Cl)c2)=C(C1=O)c1ccc(Cl)cc1	3	GRK4	GRK4	>	10000
UNC10225212A	CN1C(=O)C(Nc2cc(Cl)c(O)c(Cl)c2)=C(C1=O)c1ccc(Cl)cc1	3	MAPKAPK2	MAPKAPK2	>	10000
UNC10225212A	CN1C(=O)C(Nc2cc(Cl)c(O)c(Cl)c2)=C(C1=O)c1ccc(Cl)cc1	3	SRPK3	SRPK3	>	10000
UNC10225212A	CN1C(=O)C(Nc2cc(Cl)c(O)c(Cl)c2)=C(C1=O)c1ccc(Cl)cc1	3	VRK2	VRK2	>	10000
UNC10225213A	COc1nccc(n1)-c1c(ncn1CCCN1CCOCC1)-c1ccc(F)cc1	3	CDK4	CDK4	>	10000
UNC10225213A	COc1nccc(n1)-c1c(ncn1CCCN1CCOCC1)-c1ccc(F)cc1	3	p38-alpha	MAPK14	=	160
UNC10225220A	Fc1cc(Cl)ccc1Nc1ccnc2cc(ccc12)-c1ccc(CN2CCCCC2)o1	8	AURKA	AURKA	>	10000
UNC10225220A	Fc1cc(Cl)ccc1Nc1ccnc2cc(ccc12)-c1ccc(CN2CCCCC2)o1	8	CSNK2A2	CSNK2A2	>	10000
UNC10225220A	Fc1cc(Cl)ccc1Nc1ccnc2cc(ccc12)-c1ccc(CN2CCCCC2)o1	8	MEK2	MAP2K2	>	10000
UNC10225220A	Fc1cc(Cl)ccc1Nc1ccnc2cc(ccc12)-c1ccc(CN2CCCCC2)o1	8	MEK5	MAP2K5	=	890
UNC10225220A	Fc1cc(Cl)ccc1Nc1ccnc2cc(ccc12)-c1ccc(CN2CCCCC2)o1	8	PDGFRB	PDGFRB	=	210
UNC10225220A	Fc1cc(Cl)ccc1Nc1ccnc2cc(ccc12)-c1ccc(CN2CCCCC2)o1	8	PDPK1	PDPK1	>	10000
UNC10225220A	Fc1cc(Cl)ccc1Nc1ccnc2cc(ccc12)-c1ccc(CN2CCCCC2)o1	8	PKAC-alpha	PRKACA	>	10000
UNC10225220A	Fc1cc(Cl)ccc1Nc1ccnc2cc(ccc12)-c1ccc(CN2CCCCC2)o1	8	RSK4(Kin.Dc	RPS6KA6	=	670
UNC10225220A	Fc1cc(Cl)ccc1Nc1ccnc2cc(ccc12)-c1ccc(CN2CCCCC2)o1	8	SRC	SRC	=	4400
UNC10225222A	OC(=O)C(F)(F)F.Cc1cc(nc(Nc2cccc(c2)C#N)n1)N1CCC(CC1)NS(=O)(=O)c1ccc(F)cc1	1	DYRK1B	DYRK1B	=	2800
UNC10225225A	Cc1c(C)n2c(nc(cc2=O)N2CCOCC2)n1Cc1cccc(c1C)C(F)(F)F	5	CDKL1	CDKL1	>	10000
UNC10225225A	Cc1c(C)n2c(nc(cc2=O)N2CCOCC2)n1Cc1cccc(c1C)C(F)(F)F	5	LATS1	LATS1	>	10000
UNC10225225A	Cc1c(C)n2c(nc(cc2=O)N2CCOCC2)n1Cc1cccc(c1C)C(F)(F)F	5	PIK3CB	PIK3CB	=	16
UNC10225225A	Cc1c(C)n2c(nc(cc2=O)N2CCOCC2)n1Cc1cccc(c1C)C(F)(F)F	5	PIK3CD	PIK3CD	=	620
UNC10225225A	Cc1c(C)n2c(nc(cc2=O)N2CCOCC2)n1Cc1cccc(c1C)C(F)(F)F	5	PLK2	PLK2	>	10000
UNC10225225A	Cc1c(C)n2c(nc(cc2=O)N2CCOCC2)n1Cc1cccc(c1C)C(F)(F)F	5	PRKG2	PRKG2	>	10000

UNC10225227A	OC(=O)C(F)(F)F.OC(=O)C(F)(F)F.OC(=O)C(F)(F)F.Cc1cc(C)cc(Oc2nccc(n2)-c2c(ncn2C2CCNCC2)-c2ccc(F)cc2)c1	4	CIT	CIT	=	410
UNC10225227A	OC(=O)C(F)(F)F.OC(=O)C(F)(F)F.OC(=O)C(F)(F)F.Cc1cc(C)cc(Oc2nccc(n2)-c2c(ncn2C2CCNCC2)-c2ccc(F)cc2)c1	4	CSNK1D	CSNK1D	=	160
UNC10225227A	OC(=O)C(F)(F)F.OC(=O)C(F)(F)F.OC(=O)C(F)(F)F.Cc1cc(C)cc(Oc2nccc(n2)-c2c(ncn2C2CCNCC2)-c2ccc(F)cc2)c1	4	ERBB2	ERBB2	=	250
UNC10225227A	OC(=O)C(F)(F)F.OC(=O)C(F)(F)F.OC(=O)C(F)(F)F.Cc1cc(C)cc(Oc2nccc(n2)-c2c(ncn2C2CCNCC2)-c2ccc(F)cc2)c1	4	JAK1(JH1do	JAK1	>	10000
UNC10225227A	OC(=O)C(F)(F)F.OC(=O)C(F)(F)F.OC(=O)C(F)(F)F.Cc1cc(C)cc(Oc2nccc(n2)-c2c(ncn2C2CCNCC2)-c2ccc(F)cc2)c1	4	p38-alpha	MAPK14	=	38
UNC10225227A	OC(=O)C(F)(F)F.OC(=O)C(F)(F)F.OC(=O)C(F)(F)F.Cc1cc(C)cc(Oc2nccc(n2)-c2c(ncn2C2CCNCC2)-c2ccc(F)cc2)c1	4	PIM1	PIM1	>	10000
UNC10225227A	OC(=O)C(F)(F)F.OC(=O)C(F)(F)F.OC(=O)C(F)(F)F.Cc1cc(C)cc(Oc2nccc(n2)-c2c(ncn2C2CCNCC2)-c2ccc(F)cc2)c1	4	PRKCI	PRKCI	>	10000
UNC10225227A	OC(=O)C(F)(F)F.OC(=O)C(F)(F)F.OC(=O)C(F)(F)F.Cc1cc(C)cc(Oc2nccc(n2)-c2c(ncn2C2CCNCC2)-c2ccc(F)cc2)c1	4	YANK2	STK32B	=	160
UNC10225229A	COCCNC(=O)c1ccc(cc1)-c1cc(cc(F)c1C)C(=O)NC1CC1	2	p38-alpha	MAPK14	=	7.3
UNC10225229A	COCCNC(=O)c1ccc(cc1)-c1cc(cc(F)c1C)C(=O)NC1CC1	2	p38-beta	MAPK11	=	88
UNC10225230A	O=C(N1CCCC1)c1ccc(cc1)-c1cc(ccn1)-c1c[nH]nc1-c1ccccn1	4	DAPK3	DAPK3	>	10000
UNC10225230A	O=C(N1CCCC1)c1ccc(cc1)-c1cc(ccn1)-c1c[nH]nc1-c1ccccn1	4	PIM1	PIM1	>	10000
UNC10225230A	O=C(N1CCCC1)c1ccc(cc1)-c1cc(ccn1)-c1c[nH]nc1-c1ccccn1	4	PIP5K2C	PIP4K2C	=	21
UNC10225230A	O=C(N1CCCC1)c1ccc(cc1)-c1cc(ccn1)-c1c[nH]nc1-c1ccccn1	4	PRKCI	PRKCI	>	10000
UNC10225230A	O=C(N1CCCC1)c1ccc(cc1)-c1cc(ccn1)-c1c[nH]nc1-c1ccccn1	4	TGFBR1	TGFBR1	=	170
UNC10225231A	Fc1ccccc1Cn1ccn2c1nc(cc2=O)N1CCOCC1	4	BRSK2	BRSK2	>	10000
UNC10225231A	Fc1ccccc1Cn1ccn2c1nc(cc2=O)N1CCOCC1	4	GSK3A	GSK3A	>	10000
UNC10225231A	Fc1ccccc1Cn1ccn2c1nc(cc2=O)N1CCOCC1	4	MARK1	MARK1	>	10000
UNC10225231A	Fc1ccccc1Cn1ccn2c1nc(cc2=O)N1CCOCC1	4	PIK3CB	PIK3CB	=	180
UNC10225231A	Fc1ccccc1Cn1ccn2c1nc(cc2=O)N1CCOCC1	4	VPS34	PIK3C3	=	68
UNC10225233A	Cl.COc1cc2nccc(Nc3ccc(OCc4ccc(NC(C)=O)cc4)cc3)c2cc1OC	10	FLT1	FLT1	=	680
UNC10225233A	Cl.COc1cc2nccc(Nc3ccc(OCc4ccc(NC(C)=O)cc4)cc3)c2cc1OC	10	FLT4	FLT4	=	480
UNC10225233A	Cl.COc1cc2nccc(Nc3ccc(OCc4ccc(NC(C)=O)cc4)cc3)c2cc1OC	10	KIT	KIT	=	7.4
UNC10225233A	Cl.COc1cc2nccc(Nc3ccc(OCc4ccc(NC(C)=O)cc4)cc3)c2cc1OC	10	MEK5	MAP2K5	=	52
UNC10225233A	Cl.COc1cc2nccc(Nc3ccc(OCc4ccc(NC(C)=O)cc4)cc3)c2cc1OC	10	NEK7	NEK7	>	10000
UNC10225233A	Cl.COc1cc2nccc(Nc3ccc(OCc4ccc(NC(C)=O)cc4)cc3)c2cc1OC	10	NEK9	NEK9	>	10000
UNC10225233A	Cl.COc1cc2nccc(Nc3ccc(OCc4ccc(NC(C)=O)cc4)cc3)c2cc1OC	10	PDGFRA	PDGFRA	=	60
UNC10225233A	Cl.COc1cc2nccc(Nc3ccc(OCc4ccc(NC(C)=O)cc4)cc3)c2cc1OC	10	PDGFRB	PDGFRB	=	190
UNC10225233A	Cl.COc1cc2nccc(Nc3ccc(OCc4ccc(NC(C)=O)cc4)cc3)c2cc1OC	10	VEGFR2	KDR	=	53
UNC10225235A	Fc1ccc(Nc2nccc(n2)N2CCC(C2)NC(=O)Nc2ccc3OCOc3c2)cc1	2	AURKB	AURKB	=	6900
UNC10225235A	Fc1ccc(Nc2nccc(n2)N2CCC(C2)NC(=O)Nc2ccc3OCOc3c2)cc1	2	GRK3	ADRBK2	>	10000

UNC10225238A	Cl.Cc1cnc(N)c(\C=C\c2cncc(c2)C(=O)NCCc2ccc(Cl)cc2Cl)c1	6	CDK11	CDK19	=	410
UNC10225238A	Cl.Cc1cnc(N)c(\C=C\c2cncc(c2)C(=O)NCCc2ccc(Cl)cc2Cl)c1	6	CDK8	CDK8	=	1100
UNC10225238A	Cl.Cc1cnc(N)c(\C=C\c2cncc(c2)C(=O)NCCc2ccc(Cl)cc2Cl)c1	6	KIT	KIT	=	150
UNC10225238A	Cl.Cc1cnc(N)c(\C=C\c2cncc(c2)C(=O)NCCc2ccc(Cl)cc2Cl)c1	6	PDGFRA	PDGFRA	=	39
UNC10225238A	Cl.Cc1cnc(N)c(\C=C\c2cncc(c2)C(=O)NCCc2ccc(Cl)cc2Cl)c1	6	PDGFRB	PDGFRB	=	140
UNC10225239A	Cc1cccc(n1)-c1nc(Nc2ccc3[nH]nnc3c2)c2ccccc2n1	2	CSNK2A1	CSNK2A1	=	770
UNC10225239A	Cc1cccc(n1)-c1nc(Nc2ccc3[nH]nnc3c2)c2ccccc2n1	2	PRKD1	PRKD1	=	61
UNC10225239A	Cc1cccc(n1)-c1nc(Nc2ccc3[nH]nnc3c2)c2ccccc2n1	2	PRKD2	PRKD2	=	83
UNC10225240A	COC(=O)C(Cc1ccccc1)NC(=O)NC1CCN(C1)c1ccnc(Nc2ccc(F)cc2)n1	3	CHEK1	CHEK1	>	10000
UNC10225240A	COC(=O)C(Cc1ccccc1)NC(=O)NC1CCN(C1)c1ccnc(Nc2ccc(F)cc2)n1	3	GRK1	GRK1	>	10000
UNC10225240A	COC(=O)C(Cc1ccccc1)NC(=O)NC1CCN(C1)c1ccnc(Nc2ccc(F)cc2)n1	3	PRKD3	PRKD3	>	10000
UNC10225240A	COC(=O)C(Cc1ccccc1)NC(=O)NC1CCN(C1)c1ccnc(Nc2ccc(F)cc2)n1	3	TRKA	NTRK1	=	1500
UNC10225240A	COC(=O)C(Cc1ccccc1)NC(=O)NC1CCN(C1)c1ccnc(Nc2ccc(F)cc2)n1	3	YSK4	MAP3K19	=	1400
UNC10225241A	FC(F)(F)c1ccc(cc1)-c1cc(ccn1)-c1c[nH]nc1-c1ccccn1	4	DAPK3	DAPK3	>	10000
UNC10225241A	FC(F)(F)c1ccc(cc1)-c1cc(ccn1)-c1c[nH]nc1-c1ccccn1	4	EIF2AK1	EIF2AK1	>	10000
UNC10225241A	FC(F)(F)c1ccc(cc1)-c1cc(ccn1)-c1c[nH]nc1-c1ccccn1	4	SNRK	SNRK	>	10000
UNC10225241A	FC(F)(F)c1ccc(cc1)-c1cc(ccn1)-c1c[nH]nc1-c1ccccn1	4	TGFBR1	TGFBR1	=	220
UNC10225241A	FC(F)(F)c1ccc(cc1)-c1cc(ccn1)-c1c[nH]nc1-c1ccccn1	4	YANK1	STK32A	>	10000
UNC10225242A	CN1C(=O)C(Nc2ccccc2)=C(C1=O)c1ccccc1	1	MAST1	MAST1	>	10000
UNC10225243A	Cc1ccccc1-c1cc(ccn1)-c1c[nH]nc1-c1ccccn1	1	CHEK1	CHEK1	>	10000
UNC10225243A	Cc1ccccc1-c1cc(ccn1)-c1c[nH]nc1-c1ccccn1	1	p38-alpha	MAPK14	=	130
UNC10225243A	Cc1ccccc1-c1cc(ccn1)-c1c[nH]nc1-c1ccccn1	1	PRKD3	PRKD3	>	10000
UNC10225244A	COc1ccc(cc1)C1=C(Nc2cccc(c2)C(O)=O)C(=O)NC1=O	2	ACVR1	ACVR1	>	10000
UNC10225244A	COc1ccc(cc1)C1=C(Nc2cccc(c2)C(O)=O)C(=O)NC1=O	2	LIMK1	LIMK1	>	10000
UNC10225245A	OC(=O)C(F)(F)F.COc1ccc(cc1)S(=O)(=O)NC1CCN(CC1)c1cc(C)nc(Nc2cccc(c2)C#N)n1	3	PIK3CB	PIK3CB	>	10000
UNC10225247A	CCCCc1nn2ncccc2c1-c1ccnc(NC2CC2)n1	4	BUB1	BUB1	=	3300
UNC10225247A	CCCCc1nn2ncccc2c1-c1ccnc(NC2CC2)n1	4	CLK3	CLK3	>	10000
UNC10225247A	CCCCc1nn2ncccc2c1-c1ccnc(NC2CC2)n1	4	CSNK1D	CSNK1D	=	480
UNC10225247A	CCCCc1nn2ncccc2c1-c1ccnc(NC2CC2)n1	4	CSNK1E	CSNK1E	=	2900
UNC10225247A	CCCCc1nn2ncccc2c1-c1ccnc(NC2CC2)n1	4	JNK2	MAPK9	=	1300
UNC10225247A	CCCCc1nn2ncccc2c1-c1ccnc(NC2CC2)n1	4	JNK3	MAPK10	=	740
UNC10225251A	Cl.C(N1CCOCC1)c1ccc(cc1)-c1cc(ccn1)-c1c[nH]nc1- c1ccccn1	1	ACVR1B	ACVR1B	=	180
UNC10225251A	Cl.C(N1CCOCC1)c1ccc(cc1)-c1cc(ccn1)-c1c[nH]nc1- c1ccccn1	1	TGFBR1	TGFBR1	=	150
UNC10225251A	Cl.C(N1CCOCC1)c1ccc(cc1)-c1cc(ccn1)-c1c[nH]nc1- c1ccccn1	1	TGFBR2	TGFBR2	=	210
UNC10225252A	Oc1ccc(NC2=C(C(=O)NC2=O)c2ccc(Cl)cc2)cc1Cl	0	PCTK2	CDK17	>	10000
UNC10225255A	Clc1ccccc1C1=C(N2CCc3ccccc23)C(=O)NC1=O	10	ABL1-phosp	ABL1	>	10000
UNC10225255A	Clc1ccccc1C1=C(N2CCc3ccccc23)C(=O)NC1=O	10	BMPR2	BMPR2	>	10000
UNC10225255A	Clc1ccccc1C1=C(N2CCc3ccccc23)C(=O)NC1=O	10	HASPIN	GSG2	>	10000
UNC10225255A	Clc1ccccc1C1=C(N2CCc3ccccc23)C(=O)NC1=O	10	IKK-alpha	CHUK	>	10000
UNC10225255A	Clc1ccccc1C1=C(N2CCc3ccccc23)C(=O)NC1=O	10	LTK	LTK	>	10000
UNC10225255A	Clc1ccccc1C1=C(N2CCc3ccccc23)C(=O)NC1=O	10	MST1	STK4	>	10000

UNC10225255A	Clc1ccccc1C1=C(N2CCc3ccccc23)C(=O)NC1=O	10	PAK3	PAK3	>	10000
UNC10225255A	Clc1ccccc1C1=C(N2CCc3ccccc23)C(=O)NC1=O	10	SRMS	SRMS	>	10000
UNC10225255A	Clc1ccccc1C1=C(N2CCc3ccccc23)C(=O)NC1=O	10	TAOK2	TAOK2	>	10000
UNC10225256A	Cl.Cl.CN(C)CCN(C)C(=O)c1cc(c(o1)-c1ccc(Cl)c(O)c1)-c1ccncc1	5	BRAF	BRAF	=	45
UNC10225256A	Cl.Cl.CN(C)CCN(C)C(=O)c1cc(c(o1)-c1ccc(Cl)c(O)c1)-c1ccncc1	5	BUB1	BUB1	=	390
UNC10225256A	Cl.Cl.CN(C)CCN(C)C(=O)c1cc(c(o1)-c1ccc(Cl)c(O)c1)-c1ccncc1	5	CSNK1D	CSNK1D	=	220
UNC10225256A	Cl.Cl.CN(C)CCN(C)C(=O)c1cc(c(o1)-c1ccc(Cl)c(O)c1)-c1ccncc1	5	CSNK1E	CSNK1E	=	410
UNC10225256A	Cl.Cl.CN(C)CCN(C)C(=O)c1cc(c(o1)-c1ccc(Cl)c(O)c1)-c1ccncc1	5	p38-alpha	MAPK14	=	740
UNC10225256A	Cl.Cl.CN(C)CCN(C)C(=O)c1cc(c(o1)-c1ccc(Cl)c(O)c1)-c1ccncc1	5	RAF1	RAF1	=	650
UNC10225256A	Cl.Cl.CN(C)CCN(C)C(=O)c1cc(c(o1)-c1ccc(Cl)c(O)c1)-c1ccncc1	5	SLK	SLK	=	3600
UNC10225258A	CC(C)N1CCC(CC1)n1cnc(c1-c1ccnc(N)n1)-c1ccc(F)cc1	5	CSNK1A1	CSNK1A1	=	59
UNC10225258A	CC(C)N1CCC(CC1)n1cnc(c1-c1ccnc(N)n1)-c1ccc(F)cc1	5	CSNK1D	CSNK1D	=	39
UNC10225258A	CC(C)N1CCC(CC1)n1cnc(c1-c1ccnc(N)n1)-c1ccc(F)cc1	5	CSNK1E	CSNK1E	=	230
UNC10225258A	CC(C)N1CCC(CC1)n1cnc(c1-c1ccnc(N)n1)-c1ccc(F)cc1	5	p38-alpha	MAPK14	=	160
UNC10225258A	CC(C)N1CCC(CC1)n1cnc(c1-c1ccnc(N)n1)-c1ccc(F)cc1	5	TIE2	TEK	>	10000
UNC10225259A	Brc1cc2c(NC(=O)C3CC3)n[nH]c2nc1-c1ccccc1	6	ERK5	MAPK7	>	10000
UNC10225259A	Brc1cc2c(NC(=O)C3CC3)n[nH]c2nc1-c1ccccc1	6	GSK3A	GSK3A	=	410
UNC10225259A	Brc1cc2c(NC(=O)C3CC3)n[nH]c2nc1-c1ccccc1	6	MST1R	MST1R	>	10000
UNC10225259A	Brc1cc2c(NC(=O)C3CC3)n[nH]c2nc1-c1ccccc1	6	MYLK2	MYLK2	=	60
UNC10225259A	Brc1cc2c(NC(=O)C3CC3)n[nH]c2nc1-c1ccccc1	6	PDGFRB	PDGFRB	=	230
UNC10225259A	Brc1cc2c(NC(=O)C3CC3)n[nH]c2nc1-c1ccccc1	6	YSK4	MAP3K19	=	640
UNC10225260A	NC(=O)c1sc(cc1OCc1ccccc1F)-n1cnc2ccccc12	9	EGFR	EGFR	=	1300
UNC10225260A	NC(=O)c1sc(cc1OCc1ccccc1F)-n1cnc2ccccc12	9	p38-alpha	MAPK14	=	280
UNC10225260A	NC(=O)c1sc(cc1OCc1ccccc1F)-n1cnc2ccccc12	9	PLK1	PLK1	=	9.6
UNC10225260A	NC(=O)c1sc(cc1OCc1ccccc1F)-n1cnc2ccccc12	9	PLK2	PLK2	=	25
UNC10225260A	NC(=O)c1sc(cc1OCc1ccccc1F)-n1cnc2ccccc12	9	PLK3	PLK3	=	52
UNC10225260A	NC(=O)c1sc(cc1OCc1ccccc1F)-n1cnc2ccccc12	9	ROCK1	ROCK1	=	460
UNC10225260A	NC(=O)c1sc(cc1OCc1ccccc1F)-n1cnc2ccccc12	9	ROCK2	ROCK2	=	470
UNC10225260A	NC(=O)c1sc(cc1OCc1ccccc1F)-n1cnc2ccccc12	9	RSK4(Kin.Dc	RPS6KA6	=	670
UNC10225261A	Cl.COc1cc2ncnc(Nc3ccc(OCc4ccccc4Br)c(Cl)c3)c2cc1OC	17	EGFR	EGFR	=	2.4
UNC10225261A	Cl.COc1cc2ncnc(Nc3ccc(OCc4ccccc4Br)c(Cl)c3)c2cc1OC	17	ERBB2	ERBB2	=	17
UNC10225261A	Cl.COc1cc2ncnc(Nc3ccc(OCc4ccccc4Br)c(Cl)c3)c2cc1OC	17	ERBB4	ERBB4	=	27
UNC10225261A	Cl.COc1cc2ncnc(Nc3ccc(OCc4ccccc4Br)c(Cl)c3)c2cc1OC	17	LOK	STK10	=	2000
UNC10225261A	Cl.COc1cc2ncnc(Nc3ccc(OCc4ccccc4Br)c(Cl)c3)c2cc1OC	17	MEK5	MAP2K5	=	98
UNC10225261A	Cl.COc1cc2ncnc(Nc3ccc(OCc4ccccc4Br)c(Cl)c3)c2cc1OC	17	PKN1	PKN1	>	10000
UNC10225261A	Cl.COc1cc2ncnc(Nc3ccc(OCc4ccccc4Br)c(Cl)c3)c2cc1OC	17	PRKCQ	PRKCQ	>	10000
UNC10225261A	Cl.COc1cc2ncnc(Nc3ccc(OCc4ccccc4Br)c(Cl)c3)c2cc1OC	17	RIPK2	RIPK2	=	410
UNC10225261A	Cl.COc1cc2ncnc(Nc3ccc(OCc4ccccc4Br)c(Cl)c3)c2cc1OC	17	RSK3(Kin.Dc	RPS6KA2	>	10000
UNC10225261A	Cl.COc1cc2ncnc(Nc3ccc(OCc4ccccc4Br)c(Cl)c3)c2cc1OC	17	SLK	SLK	=	9300
UNC10225263A	Cc1ccc(NC(=O)c2cccc(c2)C#N)cc1-c1ccc(cc1)C(=O)Nc1ccncc1	8	CSF1R	CSF1R	=	780
UNC10225263A	Cc1ccc(NC(=O)c2cccc(c2)C#N)cc1-c1ccc(cc1)C(=O)Nc1ccncc1	8	DDR1	DDR1	=	22
UNC10225263A	Cc1ccc(NC(=O)c2cccc(c2)C#N)cc1-c1ccc(cc1)C(=O)Nc1ccncc1	8	DDR2	DDR2	=	140
UNC10225263A	Cc1ccc(NC(=O)c2cccc(c2)C#N)cc1-c1ccc(cc1)C(=O)Nc1ccncc1	8	ERK5	MAPK7	>	10000

UNC10225263A	Cc1ccc(NC(=O)c2cccc(c2)C#N)cc1-c1ccc(cc1)C(=O)Nc1ccncc1	8	FRK	FRK	=	190
UNC10225263A	Cc1ccc(NC(=O)c2cccc(c2)C#N)cc1-c1ccc(cc1)C(=O)Nc1ccncc1	8	KIT	KIT	=	310
UNC10225263A	Cc1ccc(NC(=O)c2cccc(c2)C#N)cc1-c1ccc(cc1)C(=O)Nc1ccncc1	8	LCK	LCK	=	560
UNC10225263A	Cc1ccc(NC(=O)c2cccc(c2)C#N)cc1-c1ccc(cc1)C(=O)Nc1ccncc1	8	PDGFRA	PDGFRA	=	170
UNC10225263A	Cc1ccc(NC(=O)c2cccc(c2)C#N)cc1-c1ccc(cc1)C(=O)Nc1ccncc1	8	PDGFRB	PDGFRB	=	13
UNC10225267A	COc1cccc(c1)C1=C(Nc2cc(Cl)c(O)c(Cl)c2)C(=O)NC1=O	10	CHEK1	CHEK1	>	10000
UNC10225267A	COc1cccc(c1)C1=C(Nc2cc(Cl)c(O)c(Cl)c2)C(=O)NC1=O	10	CSNK1G1	CSNK1G1	>	10000
UNC10225267A	COc1cccc(c1)C1=C(Nc2cc(Cl)c(O)c(Cl)c2)C(=O)NC1=O	10	DDR1	DDR1	>	10000
UNC10225267A	COc1cccc(c1)C1=C(Nc2cc(Cl)c(O)c(Cl)c2)C(=O)NC1=O	10	GAK	GAK	>	10000
UNC10225267A	COc1cccc(c1)C1=C(Nc2cc(Cl)c(O)c(Cl)c2)C(=O)NC1=O	10	JAK3(JH1do	JAK3	=	120
UNC10225267A	COc1cccc(c1)C1=C(Nc2cc(Cl)c(O)c(Cl)c2)C(=O)NC1=O	10	LTK	LTK	>	10000
UNC10225267A	COc1cccc(c1)C1=C(Nc2cc(Cl)c(O)c(Cl)c2)C(=O)NC1=O	10	MST1	STK4	=	1800
UNC10225267A	COc1cccc(c1)C1=C(Nc2cc(Cl)c(O)c(Cl)c2)C(=O)NC1=O	10	NEK2	NEK2	>	10000
UNC10225267A	COc1cccc(c1)C1=C(Nc2cc(Cl)c(O)c(Cl)c2)C(=O)NC1=O	10	PAK3	PAK3	>	10000
UNC10225267A	COc1cccc(c1)C1=C(Nc2cc(Cl)c(O)c(Cl)c2)C(=O)NC1=O	10	PRKD3	PRKD3	>	10000
UNC10225268A	Cl.NC(=O)c1cccc(Nc2nccc(Nc3ccc(Oc4cccc(Cl)c4)c(Cl)c3)n2)c1	5	BLK	BLK	=	2600
UNC10225268A	Cl.NC(=O)c1cccc(Nc2nccc(Nc3ccc(Oc4cccc(Cl)c4)c(Cl)c3)n2)c1	5	EGFR	EGFR	=	120
UNC10225268A	Cl.NC(=O)c1cccc(Nc2nccc(Nc3ccc(Oc4cccc(Cl)c4)c(Cl)c3)n2)c1	5	ERBB3	ERBB3	=	260
UNC10225268A	Cl.NC(=O)c1cccc(Nc2nccc(Nc3ccc(Oc4cccc(Cl)c4)c(Cl)c3)n2)c1	5	ICK	ICK	>	10000
UNC10225268A	Cl.NC(=O)c1cccc(Nc2nccc(Nc3ccc(Oc4cccc(Cl)c4)c(Cl)c3)n2)c1	5	JAK2(JH1do	JAK2	=	400
UNC10225268A	Cl.NC(=O)c1cccc(Nc2nccc(Nc3ccc(Oc4cccc(Cl)c4)c(Cl)c3)n2)c1	5	MEK5	MAP2K5	=	34
UNC10225268A	Cl.NC(=O)c1cccc(Nc2nccc(Nc3ccc(Oc4cccc(Cl)c4)c(Cl)c3)n2)c1	5	PIK4CB	PI4KB	>	10000
UNC10225268A	Cl.NC(=O)c1cccc(Nc2nccc(Nc3ccc(Oc4cccc(Cl)c4)c(Cl)c3)n2)c1	5	RIPK2	RIPK2	=	44
UNC10225270A	Cc1ccc(cc1-c1ccc(cc1)C(=O)NCC1CC1)C(=O)NC1CCCC1	2	AMPK-alpha	PRKAA2	>	10000
UNC10225270A	Cc1ccc(cc1-c1ccc(cc1)C(=O)NCC1CC1)C(=O)NC1CCCC1	2	EGFR	EGFR	>	10000
UNC10225271A	COc1cc2c(Nc3cccc(NC(=O)c4ccccc4)c3)ncnc2cc1OCCCN1CCOCC1	9	CSF1R	CSF1R	=	420
UNC10225271A	COc1cc2c(Nc3cccc(NC(=O)c4ccccc4)c3)ncnc2cc1OCCCN1CCOCC1	9	EGFR	EGFR	=	1400
UNC10225271A	COc1cc2c(Nc3cccc(NC(=O)c4ccccc4)c3)ncnc2cc1OCCCN1CCOCC1	9	KIT	KIT	=	300
UNC10225271A	COc1cc2c(Nc3cccc(NC(=O)c4ccccc4)c3)ncnc2cc1OCCCN1CCOCC1	9	LCK	LCK	=	390
UNC10225271A	COc1cc2c(Nc3cccc(NC(=O)c4ccccc4)c3)ncnc2cc1OCCCN1CCOCC1	9	LYN	LYN	=	130
UNC10225271A	COc1cc2c(Nc3cccc(NC(=O)c4ccccc4)c3)ncnc2cc1OCCCN1CCOCC1	9	MEK5	MAP2K5	=	940
UNC10225271A	COc1cc2c(Nc3cccc(NC(=O)c4ccccc4)c3)ncnc2cc1OCCCN1CCOCC1	9	p38-alpha	MAPK14	=	440
UNC10225271A	COc1cc2c(Nc3cccc(NC(=O)c4ccccc4)c3)ncnc2cc1OCCCN1CCOCC1	9	p38-beta	MAPK11	=	100

UNC10225271A	COc1cc2c(Nc3cccc(NC(=O)c4ccccc4)c3)ncnc2cc1OCCCN1CCOCC1	9	PDGFRB	PDGFRB	=	560
UNC10225271A	COc1cc2c(Nc3cccc(NC(=O)c4ccccc4)c3)ncnc2cc1OCCCN1CCOCC1	9	PDPK1	PDPK1	>	10000
UNC10225271A	COc1cc2c(Nc3cccc(NC(=O)c4ccccc4)c3)ncnc2cc1OCCCN1CCOCC1	9	PKAC-alpha	PRKACA	>	10000
UNC10225271A	COc1cc2c(Nc3cccc(NC(=O)c4ccccc4)c3)ncnc2cc1OCCCN1CCOCC1	9	RET	RET	=	110
UNC10225271A	COc1cc2c(Nc3cccc(NC(=O)c4ccccc4)c3)ncnc2cc1OCCCN1CCOCC1	9	SRC	SRC	=	4800
UNC10225274A	CNc1nccc(n1)-c1c(ncn1C1CCN(C)CC1)-c1ccc(F)cc1	4	CSNK1A1	CSNK1A1	=	24
UNC10225274A	CNc1nccc(n1)-c1c(ncn1C1CCN(C)CC1)-c1ccc(F)cc1	4	CSNK1D	CSNK1D	=	5.7
UNC10225274A	CNc1nccc(n1)-c1c(ncn1C1CCN(C)CC1)-c1ccc(F)cc1	4	CSNK1E	CSNK1E	=	17
UNC10225274A	CNc1nccc(n1)-c1c(ncn1C1CCN(C)CC1)-c1ccc(F)cc1	4	GRK2	ADRBK1	>	10000
UNC10225274A	CNc1nccc(n1)-c1c(ncn1C1CCN(C)CC1)-c1ccc(F)cc1	4	NLK	NLK	=	130
UNC10225274A	CNc1nccc(n1)-c1c(ncn1C1CCN(C)CC1)-c1ccc(F)cc1	4	p38-alpha	MAPK14	=	53
UNC10225274A	CNc1nccc(n1)-c1c(ncn1C1CCN(C)CC1)-c1ccc(F)cc1	4	PIP5K2C	PIP4K2C	=	39
UNC10225278A	COc1ccc(Nc2nccc(n2)N2CCC(C2)NC(=O)c2cccc(NC(C)=O)c2)cc1	1	DYRK1B	DYRK1B	>	10000
UNC10225278A	COc1ccc(Nc2nccc(n2)N2CCC(C2)NC(=O)c2cccc(NC(C)=O)c2)cc1	1	WNK4	WNK4	>	10000
UNC10225279B	Cl.CS(=O)(=O)CCNCc1nc(cs1)-c1ccc2ncnc(Nc3ccc(OCc4ccccc4)c(Cl)c3)c2c1	17	CHEK1	CHEK1	>	10000
UNC10225279B	Cl.CS(=O)(=O)CCNCc1nc(cs1)-c1ccc2ncnc(Nc3ccc(OCc4ccccc4)c(Cl)c3)c2c1	17	DDR1	DDR1	>	10000
UNC10225279B	Cl.CS(=O)(=O)CCNCc1nc(cs1)-c1ccc2ncnc(Nc3ccc(OCc4ccccc4)c(Cl)c3)c2c1	17	EGFR	EGFR	=	1.4
UNC10225279B	Cl.CS(=O)(=O)CCNCc1nc(cs1)-c1ccc2ncnc(Nc3ccc(OCc4ccccc4)c(Cl)c3)c2c1	17	ERBB2	ERBB2	=	4
UNC10225279B	Cl.CS(=O)(=O)CCNCc1nc(cs1)-c1ccc2ncnc(Nc3ccc(OCc4ccccc4)c(Cl)c3)c2c1	17	ERBB4	ERBB4	=	69
UNC10225279B	Cl.CS(=O)(=O)CCNCc1nc(cs1)-c1ccc2ncnc(Nc3ccc(OCc4ccccc4)c(Cl)c3)c2c1	17	GRK3	ADRBK2	>	10000
UNC10225279B	Cl.CS(=O)(=O)CCNCc1nc(cs1)-c1ccc2ncnc(Nc3ccc(OCc4ccccc4)c(Cl)c3)c2c1	17	MEK5	MAP2K5	=	700
UNC10225279B	Cl.CS(=O)(=O)CCNCc1nc(cs1)-c1ccc2ncnc(Nc3ccc(OCc4ccccc4)c(Cl)c3)c2c1	17	MLCK	MYLK3	>	10000
UNC10225279B	Cl.CS(=O)(=O)CCNCc1nc(cs1)-c1ccc2ncnc(Nc3ccc(OCc4ccccc4)c(Cl)c3)c2c1	17	PRKD3	PRKD3	>	10000
UNC10225285A	Cc1nnc(o1)-c1ccc(C)c(c1)-c1ccc(cc1)C(=O)Nc1ccc(NS(C)(=O)=O)cc1	1	p38-alpha	MAPK14	=	140
UNC10225286A	CSc1ccc(NC2=C(C(=O)NC2=O)c2cccc(Cl)c2)cc1	2	BUB1	BUB1	>	10000
UNC10225286A	CSc1ccc(NC2=C(C(=O)NC2=O)c2cccc(Cl)c2)cc1	2	PIP5K1A	PIP5K1A	>	10000
UNC10225287A	C[C@@H](Oc1cc(sc1C(N)=O)-n1cnc2ccc(NC(=O)NC(C)(C)C)cc12)c1ccccc1Cl	2	PLK1	PLK1	=	7.3
UNC10225287A	C[C@@H](Oc1cc(sc1C(N)=O)-n1cnc2ccc(NC(=O)NC(C)(C)C)cc12)c1ccccc1Cl	2	PLK2	PLK2	=	77
UNC10225287A	C[C@@H](Oc1cc(sc1C(N)=O)-n1cnc2ccc(NC(=O)NC(C)(C)C)cc12)c1ccccc1Cl	2	PLK3	PLK3	=	150
UNC10225289A	c1[nH]nc(c1-c1ccnc2ccccc12)-c1ccccn1	1	TGFBR1	TGFBR1	=	110
UNC10225291A	COc1ccc2cc(ccc2c1)-c1nc([nH]c1-c1ccncc1)-c1ccccc1C(C)C	3	BUB1	BUB1	=	470
UNC10225291A	COc1ccc2cc(ccc2c1)-c1nc([nH]c1-c1ccncc1)-c1ccccc1C(C)C	3	DDR1	DDR1	=	330

UNC10225291A	COc1ccc2cc(ccc2c1)-c1nc([nH]c1-c1ccncc1)-c1ccccc1C(C)C	3	JAK2(JH1do	JAK2	>	10000
UNC10225291A	COc1ccc2cc(ccc2c1)-c1nc([nH]c1-c1ccncc1)-c1ccccc1C(C)C	3	TGFBR1	TGFBR1	=	770
UNC10225291A	COc1ccc2cc(ccc2c1)-c1nc([nH]c1-c1ccncc1)-c1ccccc1C(C)C	3	TIE2	TEK	=	160
UNC10225292A	Fc1ccccc1-c1ccc2c(NC(=O)C3CC3)n[nH]c2c1	2	MYLK2	MYLK2	=	110
UNC10225292A	Fc1ccccc1-c1ccc2c(NC(=O)C3CC3)n[nH]c2c1	2	YSK4	MAP3K19	=	200
UNC10225293A	COc1cccc(c1)C1=C(N(C)c2ccccc2)C(=O)NC1=O	2	NIK	MAP3K14	>	10000
UNC10225294A	COc1ccccc1CNC(=O)c1cc(c[nH]1)C(=O)c1ccccc1C	5	AURKA	AURKA	>	10000
UNC10225294A	COc1ccccc1CNC(=O)c1cc(c[nH]1)C(=O)c1ccccc1C	5	BRSK2	BRSK2	>	10000
UNC10225294A	COc1ccccc1CNC(=O)c1cc(c[nH]1)C(=O)c1ccccc1C	5	CSNK2A2	CSNK2A2	=	5100
UNC10225294A	COc1ccccc1CNC(=O)c1cc(c[nH]1)C(=O)c1ccccc1C	5	MARK1	MARK1	>	10000
UNC10225294A	COc1ccccc1CNC(=O)c1cc(c[nH]1)C(=O)c1ccccc1C	5	p38-alpha	MAPK14	=	270
UNC10225294A	COc1ccccc1CNC(=O)c1cc(c[nH]1)C(=O)c1ccccc1C	5	PLK4	PLK4	>	10000
UNC10225298A	NS(=O)(=O)c1cccc(N\C=C2/C(=O)Nc3ccc(cc23)S(=O)(=O)Cc2c(Cl)cccc2Cl)c1	5	CDKL1	CDKL1	>	10000
UNC10225298A	NS(=O)(=O)c1cccc(N\C=C2/C(=O)Nc3ccc(cc23)S(=O)(=O)Cc2c(Cl)cccc2Cl)c1	5	DAPK1	DAPK1	>	10000
UNC10225298A	NS(=O)(=O)c1cccc(N\C=C2/C(=O)Nc3ccc(cc23)S(=O)(=O)Cc2c(Cl)cccc2Cl)c1	5	MINK	MINK1	=	790
UNC10225298A	NS(=O)(=O)c1cccc(N\C=C2/C(=O)Nc3ccc(cc23)S(=O)(=O)Cc2c(Cl)cccc2Cl)c1	5	PIK4CB	PI4KB	=	1200
UNC10225298A	NS(=O)(=O)c1cccc(N\C=C2/C(=O)Nc3ccc(cc23)S(=O)(=O)Cc2c(Cl)cccc2Cl)c1	5	PRKG2	PRKG2	>	10000
UNC10225298A	NS(=O)(=O)c1cccc(N\C=C2/C(=O)Nc3ccc(cc23)S(=O)(=O)Cc2c(Cl)cccc2Cl)c1	5	RSK1(Kin.Dc	RPS6KA1	>	10000
UNC10225298A	NS(=O)(=O)c1cccc(N\C=C2/C(=O)Nc3ccc(cc23)S(=O)(=O)Cc2c(Cl)cccc2Cl)c1	5	TNIK	TNIK	=	330
UNC10225300A	Fc1cccc(-c2ccc3c(NC(=O)C4CC4)n[nH]c3c2)c1F	6	AAK1	AAK1	=	2100
UNC10225300A	Fc1cccc(-c2ccc3c(NC(=O)C4CC4)n[nH]c3c2)c1F	6	CAMK1G	CAMK1G	>	10000
UNC10225300A	Fc1cccc(-c2ccc3c(NC(=O)C4CC4)n[nH]c3c2)c1F	6	CDK8	CDK8	>	10000
UNC10225300A	Fc1cccc(-c2ccc3c(NC(=O)C4CC4)n[nH]c3c2)c1F	6	CDKL2	CDKL2	=	300
UNC10225300A	Fc1cccc(-c2ccc3c(NC(=O)C4CC4)n[nH]c3c2)c1F	6	LZK	MAP3K13	>	10000
UNC10225300A	Fc1cccc(-c2ccc3c(NC(=O)C4CC4)n[nH]c3c2)c1F	6	MYLK2	MYLK2	=	92
UNC10225300A	Fc1cccc(-c2ccc3c(NC(=O)C4CC4)n[nH]c3c2)c1F	6	PIKFYVE	PIKFYVE	>	10000
UNC10225301A	Nc1nc(c(s1)-c1ccnc2ccccc12)-c1ccccc1F	6	INSR	INSR	>	10000
UNC10225301A	Nc1nc(c(s1)-c1ccnc2ccccc12)-c1ccccc1F	6	JNK2	MAPK9	=	1200
UNC10225301A	Nc1nc(c(s1)-c1ccnc2ccccc12)-c1ccccc1F	6	JNK3	MAPK10	=	610
UNC10225301A	Nc1nc(c(s1)-c1ccnc2ccccc12)-c1ccccc1F	6	MINK	MINK1	=	1700
UNC10225301A	Nc1nc(c(s1)-c1ccnc2ccccc12)-c1ccccc1F	6	p38-alpha	MAPK14	=	240
UNC10225301A	Nc1nc(c(s1)-c1ccnc2ccccc12)-c1ccccc1F	6	RIPK2	RIPK2	>	10000
UNC10225301A	Nc1nc(c(s1)-c1ccnc2ccccc12)-c1ccccc1F	6	RSK2(Kin.Dc	RPS6KA3	=	790
UNC10225301A	Nc1nc(c(s1)-c1ccnc2ccccc12)-c1ccccc1F	6	RSK4(Kin.Dc	RPS6KA6	=	28
UNC10225301A	Nc1nc(c(s1)-c1ccnc2ccccc12)-c1ccccc1F	6	TNNI3K	TNNI3K	>	10000
UNC10225302A	CCC(CC)(CC)n1cnc2c(N)ncnc12	4	EPHA2	EPHA2	>	10000
UNC10225302A	CCC(CC)(CC)n1cnc2c(N)ncnc12	4	FGFR2	FGFR2	>	10000
UNC10225302A	CCC(CC)(CC)n1cnc2c(N)ncnc12	4	TNIK	TNIK	>	10000
UNC10225303A	COc1ccc(cc1)C1=C(Nc2cccc(Cl)c2)C(=O)NC1=O	8	ARK5	NUAK1	>	10000
UNC10225303A	COc1ccc(cc1)C1=C(Nc2cccc(Cl)c2)C(=O)NC1=O	8	CLK1	CLK1	=	390
UNC10225303A	COc1ccc(cc1)C1=C(Nc2cccc(Cl)c2)C(=O)NC1=O	8	CLK2	CLK2	=	1300
UNC10225303A	COc1ccc(cc1)C1=C(Nc2cccc(Cl)c2)C(=O)NC1=O	8	GCN2(Kin.D	EIF2AK4	>	10000
UNC10225303A	COc1ccc(cc1)C1=C(Nc2cccc(Cl)c2)C(=O)NC1=O	8	GSK3A	GSK3A	=	440
UNC10225303A	COc1ccc(cc1)C1=C(Nc2cccc(Cl)c2)C(=O)NC1=O	8	JAK1(JH1do	JAK1	>	10000
UNC10225303A	COc1ccc(cc1)C1=C(Nc2cccc(Cl)c2)C(=O)NC1=O	8	PIK3C2G	PIK3C2G	>	10000

UNC10225303A	COc1ccc(cc1)C1=C(Nc2cccc(Cl)c2)C(=O)NC1=O	8	PIK3CD	PIK3CD	>	10000
UNC10225303A	COc1ccc(cc1)C1=C(Nc2cccc(Cl)c2)C(=O)NC1=O	8	SLK	SLK	=	6300
UNC10225308A	COc1ccc(Nc2nccc(n2)-c2ccc(NC(=O)c3cc4cc(F)ccc4[nH]3)cc2)cc1	2	CHEK1	CHEK1	>	10000
UNC10225308A	COc1ccc(Nc2nccc(n2)-c2ccc(NC(=O)c3cc4cc(F)ccc4[nH]3)cc2)cc1	2	PRKD3	PRKD3	>	10000
UNC10225309A	Nc1nc(c(s1)-c1ccnc2ccccc12)-c1cccnc1	2	CLK3	CLK3	>	10000
UNC10225309A	Nc1nc(c(s1)-c1ccnc2ccccc12)-c1cccnc1	2	HASPIN	GSG2	=	650
UNC10225309A	Nc1nc(c(s1)-c1ccnc2ccccc12)-c1cccnc1	2	MYLK4	MYLK4	=	490
UNC10225309A	Nc1nc(c(s1)-c1ccnc2ccccc12)-c1cccnc1	2	TNIK	TNIK	>	10000
UNC10225311A	COC(=O)c1cccc(NC(=O)NCCNc2ccnc(Nc3ccc(F)cc3)n2)c1	0	DCAMKL1	DCLK1	>	10000
UNC10225311A	COC(=O)c1cccc(NC(=O)NCCNc2ccnc(Nc3ccc(F)cc3)n2)c1	0	HUNK	HUNK	>	10000
UNC10225319A	Fc1cccc(COc2ccc(Nc3ncnc4nn5ccccc5c34)cc2Cl)c1	10	DRAK1	STK17A	>	10000
UNC10225319A	Fc1cccc(COc2ccc(Nc3ncnc4nn5ccccc5c34)cc2Cl)c1	10	EGFR	EGFR	=	1.8
UNC10225319A	Fc1cccc(COc2ccc(Nc3ncnc4nn5ccccc5c34)cc2Cl)c1	10	ERBB2	ERBB2	=	24
UNC10225319A	Fc1cccc(COc2ccc(Nc3ncnc4nn5ccccc5c34)cc2Cl)c1	10	ERBB4	ERBB4	=	160
UNC10225322A	CC(C)c1ccc(O)cc1Nc1ccnc2ccc(cc12)C(F)(F)F	0	CDK4-cyclin	CDK4	>	10000
UNC10225322A	CC(C)c1ccc(O)cc1Nc1ccnc2ccc(cc12)C(F)(F)F	0	RIPK2	RIPK2	>	10000
UNC10225322A	CC(C)c1ccc(O)cc1Nc1ccnc2ccc(cc12)C(F)(F)F	0	ROCK1	ROCK1	>	10000
UNC10225326A	O=C(Nc1n[nH]c2cc(ccc12)-c1ccsc1)C1CC1	4	CDK4	CDK4	>	10000
UNC10225326A	O=C(Nc1n[nH]c2cc(ccc12)-c1ccsc1)C1CC1	4	CDKL2	CDKL2	=	100
UNC10225326A	O=C(Nc1n[nH]c2cc(ccc12)-c1ccsc1)C1CC1	4	ERK8	MAPK15	=	48
UNC10225326A	O=C(Nc1n[nH]c2cc(ccc12)-c1ccsc1)C1CC1	4	MYLK2	MYLK2	=	41
UNC10225326A	O=C(Nc1n[nH]c2cc(ccc12)-c1ccsc1)C1CC1	4	PDGFRB	PDGFRB	=	700
UNC10225326A	O=C(Nc1n[nH]c2cc(ccc12)-c1ccsc1)C1CC1	4	PRKD1	PRKD1	>	10000
UNC10225326A	O=C(Nc1n[nH]c2cc(ccc12)-c1ccsc1)C1CC1	4	RIOK1	RIOK1	=	180
UNC10225326A	O=C(Nc1n[nH]c2cc(ccc12)-c1ccsc1)C1CC1	4	TRKA	NTRK1	=	3100
UNC10225326A	O=C(Nc1n[nH]c2cc(ccc12)-c1ccsc1)C1CC1	4	YSK4	MAP3K19	=	120
UNC10225327A	Cc1cccc(n1)-c1nc(Nc2ccncc2)c2ccccc2n1	5	ACVR1B	ACVR1B	=	3.3
UNC10225327A	Cc1cccc(n1)-c1nc(Nc2ccncc2)c2ccccc2n1	5	CIT	CIT	=	440
UNC10225327A	Cc1cccc(n1)-c1nc(Nc2ccncc2)c2ccccc2n1	5	PRKD1	PRKD1	=	9.7
UNC10225327A	Cc1cccc(n1)-c1nc(Nc2ccncc2)c2ccccc2n1	5	PRKD2	PRKD2	=	17
UNC10225327A	Cc1cccc(n1)-c1nc(Nc2ccncc2)c2ccccc2n1	5	TGFBR1	TGFBR1	=	3.5
UNC10225327A	Cc1cccc(n1)-c1nc(Nc2ccncc2)c2ccccc2n1	5	TGFBR2	TGFBR2	=	290
UNC10225329A	Cn1cncc1C(=O)NC1CCN(C1)c1ccnc(Nc2ccc(F)cc2)n1	10	CDKL1	CDKL1	>	10000
UNC10225329A	Cn1cncc1C(=O)NC1CCN(C1)c1ccnc(Nc2ccc(F)cc2)n1	10	CDKL3	CDKL3	>	10000
UNC10225329A	Cn1cncc1C(=O)NC1CCN(C1)c1ccnc(Nc2ccc(F)cc2)n1	10	FLT4	FLT4	>	10000
UNC10225329A	Cn1cncc1C(=O)NC1CCN(C1)c1ccnc(Nc2ccc(F)cc2)n1	10	JAK2(JH1do	JAK2	=	1500
UNC10225329A	Cn1cncc1C(=O)NC1CCN(C1)c1ccnc(Nc2ccc(F)cc2)n1	10	PRKG2	PRKG2	>	10000
UNC10225329A	Cn1cncc1C(=O)NC1CCN(C1)c1ccnc(Nc2ccc(F)cc2)n1	10	SRPK1	SRPK1	>	10000
UNC10225329A	Cn1cncc1C(=O)NC1CCN(C1)c1ccnc(Nc2ccc(F)cc2)n1	10	TESK1	TESK1	>	10000
UNC10225329A	Cn1cncc1C(=O)NC1CCN(C1)c1ccnc(Nc2ccc(F)cc2)n1	10	TXK	TXK	>	10000
UNC10225329A	Cn1cncc1C(=O)NC1CCN(C1)c1ccnc(Nc2ccc(F)cc2)n1	10	ZAK	ZAK	>	10000
UNC10225330A	COC(=O)c1cccc(NC(=O)NC2CCN(C2)c2ccnc(Nc3ccc(OC)cc3)n2)c1	3	CAMK2A	CAMK2A	>	10000
UNC10225330A	COC(=O)c1cccc(NC(=O)NC2CCN(C2)c2ccnc(Nc3ccc(OC)cc3)n2)c1	3	CAMK2B	CAMK2B	>	10000
UNC10225330A	COC(=O)c1cccc(NC(=O)NC2CCN(C2)c2ccnc(Nc3ccc(OC)cc3)n2)c1	3	CLK2	CLK2	>	10000
UNC10225330A	COC(=O)c1cccc(NC(=O)NC2CCN(C2)c2ccnc(Nc3ccc(OC)cc3)n2)c1	3	TRKC	NTRK3	=	1000
UNC10225331A	CC[C@@H]1CO[C@@H](CN1c1cc(nc(NC)n1)-c1ccc2c(N)n[nH]c2c1)C(=O)Nc1ccccc1	6	CIT	CIT	=	200
UNC10225331A	CC[C@@H]1CO[C@@H](CN1c1cc(nc(NC)n1)-c1ccc2c(N)n[nH]c2c1)C(=O)Nc1ccccc1	6	DCAMKL3	DCLK3	=	570

UNC10225331A	CC[C@@H]1CO[C@@H](CN1c1cc(nc(NC)n1)-c1ccc2c(N)n[nH]c2c1)C(=O)Nc1ccccc1	6	DRAK1	STK17A	=	220
UNC10225331A	CC[C@@H]1CO[C@@H](CN1c1cc(nc(NC)n1)-c1ccc2c(N)n[nH]c2c1)C(=O)Nc1ccccc1	6	PDPK1	PDPK1	=	1.7
UNC10225331A	CC[C@@H]1CO[C@@H](CN1c1cc(nc(NC)n1)-c1ccc2c(N)n[nH]c2c1)C(=O)Nc1ccccc1	6	PKN1	PKN1	>	10000
UNC10225331A	CC[C@@H]1CO[C@@H](CN1c1cc(nc(NC)n1)-c1ccc2c(N)n[nH]c2c1)C(=O)Nc1ccccc1	6	PRKG2	PRKG2	=	400
UNC10225331A	CC[C@@H]1CO[C@@H](CN1c1cc(nc(NC)n1)-c1ccc2c(N)n[nH]c2c1)C(=O)Nc1ccccc1	6	PRKX	PRKX	=	130
UNC10225332A	Fc1cccc(Cn2ccn3c2nc(cc3=O)N2CCOCC2)c1	2	PIK3CB	PIK3CB	=	55
UNC10225332A	Fc1cccc(Cn2ccn3c2nc(cc3=O)N2CCOCC2)c1	2	VPS34	PIK3C3	=	33
UNC10225334A	COc1ccc(cc1)-c1nc2CCCn2c1-c1ccncc1	1	DAPK1	DAPK1	>	10000
UNC10225334A	COc1ccc(cc1)-c1nc2CCCn2c1-c1ccncc1	1	p38-alpha	MAPK14	=	220
UNC10225334A	COc1ccc(cc1)-c1nc2CCCn2c1-c1ccncc1	1	RSK1(Kin.Dc	RPS6KA1	=	440
UNC10225334B	COc1ccc(cc1)-c1nc2CCCn2c1-c1ccncc1	2	p38-alpha	MAPK14	=	130
UNC10225334B	COc1ccc(cc1)-c1nc2CCCn2c1-c1ccncc1	2	YSK4	MAP3K19	>	10000
UNC10225335A	COc1ccc(Nc2nccc(NCCNC(=O)Nc3ccccc3F)n2)cc1	2	AAK1	AAK1	>	10000
UNC10225335A	COc1ccc(Nc2nccc(NCCNC(=O)Nc3ccccc3F)n2)cc1	2	CLK3	CLK3	>	10000
UNC10225335A	COc1ccc(Nc2nccc(NCCNC(=O)Nc3ccccc3F)n2)cc1	2	CSNK1E	CSNK1E	>	10000
UNC10225335A	COc1ccc(Nc2nccc(NCCNC(=O)Nc3ccccc3F)n2)cc1	2	PRKD2	PRKD2	>	10000
UNC10225336A	Clc1cc(Cl)cc(Nc2ccnc(Nc3cc(Cl)cc(Cl)c3)n2)c1	4	AURKA	AURKA	>	10000
UNC10225336A	Clc1cc(Cl)cc(Nc2ccnc(Nc3cc(Cl)cc(Cl)c3)n2)c1	4	CSNK2A2	CSNK2A2	>	10000
UNC10225336A	Clc1cc(Cl)cc(Nc2ccnc(Nc3cc(Cl)cc(Cl)c3)n2)c1	4	GRK1	GRK1	>	10000
UNC10225336A	Clc1cc(Cl)cc(Nc2ccnc(Nc3cc(Cl)cc(Cl)c3)n2)c1	4	RIOK2	RIOK2	=	150
UNC10225337A	c1cc2c(ccnc2[nH]1)-c1ccccc1	4	BRSK2	BRSK2	>	10000
UNC10225337A	c1cc2c(ccnc2[nH]1)-c1ccccc1	4	CSNK1G1	CSNK1G1	>	10000
UNC10225337A	c1cc2c(ccnc2[nH]1)-c1ccccc1	4	GAK	GAK	=	3700
UNC10225337A	c1cc2c(ccnc2[nH]1)-c1ccccc1	4	MARK1	MARK1	>	10000
UNC10225337A	c1cc2c(ccnc2[nH]1)-c1ccccc1	4	ROCK1	ROCK1	=	150
UNC10225337A	c1cc2c(ccnc2[nH]1)-c1ccccc1	4	ROCK2	ROCK2	=	530
UNC10225337A	c1cc2c(ccnc2[nH]1)-c1ccccc1	4	YSK4	MAP3K19	=	470
UNC10225339A	CC1=C(C(CC(=O)N1)c1ccc(cc1)C(F)(F)F)C(=O)Nc1cc2cn[nH]c2cc1F	2	ACVR2A	ACVR2A	>	10000
UNC10225339A	CC1=C(C(CC(=O)N1)c1ccc(cc1)C(F)(F)F)C(=O)Nc1cc2cn[nH]c2cc1F	2	PRKX	PRKX	=	350
UNC10225339A	CC1=C(C(CC(=O)N1)c1ccc(cc1)C(F)(F)F)C(=O)Nc1cc2cn[nH]c2cc1F	2	ROCK1	ROCK1	=	2.7
UNC10225339A	CC1=C(C(CC(=O)N1)c1ccc(cc1)C(F)(F)F)C(=O)Nc1cc2cn[nH]c2cc1F	2	ROCK2	ROCK2	=	2.7
UNC10225339A	CC1=C(C(CC(=O)N1)c1ccc(cc1)C(F)(F)F)C(=O)Nc1cc2cn[nH]c2cc1F	2	SNARK	NUAK2	=	470
UNC10225343A	Fc1ccc(Cn2ccn3c2nc(cc3=O)N2CCOCC2)cc1Cl	3	JAK2(JH1do	JAK2	>	10000
UNC10225343A	Fc1ccc(Cn2ccn3c2nc(cc3=O)N2CCOCC2)cc1Cl	3	PIK3CB	PIK3CB	=	11
UNC10225343A	Fc1ccc(Cn2ccn3c2nc(cc3=O)N2CCOCC2)cc1Cl	3	VPS34	PIK3C3	=	15
UNC10225344A	Nc1n[nH]c2cc(ccc12)-c1cc(nc(N)n1)N1CCCCC1	2	DRAK1	STK17A	=	110
UNC10225344A	Nc1n[nH]c2cc(ccc12)-c1cc(nc(N)n1)N1CCCCC1	2	PDPK1	PDPK1	=	540
UNC10225346A	CSc1ccc(NC2=C(C(=O)NC2=O)c2ccccc2Cl)cc1	2	CLK2	CLK2	=	460
UNC10225346A	CSc1ccc(NC2=C(C(=O)NC2=O)c2ccccc2Cl)cc1	2	GSK3A	GSK3A	=	580
UNC10225346A	CSc1ccc(NC2=C(C(=O)NC2=O)c2ccccc2Cl)cc1	2	MKNK1	MKNK1	>	10000
UNC10225346A	CSc1ccc(NC2=C(C(=O)NC2=O)c2ccccc2Cl)cc1	2	PDPK1	PDPK1	>	10000
UNC10225346A	CSc1ccc(NC2=C(C(=O)NC2=O)c2ccccc2Cl)cc1	2	SLK	SLK	=	770
UNC10225352A	OCCCNc1nccc(n1)-c1c(nn2cc(ccc12)C(F)(F)F)-c1cccc(c1)C(F)(F)F	3	GRK1	GRK1	>	10000

UNC10225352A	OCCCNc1nccc(n1)-c1c(nn2cc(ccc12)C(F)(F)F)-c1cccc(c1)C(F)(F)F	3	NLK	NLK	=	230
UNC10225352A	OCCCNc1nccc(n1)-c1c(nn2cc(ccc12)C(F)(F)F)-c1cccc(c1)C(F)(F)F	3	p38-alpha	MAPK14	=	1300
UNC10225352A	OCCCNc1nccc(n1)-c1c(nn2cc(ccc12)C(F)(F)F)-c1cccc(c1)C(F)(F)F	3	RSK4(Kin.Dc	RPS6KA6	=	2700
UNC10225352A	OCCCNc1nccc(n1)-c1c(nn2cc(ccc12)C(F)(F)F)-c1cccc(c1)C(F)(F)F	3	WNK4	WNK4	>	10000
UNC10225353A	Cc1ccc(NC(=O)Nc2cc(nn2-c2ccccc2)C(C)(C)C)cc1	13	BRSK1	BRSK1	>	10000
UNC10225353A	Cc1ccc(NC(=O)Nc2cc(nn2-c2ccccc2)C(C)(C)C)cc1	13	DDR1	DDR1	=	1300
UNC10225353A	Cc1ccc(NC(=O)Nc2cc(nn2-c2ccccc2)C(C)(C)C)cc1	13	DDR2	DDR2	=	410
UNC10225353A	Cc1ccc(NC(=O)Nc2cc(nn2-c2ccccc2)C(C)(C)C)cc1	13	FLT3	FLT3	=	26
UNC10225353A	Cc1ccc(NC(=O)Nc2cc(nn2-c2ccccc2)C(C)(C)C)cc1	13	GRK3	ADRBK2	>	10000
UNC10225353A	Cc1ccc(NC(=O)Nc2cc(nn2-c2ccccc2)C(C)(C)C)cc1	13	IRAK3	IRAK3	>	10000
UNC10225353A	Cc1ccc(NC(=O)Nc2cc(nn2-c2ccccc2)C(C)(C)C)cc1	13	KIT	KIT	=	260
UNC10225353A	Cc1ccc(NC(=O)Nc2cc(nn2-c2ccccc2)C(C)(C)C)cc1	13	MKNK2	MKNK2	=	370
UNC10225353A	Cc1ccc(NC(=O)Nc2cc(nn2-c2ccccc2)C(C)(C)C)cc1	13	p38-alpha	MAPK14	=	58
UNC10225353A	Cc1ccc(NC(=O)Nc2cc(nn2-c2ccccc2)C(C)(C)C)cc1	13	p38-beta	MAPK11	=	540
UNC10225353A	Cc1ccc(NC(=O)Nc2cc(nn2-c2ccccc2)C(C)(C)C)cc1	13	PDGFRB	PDGFRB	=	340
UNC10225353A	Cc1ccc(NC(=O)Nc2cc(nn2-c2ccccc2)C(C)(C)C)cc1	13	RAF1	RAF1	=	270
UNC10225355A	Cc1ccccc1Cn1ccn2c1nc(cc2=O)N1CCOCC1	4	BRSK2	BRSK2	>	10000
UNC10225355A	Cc1ccccc1Cn1ccn2c1nc(cc2=O)N1CCOCC1	4	MARK1	MARK1	>	10000
UNC10225355A	Cc1ccccc1Cn1ccn2c1nc(cc2=O)N1CCOCC1	4	MARK4	MARK4	>	10000
UNC10225355A	Cc1ccccc1Cn1ccn2c1nc(cc2=O)N1CCOCC1	4	PIK3CB	PIK3CB	=	27
UNC10225355A	Cc1ccccc1Cn1ccn2c1nc(cc2=O)N1CCOCC1	4	VPS34	PIK3C3	=	180
UNC10225359A	Cc1ccc(NC(=O)Nc2ccc(Cl)c(c2)C(F)(F)F)cc1-c1ccc(cc1)C(=O)Nc1ccncc1	8	CDKL1	CDKL1	>	10000
UNC10225359A	Cc1ccc(NC(=O)Nc2ccc(Cl)c(c2)C(F)(F)F)cc1-c1ccc(cc1)C(=O)Nc1ccncc1	8	CIT	CIT	=	380
UNC10225359A	Cc1ccc(NC(=O)Nc2ccc(Cl)c(c2)C(F)(F)F)cc1-c1ccc(cc1)C(=O)Nc1ccncc1	8	DDR1	DDR1	=	120
UNC10225359A	Cc1ccc(NC(=O)Nc2ccc(Cl)c(c2)C(F)(F)F)cc1-c1ccc(cc1)C(=O)Nc1ccncc1	8	KIT	KIT	=	560
UNC10225359A	Cc1ccc(NC(=O)Nc2ccc(Cl)c(c2)C(F)(F)F)cc1-c1ccc(cc1)C(=O)Nc1ccncc1	8	p38-alpha	MAPK14	=	310
UNC10225359A	Cc1ccc(NC(=O)Nc2ccc(Cl)c(c2)C(F)(F)F)cc1-c1ccc(cc1)C(=O)Nc1ccncc1	8	p38-beta	MAPK11	=	90
UNC10225359A	Cc1ccc(NC(=O)Nc2ccc(Cl)c(c2)C(F)(F)F)cc1-c1ccc(cc1)C(=O)Nc1ccncc1	8	PDGFRB	PDGFRB	=	270
UNC10225359A	Cc1ccc(NC(=O)Nc2ccc(Cl)c(c2)C(F)(F)F)cc1-c1ccc(cc1)C(=O)Nc1ccncc1	8	RAF1	RAF1	=	340
UNC10225360A	Oc1ccc2ncnc(Nc3ccc(OCc4ccccc4)cc3)c2c1	8	EGFR	EGFR	=	9.3
UNC10225360A	Oc1ccc2ncnc(Nc3ccc(OCc4ccccc4)cc3)c2c1	8	ERBB2	ERBB2	=	27
UNC10225360A	Oc1ccc2ncnc(Nc3ccc(OCc4ccccc4)cc3)c2c1	8	LTK	LTK	>	10000
UNC10225360A	Oc1ccc2ncnc(Nc3ccc(OCc4ccccc4)cc3)c2c1	8	MEK5	MAP2K5	=	82
UNC10225360A	Oc1ccc2ncnc(Nc3ccc(OCc4ccccc4)cc3)c2c1	8	PAK3	PAK3	>	10000
UNC10225360A	Oc1ccc2ncnc(Nc3ccc(OCc4ccccc4)cc3)c2c1	8	RIPK2	RIPK2	=	8.1
UNC10225362A	CC(C)(C)n1cnc2c(N)ncnc12	1	RSK1(Kin.Dc	RPS6KA1	>	10000
UNC10225362A	CC(C)(C)n1cnc2c(N)ncnc12	1	SgK110	SgK110	>	10000
UNC10225362A	CC(C)(C)n1cnc2c(N)ncnc12	1	VRK2	VRK2	>	10000
UNC10225364A	Cc1cc(NCC(c2ccccc2)c2ccccc2)nc(NCc2ccc(cc2)C(F)(F)F)n1	0	ADCK4	ADCK4	=	2300
UNC10225365A	O[C@H]([C@@H](O)C(O)=O)C(O)=O.Brc1cc2c(NC(=O)C3CCN(Cc4ccccc4)C3)n[nH]c2nc1-c1ccco1	4	CDK7	CDK7	=	1100

UNC10225365A	O[C@H]([C@@H](O)C(O)=O)C(O)=O.Brc1cc2c(NC(=O)C3CCN(Cc4ccccc4)C3)n[nH]c2nc1-c1ccco1	4	CDKL5	CDKL5	=	1100
UNC10225365A	O[C@H]([C@@H](O)C(O)=O)C(O)=O.Brc1cc2c(NC(=O)C3CCN(Cc4ccccc4)C3)n[nH]c2nc1-c1ccco1	4	GSK3A	GSK3A	=	130
UNC10225365A	O[C@H]([C@@H](O)C(O)=O)C(O)=O.Brc1cc2c(NC(=O)C3CCN(Cc4ccccc4)C3)n[nH]c2nc1-c1ccco1	4	MYLK2	MYLK2	=	460
UNC10225365A	O[C@H]([C@@H](O)C(O)=O)C(O)=O.Brc1cc2c(NC(=O)C3CCN(Cc4ccccc4)C3)n[nH]c2nc1-c1ccco1	4	PCTK1	CDK16	=	150
UNC10225365A	O[C@H]([C@@H](O)C(O)=O)C(O)=O.Brc1cc2c(NC(=O)C3CCN(Cc4ccccc4)C3)n[nH]c2nc1-c1ccco1	4	PCTK2	CDK17	=	240
UNC10225367A	CC(C)(C)c1ccc(cc1)-n1cnc2c(N)ncnc12	3	DRAK1	STK17A	=	450
UNC10225367A	CC(C)(C)c1ccc(cc1)-n1cnc2c(N)ncnc12	3	JAK1(JH2do	JAK1	=	250
UNC10225367A	CC(C)(C)c1ccc(cc1)-n1cnc2c(N)ncnc12	3	PIK3C2B	PIK3C2B	=	8300
UNC10225367A	CC(C)(C)c1ccc(cc1)-n1cnc2c(N)ncnc12	3	PIK3CA	PIK3CA	>	10000
UNC10225367A	CC(C)(C)c1ccc(cc1)-n1cnc2c(N)ncnc12	3	PIK3CG	PIK3CG	=	2400
UNC10225372A	COc1cc2ncn(-c3cc(OCC4CCCO4)c(s3)C(N)=O)c2cc1OC	9	AURKC	AURKC	>	10000
UNC10225372A	COc1cc2ncn(-c3cc(OCC4CCCO4)c(s3)C(N)=O)c2cc1OC	9	LOK	STK10	=	100
UNC10225372A	COc1cc2ncn(-c3cc(OCC4CCCO4)c(s3)C(N)=O)c2cc1OC	9	MEK5	MAP2K5	=	81
UNC10225372A	COc1cc2ncn(-c3cc(OCC4CCCO4)c(s3)C(N)=O)c2cc1OC	9	MYLK4	MYLK4	=	500
UNC10225372A	COc1cc2ncn(-c3cc(OCC4CCCO4)c(s3)C(N)=O)c2cc1OC	9	PIP5K1C	PIP5K1C	=	7700
UNC10225372A	COc1cc2ncn(-c3cc(OCC4CCCO4)c(s3)C(N)=O)c2cc1OC	9	PLK1	PLK1	=	13
UNC10225372A	COc1cc2ncn(-c3cc(OCC4CCCO4)c(s3)C(N)=O)c2cc1OC	9	PLK2	PLK2	=	4.4
UNC10225372A	COc1cc2ncn(-c3cc(OCC4CCCO4)c(s3)C(N)=O)c2cc1OC	9	PLK3	PLK3	=	40
UNC10225372A	COc1cc2ncn(-c3cc(OCC4CCCO4)c(s3)C(N)=O)c2cc1OC	9	PRKCI	PRKCI	>	10000
UNC10225372A	COc1cc2ncn(-c3cc(OCC4CCCO4)c(s3)C(N)=O)c2cc1OC	9	RIOK3	RIOK3	=	670
UNC10225372A	COc1cc2ncn(-c3cc(OCC4CCCO4)c(s3)C(N)=O)c2cc1OC	9	SIK2	SIK2	>	10000
UNC10225372A	COc1cc2ncn(-c3cc(OCC4CCCO4)c(s3)C(N)=O)c2cc1OC	9	SLK	SLK	>	10000
UNC10225372A	COc1cc2ncn(-c3cc(OCC4CCCO4)c(s3)C(N)=O)c2cc1OC	9	STK35	STK35	=	3600
UNC10225373A	Oc1c(Cl)cc(NC2=C(C(=O)NC2=O)c2ccccc2)cc1Cl	1	JAK3(JH1do	JAK3	=	380
UNC10225373A	Oc1c(Cl)cc(NC2=C(C(=O)NC2=O)c2ccccc2)cc1Cl	1	PIP5K1C	PIP5K1C	=	1500
UNC10225374A	CNc1cc(nc(N)n1)-c1ccc2c(N)n[nH]c2c1	2	CHEK2	CHEK2	=	2000
UNC10225374A	CNc1cc(nc(N)n1)-c1ccc2c(N)n[nH]c2c1	2	CLK1	CLK1	=	330
UNC10225374A	CNc1cc(nc(N)n1)-c1ccc2c(N)n[nH]c2c1	2	CLK2	CLK2	=	410
UNC10225374A	CNc1cc(nc(N)n1)-c1ccc2c(N)n[nH]c2c1	2	CLK4	CLK4	=	140
UNC10225374A	CNc1cc(nc(N)n1)-c1ccc2c(N)n[nH]c2c1	2	DRAK1	STK17A	=	89
UNC10225374A	CNc1cc(nc(N)n1)-c1ccc2c(N)n[nH]c2c1	2	DYRK2	DYRK2	=	33
UNC10225374A	CNc1cc(nc(N)n1)-c1ccc2c(N)n[nH]c2c1	2	PRKG2	PRKG2	=	39
UNC10225374A	CNc1cc(nc(N)n1)-c1ccc2c(N)n[nH]c2c1	2	RIOK1	RIOK1	=	1100
UNC10225374A	CNc1cc(nc(N)n1)-c1ccc2c(N)n[nH]c2c1	2	WNK4	WNK4	>	10000
UNC10225375A	COc1ccc(Nc2nccc(n2)N2CCC(C2)NC(=O)Nc2cccc(c2)C(C)=O)cc1	1	VRK2	VRK2	>	10000
UNC10225378A	Cl.C(c1ccccc1)n1cnc2cc(Nc3ncnc4ccccc34)ccc12	0	EPHB6	EPHB6	=	510
UNC10225379A	NS(=O)(=O)c1cccc(c1)-c1ccc2nccc(-c3cccc(c3)S(N)(=O)=O)c2c1	1	PIK3CG	PIK3CG	=	81
UNC10225379A	NS(=O)(=O)c1cccc(c1)-c1ccc2nccc(-c3cccc(c3)S(N)(=O)=O)c2c1	1	PIK4CB	PI4KB	=	32
UNC10225379A	NS(=O)(=O)c1cccc(c1)-c1ccc2nccc(-c3cccc(c3)S(N)(=O)=O)c2c1	1	ULK2	ULK2	>	10000
UNC10225382A	Fc1ccc(cc1)-c1ncn(CCCN2CCOCC2)c1-c1ccccn1	1	PRKCI	PRKCI	>	10000
UNC10225384A	Clc1cc2c(NC(=O)C3CC3)n[nH]c2nc1-c1ccccc1	3	GRK3	ADRBK2	>	10000
UNC10225384A	Clc1cc2c(NC(=O)C3CC3)n[nH]c2nc1-c1ccccc1	3	GSK3A	GSK3A	=	390
UNC10225384A	Clc1cc2c(NC(=O)C3CC3)n[nH]c2nc1-c1ccccc1	3	MYLK2	MYLK2	=	150
UNC10225384A	Clc1cc2c(NC(=O)C3CC3)n[nH]c2nc1-c1ccccc1	3	PDGFRB	PDGFRB	=	170
UNC10225384A	Clc1cc2c(NC(=O)C3CC3)n[nH]c2nc1-c1ccccc1	3	YSK4	MAP3K19	=	7700
UNC10225386A	COc1cc2ncn(-c3cc(OCc4ccncc4Br)c(s3)C(N)=O)c2cc1OC	5	LOK	STK10	=	500

UNC10225386A	COc1cc2ncn(-c3cc(OCc4ccncc4Br)c(s3)C(N)=O)c2cc1OC	5	MEK5	MAP2K5	=	78
UNC10225386A	COc1cc2ncn(-c3cc(OCc4ccncc4Br)c(s3)C(N)=O)c2cc1OC	5	PIP5K1C	PIP5K1C	=	610
UNC10225386A	COc1cc2ncn(-c3cc(OCc4ccncc4Br)c(s3)C(N)=O)c2cc1OC	5	PIP5K2C	PIP4K2C	=	330
UNC10225386A	COc1cc2ncn(-c3cc(OCc4ccncc4Br)c(s3)C(N)=O)c2cc1OC	5	PLK1	PLK1	=	1.1
UNC10225386A	COc1cc2ncn(-c3cc(OCc4ccncc4Br)c(s3)C(N)=O)c2cc1OC	5	PLK2	PLK2	=	1.4
UNC10225386A	COc1cc2ncn(-c3cc(OCc4ccncc4Br)c(s3)C(N)=O)c2cc1OC	5	PLK3	PLK3	=	9.4
UNC10225386A	COc1cc2ncn(-c3cc(OCc4ccncc4Br)c(s3)C(N)=O)c2cc1OC	5	SLK	SLK	=	680
UNC10225387B	Cl.C[C@H](N[C@@H]1CCc2cc(ccc2C1)-c1cnc(N)c(C)c1)c1ccccc1	5	AAK1	AAK1	=	3600
UNC10225387B	Cl.C[C@H](N[C@@H]1CCc2cc(ccc2C1)-c1cnc(N)c(C)c1)c1ccccc1	5	CAMK1G	CAMK1G	>	10000
UNC10225387B	Cl.C[C@H](N[C@@H]1CCc2cc(ccc2C1)-c1cnc(N)c(C)c1)c1ccccc1	5	CAMK2D	CAMK2D	>	10000
UNC10225387B	Cl.C[C@H](N[C@@H]1CCc2cc(ccc2C1)-c1cnc(N)c(C)c1)c1ccccc1	5	DLK	MAP3K12	=	280
UNC10225387B	Cl.C[C@H](N[C@@H]1CCc2cc(ccc2C1)-c1cnc(N)c(C)c1)c1ccccc1	5	MAP4K2	MAP4K2	=	43
UNC10225387B	Cl.C[C@H](N[C@@H]1CCc2cc(ccc2C1)-c1cnc(N)c(C)c1)c1ccccc1	5	RIOK2	RIOK2	=	650
UNC10225387B	Cl.C[C@H](N[C@@H]1CCc2cc(ccc2C1)-c1cnc(N)c(C)c1)c1ccccc1	5	TRKA	NTRK1	=	2500
UNC10225387B	Cl.C[C@H](N[C@@H]1CCc2cc(ccc2C1)-c1cnc(N)c(C)c1)c1ccccc1	5	YSK4	MAP3K19	=	2400
UNC10225388A	Cc1ccc(NC(=O)Nc2ccc(Cl)cc2)cc1-c1ccc(cc1)C(=O)Nc1ccncc1	6	CIT	CIT	=	430
UNC10225388A	Cc1ccc(NC(=O)Nc2ccc(Cl)cc2)cc1-c1ccc(cc1)C(=O)Nc1ccncc1	6	CSF1R	CSF1R	=	6700
UNC10225388A	Cc1ccc(NC(=O)Nc2ccc(Cl)cc2)cc1-c1ccc(cc1)C(=O)Nc1ccncc1	6	DDR1	DDR1	=	73
UNC10225388A	Cc1ccc(NC(=O)Nc2ccc(Cl)cc2)cc1-c1ccc(cc1)C(=O)Nc1ccncc1	6	KIT	KIT	=	140
UNC10225388A	Cc1ccc(NC(=O)Nc2ccc(Cl)cc2)cc1-c1ccc(cc1)C(=O)Nc1ccncc1	6	PDGFRB	PDGFRB	=	86
UNC10225388A	Cc1ccc(NC(=O)Nc2ccc(Cl)cc2)cc1-c1ccc(cc1)C(=O)Nc1ccncc1	6	RAF1	RAF1	=	350
UNC10225389A	CCCCCc1nc2c(N)ncnc2n1CCCCC	1	ROCK1	ROCK1	=	280
UNC10225391A	COc1ccc(CNC(=O)NC2CCN(C2)c2ccnc(Nc3cc(OC)c(OC)c(OC)c3)n2)cc1	5	AURKB	AURKB	=	330
UNC10225391A	COc1ccc(CNC(=O)NC2CCN(C2)c2ccnc(Nc3cc(OC)c(OC)c(OC)c3)n2)cc1	5	DLK	MAP3K12	=	5100
UNC10225391A	COc1ccc(CNC(=O)NC2CCN(C2)c2ccnc(Nc3cc(OC)c(OC)c(OC)c3)n2)cc1	5	EPHB6	EPHB6	=	180
UNC10225391A	COc1ccc(CNC(=O)NC2CCN(C2)c2ccnc(Nc3cc(OC)c(OC)c(OC)c3)n2)cc1	5	JAK2(JH1do	JAK2	=	350
UNC10225391A	COc1ccc(CNC(=O)NC2CCN(C2)c2ccnc(Nc3cc(OC)c(OC)c(OC)c3)n2)cc1	5	MEK5	MAP2K5	=	95
UNC10225391A	COc1ccc(CNC(=O)NC2CCN(C2)c2ccnc(Nc3cc(OC)c(OC)c(OC)c3)n2)cc1	5	NEK5	NEK5	=	730
UNC10225391A	COc1ccc(CNC(=O)NC2CCN(C2)c2ccnc(Nc3cc(OC)c(OC)c(OC)c3)n2)cc1	5	PIP5K2C	PIP4K2C	=	600
UNC10225391A	COc1ccc(CNC(=O)NC2CCN(C2)c2ccnc(Nc3cc(OC)c(OC)c(OC)c3)n2)cc1	5	ULK3	ULK3	=	550
UNC10225398A	Cc1ccc(NC(=O)Nc2ccccc2F)cc1-c1ccc(cc1)C(=O)Nc1ccncc1	5	CIT	CIT	=	570

UNC10225398A	Cc1ccc(NC(=O)Nc2ccccc2F)cc1-c1ccc(cc1)C(=O)Nc1ccncc1	5	CSF1R	CSF1R	=	2600
UNC10225398A	Cc1ccc(NC(=O)Nc2ccccc2F)cc1-c1ccc(cc1)C(=O)Nc1ccncc1	5	DDR1	DDR1	=	180
UNC10225398A	Cc1ccc(NC(=O)Nc2ccccc2F)cc1-c1ccc(cc1)C(=O)Nc1ccncc1	5	KIT	KIT	=	450
UNC10225398A	Cc1ccc(NC(=O)Nc2ccccc2F)cc1-c1ccc(cc1)C(=O)Nc1ccncc1	5	PDGFRB	PDGFRB	=	47
UNC10225398A	Cc1ccc(NC(=O)Nc2ccccc2F)cc1-c1ccc(cc1)C(=O)Nc1ccncc1	5	YSK4	MAP3K19	=	1500
UNC10225400A	NS(=O)(=O)c1cccc(c1)-c1ccc2ncc(nc2c1)N1CCOCC1	1	PIK4CB	PI4KB	=	860
UNC10225400A	NS(=O)(=O)c1cccc(c1)-c1ccc2ncc(nc2c1)N1CCOCC1	1	VPS34	PIK3C3	>	10000
UNC10225400A	NS(=O)(=O)c1cccc(c1)-c1ccc2ncc(nc2c1)N1CCOCC1	1	YSK4	MAP3K19	=	3400
UNC10225401A	OC(=O)c1cccc(NC2=C(C(=O)NC2=O)c2ccccc2Cl)c1	11	ABL1-phosp	ABL1	>	10000
UNC10225401A	OC(=O)c1cccc(NC2=C(C(=O)NC2=O)c2ccccc2Cl)c1	11	CLK2	CLK2	=	400
UNC10225401A	OC(=O)c1cccc(NC2=C(C(=O)NC2=O)c2ccccc2Cl)c1	11	HIPK1	HIPK1	=	210
UNC10225401A	OC(=O)c1cccc(NC2=C(C(=O)NC2=O)c2ccccc2Cl)c1	11	HIPK2	HIPK2	=	140
UNC10225401A	OC(=O)c1cccc(NC2=C(C(=O)NC2=O)c2ccccc2Cl)c1	11	HIPK3	HIPK3	=	340
UNC10225401A	OC(=O)c1cccc(NC2=C(C(=O)NC2=O)c2ccccc2Cl)c1	11	IKK-alpha	CHUK	>	10000
UNC10225401A	OC(=O)c1cccc(NC2=C(C(=O)NC2=O)c2ccccc2Cl)c1	11	MST2	STK3	>	10000
UNC10225401A	OC(=O)c1cccc(NC2=C(C(=O)NC2=O)c2ccccc2Cl)c1	11	ULK3	ULK3	>	10000
UNC10225403A	C[C@@H]1CN(CCO1)c1cc(=O)n2nc(C)n(Cc3cccc(c3C)C(F)(F)F)c2n1	2	MYLK4	MYLK4	>	10000
UNC10225403A	C[C@@H]1CN(CCO1)c1cc(=O)n2nc(C)n(Cc3cccc(c3C)C(F)(F)F)c2n1	2	PIK3CB	PIK3CB	=	1.8
UNC10225404A	Cc1nnc(o1)-c1ccc(C)c(c1)-c1ccc(cc1)C(=O)NCC1CC1	1	p38-alpha	MAPK14	=	250
UNC10225404A	Cc1nnc(o1)-c1ccc(C)c(c1)-c1ccc(cc1)C(=O)NCC1CC1	1	PKN1	PKN1	>	10000
UNC10225406A	CN1C(=O)C(Nc2ccc(Cl)c(c2)C(O)=O)=C(C1=O)c1ccc(Cl)cc1	3	AAK1	AAK1	>	10000
UNC10225406A	CN1C(=O)C(Nc2ccc(Cl)c(c2)C(O)=O)=C(C1=O)c1ccc(Cl)cc1	3	CAMK1G	CAMK1G	>	10000
UNC10225406A	CN1C(=O)C(Nc2ccc(Cl)c(c2)C(O)=O)=C(C1=O)c1ccc(Cl)cc1	3	PKN1	PKN1	>	10000
UNC10225407A	CCn1c(nc2cnccc12)-c1ccccc1	1	CDC2L5	CDK13	>	10000
UNC10225410A	Nc1ncnc2n(cnc12)-c1cccc2ccccc12	3	LKB1	STK11	>	10000
UNC10225410A	Nc1ncnc2n(cnc12)-c1cccc2ccccc12	3	PCTK2	CDK17	>	10000
UNC10225410A	Nc1ncnc2n(cnc12)-c1cccc2ccccc12	3	YANK2	STK32B	>	10000
UNC10225413A	COc1ccc(cc1)-n1cnc2c(N)ncnc12	3	FLT3	FLT3	=	120
UNC10225413A	COc1ccc(cc1)-n1cnc2c(N)ncnc12	3	KIT	KIT	=	200
UNC10225413A	COc1ccc(cc1)-n1cnc2c(N)ncnc12	3	PDGFRB	PDGFRB	=	200
UNC10225415A	Cc1cn2c(nc(cc2=O)N2CCOCC2)n1Cc1cccc(Cl)c1Cl	4	PIK3CB	PIK3CB	=	2.4
UNC10225415A	Cc1cn2c(nc(cc2=O)N2CCOCC2)n1Cc1cccc(Cl)c1Cl	4	PIK3CD	PIK3CD	=	120
UNC10225415A	Cc1cn2c(nc(cc2=O)N2CCOCC2)n1Cc1cccc(Cl)c1Cl	4	VPS34	PIK3C3	=	2.2
UNC10225417A	CSc1nn2c(nc(cc2=O)N2CCOCC2)n1Cc1cccc(c1C)C(F)(F)F	10	ASK2	MAP3K6	>	10000
UNC10225417A	CSc1nn2c(nc(cc2=O)N2CCOCC2)n1Cc1cccc(c1C)C(F)(F)F	10	CSNK1G1	CSNK1G1	>	10000
UNC10225417A	CSc1nn2c(nc(cc2=O)N2CCOCC2)n1Cc1cccc(c1C)C(F)(F)F	10	GAK	GAK	>	10000
UNC10225417A	CSc1nn2c(nc(cc2=O)N2CCOCC2)n1Cc1cccc(c1C)C(F)(F)F	10	JAK2(JH1do	JAK2	>	10000
UNC10225417A	CSc1nn2c(nc(cc2=O)N2CCOCC2)n1Cc1cccc(c1C)C(F)(F)F	10	MST4	MST4	>	10000
UNC10225417A	CSc1nn2c(nc(cc2=O)N2CCOCC2)n1Cc1cccc(c1C)C(F)(F)F	10	NEK2	NEK2	>	10000
UNC10225417A	CSc1nn2c(nc(cc2=O)N2CCOCC2)n1Cc1cccc(c1C)C(F)(F)F	10	PIK3CB	PIK3CB	=	0.48
UNC10225417A	CSc1nn2c(nc(cc2=O)N2CCOCC2)n1Cc1cccc(c1C)C(F)(F)F	10	PIK3CD	PIK3CD	=	64
UNC10225417A	CSc1nn2c(nc(cc2=O)N2CCOCC2)n1Cc1cccc(c1C)C(F)(F)F	10	ROCK1	ROCK1	=	46
UNC10225417A	CSc1nn2c(nc(cc2=O)N2CCOCC2)n1Cc1cccc(c1C)C(F)(F)F	10	ROCK2	ROCK2	=	82
UNC10225417A	CSc1nn2c(nc(cc2=O)N2CCOCC2)n1Cc1cccc(c1C)C(F)(F)F	10	VPS34	PIK3C3	=	78
UNC10225418A	Fc1ccc(cc1)-c1nc[nH]c1-c1ccncc1	7	BUB1	BUB1	=	890
UNC10225418A	Fc1ccc(cc1)-c1nc[nH]c1-c1ccncc1	7	CIT	CIT	=	310
UNC10225418A	Fc1ccc(cc1)-c1nc[nH]c1-c1ccncc1	7	CSNK1A1	CSNK1A1	=	87
UNC10225418A	Fc1ccc(cc1)-c1nc[nH]c1-c1ccncc1	7	CSNK1D	CSNK1D	=	41

UNC10225418A	Fc1ccc(cc1)-c1nc[nH]c1-c1ccncc1	7	CSNK1E	CSNK1E	=	140
UNC10225418A	Fc1ccc(cc1)-c1nc[nH]c1-c1ccncc1	7	JNK2	MAPK9	=	85
UNC10225418A	Fc1ccc(cc1)-c1nc[nH]c1-c1ccncc1	7	JNK3	MAPK10	=	11
UNC10225418A	Fc1ccc(cc1)-c1nc[nH]c1-c1ccncc1	7	p38-alpha	MAPK14	=	67
UNC10225418A	Fc1ccc(cc1)-c1nc[nH]c1-c1ccncc1	7	RSK4(Kin.Dc	RPS6KA6	=	310
UNC10225421A	O=C(N1CCOCC1)c1ccc(cc1)-c1cc(ccn1)-c1c[nH]nc1-c1ccccn1	1	TGFBR1	TGFBR1	=	180
UNC10225422A	Cl.Fc1ccccc1COc1ccc(Nc2ncnc3ccc(cc23)-c2nnc(o2)C(F)(F)F)cc1Cl	5	EGFR	EGFR	=	43
UNC10225422A	Cl.Fc1ccccc1COc1ccc(Nc2ncnc3ccc(cc23)-c2nnc(o2)C(F)(F)F)cc1Cl	5	ERBB2	ERBB2	=	110
UNC10225424A	COc1ccc(Nc2nccc(n2)N2CCC(C2)NC(=O)Nc2cc(C)ccc2C)cc1	1	STK36	STK36	=	1600
UNC10225425A	FC(F)(F)c1ccc(cc1)-c1ncn(CCCN2CCOCC2)c1-c1ccncc1	0	FGFR1	FGFR1	>	10000
UNC10225425A	FC(F)(F)c1ccc(cc1)-c1ncn(CCCN2CCOCC2)c1-c1ccncc1	0	PRKCD	PRKCD	>	10000
UNC10225429A	Nc1nc(-c2nccs2)c(s1)-c1ccnc2ccccc12	2	AKT3	AKT3	>	10000
UNC10225429A	Nc1nc(-c2nccs2)c(s1)-c1ccnc2ccccc12	2	DMPK	DMPK	>	10000
UNC10225429A	Nc1nc(-c2nccs2)c(s1)-c1ccnc2ccccc12	2	RSK2(Kin.Dc	RPS6KA3	>	10000
UNC10225431A	COc1ccc(cc1)-c1oc2ncnc(N)c2c1-c1ccc(OC)cc1	5	DDR1	DDR1	=	650
UNC10225431A	COc1ccc(cc1)-c1oc2ncnc(N)c2c1-c1ccc(OC)cc1	5	LATS1	LATS1	>	10000
UNC10225431A	COc1ccc(cc1)-c1oc2ncnc(N)c2c1-c1ccc(OC)cc1	5	MEK5	MAP2K5	=	3000
UNC10225431A	COc1ccc(cc1)-c1oc2ncnc(N)c2c1-c1ccc(OC)cc1	5	TYK2(JH2do	TYK2	=	1600
UNC10225435A	CSc1nn2c(nc(cc2=O)N2CCOCC2)n1Cc1cccc(Cl)c1Cl	4	CLK3	CLK3	>	10000
UNC10225435A	CSc1nn2c(nc(cc2=O)N2CCOCC2)n1Cc1cccc(Cl)c1Cl	4	CSNK1E	CSNK1E	>	10000
UNC10225435A	CSc1nn2c(nc(cc2=O)N2CCOCC2)n1Cc1cccc(Cl)c1Cl	4	DLK	MAP3K12	>	10000
UNC10225435A	CSc1nn2c(nc(cc2=O)N2CCOCC2)n1Cc1cccc(Cl)c1Cl	4	PIK3C2B	PIK3C2B	=	360
UNC10225435A	CSc1nn2c(nc(cc2=O)N2CCOCC2)n1Cc1cccc(Cl)c1Cl	4	PIK3CB	PIK3CB	=	0.8
UNC10225435A	CSc1nn2c(nc(cc2=O)N2CCOCC2)n1Cc1cccc(Cl)c1Cl	4	PIK3CD	PIK3CD	=	240
UNC10225435A	CSc1nn2c(nc(cc2=O)N2CCOCC2)n1Cc1cccc(Cl)c1Cl	4	VPS34	PIK3C3	=	0.72
UNC10225439A	O=c1cc(nc2n(Cc3cccc4ccccc34)ccn12)N1CCOCC1	2	PIK3CB	PIK3CB	=	2.9
UNC10225439A	O=c1cc(nc2n(Cc3cccc4ccccc34)ccn12)N1CCOCC1	2	PIK3CD	PIK3CD	=	150
UNC10225439A	O=c1cc(nc2n(Cc3cccc4ccccc34)ccn12)N1CCOCC1	2	RET	RET	>	10000
UNC10225439A	O=c1cc(nc2n(Cc3cccc4ccccc34)ccn12)N1CCOCC1	2	VPS34	PIK3C3	=	60
UNC10225440A	Cl.Fc1cccc(F)c1C(=O)Nc1cccc(c1)-c1nn2ncccc2c1-c1ccnc(Nc2cccnc2)n1	13	BRAF	BRAF	=	62
UNC10225440A	Cl.Fc1cccc(F)c1C(=O)Nc1cccc(c1)-c1nn2ncccc2c1-c1ccnc(Nc2cccnc2)n1	13	DDR1	DDR1	=	140
UNC10225440A	Cl.Fc1cccc(F)c1C(=O)Nc1cccc(c1)-c1nn2ncccc2c1-c1ccnc(Nc2cccnc2)n1	13	EGFR	EGFR	=	38
UNC10225440A	Cl.Fc1cccc(F)c1C(=O)Nc1cccc(c1)-c1nn2ncccc2c1-c1ccnc(Nc2cccnc2)n1	13	ERBB2	ERBB2	=	29
UNC10225440A	Cl.Fc1cccc(F)c1C(=O)Nc1cccc(c1)-c1nn2ncccc2c1-c1ccnc(Nc2cccnc2)n1	13	ERBB4	ERBB4	=	64
UNC10225440A	Cl.Fc1cccc(F)c1C(=O)Nc1cccc(c1)-c1nn2ncccc2c1-c1ccnc(Nc2cccnc2)n1	13	MEK5	MAP2K5	=	230
UNC10225440A	Cl.Fc1cccc(F)c1C(=O)Nc1cccc(c1)-c1nn2ncccc2c1-c1ccnc(Nc2cccnc2)n1	13	RAF1	RAF1	=	430
UNC10225440A	Cl.Fc1cccc(F)c1C(=O)Nc1cccc(c1)-c1nn2ncccc2c1-c1ccnc(Nc2cccnc2)n1	13	STK36	STK36	=	98
UNC10225443A	CN1CCC(CC1)n1cnc(c1-c1ccnc(NC2CCNCC2)n1)-c1ccc(F)cc1	6	CSNK1A1	CSNK1A1	=	160
UNC10225443A	CN1CCC(CC1)n1cnc(c1-c1ccnc(NC2CCNCC2)n1)-c1ccc(F)cc1	6	CSNK1D	CSNK1D	=	89
UNC10225443A	CN1CCC(CC1)n1cnc(c1-c1ccnc(NC2CCNCC2)n1)-c1ccc(F)cc1	6	CSNK1E	CSNK1E	=	210

UNC10225443A	CN1CCC(CC1)n1cnc(c1-c1ccnc(NC2CCNCC2)n1)-c1ccc(F)cc1	6	JNK2	MAPK9	=	170
UNC10225443A	CN1CCC(CC1)n1cnc(c1-c1ccnc(NC2CCNCC2)n1)-c1ccc(F)cc1	6	JNK3	MAPK10	=	140
UNC10225443A	CN1CCC(CC1)n1cnc(c1-c1ccnc(NC2CCNCC2)n1)-c1ccc(F)cc1	6	p38-alpha	MAPK14	=	220
UNC10225443A	CN1CCC(CC1)n1cnc(c1-c1ccnc(NC2CCNCC2)n1)-c1ccc(F)cc1	6	PIP5K2C	PIP4K2C	=	220
UNC10225443A	CN1CCC(CC1)n1cnc(c1-c1ccnc(NC2CCNCC2)n1)-c1ccc(F)cc1	6	SGK2	SGK2	>	10000
UNC10225444A	O[C@H]([C@@H](O)C(O)=O)C(O)=O.Brc1cc2c(NC(=O)c3ccc(CN4CCCC4)cc3)n[nH]c2nc1-c1ccco1	9	EPHA4	EPHA4	>	10000
UNC10225444A	O[C@H]([C@@H](O)C(O)=O)C(O)=O.Brc1cc2c(NC(=O)c3ccc(CN4CCCC4)cc3)n[nH]c2nc1-c1ccco1	9	EPHA6	EPHA6	>	10000
UNC10225444A	O[C@H]([C@@H](O)C(O)=O)C(O)=O.Brc1cc2c(NC(=O)c3ccc(CN4CCCC4)cc3)n[nH]c2nc1-c1ccco1	9	EPHB6	EPHB6	=	160
UNC10225444A	O[C@H]([C@@H](O)C(O)=O)C(O)=O.Brc1cc2c(NC(=O)c3ccc(CN4CCCC4)cc3)n[nH]c2nc1-c1ccco1	9	FLT3	FLT3	=	45
UNC10225444A	O[C@H]([C@@H](O)C(O)=O)C(O)=O.Brc1cc2c(NC(=O)c3ccc(CN4CCCC4)cc3)n[nH]c2nc1-c1ccco1	9	KIT	KIT	=	66
UNC10225444A	O[C@H]([C@@H](O)C(O)=O)C(O)=O.Brc1cc2c(NC(=O)c3ccc(CN4CCCC4)cc3)n[nH]c2nc1-c1ccco1	9	MEK5	MAP2K5	=	550
UNC10225444A	O[C@H]([C@@H](O)C(O)=O)C(O)=O.Brc1cc2c(NC(=O)c3ccc(CN4CCCC4)cc3)n[nH]c2nc1-c1ccco1	9	MYLK	MYLK	=	1300
UNC10225444A	O[C@H]([C@@H](O)C(O)=O)C(O)=O.Brc1cc2c(NC(=O)c3ccc(CN4CCCC4)cc3)n[nH]c2nc1-c1ccco1	9	MYLK2	MYLK2	=	120
UNC10225444A	O[C@H]([C@@H](O)C(O)=O)C(O)=O.Brc1cc2c(NC(=O)c3ccc(CN4CCCC4)cc3)n[nH]c2nc1-c1ccco1	9	PDGFRB	PDGFRB	=	67
UNC10225445A	COc1ccc(NC(=O)Nc2ccc(C)c(c2)-c2ccc(cc2)C(=O)Nc2ccncc2)cc1	6	CSF1R	CSF1R	=	1800
UNC10225445A	COc1ccc(NC(=O)Nc2ccc(C)c(c2)-c2ccc(cc2)C(=O)Nc2ccncc2)cc1	6	DDR1	DDR1	=	860
UNC10225445A	COc1ccc(NC(=O)Nc2ccc(C)c(c2)-c2ccc(cc2)C(=O)Nc2ccncc2)cc1	6	FLT3	FLT3	=	2200
UNC10225445A	COc1ccc(NC(=O)Nc2ccc(C)c(c2)-c2ccc(cc2)C(=O)Nc2ccncc2)cc1	6	KIT	KIT	=	44
UNC10225445A	COc1ccc(NC(=O)Nc2ccc(C)c(c2)-c2ccc(cc2)C(=O)Nc2ccncc2)cc1	6	PDGFRB	PDGFRB	=	48
UNC10225448A	COc1cccc(c1)-c1cc(ccn1)-c1c[nH]nc1-c1ccccn1	4	AURKC	AURKC	>	10000
UNC10225448A	COc1cccc(c1)-c1cc(ccn1)-c1c[nH]nc1-c1ccccn1	4	FLT4	FLT4	>	10000
UNC10225448A	COc1cccc(c1)-c1cc(ccn1)-c1c[nH]nc1-c1ccccn1	4	MAK	MAK	>	10000
UNC10225448A	COc1cccc(c1)-c1cc(ccn1)-c1c[nH]nc1-c1ccccn1	4	RIOK3	RIOK3	=	9600
UNC10225448A	COc1cccc(c1)-c1cc(ccn1)-c1c[nH]nc1-c1ccccn1	4	SRPK1	SRPK1	>	10000
UNC10225449A	Cl.COc1cc2ncnc(Nc3cccc(Br)c3)c2cc1OC	13	BUB1	BUB1	=	130
UNC10225449A	Cl.COc1cc2ncnc(Nc3cccc(Br)c3)c2cc1OC	13	EGFR	EGFR	=	0.32
UNC10225449A	Cl.COc1cc2ncnc(Nc3cccc(Br)c3)c2cc1OC	13	ERBB2	ERBB2	=	85
UNC10225449A	Cl.COc1cc2ncnc(Nc3cccc(Br)c3)c2cc1OC	13	GAK	GAK	=	29
UNC10225449A	Cl.COc1cc2ncnc(Nc3cccc(Br)c3)c2cc1OC	13	MEK5	MAP2K5	=	700
UNC10225450A	COc1cc2ncn(-c3cc(OCCCc4ccccc4)c(s3)C(N)=O)c2cc1OC	4	MEK5	MAP2K5	=	280
UNC10225450A	COc1cc2ncn(-c3cc(OCCCc4ccccc4)c(s3)C(N)=O)c2cc1OC	4	PLK1	PLK1	=	18
UNC10225450A	COc1cc2ncn(-c3cc(OCCCc4ccccc4)c(s3)C(N)=O)c2cc1OC	4	PLK2	PLK2	=	34
UNC10225450A	COc1cc2ncn(-c3cc(OCCCc4ccccc4)c(s3)C(N)=O)c2cc1OC	4	PLK3	PLK3	=	120
UNC10225454A	COc1cccc(c1)-n1cnc2c(N)ncnc12	1	MEK5	MAP2K5	=	1300
UNC10225454A	COc1cccc(c1)-n1cnc2c(N)ncnc12	1	TYK2(JH1do	TYK2	>	10000
UNC10225455A	Brc1cc2c(NC(=O)C3CC3)n[nH]c2nc1-c1cccs1	11	CAMK2A	CAMK2A	>	10000

UNC10225455A	Brc1cc2c(NC(=O)C3CC3)n[nH]c2nc1-c1cccs1	11	CAMK2B	CAMK2B	>	10000
UNC10225455A	Brc1cc2c(NC(=O)C3CC3)n[nH]c2nc1-c1cccs1	11	CDKL2	CDKL2	=	140
UNC10225455A	Brc1cc2c(NC(=O)C3CC3)n[nH]c2nc1-c1cccs1	11	ERK8	MAPK15	=	130
UNC10225455A	Brc1cc2c(NC(=O)C3CC3)n[nH]c2nc1-c1cccs1	11	FLT3	FLT3	=	150
UNC10225455A	Brc1cc2c(NC(=O)C3CC3)n[nH]c2nc1-c1cccs1	11	GSK3A	GSK3A	=	91
UNC10225455A	Brc1cc2c(NC(=O)C3CC3)n[nH]c2nc1-c1cccs1	11	MEK4	MAP2K4	>	10000
UNC10225455A	Brc1cc2c(NC(=O)C3CC3)n[nH]c2nc1-c1cccs1	11	MLCK	MYLK3	=	180
UNC10225455A	Brc1cc2c(NC(=O)C3CC3)n[nH]c2nc1-c1cccs1	11	MYLK2	MYLK2	=	8.4
UNC10225455A	Brc1cc2c(NC(=O)C3CC3)n[nH]c2nc1-c1cccs1	11	RIPK2	RIPK2	>	10000
UNC10225455A	Brc1cc2c(NC(=O)C3CC3)n[nH]c2nc1-c1cccs1	11	RSK4(Kin.Dc	RPS6KA6	=	3800
UNC10225455A	Brc1cc2c(NC(=O)C3CC3)n[nH]c2nc1-c1cccs1	11	TNNI3K	TNNI3K	>	10000
UNC10225455A	Brc1cc2c(NC(=O)C3CC3)n[nH]c2nc1-c1cccs1	11	YSK4	MAP3K19	=	200
UNC10225462A	O=S(=O)(N1CCCC1)c1ccc(cc1)-c1ccnc2[nH]ccc12	1	BIKE	BMP2K	=	7200
UNC10225462A	O=S(=O)(N1CCCC1)c1ccc(cc1)-c1ccnc2[nH]ccc12	1	IKK-beta	IKBKB	=	270
UNC10225462A	O=S(=O)(N1CCCC1)c1ccc(cc1)-c1ccnc2[nH]ccc12	1	JAK2(JH1do	JAK2	=	360
UNC10225462A	O=S(=O)(N1CCCC1)c1ccc(cc1)-c1ccnc2[nH]ccc12	1	PKAC-alpha	PRKACA	=	260
UNC10225462A	O=S(=O)(N1CCCC1)c1ccc(cc1)-c1ccnc2[nH]ccc12	1	RIOK1	RIOK1	=	610
UNC10225462A	O=S(=O)(N1CCCC1)c1ccc(cc1)-c1ccnc2[nH]ccc12	1	RIOK2	RIOK2	=	960
UNC10225462A	O=S(=O)(N1CCCC1)c1ccc(cc1)-c1ccnc2[nH]ccc12	1	RIOK3	RIOK3	=	540
UNC10225462A	O=S(=O)(N1CCCC1)c1ccc(cc1)-c1ccnc2[nH]ccc12	1	ROCK1	ROCK1	=	330
UNC10225462A	O=S(=O)(N1CCCC1)c1ccc(cc1)-c1ccnc2[nH]ccc12	1	ROCK2	ROCK2	=	350
UNC10225463A	Cl.Cl.C1CC(CCN1)Oc1cc2cnccc2cc1-c1ccccc1	6	DAPK1	DAPK1	=	300
UNC10225463A	Cl.Cl.C1CC(CCN1)Oc1cc2cnccc2cc1-c1ccccc1	6	DAPK3	DAPK3	=	170
UNC10225463A	Cl.Cl.C1CC(CCN1)Oc1cc2cnccc2cc1-c1ccccc1	6	HASPIN	GSG2	=	90
UNC10225463A	Cl.Cl.C1CC(CCN1)Oc1cc2cnccc2cc1-c1ccccc1	6	IKK-beta	IKBKB	=	24
UNC10225463A	Cl.Cl.C1CC(CCN1)Oc1cc2cnccc2cc1-c1ccccc1	6	PRKCE	PRKCE	=	640
UNC10225463A	Cl.Cl.C1CC(CCN1)Oc1cc2cnccc2cc1-c1ccccc1	6	PRKCH	PRKCH	=	390
UNC10225463A	Cl.Cl.C1CC(CCN1)Oc1cc2cnccc2cc1-c1ccccc1	6	ROCK1	ROCK1	=	130
UNC10225463A	Cl.Cl.C1CC(CCN1)Oc1cc2cnccc2cc1-c1ccccc1	6	ROCK2	ROCK2	=	180
UNC10225463A	Cl.Cl.C1CC(CCN1)Oc1cc2cnccc2cc1-c1ccccc1	6	STK33	STK33	>	10000
UNC10225463A	Cl.Cl.C1CC(CCN1)Oc1cc2cnccc2cc1-c1ccccc1	6	STK36	STK36	>	10000
UNC10225464A	Clc1ccc(cc1)C1=C(Nc2cccc(Cl)c2)C(=O)NC1=O	6	CDK4	CDK4	>	10000
UNC10225464A	Clc1ccc(cc1)C1=C(Nc2cccc(Cl)c2)C(=O)NC1=O	6	CLK1	CLK1	=	4400
UNC10225464A	Clc1ccc(cc1)C1=C(Nc2cccc(Cl)c2)C(=O)NC1=O	6	CSNK1G3	CSNK1G3	>	10000
UNC10225464A	Clc1ccc(cc1)C1=C(Nc2cccc(Cl)c2)C(=O)NC1=O	6	PIK3CB	PIK3CB	=	160
UNC10225464A	Clc1ccc(cc1)C1=C(Nc2cccc(Cl)c2)C(=O)NC1=O	6	PKAC-beta	PRKACB	>	10000
UNC10225464A	Clc1ccc(cc1)C1=C(Nc2cccc(Cl)c2)C(=O)NC1=O	6	RPS6KA4(Ki	RPS6KA4	>	10000
UNC10225464A	Clc1ccc(cc1)C1=C(Nc2cccc(Cl)c2)C(=O)NC1=O	6	VPS34	PIK3C3	=	300
UNC10225464A	Clc1ccc(cc1)C1=C(Nc2cccc(Cl)c2)C(=O)NC1=O	6	VRK2	VRK2	>	10000
UNC10225468A	Nc1n[nH]c2ncc(cc12)-c1ccccc1	2	KIT	KIT	=	92
UNC10225468A	Nc1n[nH]c2ncc(cc12)-c1ccccc1	2	MEK5	MAP2K5	=	660
UNC10225468A	Nc1n[nH]c2ncc(cc12)-c1ccccc1	2	MKNK2	MKNK2	=	260
UNC10225468A	Nc1n[nH]c2ncc(cc12)-c1ccccc1	2	PDGFRB	PDGFRB	=	200
UNC10225468A	Nc1n[nH]c2ncc(cc12)-c1ccccc1	2	TYK2(JH2do	TYK2	=	950
UNC10225473A	CN(C1CCOCC1)C(=O)c1ccc(cc1)-c1cc(ccn1)-c1c[nH]nc1-c1ccccn1	1	VRK2	VRK2	>	10000
UNC10225474A	CCn1c(nc2cnccc12)-c1ccoc1	6	GRK7	GRK7	>	10000
UNC10225474A	CCn1c(nc2cnccc12)-c1ccoc1	6	INSR	INSR	>	10000
UNC10225474A	CCn1c(nc2cnccc12)-c1ccoc1	6	TAK1	MAP3K7	>	10000
UNC10225475A	COc1ccc(NC(=O)c2ccc(cc2)-c2cc(ccc2C)-c2nnc(C)o2)cc1	3	CLK3	CLK3	>	10000
UNC10225475A	COc1ccc(NC(=O)c2ccc(cc2)-c2cc(ccc2C)-c2nnc(C)o2)cc1	3	GRK3	ADRBK2	>	10000
UNC10225475A	COc1ccc(NC(=O)c2ccc(cc2)-c2cc(ccc2C)-c2nnc(C)o2)cc1	3	p38-alpha	MAPK14	=	180
UNC10225477A	Cc1cccc(n1)-c1nc(Nc2ccc3ncsc3c2)c2ccccc2n1	2	PRKD1	PRKD1	=	16
UNC10225477A	Cc1cccc(n1)-c1nc(Nc2ccc3ncsc3c2)c2ccccc2n1	2	PRKD2	PRKD2	=	22
UNC10225480A	Cc1cccc(n1)-c1nc(Nc2ccc3n(C)ncc3c2)c2ccccc2n1	6	MAST1	MAST1	>	10000

UNC10225480A	Cc1cccc(n1)-c1nc(Nc2ccc3n(C)ncc3c2)c2ccccc2n1	6	MKNK1	MKNK1	>	10000
UNC10225480A	Cc1cccc(n1)-c1nc(Nc2ccc3n(C)ncc3c2)c2ccccc2n1	6	PIKFYVE	PIKFYVE	=	380
UNC10225480A	Cc1cccc(n1)-c1nc(Nc2ccc3n(C)ncc3c2)c2ccccc2n1	6	PRKD1	PRKD1	=	7.2
UNC10225480A	Cc1cccc(n1)-c1nc(Nc2ccc3n(C)ncc3c2)c2ccccc2n1	6	PRKD2	PRKD2	=	12
UNC10225480A	Cc1cccc(n1)-c1nc(Nc2ccc3n(C)ncc3c2)c2ccccc2n1	6	PRKD3	PRKD3	=	13
UNC10225482A	Cl.Oc1ccc(cc1)-c1nc([nH]c1-c1ccncc1)-c1ccccc1	4	ADCK4	ADCK4	>	10000
UNC10225482A	Cl.Oc1ccc(cc1)-c1nc([nH]c1-c1ccncc1)-c1ccccc1	4	BUB1	BUB1	=	560
UNC10225482A	Cl.Oc1ccc(cc1)-c1nc([nH]c1-c1ccncc1)-c1ccccc1	4	TYK2(JH1do	TYK2	>	10000
UNC10225486A	CSc1ccc(cc1)-c1nc2CCCn2c1-c1ccncc1	0	BUB1	BUB1	=	260
UNC10225488A	Cl.C(c1ccccc1)n1ccc2cc(Nc3ncnc4ccccc34)ccc12	9	EGFR	EGFR	=	5
UNC10225488A	Cl.C(c1ccccc1)n1ccc2cc(Nc3ncnc4ccccc34)ccc12	9	ERBB2	ERBB2	=	14
UNC10225488A	Cl.C(c1ccccc1)n1ccc2cc(Nc3ncnc4ccccc34)ccc12	9	ERBB4	ERBB4	=	140
UNC10225488A	Cl.C(c1ccccc1)n1ccc2cc(Nc3ncnc4ccccc34)ccc12	9	JAK2(JH1do	JAK2	>	10000
UNC10225488A	Cl.C(c1ccccc1)n1ccc2cc(Nc3ncnc4ccccc34)ccc12	9	MEK5	MAP2K5	=	770
UNC10225489A	C[C@@H]1CC[C@@H](CN1c1cc(nc(N)n1)-c1ccc2c(N)n[nH]c2c1)C(=O)NC1CCCCC1	7	EPHB6	EPHB6	=	49
UNC10225489A	C[C@@H]1CC[C@@H](CN1c1cc(nc(N)n1)-c1ccc2c(N)n[nH]c2c1)C(=O)NC1CCCCC1	7	PDPK1	PDPK1	=	2.8
UNC10225489A	C[C@@H]1CC[C@@H](CN1c1cc(nc(N)n1)-c1ccc2c(N)n[nH]c2c1)C(=O)NC1CCCCC1	7	PIP5K1A	PIP5K1A	=	440
UNC10225489A	C[C@@H]1CC[C@@H](CN1c1cc(nc(N)n1)-c1ccc2c(N)n[nH]c2c1)C(=O)NC1CCCCC1	7	PRKX	PRKX	=	460
UNC10225489A	C[C@@H]1CC[C@@H](CN1c1cc(nc(N)n1)-c1ccc2c(N)n[nH]c2c1)C(=O)NC1CCCCC1	7	RIOK1	RIOK1	=	210
UNC10225489A	C[C@@H]1CC[C@@H](CN1c1cc(nc(N)n1)-c1ccc2c(N)n[nH]c2c1)C(=O)NC1CCCCC1	7	RIOK3	RIOK3	=	150
UNC10225489A	C[C@@H]1CC[C@@H](CN1c1cc(nc(N)n1)-c1ccc2c(N)n[nH]c2c1)C(=O)NC1CCCCC1	7	ROCK1	ROCK1	=	210
UNC10225489A	C[C@@H]1CC[C@@H](CN1c1cc(nc(N)n1)-c1ccc2c(N)n[nH]c2c1)C(=O)NC1CCCCC1	7	ROCK2	ROCK2	=	150
UNC10225491A	Cc1ccccc1COc1cc(sc1C(N)=O)-n1cnc2cc(Cl)ccc12	3	LRRK2	LRRK2	>	10000
UNC10225491A	Cc1ccccc1COc1cc(sc1C(N)=O)-n1cnc2cc(Cl)ccc12	3	PLK1	PLK1	=	19
UNC10225491A	Cc1ccccc1COc1cc(sc1C(N)=O)-n1cnc2cc(Cl)ccc12	3	PLK2	PLK2	=	68
UNC10225491A	Cc1ccccc1COc1cc(sc1C(N)=O)-n1cnc2cc(Cl)ccc12	3	PLK3	PLK3	=	99
UNC10225491A	Cc1ccccc1COc1cc(sc1C(N)=O)-n1cnc2cc(Cl)ccc12	3	VPS34	PIK3C3	>	10000
UNC10225492A	Fc1ccc(Nc2nccc(NCCNC(=O)Nc3ccc4OCOc4c3)n2)cc1	4	JAK2(JH1do	JAK2	=	2000
UNC10225492A	Fc1ccc(Nc2nccc(NCCNC(=O)Nc3ccc4OCOc4c3)n2)cc1	4	TESK1	TESK1	>	10000
UNC10225492A	Fc1ccc(Nc2nccc(NCCNC(=O)Nc3ccc4OCOc4c3)n2)cc1	4	TXK	TXK	>	10000
UNC10225492A	Fc1ccc(Nc2nccc(NCCNC(=O)Nc3ccc4OCOc4c3)n2)cc1	4	ZAK	ZAK	>	10000
UNC10225493A	Cl.Cc1nccc(n1)-c1c(ncn1CCCN1CCOCC1)-c1ccc(F)cc1	9	CDC2L1	CDK11B	>	10000
UNC10225493A	Cl.Cc1nccc(n1)-c1c(ncn1CCCN1CCOCC1)-c1ccc(F)cc1	9	CDK9	CDK9	>	10000
UNC10225493A	Cl.Cc1nccc(n1)-c1c(ncn1CCCN1CCOCC1)-c1ccc(F)cc1	9	CSNK1D	CSNK1D	=	75
UNC10225493A	Cl.Cc1nccc(n1)-c1c(ncn1CCCN1CCOCC1)-c1ccc(F)cc1	9	CSNK1E	CSNK1E	=	460
UNC10225493A	Cl.Cc1nccc(n1)-c1c(ncn1CCCN1CCOCC1)-c1ccc(F)cc1	9	DAPK2	DAPK2	>	10000
UNC10225493A	Cl.Cc1nccc(n1)-c1c(ncn1CCCN1CCOCC1)-c1ccc(F)cc1	9	ERK4	MAPK4	>	10000
UNC10225493A	Cl.Cc1nccc(n1)-c1c(ncn1CCCN1CCOCC1)-c1ccc(F)cc1	9	PHKG2	PHKG2	>	10000
UNC10225493A	Cl.Cc1nccc(n1)-c1c(ncn1CCCN1CCOCC1)-c1ccc(F)cc1	9	RPS6KA4(Ki	RPS6KA4	>	10000
UNC10225493A	Cl.Cc1nccc(n1)-c1c(ncn1CCCN1CCOCC1)-c1ccc(F)cc1	9	TIE2	TEK	>	10000
UNC10225494A	Nc1nccc2scc(-c3ccc4N(CCc4c3)C(=O)Cc3cc(F)ccc3F)c12	1	CLK3	CLK3	>	10000
UNC10225494A	Nc1nccc2scc(-c3ccc4N(CCc4c3)C(=O)Cc3cc(F)ccc3F)c12	1	RIPK1	RIPK1	=	570
UNC10225497A	Cn1cncc1-c1ccc2ncnc(Nc3ccc(OCc4ccccc4)cc3)c2c1	12	AURKB	AURKB	>	10000
UNC10225497A	Cn1cncc1-c1ccc2ncnc(Nc3ccc(OCc4ccccc4)cc3)c2c1	12	CLK3	CLK3	>	10000
UNC10225497A	Cn1cncc1-c1ccc2ncnc(Nc3ccc(OCc4ccccc4)cc3)c2c1	12	CSNK1E	CSNK1E	>	10000
UNC10225497A	Cn1cncc1-c1ccc2ncnc(Nc3ccc(OCc4ccccc4)cc3)c2c1	12	EGFR	EGFR	=	49
UNC10225497A	Cn1cncc1-c1ccc2ncnc(Nc3ccc(OCc4ccccc4)cc3)c2c1	12	EIF2AK1	EIF2AK1	>	10000

UNC10225497A	Cn1cncc1-c1ccc2ncnc(Nc3ccc(OCc4ccccc4)cc3)c2c1	12	ERBB2	ERBB2	=	41
UNC10225497A	Cn1cncc1-c1ccc2ncnc(Nc3ccc(OCc4ccccc4)cc3)c2c1	12	MEK5	MAP2K5	=	450
UNC10225497A	Cn1cncc1-c1ccc2ncnc(Nc3ccc(OCc4ccccc4)cc3)c2c1	12	RIPK2	RIPK2	=	91
UNC10225497A	Cn1cncc1-c1ccc2ncnc(Nc3ccc(OCc4ccccc4)cc3)c2c1	12	SNRK	SNRK	=	8700
UNC10225497A	Cn1cncc1-c1ccc2ncnc(Nc3ccc(OCc4ccccc4)cc3)c2c1	12	YANK1	STK32A	>	10000
UNC10225499A	Cc1cccc(c1)-c1cc(ccn1)-c1c[nH]nc1-c1ccccn1	1	GAK	GAK	=	130
UNC10225501A	Cc1nnc(o1)-c1ccc(C)c(c1)-c1ccc(cc1)C(O)=O	1	LOK	STK10	>	10000
UNC10225502A	Clc1cccc(Cl)c1C(=O)c1c[nH]c(c1)C(=O)NCc1cccs1	1	FAK	PTK2	>	10000
UNC10225504A	Cl.COc1cc2ncnc(Nc3ccc4nc([nH]c4c3)-c3ccccc3)c2cc1OC	5	AURKA	AURKA	=	2300
UNC10225504A	Cl.COc1cc2ncnc(Nc3ccc4nc([nH]c4c3)-c3ccccc3)c2cc1OC	5	CSNK2A2	CSNK2A2	>	10000
UNC10225504A	Cl.COc1cc2ncnc(Nc3ccc4nc([nH]c4c3)-c3ccccc3)c2cc1OC	5	INSR	INSR	>	10000
UNC10225504A	Cl.COc1cc2ncnc(Nc3ccc4nc([nH]c4c3)-c3ccccc3)c2cc1OC	5	MEK5	MAP2K5	=	670
UNC10225504A	Cl.COc1cc2ncnc(Nc3ccc4nc([nH]c4c3)-c3ccccc3)c2cc1OC	5	PLK4	PLK4	=	1600
UNC10243860A	[2H]C([2H])(c1cccc(c1C)C(F)(F)F)n1ccn2c1cc(nc2=O)N1CCOCC1	1	PIK3CB	PIK3CB	=	51
UNC10243861A	Cc1c(Cn2ccn3c2nc(cc3=O)N2CCOCC2)cccc1C(F)(F)F	3	AKT3	AKT3	>	10000
UNC10243861A	Cc1c(Cn2ccn3c2nc(cc3=O)N2CCOCC2)cccc1C(F)(F)F	3	CAMK2A	CAMK2A	>	10000
UNC10243861A	Cc1c(Cn2ccn3c2nc(cc3=O)N2CCOCC2)cccc1C(F)(F)F	3	JAK1(JH1do	JAK1	>	10000
UNC10243861A	Cc1c(Cn2ccn3c2nc(cc3=O)N2CCOCC2)cccc1C(F)(F)F	3	PIK3CB	PIK3CB	=	1.5
UNC10243861A	Cc1c(Cn2ccn3c2nc(cc3=O)N2CCOCC2)cccc1C(F)(F)F	3	PIK3CD	PIK3CD	=	170
UNC10243861A	Cc1c(Cn2ccn3c2nc(cc3=O)N2CCOCC2)cccc1C(F)(F)F	3	VPS34	PIK3C3	=	64
UNC10243862A	CNc1ncnc2[nH]ncc12	1	ROCK1	ROCK1	=	380
UNC10243862A	CNc1ncnc2[nH]ncc12	1	ROCK2	ROCK2	=	330
UNC10243863A	Nc1ncnc2[nH]c(nc12)-c1ccccc1	11	AURKA	AURKA	>	10000
UNC10243863A	Nc1ncnc2[nH]c(nc12)-c1ccccc1	11	CSNK2A2	CSNK2A2	=	1100
UNC10243863A	Nc1ncnc2[nH]c(nc12)-c1ccccc1	11	EIF2AK1	EIF2AK1	>	10000
UNC10243863A	Nc1ncnc2[nH]c(nc12)-c1ccccc1	11	LTK	LTK	>	10000
UNC10243863A	Nc1ncnc2[nH]c(nc12)-c1ccccc1	11	MST1	STK4	>	10000
UNC10243863A	Nc1ncnc2[nH]c(nc12)-c1ccccc1	11	PAK3	PAK3	>	10000
UNC10243863A	Nc1ncnc2[nH]c(nc12)-c1ccccc1	11	PLK4	PLK4	>	10000
UNC10243863A	Nc1ncnc2[nH]c(nc12)-c1ccccc1	11	SNRK	SNRK	>	10000
UNC10243863A	Nc1ncnc2[nH]c(nc12)-c1ccccc1	11	TYK2(JH2do	TYK2	=	96
UNC10243863A	Nc1ncnc2[nH]c(nc12)-c1ccccc1	11	YANK1	STK32A	>	10000
UNC10243863A	Nc1ncnc2[nH]c(nc12)-c1ccccc1	11	YSK4	MAP3K19	=	270
UNC10243881A	CSc1nn2c(nc(cc2=O)N2CCOCC2)n1Cc1cccc(c1C)C(F)(F)F	3	PIK3CB	PIK3CB	=	0.26
UNC10243881A	CSc1nn2c(nc(cc2=O)N2CCOCC2)n1Cc1cccc(c1C)C(F)(F)F	3	PIK3CD	PIK3CD	=	47
UNC10243881A	CSc1nn2c(nc(cc2=O)N2CCOCC2)n1Cc1cccc(c1C)C(F)(F)F	3	VPS34	PIK3C3	=	33
