# Supplementary material for: Progress towards a public chemogenomic set for protein kinases and a call for contributions
Source: PLoS One. 2017 Aug 2;12(8):e0181585. doi: 10.1371/journal.pone.0181585 (PMC5540273; doi:10.1371/journal.pone.0181585)
Supplement: S5 Table — (PDF) [file pone.0181585.s005.pdf]

CompoundName	other name	Over_90%_1000nM	Meets S(35) <0.04
PFE-PKIS 40		11	Y
PFE-PKIS 29		8	Y
PFE-PKIS 35		7	Y
UNC10225101A		6	Y
UNC10225200A		11	Y
PFE-PKIS 39		11	Y
TPKI-69		13	Y
UNC10225353A		13	Y
UNC10225440A		13	Y
UNC10224990A		12	Y
UNC10225019A		12	Y
UNC10225035A		12	Y
UNC10225497A		12	Y
UNC10225455A		11	Y
UNC10243863A		11	Y
PFE-PKIS 25		10	Y
TPKI-64		10	Y
UNC10225233A		10	Y
UNC10225417A		10	Y
PFE-PKIS 34		9	Y
TPKI-110		9	Y
TPKI-61		9	Y
TPKI-66		9	Y
UNC10225058A		9	Y
UNC10225271A		9	Y
UNC10225372A		9	Y
UNC10225493A		9	Y
TPKI-39		8	Y
PFE-PKIS 32		8	Y
PFE-PKIS 41		8	Y
TPKI-65		8	Y
TPKI-73		8	Y
TPKI-98		8	Y
UNC10225163A		8	Y
UNC10225263A		8	Y
UNC10225359A		8	Y
GSK350559		8	Y
PFE-PKIS 3		7	Y
UNC10225116A		7	Y
UNC10225489A		7	Y
GW857175		7	Y

UNC10225279B	GW582764	7	Y
UNC10225418A	SB-217360	7	Y
UNC10225444A	SB-742352	7	Y
TPKI-24		6	Y
TPKI-27		6	Y
TPKI-38		6	Y
UNC10224985A	GSK1398467	6	Y
UNC10225008A	GSK223675	6	Y
UNC10225042A	GSK204919	6	Y
UNC10225105A	GSK1379762	6	Y
UNC10225115A	GW569716	6	Y
UNC10225148A	GSK2286096	6	Y
UNC10225274A	SB-222903	6	Y
UNC10225480A	GW876731	6	Y
PFE-PKIS 2		6	Y
PFE-PKIS 44		6	Y
TPKI-105		6	Y
TPKI-107		6	Y
TPKI-58		6	Y
TPKI-62		6	Y
TPKI-92		6	Y
TPKI-95		6	Y
UNC10225134A		6	Y
UNC10225182A		6	Y
UNC10225259A		6	Y
UNC10225300A		6	Y
UNC10225331A		6	Y
UNC10225443A		6	Y
UNC10225463A		6	Y
PFE-PKIS 9		5	Y
TPKI-63		5	Y
TPKI-72		5	Y
TPKI-99		5	Y
UNC10225006A		5	Y
UNC10225050A		5	Y
UNC10225256A		5	Y
UNC10225387B		5	Y
UNC10224992A	GSK1398471	5	Y
UNC10225013A	GW440132	5	Y
UNC10225091A	GW583340	5	Y
UNC10225258A	SB-223132	5	Y
UNC10225301A	GW560459	5	Y

UNC10225327A	GW855857	5	Y
UNC10225386A	GW854278	5	Y
UNC10112604A	GW574783	4	Y
UNC10225052A	GSK1379761	4	Y
UNC10225069A	GSK336313	4	Y
UNC10225085A	GW273749	4	Y
UNC10225141A	GW525701	4	Y
UNC10225167A	GSK2286295	4	Y
UNC10225205A	GSK1645895	4	Y
UNC10225225A	GSK2328680	4	Y
UNC10225227A	SB-284851	4	Y
UNC10225230A	GW792479	4	Y
UNC10225231A	GSK2297428	4	Y
UNC10225238A	GSK190937	4	Y
UNC10225260A	GSK1024306	4	Y
UNC10225355A	GSK2269905	4	Y
UNC10225360A	GI262866	4	Y
UNC10225391A	GSK1379748	4	Y
UNC10225435A	GSK2347225	4	Y
UNC10225450A	GSK205189	4	Y
TPKI-106		4	Y
TPKI-85		4	Y
TPKI-97		4	Y
GW869979		4	Y
TPKI-100		3	Y
TPKI-101		3	Y
TPKI-102		3	Y
TPKI-103		3	Y
TPKI-104		3	Y
TPKI-60		3	Y
SB-210486		3	Y
TPKI-26		3	Y
TPKI-28		3	Y
UNC10112764A	GW824645	3	Y
UNC10225018A	GSK2298859	3	Y
UNC10225126A	GW869640	3	Y
UNC10225127A	GSK1024304	3	Y
UNC10225131A	GSK2296823	3	Y
UNC10225178A	GSK2342769	3	Y
UNC10225268A	GW659008	3	Y
UNC10225319A	GW689066	3	Y
UNC10225343A	GSK2297099	3	Y

UNC10225374A	GSK2224810	3	Y
UNC10225388A	GSK1398472	3	Y
UNC10225398A	GSK1398477	3	Y
UNC10225445A	GSK1398475	3	Y
UNC10225449A	GI230329	3	Y
UNC10225488A	GW275568	3	Y
UNC10225491A	GW813349	3	Y
UNC10243861A	GSK2289044	3	Y
UNC10243881A	GSK2363608	3	Y
TPKI-16		2	Y
UNC10224997A	GW872411	2	Y
UNC10225005A	GSK2344444	2	Y
UNC10225080A	GSK1379757	2	Y
UNC10225109A	GW577382	2	Y
UNC10225125A	GW684088	2	Y
UNC10225140A	SB-743341	2	Y
UNC10225143A	GSK2283293	2	Y
UNC10225161A	GSK1379712	2	Y
UNC10225202A	GSK2297542	2	Y
UNC10225229A	GW868318	2	Y
UNC10225239A	GW867587	2	Y
UNC10225287A	GSK346294	2	Y
UNC10225332A	GSK2297430	2	Y
UNC10225339A	GSK429286	2	Y
UNC10225403A	GSK2373690	2	Y
UNC10225415A	GSK2334006	2	Y
UNC10225422A	GW424170	2	Y
UNC10225439A	GSK2333389	2	Y
UNC10225468A	SB-517081	2	Y
UNC10225477A	GW867588	2	Y
PFE-PKIS 1		2	Y
PFE-PKIS 12		2	Y
PFE-PKIS 21		2	Y
PFE-PKIS 43		2	Y
TPKI-81		2	Y
TPKI-91		2	Y
UNC10225468A		2	Y
GSK2358994		2	Y
TPKI-71		1	Y
UNC10225002A		1	Y
TPKI-25		1	Y
UNC10225030A	GSK2250882	1	Y

UNC10225056A	GSK1229782	1	Y
UNC10225083A	GSK2258759	1	Y
UNC10225170A	GW284543	1	Y
UNC10225379A	GW867253	1	Y
UNC10243860A	GSK2336394	1	Y
UNC10112830A		0	Y
UNC10225088A		0	Y
