# Supplementary material for: Progress towards a public chemogenomic set for protein kinases and a call for contributions
Source: PLoS One. 2017 Aug 2;12(8):e0181585. doi: 10.1371/journal.pone.0181585 (PMC5540273; doi:10.1371/journal.pone.0181585)
Supplement: S6 Table — (PDF) [file pone.0181585.s006.pdf]

name of compound	pub med ID
compound 13	25415869
5 iodo tubericidin	23071153
AC220, quizartinib	19654408
AM-0216	24980832
AM-0561	24980832
AMG-706 motesanib	16951187
AMG-900	20935223
Amgen compound 44	25587754
Amgen compound 52	25587754
AnnH75	26192590
AR-470	20854944
ARA014418	12928438
AZ compound 7b	24900749
AZ compound 7h	26985319
AZD-1208	22727640
AZD1152	17495131
AZD1775, MK1775	19887545
AZD2014	23375793
B19 Gray lab	21802008
Bafetinib, NS-187, INNO-406	16105974 & 21154127
BAY-61-3606	22815993
BAY320	26885717
BGJ-398 (Infigratinib)	21936542
BI-2536	17291758
BI6727, volasertib	19383823
BIBW-2992 (Afatinib)	18408761
BIX-02188	18834865
BIX-02565	22056746
BLU9931	25776529
BMS-540215, brivanib	16570908
BMX-IN-1	23594111
canertinib, CI-1033	10753475
CCT244747	22929806
CCT245737	26295308 and JMC 2016 epub ahead of print
CCT251545	26502155
CFI-400945	25043604
CGI1746	21113169
CHIR-99021	12606497
CHMFL-KIT-110	27077705
CI-1040, PD184352	10395327
Compound 10.HCl	23147077

compound 47 LJI308; pan-RSK	26270416 & 24554780
compound 48	26355916
CRT0105446	26540348
CRUK naphthyridine compound 51	27326329
CX-4945, silmasertib	21159648 & 21174434
D4476	14710188
DDR1-IN-1	23899692
EHT5372	25264830; 25556849; AACR poster as well
EMD 1214063, Tepotinib	23553846
ERK5-IN-1	24239623
erlotinib, OSI-744	18183025
EW-7197	24817629
FRAX1036	25596744
FRAX597	23960073
G-5555	26713112
G1T28	26826116
GDC-0879	19276360 & 22037378
gefitinib, ZD1839	11585753
Genentech compound 13	26985305
Genentech compound 32	26985305
GNE7915	22985112
GNF-5	20072125
GS9973, entospletinib	24779514
GSK114	27246618
GSK2334470	21341675
GSK2656157	24900593
GSK269962A	17018693
GSK429286	17201405
GSK461364A	19690138
GW2580	16249345
HG-10-102-01	23066449
HS38	24070067
ibrutinib, PCI-32765	20615965
IKK-2 inhibitor VIII	15225718
imatinib	10910906
JH-II-127	26005538
JH-IX-179	27190596
JNK-IN-11	22284361
JNK-IN-12	22284361
JNK-IN-7	22284361
JNK-IN-8	22284361
Knapp et al compound 12g	25822739

KU-60019	19808981
lapatinib, GW2016	12467226
LIMKi, compound 3 and 4 in Mol Canc Ther paper	19001433
LIMKi, compound 3 in Mol Canc Ther paper	26540348
LY-317615, enzastaurin	16103100
LY2857785	24688048
LY3009120	25965804 & 26343583
masitinib AB1010	19789626
MELK-T1, compound 7 Astex Janssen	25589925
Merck amidopyrazole compound 32	26101573
Merck compound 14	26101574
MK-5108, VX-689	20053775
MK2206	20571069
ML281	23256033
ML315	23642479
Mli-2	26407721
MLN-120B	16439676
MLN-518, Tandutinib	12124172
MLN-8054	17360485
Mps-BAY2a	23933817
MSC 2032964A	21064192
ND-2110	26621451
ND-2158	26621451
Novartis compound 11	25633741
NVS-MELK8a	27187609
NVS-PAK1-1	26191365
OSI compound 13f	23856049
OSI-906, linsitinib	21425998
P505-15 (PRT062607)	22040680
palbociclib	15542782
PD 0325901	18952427
PF-4708671	20704563
PF-477736	18723486
PF3644022	20237073
PIM447	26505898
PLX-4720; Vemurafenib	18287029
PTK-787, vatalanib	15742376
RAF-265, CHIR-265	20124452
ribociclib	26390342
Roche compound 17	23664880
Roche compound 24f	23352510
SB-203580	10702313

SB-590885	17145850
SCH772984	23614898 & 25195011
Scripps compound 35	20684608
selumetinib (AZD6244, ARRY886)	17332304 & 17699718
SGK1 Sanofi 14n	25589934
SGK1 Sanofi ex.290-R	patent - WO201414006
SGX-523	19934279
Shionogi 19c	25801152
Shionogi 27f	25625617
skepinone-L	22198732
SR-3029	23787102
St Jude compound 27g	26632965
Sunesis Biogen Idec	21118801
TAK960	22188812
THZ1	25043025
THZ531	27571479
Tofacitinib, tasocitinib, CP-690550	14593182
Trametinib (GSK1120212)	23237773 & 24900312
TX-85-1	25326665
VE-821	21490603
VER-246608	25404640
Vertex 11e	19827834
VX-745	18183025 & 24900264
WNK463	27595330
WZ4003, HTH-01-015	24171924
XL-413	22560567
XMD8-92	20832753
