# Supplementary material for: Progress towards a public chemogenomic set for protein kinases and a call for contributions
Source: PLoS One. 2017 Aug 2;12(8):e0181585. doi: 10.1371/journal.pone.0181585 (PMC5540273; doi:10.1371/journal.pone.0181585)
Supplement: S7 Table — (PDF) [file pone.0181585.s007.pdf]

approved symbol	status	assay?	covered	covered by PKIS	covered by PKIS2	covered by literature
AXL	kinase	yes	y	no	no	yes
BMP2K	kinase	yes	y	no	no	yes
ICK	kinase	yes	y	no	no	yes
PAK3	kinase	yes	y	no	no	yes
SIK2	kinase	yes	y	no	no	yes
SLK	kinase	yes	y	no	no	yes
STK35	kinase	yes	y	no	no	yes
WNK2	kinase	yes	y	no	no	yes
RPS6KA2	kinase	yes	y	no	no	yes
AAK1	kinase	yes	y	no	no	yes
ABL2	kinase	yes	y	no	no	yes
ACVR1	kinase	yes	y	no	no	yes
ARAF	kinase	yes	y	no	no	yes
ATM	kinase	yes	y	no	no	yes
ATR	kinase	yes	y	no	no	yes
BMX	kinase	yes	y	no	no	yes
BTK	kinase	yes	y	no	no	yes
CAMK1	kinase	yes	y	no	no	yes
CAMK2G	kinase	yes	y	no	no	yes
CAMKK1	kinase	yes	y	no	no	yes
CAMKK2	kinase	yes	y	no	no	yes
CDC42BPG	kinase	yes	y	no	no	yes
CDC7	kinase	yes	y	no	no	yes
CDK11B	kinase	yes	y	no	no	yes
CDK12	kinase	yes	y	no	no	yes
CDK13	kinase	yes	y	no	no	yes
CDK19	kinase	yes	y	no	no	yes
CDK7	kinase	yes	y	no	no	yes
CDK8	kinase	yes	y	no	no	yes
CDK9	kinase	yes	y	no	no	yes
CDKL5	kinase	yes	y	no	no	yes
CHEK1	kinase	yes	y	no	no	yes
CHEK2	kinase	yes	y	no	no	yes
CLK4	kinase	yes	y	no	no	yes
DAPK1	kinase	yes	y	no	no	yes
DAPK2	kinase	yes	y	no	no	yes
DCLK2	kinase	yes	y	no	no	yes
DYRK1B	kinase	yes	y	no	no	yes
DYRK3	kinase	yes	y	no	no	yes
EIF2AK3	kinase	yes	y	no	no	yes

EIF2AK4	pseudokinase	yes	y	no	no	yes
EPHB1	kinase	yes	y	no	no	yes
EPHB4	kinase	yes	y	no	no	yes
FES	kinase	yes	y	no	no	yes
FGFR2	kinase	yes	y	no	no	yes
FGFR3	kinase	yes	y	no	no	yes
FGFR4	kinase	yes	y	no	no	yes
FGR	kinase	yes	y	no	no	yes
FYN	kinase	yes	y	no	no	yes
HIPK2	kinase	yes	y	no	no	yes
HIPK3	kinase	yes	y	no	no	yes
HIPK4	kinase	yes	y	no	no	yes
IRAK1	kinase	yes	y	no	no	yes
IRAK3	kinase	yes	y	no	no	yes
IRAK4	kinase	yes	y	no	no	yes
LIMK1	kinase	yes	y	no	no	yes
LIMK2	kinase	yes	y	no	no	yes
LYN	kinase	yes	y	no	no	yes
MAP2K2	kinase	yes	y	no	no	yes
MAP2K4	kinase	yes	n	no	no	yes
MAP3K1	kinase	yes	y	no	no	yes
MAP3K14	kinase	yes	y	no	no	yes
MAP3K20	kinase	yes	y	no	no	yes
MAP3K7	kinase	yes	y	no	no	yes
MAP3K9	kinase	yes	y	no	no	yes
MAP4K3	kinase	yes	y	no	no	yes
MAPK1	kinase	yes	y	no	no	yes
MAPK12	kinase	yes	y	no	no	yes
MAPK13	kinase	yes	y	no	no	yes
MAPK6	kinase	yes	y	no	no	yes
MAPK7	kinase	yes	y	no	no	yes
MAPKAPK2	kinase	yes	y	no	no	yes
MAPKAPK3	kinase	yes	y	no	no	yes
MAPKAPK5	kinase	yes	y	no	no	yes
MARK2	kinase	yes	y	no	no	yes
MERTK	kinase	yes	y	no	no	yes
MYLK4	kinase	yes	y	no	no	yes
NEK10	kinase	yes	y	no	no	yes
NEK2	kinase	yes	y	no	no	yes
NEK6	kinase	yes	y	no	no	yes
NTRK2	kinase	yes	y	no	no	yes
NUAK2	kinase	yes	y	no	no	yes

**PAK1**	**kinase**	**yes**	**y**	**no**	**no**	**yes**
**PAK2**	**kinase**	**yes**	**y**	**no**	**no**	**yes**
**PAK4**	**kinase**	**yes**	**y**	**no**	**no**	**yes**
**PAK5**	**kinase**	**yes**	**y**	**no**	**no**	**yes**
**PDK1**	**kinase**	**yes**	**y**	**no**	**no**	**yes**
**PDK2**	**kinase**	**yes**	**y**	**no**	**no**	**yes**
**PDK3**	**kinase**	**yes**	**y**	**no**	**no**	**yes**
**PDK4**	**kinase**	**yes**	**y**	**no**	**no**	**yes**
**PHKG1**	**kinase**	**yes**	**y**	**no**	**no**	**yes**
**PIM2**	**kinase**	**yes**	**y**	**no**	**no**	**yes**
**PIM3**	**kinase**	**yes**	**y**	**no**	**no**	**yes**
**PKN2**	**kinase**	**yes**	**y**	**no**	**no**	**yes**
**PLK4**	**kinase**	**yes**	**y**	**no**	**no**	**yes**
**PRKAA2**	**kinase**	**yes**	**y**	**no**	**no**	**yes**
**PRKCA**	**kinase**	**yes**	**y**	**no**	**no**	**yes**
**PRKCB**	**kinase**	**yes**	**y**	**no**	**no**	**yes**
**PRKCD**	**kinase**	**yes**	**y**	**no**	**no**	**yes**
**PRKCE**	**kinase**	**yes**	**y**	**no**	**no**	**yes**
**PRKCG**	**kinase**	**yes**	**y**	**no**	**no**	**yes**
**PRKCQ**	**kinase**	**yes**	**y**	**no**	**no**	**yes**
**PTK2**	**kinase**	**yes**	**y**	**no**	**no**	**yes**
**PTK6**	**kinase**	**yes**	**y**	**no**	**no**	**yes**
**RAF1**	**kinase**	**yes**	**y**	**no**	**no**	**yes**
**RPS6KA1**	**kinase**	**yes**	**y**	**no**	**no**	**yes**
**RPS6KA3**	**kinase**	**yes**	**y**	**no**	**no**	**yes**
**RPS6KA4**	**kinase**	**yes**	**y**	**no**	**no**	**yes**
**RPS6KA5**	**kinase**	**yes**	**y**	**no**	**no**	**yes**
**RPS6KA6**	**kinase**	**yes**	**y**	**no**	**no**	**yes**
**RPS6KB2**	**kinase**	**yes**	**y**	**no**	**no**	**yes**
**SBK1**	**kinase**	**yes**	**y**	**no**	**no**	**yes**
**SGK1**	**kinase**	**yes**	**y**	**no**	**no**	**yes**
**SRC**	**kinase**	**yes**	**y**	**no**	**no**	**yes**
**STK16**	**kinase**	**yes**	**y**	**no**	**no**	**yes**
**STK24**	**kinase**	**yes**	**y**	**no**	**no**	**yes**
**STK25**	**kinase**	**yes**	**y**	**no**	**no**	**yes**
**STK26**	**kinase**	**yes**	**y**	**no**	**no**	**yes**
**STK33**	**kinase**	**yes**	**y**	**no**	**no**	**yes**
**SYK**	**kinase**	**yes**	**y**	**no**	**no**	**yes**
**TBK1**	**kinase**	**yes**	**y**	**no**	**no**	**yes**
**TEK**	**kinase**	**yes**	**y**	**no**	**no**	**yes**
**TNIK**	**kinase**	**yes**	**y**	**no**	**no**	**yes**
**TNK2**	**kinase**	**yes**	**y**	**no**	**no**	**yes**

**TNNI3K**	**kinase**	**yes**	**y**	**no**	**no**	**yes**
**ULK1**	**kinase**	**yes**	**y**	**no**	**no**	**yes**
**ULK2**	**kinase**	**yes**	**y**	**no**	**no**	**yes**
**WEE1**	**kinase**	**yes**	**y**	**no**	**no**	**yes**
**WNK1**	**kinase**	**yes**	**y**	**no**	**no**	**yes**
**WNK3**	**kinase**	**yes**	**y**	**no**	**no**	**yes**
**WNK4**	**kinase**	**yes**	**y**	**no**	**no**	**yes**
**ZAP70**	**kinase**	**yes**	**y**	**no**	**no**	**yes**
