# Supplementary material for: Progress towards a public chemogenomic set for protein kinases and a call for contributions
Source: PLoS One. 2017 Aug 2;12(8):e0181585. doi: 10.1371/journal.pone.0181585 (PMC5540273; doi:10.1371/journal.pone.0181585)
Supplement: S8 Table — (PDF) [file pone.0181585.s008.pdf]

approved symbol	status	assay?	covered	HGNC ID	synonyms
**AATK**	**kinase**	**no**	**n**	HGNC:21	AATYK, KIAA0641, LMTK1, LMR1, AATYK1, PPP1R77
**ACVR1C**	**kinase**	**yes**	**n**	HGNC:18123	ALK7, ACVRLK7
**ACVR2A**	**kinase**	**yes**	**n**	HGNC:173	ACTRII, ACTR2
**ACVR2B**	**kinase**	**yes**	**n**	HGNC:174	ActR-IIB, ACTR2B
**ADCK1**	**kinase**	**no**	**n**	HGNC:19038	FLJ39600
**ADCK2**	**kinase**	**no**	**n**	HGNC:19039	MGC20727
**ADCK5**	**kinase**	**no**	**n**	HGNC:21738	FLJ35454
**ALK**	**kinase**	**yes**	**n**	HGNC:427	CD246
**ALPK1**	**kinase**	**no**	**n**	HGNC:20917	Lak, FLJ22670, KIAA1527, AlphaK3
**ALPK2**	**kinase**	**no**	**n**	HGNC:20565	HAK, AlphaK2
**ALPK3**	**kinase**	**no**	**n**	HGNC:17574	MAK, KIAA1330, Midori, AlphaK1
**AMHR2**	**kinase**	**no**	**n**	HGNC:465	MISR2, MISRII
**ANKK1**	**kinase**	**yes**	**n**	HGNC:21027	X-kinase, SgK288
**BCKDK**	**kinase**	**no**	**n**	HGNC:16902	BDK; BCKDKD
**BCR**	**kinase**	**no**	**n**	HGNC:1014	ALL; CML; PHL; BCR1; D22S11; D22S662
**BMPR2**	**kinase**	**yes**	**n**	HGNC:1078	BRK-3, T-ALK, BMPR3, BMPR-II
**BUB1B**	**kinase**	**yes**	**n**	HGNC:1149	BUBR1, MAD3L, Bub1A, SSK1
**CAMK1D**	**kinase**	**yes**	**n**	HGNC:19341	CKLiK; CaM-K1; CaMKID, CaMK1d
**CAMK1G**	**kinase**	**yes**	**n**	HGNC:14585	VWS1, CLICKIII, dJ272L16.1, CaMK1g
**CAMK2A**	**kinase**	**yes**	**n**	HGNC:1460	KIAA0968, CaMKIINalpha, CAMKA, CaMK2a
**CAMK2B**	**kinase**	**yes**	**n**	HGNC:1461	CAM2, CAMK2, CAMKB, CaMK2b
**CAMK2D**	**kinase**	**yes**	**n**	HGNC:1462	CAMKD, CaMK2d
**CAMK4**	**kinase**	**yes**	**n**	HGNC:1464	CaMK-GR,caMK; CaMK IV; CaMK4
**CAMKV**	**pseudokinase**	**no**	**n**	HGNC:28788	MGC8407, VACAMKL
**CDK1**	**kinase**	**yes**	**n**	HGNC:1722	CDC28A, CDC2, P34CDC2
**CDK10**	**kinase**	**yes**	**n**	HGNC:1770	PISSLRE
**CDK11A**	**kinase**	**yes**	**n**	HGNC:1730	CDC2L2; CDC2L3; p58GTA; PITSLRE; CDK11-p46; CDK11-p58; CDK11-p110
**CDK11B**	**kinase**	**yes**	**n**		p58; PK58; CDK11; CLK-1; CDC2L1; PITSLREA; p58CLK-1; CDK11-p46; CDK11-p58; p58CDC2L1; CDK11-p110
**CDK14**	**kinase**	**yes**	**n**	HGNC:8883	PFTAIRE1, PFTK1
**CDK15**	**kinase**	**yes**	**n**	HGNC:14434	PFTAIRE2, ALS2CR7, PFTK2
**CDK16**	**kinase**	**yes**	**n**	HGNC:8749	PCTAIRE, PCTAIRE1, PCTGAIRE, FLJ16665, PCTK1
**CDK17**	**kinase**	**yes**	**n**	HGNC:8750	PCTAIRE2, PCTK2
**CDK18**	**kinase**	**yes**	**n**	HGNC:8751	PCTAIRE3, PCTK3
**CDK20**	**kinase**	**yes**	**n**	HGNC:21420	p42, CCRK; CDCH; PNQALRE
**CDK5**	**kinase**	**yes**	**n**	HGNC:1774	PSSALRE
**CDKL1**	**kinase**	**yes**	**n**	HGNC:1781	KKIALRE, P42
**CDKL3**	**kinase**	**yes**	**n**	HGNC:15483	NKIAMRE
**CDKL4**	**kinase**	**no**	**n**	HGNC:19287	
**CHUK**	**kinase**	**yes**	**n**	HGNC:1974	IKK1, IKK-alpha, IkBKA, NFKBIKA, IKKA, IKBKA, TCF16, IKKa
**COQ8A**	**kinase**	**yes**	**n**	HGNC:16812	COQ8, SCAR9, ADCK3; ARCA2; CABC1; COQ10D4
**COQ8B**	**kinase**	**yes**	**n**	HGNC:19041	FLJ12229, COQ8, ADCK4, NPHS9
**CSK**	**kinase**	**yes**	**n**	HGNC:2444	
**CSNK1A1L**	**kinase**	**yes**	**n**	HGNC:20289	MGC33182, CK1a2, CK1 alpha 1L
**CSNK1G1**	**kinase**	**yes**	**n**	HGNC:2454	CK1gamma1, CK1g1
**CSNK1G2**	**kinase**	**yes**	**n**	HGNC:2455	CK1g2
**CSNK1G3**	**kinase**	**yes**	**n**	HGNC:2456	CKI-gamma 3L, CSNK1G3, CK1g3
**DCLK1**	**kinase**	**yes**	**n**	HGNC:2700	KIAA0369, DCLK, DCDC3A, CL1, CLICK1, DCAMKL1
**DMPK**	**kinase**	**yes**	**n**	HGNC:2933	DMK, DM1PK, MDPK, MT-PK, DM, DM1, DMPK1
**DSTYK**	**kinase**	**yes**	**n**	HGNC:29043	KIAA0472, DustyPK, CAKUT1, HDCMD38P, RIP5, RIPK5, SgK496
**DYRK4**	**kinase**	**yes**	**n**	HGNC:3095	
**EEF2K**	**kinase**	**yes**	**n**	HGNC:24615	eEF-2K, HSU93850
**EIF2AK1**	**kinase**	**yes**	**n**	HGNC:24921	HRI, KIAA1369, HCR
**EIF2AK2**	**kinase**	**yes**	**n**	HGNC:9437	PKR, EIF2AK1, PPP1R83, PRKR

**EPHA1**	**kinase**	**yes**	**n**	HGNC:3385	EPH, EPHT, EPHT1
**EPHA10**	**pseudokinase**	**no**	**n**	HGNC:19987	FLJ16103, FLJ33655, EphA10
**EPHA3**	**kinase**	**yes**	**n**	HGNC:3387	EK4, ETK, ETK1, HEK, HEK4, TYRO4
**EPHA4**	**kinase**	**yes**	**n**	HGNC:3388	SEK; HEK8; TYRO1
**EPHA5**	**kinase**	**yes**	**n**	HGNC:3389	Hek7, TYRO4, CEK7, EHK1, Els1, Rek7, bsk
**EPHA6**	**kinase**	**yes**	**n**	HGNC:19296	FLJ35246, EHK-2, EHK2, EK12, EPA6, HEK12, PRO57066
**EPHA7**	**kinase**	**yes**	**n**	HGNC:3390	Hek11, EHK-3, EHK3, EK11, HEK11
**EPHA8**	**kinase**	**yes**	**n**	HGNC:3391	Hek3, EEK, EK3, HEK3
**EPHB2**	**kinase**	**yes**	**n**	HGNC:3393	Hek5, Tyro5, CAPB, DRT, EK5, EPHT3, ERK, PCBC
**EPHB3**	**kinase**	**yes**	**n**	HGNC:3394	EK2, ETK2, HEK2, TYRO6
**ERN1**	**kinase**	**yes**	**n**	HGNC:3449	IRE1; IRE1P; IRE1a; hIRE1p
**ERN2**	**kinase**	**yes**	**n**	HGNC:16942	IRE1-BETA, IRE1b, IRE2p, hIRE2p, IRE2
**FASTK**	**kinase**	**yes**	**n**	HGNC:24676	FAST
**FER**	**kinase**	**yes**	**n**	HGNC:3655	PPP1R74, TYK3, p94-Fer
**FRK**	**kinase**	**yes**	**n**	HGNC:3955	RAK, GTK, PTK5
**GRK1**	**kinase**	**yes**	**n**	HGNC:10013	GPRK1, RHOK, RK
**GRK2**	**kinase**	**yes**	**n**	HGNC:289	ADRBK1, BARK1, BETA-ARK1
**GRK3**	**kinase**	**yes**	**n**	HGNC:290	ADRBK2, BARK2
**GRK4**	**kinase**	**yes**	**n**	HGNC:4543	GPRK2L, GPRK4a, IT11, GRK4, GPRK4
**GRK5**	**kinase**	**yes**	**n**	HGNC:4544	GPRK5
**GRK6**	**kinase**	**yes**	**n**	HGNC:4545	GPRK6
**GRK7**	**kinase**	**yes**	**n**	HGNC:17031	GPRK7
**GUCY2C**	**pseudokinase**	**no**	**n**	HGNC:4688	DIAR6, GUC2C, MECIL, MUCIL, STAR, HSER
**GUCY2D**	**pseudokinase**	**no**	**n**	HGNC:4689	CORD5, CORD6, CYGD, GUC1A4, GUC2D, LCA, LCA1, RCD2, RETGC-1, ROS-GC1, ROSGC, retGC
**GUCY2F**	**pseudokinase**	**no**	**n**	HGNC:4691	CYGF, GC-F, GUC2DL, GUC2F, RETGC-2, ROS-GC2
**HCK**	**kinase**	**yes**	**n**	HGNC:4840	JTK9, p59Hck, p61Hck
**HUNK**	**kinase**	**yes**	**n**	HGNC:13326	
**ILK**	**pseudokinase**	**no**	**n**	HGNC:6040	HEL-S-28-1, ILK-2, P59, p59ILK, ILK
**IRAK2**	**pseudokinase**	**yes**	**n**	HGNC:6113	IRAK-2
**ITK**	**kinase**	**yes**	**n**	HGNC:6171	EMT, PSCTK2, LYK, LPFS1
**KALRN**	**kinase**	**no**	**n**	HGNC:4814	duo, Hs.8004, TRAD, DUET, Kalirin, ARHGEF24, trad
**KSR1**	**pseudokinase**	**yes**	**n**	HGNC:6465	RSU2, KSR
**KSR2**	**pseudokinase**	**yes**	**n**	HGNC:18610	FLJ25965
**LATS1**	**kinase**	**yes**	**n**	HGNC:6514	WARTS, wts
**LATS2**	**kinase**	**yes**	**n**	HGNC:6515	KPM
**LMTK2**	**kinase**	**no**	**n**	HGNC:17880	KIAA1079, KPI2, KPI-2, cprk, LMR2, BREK, AATYK2, PPP1R100
**LMTK3**	**kinase**	**no**	**n**	HGNC:19295	KIAA1883, LMR3, TYKLM3, PPP1R101
**LRRK1**	**kinase**	**no**	**n**	HGNC:18608	FLJ23119, KIAA1790, Roco1, RIPK6
**MAK**	**kinase**	**yes**	**n**	HGNC:6816	dJ417M14.2, RP62
**MAP2K3**	**kinase**	**yes**	**n**	HGNC:6843	MAPKK3, MEK3, MKK3, PRKMK3, SAPKK-2, SAPKK2
**MAP2K4**	**kinase**	**yes**	**n**	HGNC:6844	JNKK, JNKK1, MAPKK4, MEK4, MKK4, PRKMK4, SAPKK-1, SAPKK1, SEK1, SERK1, SKK1
**MAP2K6**	**kinase**	**yes**	**n**	HGNC:6846	MEK6, MKK6, SAPKK3, MAPKK6
**MAP2K7**	**kinase**	**yes**	**n**	HGNC:6847	JNKK2, MAPKK7, MEK, MEK 7, MKK7, PRKMK7, SAPKK-4, SAPKK4
**MAP3K10**	**kinase**	**yes**	**n**	HGNC:6849	MST, MEKK10, MLK2
**MAP3K11**	**kinase**	**yes**	**n**	HGNC:6850	SPRK, MEKK11, MLK-3, MLK3, PTK1
**MAP3K12**	**kinase**	**yes**	**n**	HGNC:6851	MUK, DLK, ZPKP1, MEKK12
**MAP3K13**	**kinase**	**yes**	**n**	HGNC:6852	LZK, MEKK13, MLK
**MAP3K15**	**kinase**	**yes**	**n**	HGNC:31689	bA723P2.3, FLJ16518, ASK3, MAP3K7
**MAP3K2**	**kinase**	**yes**	**n**	HGNC:6854	MEKK2B, MEKK2
**MAP3K21**	**kinase**	**yes**	**n**	HGNC:29798	MLK4; KIAA1804; dJ862P8.3
**MAP3K3**	**kinase**	**yes**	**n**	HGNC:6855	MAPKKK3, MEKK3
**MAP3K4**	**kinase**	**yes**	**n**	HGNC:6856	MTK1, MAPKKK4, KIAA0213, MEKK4
**MAP3K6**	**kinase**	**yes**	**n**	HGNC:6858	MAPKKK6, ASK2, MEKK6

**MAP3K8**	**kinase**	**yes**	**n**	HGNC:6860	AURA2, COT, EST, ESTF, MEKK8, TPL2, Tpl-2, c-COT
**MAP4K1**	**kinase**	**yes**	**n**	HGNC:6863	HPK1
**MAP4K5**	**kinase**	**yes**	**n**	HGNC:6867	KHS1, GCKR, KHS, MAPKKKK5
**MAPK4**	**kinase**	**yes**	**n**	HGNC:6878	Erk3-related, Erk4, ERK-4, ERK4, PRKM4, p63-MAPK, p63MAPK
**MARK1**	**kinase**	**yes**	**n**	HGNC:6896	MARK, PAR-1C, Par-1c, Par1c
**MARK3**	**kinase**	**yes**	**n**	HGNC:6897	CTAK1, KP78, PAR-1A, PAR1A, Par-1a
**MARK4**	**kinase**	**yes**	**n**	HGNC:13538	Nbla00650, FLJ90097, KIAA1860, PAR-1D, MARK4LS, MARKL1, MARKL1L
**MAST1**	**kinase**	**yes**	**n**	HGNC:19034	SAST, KIAA0973, SAST170
**MAST2**	**kinase**	**no**	**n**	HGNC:19035	MAST205, KIAA0807, MTSSK
**MAST3**	**kinase**	**no**	**n**	HGNC:19036	KIAA0561
**MAST4**	**kinase**	**no**	**n**	HGNC:19037	KIAA0303
**MASTL**	**kinase**	**no**	**n**	HGNC:19042	FLJ14813, THC2, Gwl, GREATWALL, GW, GWL, MAST-L
**MATK**	**kinase**	**yes**	**n**	HGNC:6906	HYLTK, CTK, HYL, Lsk, CHK, HHYLTK, DKFZp434N1212, MGC1708, MGC2101
**MKNK1**	**kinase**	**yes**	**n**	HGNC:7110	MNK1
**MKNK2**	**kinase**	**yes**	**n**	HGNC:7111	MNK2, GRPK7
**MLKL**	**pseudokinase**	**yes**	**n**	HGNC:26617	FLJ34389, hMLKL
**MOK**	**kinase**	**yes**	**n**	HGNC:9833	RAGE1, STK30, RAGE, RAGE-1
**MOS**	**kinase**	**yes**	**n**	HGNC:7199	MSV
**MST1R**	**kinase**	**yes**	**n**	HGNC:7381	CDw136, CD136, NPCA3, PTK8, RON
**MYLK3**	**kinase**	**yes**	**n**	HGNC:29826	MLCK; MLCK2; caMLCK
**MYO3A**	**kinase**	**yes**	**n**	HGNC:7601	DFNB30
**MYO3B**	**kinase**	**yes**	**n**	HGNC:15576	
**NEK1**	**kinase**	**yes**	**n**	HGNC:7744	NY-REN-55, KIAA1901, SRPS2, SRPS2A, SRTD6
**NEK11**	**kinase**	**yes**	**n**	HGNC:18593	FLJ23495
**NEK3**	**kinase**	**yes**	**n**	HGNC:7746	HSPK36, MGC29949
**NEK4**	**kinase**	**yes**	**n**	HGNC:11399	NRK2, pp12301, STK2
**NEK5**	**kinase**	**yes**	**n**	HGNC:7748	
**NEK7**	**kinase**	**yes**	**n**	HGNC:13386	
**NEK8**	**kinase**	**yes**	**n**	HGNC:13387	NPHP9, JCK, NEK12A, RHPD2
**NIM1K**	**kinase**	**yes**	**n**	HGNC:28646	MGC42105, NIM1
**NPR1**	**pseudokinase**	**no**	**n**	HGNC:7943	GUCY2A, ANPa, A1893888, GC-A, NPR-A, NPRA, Pndr
**NPR2**	**pseudokinase**	**no**	**n**	HGNC:7944	GUCY2B, ANPb, AMDM, ANPRB, ECDM, GUC2B, NPRB, NPRBi, SNSK
**NRBP1**	**pseudokinase**	**no**	**n**	HGNC:7993	BCON3, MUDPNP, MADM, NRBP
**NRBP2**	**pseudokinase**	**no**	**n**	HGNC:19339	DKFZp434P086, TRG16, pp9320
**NRK**	**kinase**	**no**	**n**	HGNC:25391	DKFZp686A17109, NESK, NRK/ZC4
**OBSCN**	**kinase**	**no**	**n**	HGNC:15719	KIAA1556, UNC89, KIAA1639, ARHGEF30
**OXSR1**	**kinase**	**yes**	**n**	HGNC:8508	OSR1
**PASK**	**kinase**	**yes**	**n**	HGNC:17270	PASKIN, KIAA0135, STK37
**PBK**	**kinase**	**yes**	**n**	HGNC:18282	TOPK, FLJ14385, Nori-3, SPK, CT84, HEL164
**PDIK1L**	**kinase**	**no**	**n**	HGNC:18981	CLIK1L, STK35L2
**PEAK1**	**pseudokinase**	**yes**	**n**	HGNC:29431	KIAA2002, sgk269, SGK269
**PHKG2**	**kinase**	**yes**	**n**	HGNC:8931	GSD9C
**PINK1**	**kinase**	**yes**	**n**	HGNC:14581	BRPK, PARK6
**PKDCC**	**kinase**	**no**	**n**	HGNC:25123	SgK493, "vertebrate lonesome kinase", Vlk, SGK493
**PKMYT1**	**kinase**	**yes**	**n**	HGNC:29650	MYT1, PPP1R126
**PKN1**	**kinase**	**yes**	**n**	HGNC:9405	DBK, PRK1, PKN, MGC46204, PAK1, PAK-1, PKN-ALPHA, PRKCL1
**PKN3**	**kinase**	**yes**	**n**	HGNC:17999	PKNbeta, UTDP4-1
**PNCK**	**kinase**	**yes**	**n**	HGNC:13415	MGC45419, CaMK1b, BSTK3
**POMK**	**pseudokinase**	**no**	**n**	HGNC:26267	FLJ23356, SgK196, MDDGA12, MDDGC12, SGK196
**PRAG1**	**pseudokinase**	**no**	**n**	HGNC:157285	NACK, PEAK2, PRAGMIN, SGK223, SgK223
**PRKAA1**	**kinase**	**yes**	**n**	HGNC:9376	AMPKa1, AMPK-alpha1, AI194361, AI450832, AL024255, AMPKalpha1, C130083N04Rik

**PRKACA**	**kinase**	**yes**	**n**	HGNC:9380	PKACa, PKACA, PPNAD4
**PRKACB**	**kinase**	**yes**	**n**	HGNC:9381	PKACb, PKA C-beta, PKACB
**PRKACG**	**kinase**	**yes**	**n**	HGNC:9382	PKACg, BDPLT19, KAPG
**PRKCI**	**kinase**	**yes**	**n**	HGNC:9404	PKCI, DXS1179E, nPKC-iota, PKCi
**PRKCZ**	**kinase**	**yes**	**n**	HGNC:9412	PKC2, PKC-ZETA, PKCz
**PRKDC**	**kinase**	**yes**	**n**	HGNC:9413	DNPK1, p350, DNAPK, XRCC7, DNA-PKcs,HYRC, HYRC1, IMD26
**PRKG1**	**kinase**	**yes**	**n**	HGNC:9414	PGK, PKG, AAT8, PKGB, PRKGR1B, cGK, cGK 1, cGK1, cGKI, cGKI-BETA, cGKI-alpha, PKG1
**PRKY**	**kinase**	**no**	**n**	HGNC:9444	PRKYP, PRKXP3, PRKY, PRKXP3P
**PRPF4B**	**kinase**	**yes**	**n**	HGNC:17346	PR4H; PRP4; PRP4H; PRP4K; dJ1013A10.1
**PSKH1**	**kinase**	**no**	**n**	HGNC:9529	
**PSKH2**	**pseudokinase**	**no**	**n**	HGNC:18997	
**PTK7**	**pseudokinase**	**no**	**n**	HGNC:9618	CCK4, CCK-4
**PXK**	**pseudokinase**	**no**	**n**	HGNC:23326	FLJ20335, MONaKA, Slob
**RIOK1**	**kinase**	**yes**	**n**	HGNC:18656	AD034, FLJ30006, bA288G3.1, RRP10
**RIOK3**	**kinase**	**yes**	**n**	HGNC:11451	SUDD
**RIPK1**	**kinase**	**yes**	**n**	HGNC:10019	RIP, RIP-1, RIP1
**RIPK3**	**kinase**	**yes**	**n**	HGNC:10021	RIP3, Rip3
**RIPK4**	**kinase**	**yes**	**n**	HGNC:496	DIK, ANKK2, RIP4, PKK, ANKRD3, NKRD3, PPS2
**RNASEL**	**kinase**	**no**	**n**	HGNC:10050	PRCA1, RNS4
**ROR1**	**kinase**	**yes**	**n**	HGNC:10256	NTRKR1, dJ537F10.1
**ROR2**	**kinase**	**yes**	**n**	HGNC:10257	BDB, BDB1, NTRKR2
**ROS1**	**kinase**	**yes**	**n**	HGNC:10261	MCF3, ROS, c-ros-1
**RPS6KC1**	**pseudokinase**	**no**	**n**	HGNC:10439	humS6PKh1, RPK118, RSKL1, S6K-delta-1, S6PKh1
**RPS6KL1**	**pseudokinase**	**no**	**n**	HGNC:20222	MGC11287, RSKL2
**RYK**	**kinase**	**no**	**n**	HGNC:10481	D3S3195, RYK1, JTK5, JTK5A1, RYK
**SBK2**	**kinase**	**no**	**n**	HGNC:34416	SGK069
**SBK3**	**kinase**	**yes**	**n**	HGNC:44121	SGK110
**SCYL1**	**pseudokinase**	**no**	**n**	HGNC:14372	HT019, P105, GKLP, NKTL, TAPK, TRAP, TEIF, MGC78454, NTKL, SCAR21
**SCYL2**	**pseudokinase**	**no**	**n**	HGNC:19286	KIAA1360, CVAK104
**SCYL3**	**pseudokinase**	**no**	**n**	HGNC:19285	PACE-1, PACE1
**SGK2**	**kinase**	**yes**	**n**	HGNC:13900	H-SGK2, dJ138B7.2
**SGK3**	**kinase**	**yes**	**n**	HGNC:10812	CISK, SGK2, SGKL
**SGK494**	**kinase**	**no**	**n**	HPRD:08680	gene bank Acc. No. NM_144610
**SIK1**	**kinase**	**yes**	**n**	HGNC:11142	MSK; SIK; SIK-1; SNF1LK
**SIK3**	**kinase**	**yes**	**n**	HGNC:29165	FLJ12240, L19, KIAA0999, QSK, SIK, SIK-3
**SMG1**	**kinase**	**no**	**n**	MIM:607032	ATX; LIP; 61E3.4
**SNRK**	**kinase**	**yes**	**n**	HGNC:30598	FLJ20224, HSNFRK, KIAA0096
**SPEG**	**kinase**	**no**	**n**	HGNC:16901	BPEG; CNM5; APEG1; APEG-1; SPEGbeta; SPEGalpha
**SRMS**	**kinase**	**yes**	**n**	HGNC:11298	SRM, dJ697K14.1, PTK70, C20orf148
**SRPK1**	**kinase**	**yes**	**n**	HGNC:11305	SFRSK1
**SRPK2**	**kinase**	**yes**	**n**	HGNC:11306	SFRSK2
**SRPK3**	**kinase**	**yes**	**n**	HGNC:11402	MSSK1, MSSK-1, STK23
**STK11**	**kinase**	**yes**	**n**	HGNC:11389	PJS, LKB1, hLKB1
**STK19**	**kinase**	**yes**	**n**	HGNC:11398	G11; RP1; D6S60; D6S60E; HLA-RP1
**STK3**	**kinase**	**yes**	**n**	HGNC:11406	MST2, KRS1
**STK31**	**pseudokinase**	**no**	**n**	HGNC:11407	TDRD8, SgK396, SGK396
**STK32A**	**kinase**	**yes**	**n**	HGNC:28317	MGC22688, YANK1
**STK32B**	**kinase**	**yes**	**n**	HGNC:14217	STKG6, YANK2, STK32, HSA250839
**STK32C**	**kinase**	**yes**	**n**	HGNC:21332	PKE, MGC23665, YANK3
**STK38**	**kinase**	**yes**	**n**	HGNC:17847	NDR, NDR1
**STK38L**	**kinase**	**yes**	**n**	HGNC:17848	KIAA0965, NDR2
**STK39**	**kinase**	**yes**	**n**	HGNC:17717	DCHT, SPAK, PASK, STLK3
**STK4**	**kinase**	**yes**	**n**	HGNC:11408	MST1, KRS2, YSK3
**STK40**	**pseudokinase**	**no**	**n**	HGNC:21373	MGC4796, SgK495, SHIK

**STKLD1**	**pseudokinase**	**no**	**n**	HGNC:28669	MGC43306, SGK071, C9orf96, SgK071, Sk521	
**STRADA**	**pseudokinase**	**no**	**n**	HGNC:30172	LYK5; PMSE; Stlk; STRAD; NY-BR-96; STRAD alpha, STLK5	
**STRADB**	**pseudokinase**	**no**	**n**	HGNC:13205	STRAD beta; CALS-21, PAPK, ILPIPA, ILPIP, ALS2CR2, PRO1038, STLK6	
**STYK1**	**pseudokinase**	**no**	**n**	HGNC:18889	SuRTK106, DKFZp761P1010, NOK	
**TAF1**	**kinase**	**no**	**n**	HGNC:11535	OF; XDP; BA2R; CCG1; CCGS; DYT3; KAT4; P250; NSCL2; TAF2A; MRXS33; N-TAF1; TAFII250; DYT3/TAF1; TAFII-250; TAF(II)250	
**TAOK1**	**kinase**	**yes**	**n**	HGNC:29259	KIAA1361, MARKK, PSK2, MAP3K16, FLJ14314, TAO1, KFC-B, PSK-2, hKFC-B, hTAOK1	
**TAOK2**	**kinase**	**yes**	**n**	HGNC:16835	KIAA0881, PSK, PSK1, TAO2, MAP3K17, PSK1-BETA	
**TAOK3**	**kinase**	**yes**	**n**	HGNC:18133	JIK, DPK, MAP3K18, hKFC-A, TAO3	
**TBCK**	**pseudokinase**	**no**	**n**	HGNC:28261	HSPC302, MGC16169, IHPRF3L	
**TEC**	**kinase**	**yes**	**n**	HGNC:11719	PSCTK4	
**TESK1**	**kinase**	**yes**	**n**	HGNC:11731		
**TESK2**	**kinase**	**yes**	**n**	HGNC:11732		
**TEX14**	**pseudokinase**	**no**	**n**	HGNC:11737	CT113, SgK307	
**TGFBR2**	**kinase**	**yes**	**n**	HGNC:11773		0
**TIE1**	**kinase**	**yes**	**n**	HGNC:11809	JTK14, AAT3, FAA3, LDS1B, LDS2, LDS2B, MFS2, RIIC, TAAD2, TGFR-2, TGFbeta-RII, TIE	
**TLK1**	**kinase**	**yes**	**n**	HGNC:11841	KIAA0137, PKU-BETA	
**TLK2**	**kinase**	**yes**	**n**	HGNC:11842	PKU-ALPHA, MGC44450, HsHPK	
**TP53RK**	**kinase**	**no**	**n**	HGNC:16197	PRPK, dJ101A2.2, prpk, Nori-2p, BUD32, C20orf64, Nori-2, PRPKdj101A2	
**TRIB1**	**pseudokinase**	**no**	**n**	HGNC:16891	C8FW, GIG2, TRB1, GIG-2, SKIP1, TRB-1	
**TRIB2**	**pseudokinase**	**no**	**n**	HGNC:30809	TRB2, GS3955, C5FW	
**TRIB3**	**pseudokinase**	**no**	**n**	HGNC:16228	dJ1103G7.3, TRB3, C20orf97, NIPK, SINK, SKIP3	
**TRIO**	**kinase**	**yes**	**n**	HGNC:12303	ARHGEF23, MEBAS, MRD44, tgat	
**TRPM6**	**kinase**	**yes**	**n**	HGNC:17995	CHAK2, FLJ22628, HMGX, HOMG, HOMG1, HSH	
**TRPM7**	**kinase**	**yes**	**n**	HGNC:17994	CHAK1, LTRPC7, TRP-PLIK, CHAK, ALSPDC, LTrpC-7	
**TRRAP**	**pseudokinase**	**no**	**n**	HGNC:12347	TR-AP, PAF400, Tra1, PAF350/400, STAF40	
**TSSK3**	**kinase**	**yes**	**n**	HGNC:15473	SPOGA3, STK22C, STK22D, TSK3	
**TSSK4**	**kinase**	**yes**	**n**	HGNC:19825	C14orf20, STK22E, TSK-4, TSK4, TSSK5	
**TSSK6**	**kinase**	**yes**	**n**	HGNC:30410	SSTK, FLJ24002, CT72, FKSG82, TSSK4	
**TTBK1**	**kinase**	**yes**	**n**	HGNC:19140	KIAA1855, BDTK	
**TTBK2**	**kinase**	**yes**	**n**	HGNC:19141	KIAA0847, SCA11, TTBK	
**TTN**	**kinase**	**no**	**n**	HGNC:12403	CMPD4, FLJ32040, TMD, CMH9, LGMD2J, MYLK5, CMD1G EOMFC, HMERF	
**TXK**	**kinase**	**yes**	**n**	HGNC:12434	TKL, PSCTK5, BTKL, RLK, PTK4, Rlk	
**TYRO3**	**kinase**	**yes**	**n**	HGNC:12446	BYK; Dtk; RSE; Rek; Sky; Tif; Etk-2	
**UHMK1**	**kinase**	**no**	**n**	HGNC:19683	KIS, Kist, KIST, P-CIP2	
**ULK3**	**kinase**	**yes**	**n**	HGNC:19703	DKFZP434C131, FLJ90566	
**ULK4**	**pseudokinase**	**no**	**n**	HGNC:15784	FLJ20574, REC01035, FAM7C1	
**VRK1**	**kinase**	**yes**	**n**	HGNC:12718	PCH1, PCH1A	
**VRK2**	**kinase**	**yes**	**n**	HGNC:12719		
**VRK3**	**pseudokinase**	**no**	**n**	HGNC:18996		
**WEE2**	**kinase**	**yes**	**n**	HGNC:19684	WEE1B	
**STK424**	**pseudokinase**	**no**	**n**	gene bank Acc. No XM_375602		
